# Genetic and Epigenetic Profiling Reveals EZH2-mediated Down Regulation of OCT-4 Involves NR2F2 during Cardiac Differentiation of Human Embryonic Stem Cells

**DOI:** 10.1038/s41598-017-13442-9

**Published:** 2017-10-12

**Authors:** Varsha Pursani, Prasad Pethe, Mohsin Bashir, Prabha Sampath, Vivek Tanavde, Deepa Bhartiya

**Affiliations:** 1 Stem Cell Biology Department, ICMR- National Institute for Research in Reproductive Health, Mumbai, 400012 India; 2 Department of Biological Sciences, Sunandan Divatia School of Science, NMIMS University, Mumbai, 400056 India; 3 Institute of Medical Biology, Agency for Science Technology & Research (A*STAR), Singapore, 138648 Singapore; 4 Bioinformatics Institute, Agency for Science Technology & Research (A*STAR), Singapore, 138671 Singapore; 5 Division of Biological & Life Sciences, School of Arts & Sciences, Ahmedabad University, Ahmedabad, 380009 India

## Abstract

Human embryonic (hES) stem cells are widely used as an *in vitro* model to understand global genetic and epigenetic changes that occur during early embryonic development. In-house derived hES cells (KIND1) were subjected to directed differentiation into cardiovascular progenitors (D12) and beating cardiomyocytes (D20). Transcriptome profiling of undifferentiated (D0) and differentiated (D12 and 20) cells was undertaken by microarray analysis. ChIP and sequential ChIP were employed to study role of transcription factor NR2F2 during hES cells differentiation. Microarray profiling showed that an alteration of about 1400 and 1900 transcripts occurred on D12 and D20 respectively compared to D0 whereas only 19 genes were altered between D12 and D20. This was found associated with corresponding expression pattern of chromatin remodelers, histone modifiers, miRNAs and lncRNAs marking the formation of progenitors and cardiomyocytes on D12 and D20 respectively. ChIP sequencing and sequential ChIP revealed the binding of NR2F2 with polycomb group member EZH2 and pluripotent factor OCT4 indicating its crucial involvement in cardiac differentiation. The study provides a detailed insight into genetic and epigenetic changes associated with hES cells differentiation into cardiac cells and a role for NR2F2 is deciphered for the first time to down-regulate OCT-4 via EZH2 during cardiac differentiation.

## Introduction

The successful isolation of human embryonic stem (hES) cells in the late nineties^1^ followed by the first report of their differentiation into cardiac cells^2^ led to several efforts to design new protocols for their efficient differentiation into cardiac lineage including the current protocols that concentrate upon directed differentiation mimicking the *in vivo* signalling pathways during cardiac development. Pre-clinical animal studies undertaken over a decade involving transplantation of hES derived cardiomyocytes have also shown considerable promise. Studies describing differentiation and outcome of transplantation using hES cells derived cardiac cells are compiled as Supplementary Table 1. However questions have been raised regarding the integrity and maturity of differentiated counterparts^3^. In order to address these questions, it is necessary to understand stage-wise gene expression during early cardiac development and hES cells serve as an excellent model to study this differentiation *in vitro*.

In addition to various signalling cascades and transcription factors, epigenetics is another player involved in major functional genomics decisions^4,5^ and plays an important role when pluripotent hES cells become committed. Chromatin modification is one of the fundamental epigenetic mechanisms associated with active or repressed state of gene expression in a cell^6–8^. Active states are marked by methyl modifications like H3K4me3 and H3K36me3 brought about by Trithorax group (TrxG) of proteins^9,10^ while repression is catalysed by addition of H3K27me3 by Polycomb group (PcG) of proteins^11^ grouped as Polycomb Repressive Complex 1 (PRC1) (RING1A, RING1B, BMI1) and Polycomb Repressive Complex 2 (PRC2) (SUZ12, EZh2, EED)^12,13^.

Various efforts are now focussed to characterize the ES cells and their differentiated progeny with respect to epigenetic mechanisms. Importance of PcG proteins for differentiation of ES cells and not in their self-renewal was identified when the ES cells deficient for PRC2 proteins (SUZ12, EZH2, JARID2) could still be maintained in their undifferentiated state *in vitro* ^14,15^. Dynamics of PcG proteins during differentiation of human ES cells into the three lineages was for the first time reported by our group^16,17^. Transcripts of both PRC1 and PRC2 were found up regulated during endodermal differentiation while during ectodermal differentiation only PRC1 transcripts were found to be up regulated. On the other hand, both PRC1 and PRC2 were down regulated during mesoderm lineage formation *in vitro*. A study by Murry’s group determined the evolution of chromatin modifications like H3K4me3 and H3K27me3 along cardiac lineage formation wherein the dynamic acquisition of H3K4me3 and H3K27me3 by various transcription factors during differentiation at the five different time points was identified^18^. Bruneau and colleagues^19^ also revealed a unique epigenetic pattern of H3K27ac, H3K4me1, H3K4me3 and H3K4me3 during the gene expression changes of cardiomyocytes differentiation *in vitro*.

All these emerging evidences of requirement of unique pattern of epigenetic mechanisms during lineage specification indicate that the disturbed or faulty epigenetic program may contribute to various cardiac disorders. Mutations in a number of histone methyltransferases like JMJD2A, H3K36me3 methyltransferase SETD2, H3K4me3 methyltransferase MLL2, H3K27me3 methyltransferase EZH2 are implicated in congenital heart disorders^20–23^. Though much of the research has focused on providing key conclusions regarding genetic and epigenetic regulatory mechanisms, we are still left with key questions like how are only the right genes expressed or repressed in a given cell type? How does the gene activation or repression machinery gain or keeps its access into the cell? In the present study, we aim to understand the gene expression and histone modification pattern during cardiac differentiation from hES cells using directed differentiation protocol.

## Results

### Cardiac directed differentiation of human ES cells

In-house derived hES cell line (KIND1) was subjected to cardiac differentiation using a directed differentiation protocol (Supplementary Figure 1) and distinct morphological changes were observed while they differentiated into the cardiac lineage (Supplementary Figure 2). Differentiation of KIND1 cells into the cardiac lineage was characterized by the expression of transcripts specific for cardiac progenitors and cardiomyocytes by qRT-PCR (Supplementary Figure 3).

### Microarray Analysis and Data Validation

Dynamics at the mRNA and chromatin level was studied using undifferentiated KIND1 cells (D0), cardiovascular progenitors (D12) and beating cardiomyocytes (D20). A list of genes (expected and predicted) was generated showing a fold change value of >=2.0 and a p-value score < 0.05. Comparisons were made amongst three groups including D0 versus D12, D0 versus D20 and D12 versus D20.

### Gene expression analysis

#### D0 versus D12

**Figure 1:**
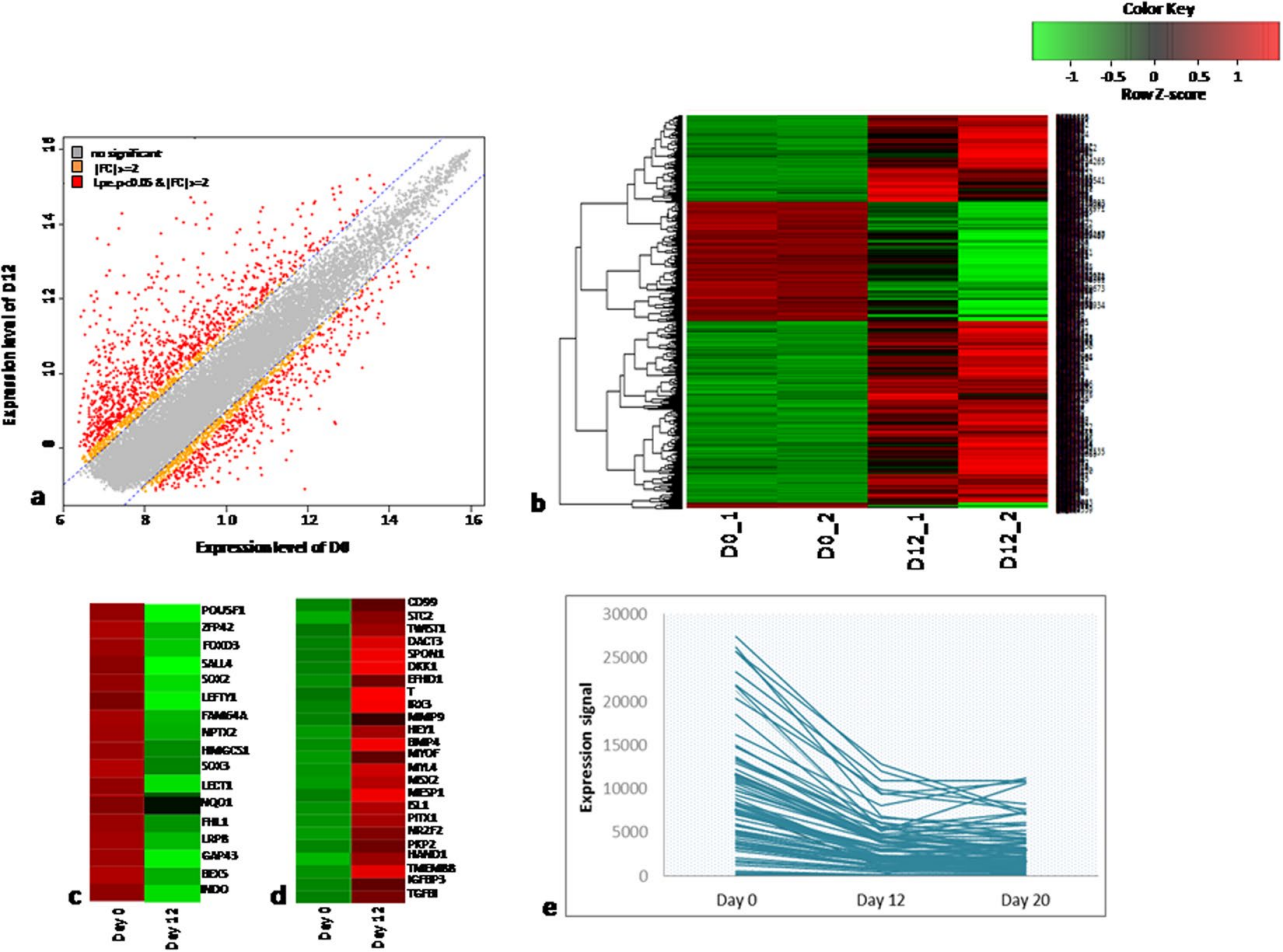
Differential gene expression profiling of D0 vs D12. (**a**) Plot of expression levels between D0 and D12. (**b**) Significant hierarchical clustering to show the clustering of the 2 biological replicates. (**c**) Heat map for the significant genes expressed on D0 and down regulated at D12. (**d**) Heat map for the significant genes not expressed at D0 and up regulated at D12. (**e**) Graphical plot for the expression levels of genes differentially expressed during conversion of D0 to D12 cells.

About 1400 genes were found to be differentially expressed when KIND1 cells (D0) differentiated into cardiovascular progenitors (D12) (Fig. 1a; Supplementary Tables 2 and 3). A heat map was generated to indicate the shift of gene expression from undifferentiated to differentiated state of hES cells (Fig. 1b). Out of the total 470 genes that were down-regulated, we analysed the genes that decreased significantly on D12 (Fig. 1c,e). This group included widely studied pluripotency genes (OCT4 also referred to as POU5F1, STAT3, ZFP42, HMGA2, SOX2, TCF3, DPPA4, LEFTY1, NANOG, FOXD3 and SALL4). Genes associated with neurogenesis and synaptic transmission (OTX2, TUBB4, EGR2, STMN3, SOX1, NPTX2, NQO1) and those essential for chondrogenesis, steroid biosynthesis, reproductive developmental processes, and regulation of cellular component size, cell motility and gliogenesis respectively (HMGCS1, ADM, SOX3 and GAP43) were expressed at D0 and down regulated on D12. Additionally some functionally unknown genes (VGF, ABCB6, AIG1, BEX5, NRIP3, MFGE8) were also found to be expressed on D0 and not on D12. On the other hand, about 1000 transcripts were found to be up regulated on D12 (Fig. 1d). These included CD99, CADM1, RHOU, SPON1, LRP2, STC2, PSTPIP2, PTPRM, CDX2, PITX2 that are known to be associated with cell adhesion, cell-cell signalling, cytoskeleton organization, cell migration, and regulation of transcription. Along with these, other genes like CXCR7, AFP, TFPI, BMPER, APOA2, MMP9, DCN required for extra cellular matrix organization; LEF1, BMP4 for mesoderm development; IRX3, FOXF1, AMOT, NR2F2, HAND1, HEY1, LECT1 essential for blood vessel development and angiogenesis; MSX2, T, MESP1, ISL1, MYL4, EFNB2, MYOF required for heart morphogenesis and vasculature development; SMAD6, ANXA1, IFI6 important for negative regulation of apoptosis; MSX1, HOXB5, PDGFRB, TWIST1, WNT5A, TNC, BMP5, BMPR2 playing role in skeletal system development, bone mineralization and blood circulation were also up regulated on D12. These genes were used to characterize the cardiac progenitors. All the above listed genes had their peak expression at D12. Additionally some novel genes yet unreported were also found to be expressed at D12 that might be essential for mesoderm or cardiac differentiation like DACT3, PKDCC, EPSTI1, GPR177, ANKRD38. Genes like FHL1, STMN3, DCLK1, INSIG1, ADM, PIM2 playing roles in metabolic processes, regulation of cell component size and organization, ER signalling pathway and apoptosis were expressed in both undifferentiated cells and progenitors.

#### D0 versus D20

**Figure 2:**
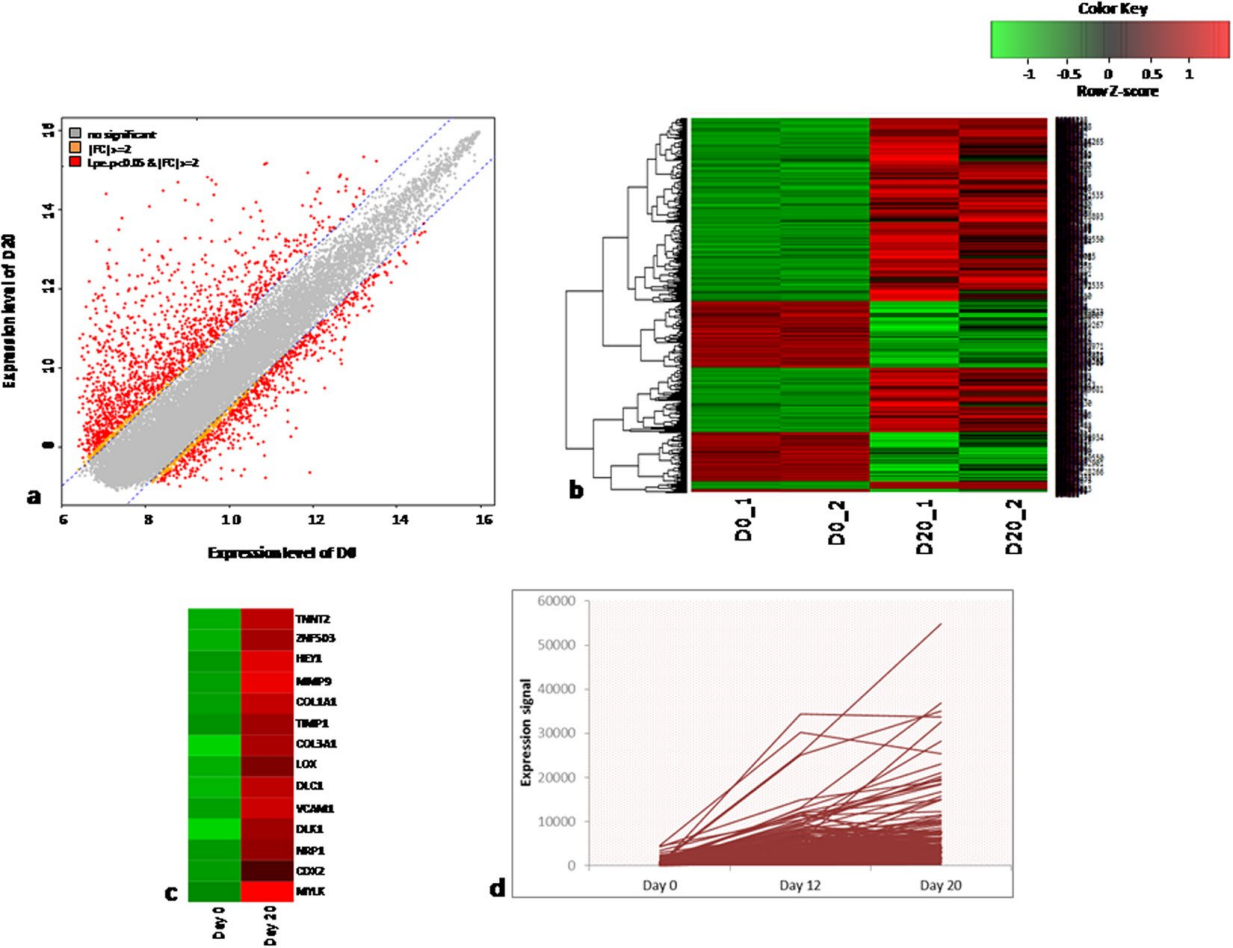
Differential gene expression profiling of D0 vs D20. (**a**) Plot of expression levels between D0 and D20. (**b**) Significant hierarchical clustering to show the clustering of the 2 biological replicates. (**c**) Heat map for the significant genes not expressed at D0 and up regulated at D20. (**d**) Graphical plot for the expression levels of genes differentially expressed during conversion of cells at D0 to D20.

Similarly about 1900 genes were differentially regulated when gene expression profiles of D0 versus D20 were compared of which 1300 genes were up-regulated and 600 genes were found to be down regulated (Supplementary Tables 4 and 5). Genes like TNNT2, ZNF503, HEY1, previously reported to be associated with cardiac structure and function were highly expressed in D20 cardiomyocytes compared the undifferentiated KIND1 cells. Along with the genes required for cardiomyocytes differentiation, genes like MMP9, COL1A1, TIMP1, COL3A1, LOX, DLC1, VCAM1, DLK1, NRP1, CDX2, MYLK were also up regulated that are known to be associated with mechanisms like cell adhesion, skeletal system and blood vessel development required for cardiac specification and maturation. In addition to pluripotency genes like OCT4, NANOG various genes like SOX3, INDO1, HBA2, PTPRZ1, NPTX2, OTX2, PMAIP1, OLFM1, NQO1 that are known to be associated with ectodermal processes like nervous system and retinal development and endodermal processes like hepatic or lung development^24–26^ were significantly down-regulated further indicating the specific differentiation of mesodermal lineage in our culture (Fig. 2).

#### D12 versus D20

**Figure 3:**
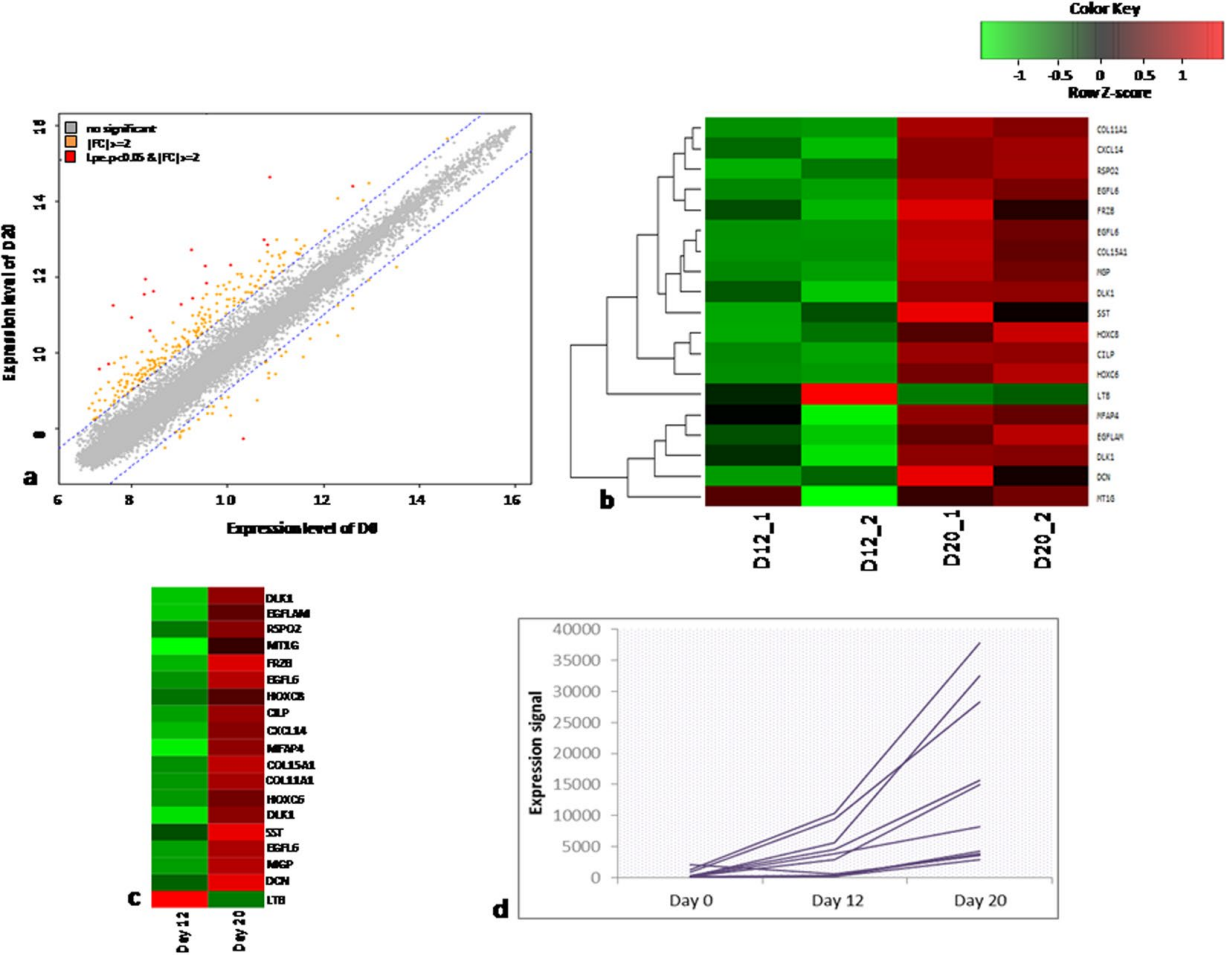
Differential gene expression profiling of D12 vs D20. (**a**) Plot of expression levels between D12 and D20. (**b**) Significant hierarchical clustering to show the clustering of the 2 biological replicates. (**c**) Heat map for the significant genes not expressed at D12 and up regulated at D20. (**e**) Graphical plot for the expression levels of genes differentially expressed during conversion of cells at D12 to D20.

Comparing cardiovascular progenitors and beating cardiomyocytes, only 19 genes were found to be differentially regulated wherein 18 were up-regulated and only a glycoprotein gene LTB was down-regulated (Fig. 3; Supplementary Tables 6 and 7). Genes like DLK1, EGFL6, MFAP4, SST, MGP and DCN were among the up-regulated transcripts and were expressed in the cardiomyocytes. Terminal cardiomyocyte formation was indicated by expression of DLK1, EGFL6 and FRZB that are known to be involved in regulation of cell growth and developmental processes^27–29^. MFAP4 is associated with calcium dependent cellular adhesions and intercellular interactions that are essential for cardiac tissue^30^. MGP is known to be responsible for inhibition of bone formation and is expressed in aorta and aortic valves while DCN related to TGFB pathway has a role in proliferation and angiogenesis. Looking at the expression pattern on D12 and D20, as expected, matrix-associated genes like COL5A1, CILP, and COL11A1 were expressed on D20.

### Gene ontology and signalling pathways involved

Functional validation of the genes enriched significantly (i.e. FC >=2.0 and P value < 0.05) on days 0, 12 and 20 of cardiac differentiation protocol was performed by gene ontology study. Genes enriched during the differentiation of undifferentiated cells (D0) into cardiac progenitors (D12) were found to be associated with biological processes related to early mesoderm and cardiac derivatives formation like ‘extracellular region development’, ‘vasculature development’, ‘regulation of cell proliferation’, ‘skeletal system development’, ‘embryonic morphogenesis’ (Supplementary Figure 6A). On the other hand the significant gene ontologies noted when D0 hES cells were subjected to cardiomyocytes at D20 included biological processes like ‘cell adhesion’, ‘ECM structural constituent development’, ‘blood vessel development’, ‘localization of cell’, ‘calcium ion binding’, ‘heart development’. Similarly while looking at the processes altered during the conversion of cells from D12 to D20, they were mostly related to the maturation of the progenitors like ‘proteinaceous ECM development’, ‘response to mechanical stimulus’, ‘extra cellular structural organization’, ‘extra cellular matrix binding’. The above observations confirm the formation of or an obvious shift of undifferentiated hES cells to cardiac progenitors to cardiomyocytes. Moreover, the ontology analysis was extended to look for the molecular functions associated with the enriched group of genes (Supplementary Figure 6B).

We next sought to identify the signalling pathways involved during the differentiation of human ES cells into cardiac lineage. Pathway analysis was performed using Biocarta and KEGG Pathway Database on the genes enriched during each of the differentiation stages (Supplementary Figure 7). ECM-receptor interaction regulating cellular processes like transcription was the most expressed functional pathway on D12 and D20 as compared to D0. The results displayed the enrichment of BMP4 pathway at all the differentiated stages, as the pathway is necessary for the stem cell self-renewal and differentiation into cardiomyocytes both *in vivo* and *in vitro*. Activated BMP4 pathway was also evident by the significant up regulation of the MAPK signalling pathway necessary for the cell proliferation and differentiation. WNT pathway is another crucial pathway essential for cardiac specification and differentiation of cardiomyocytes. Interestingly our results demonstrate the increased expression of WNT antagonists DKK1, SFRP1, SFRP2, NOTUM, SOSTDC1 and IGFBP4 at progenitor stage that were essential to inhibit the WNT pathway in order to specify the cardiac mesoderm leading to the formation of progenitors. Further the expression of markers like VIMENTIN, TENASCIN C, SNAI2 representing the EMT transition revealed the activated TGF beta pathway during cardiac lineage differentiation. Additionally signal transduction pathways that mediate cell-to-cell communication involving receptor tyrosine kinase family like platelet derived growth factor receptors, vascular endothelial growth factor receptors, transforming growth factor beta receptors that are known to be active during mammalian development were also identified.

Next, we extended our analyses to understand the epigenetic status underlying along with the varied gene expression pattern during the formation of cardiac progenitors and cardiomyocytes.

### Chromatin modifiers during cardiac differentiation

**Figure 4:**
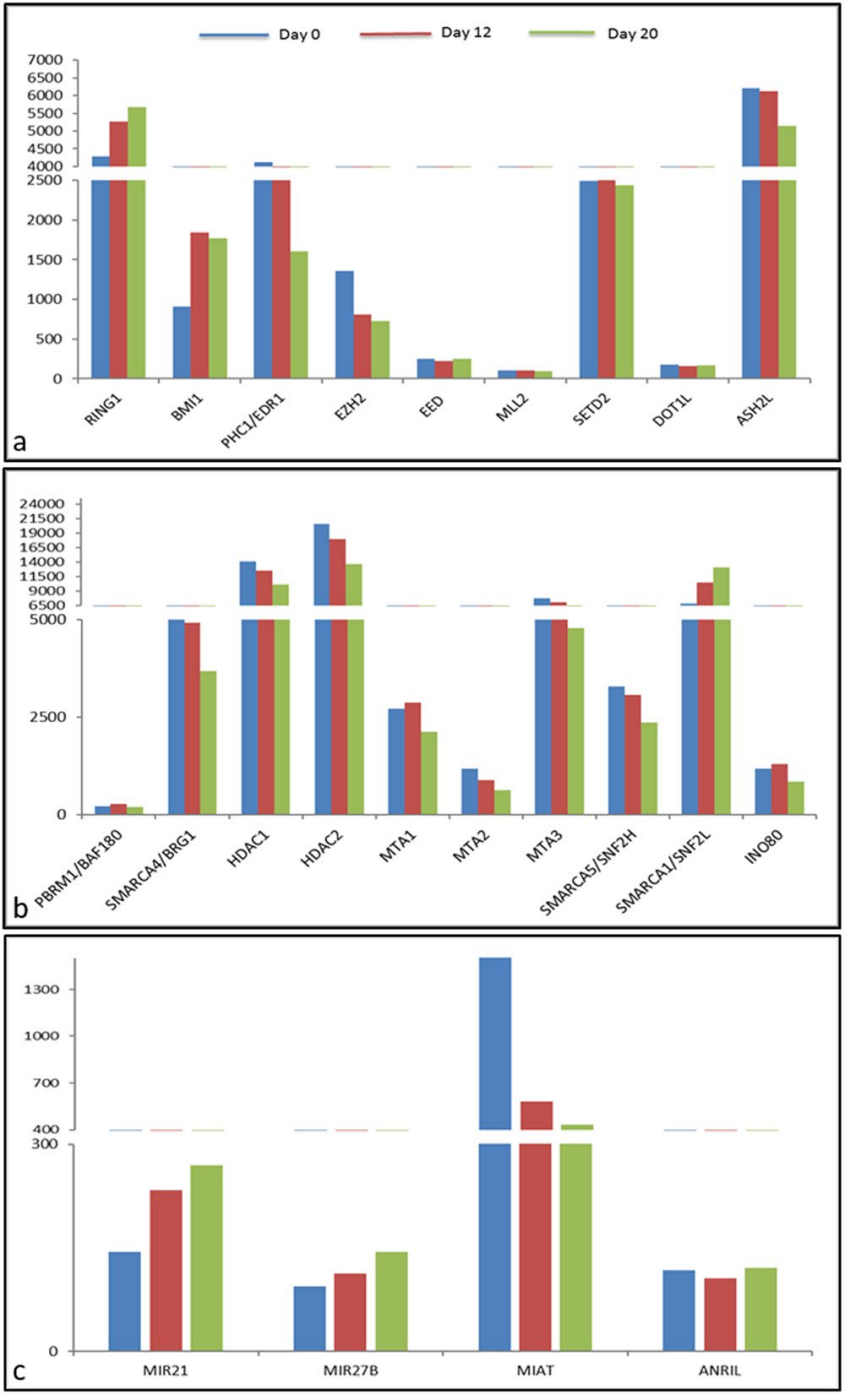
Expression of chromatin modifiers. Epigenetic analysis to identify the upregulated modifiers including histone modifiers (**a**), Chromatin remodelers (**b**) and non-coding RNAs (**c**) during different levels of cardiac differentiation.

Among the various histone modifiers, we concentrated upon the Polycomb and Trithorax group of proteins (PcG and TrxG) as they represent the key players, the repressors and activators of gene expression respectively. Various studies till date have reported the importance of PcG proteins and that the abolishment of PcG proteins like EZH2, EED or SUZ12 disrupts the stem cell identity and their developmental potential. ES cells in their undifferentiated state maintain the bivalent marks in order to keep the developmental genes poised for further activation. In consistence with all the reports, the core proteins EZH2 and MLL2 forming the bivalent marks (H3K27me3 and H3K4me3 respectively) were found expressed in undifferentiated hES cells. As the differentiation proceeded to progenitors and cardiomyocytes, expression of EZH2 began to down-regulate. Expression of MLL2 core component ASH2L was expressed in both cardiac progenitors and cardiomyocytes. Another PcG group proteins like RING1, BMI1, PHC1, that represent the Polycomb repressive Complex 1 (PRC1) group, were up-regulated while the PRC2 group like SUZ12 and EED were significantly down-regulated in progenitor and cardiomyocyte stages as compared to their status in the undifferentiated state hES cells. Histone methyltransferase expressed during the differentiation is the SET domain containing 2 or SETD2 that is involved in the activation of gene transcription and is reported to be crucial for the cardiac formation^21^ (Fig. 4a). BRG1 and BAF180 are essential remodelling enzymes for cardiac development that regulate the differentiation and EMT transition respectively, thus required during early differentiation into cardiac lineage. We found BAF180 up regulated at the differentiation of progenitor and down regulated later, while BRG1 was up regulated in undifferentiated and progenitors. Another remodeler from the NuRD complex Methyl CpG binding domain or MBD3 or DPEP3 that is known to bind to the active genes was found to decrease with differentiation. HDAC1 and HDAC2 decreased with differentiation increasing the acetylation levels for gene activation. In addition, enzymes like MIAT 1, MIAT2 and MIAT3 were also differentially expressed suggesting a role during differentiation. Genes like SMARCA5 and SMARCA1 that represent ATPases and regulate the chromatin structure and transcription were also expressed during differentiation (Fig. 4b).

### Non-coding RNAs during differentiation

miRNAs and lncRNAs represent a valuable tool to understand the role of cellular proteins. As important regulators of gene expression at the epigenetic level, they activate or repress the transcription. Cardiac formation machinery also includes the number of non-coding RNAs as regulators of gene activation and repression. Among the number of miRNAs identified to be differentially regulated, the miRNAs significantly changed were miR21, miR208a, miR423 and miR27b that have been shown to regulate endothelial and myogenic differentiation. Expression was found increased with the differentiation of hES cells in cardiomyocytes. Similarly, the long non coding RNA represents non-coding ability and act as regulators of development and pathophysiology. ANRIL, CDKN2A/2B and MIAT were among the many lncRNAs identified in our study that might be involved in lineage specific gene expression (Fig. 4c).

### Microarray data validation

**Figure 5:**
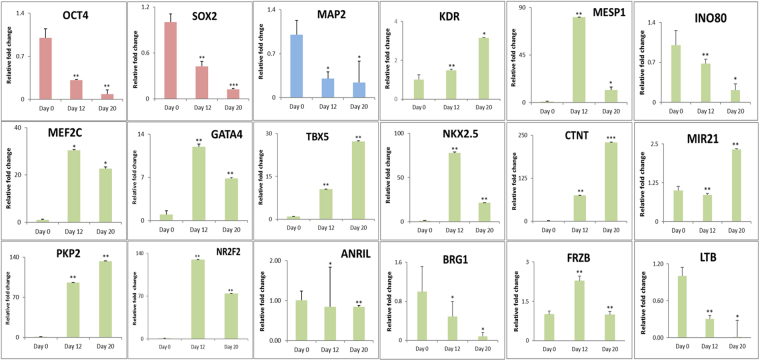
Validation of Microarray data by qRTPCR. qRT-PCR validation for the crucial genes like OCT4, SOX2 (pluripotency) (red), MAP2 (ectodermal) (blue), KDR2, MESP1, MEF2C, GATA4, TBX5, NKX2.5, CTNT, PKP2, FRZB (mesodermal- cardiac), LTB, INO80, BRG1, MIR21 and ANRIL (epigenetic machinery) (green) in the D0, D12 and D20 cells profiled by microarray. Error bars represent ± SEM, statistical significance represented as *(P < 0.5), **(P < 0.01), ***(P < 0.001).

Differential regulation of specific gene transcripts was studied by qRT-PCR to validate the microarray results. This included specific transcripts for pluripotent genes (OCT4, SOX2), ectodermal (MAP2), mesodermal into cardiac lineage (KDR2, MESP1, MEF2C, PKP2, GATA4, TBX5, NR2F2, NKX2.5, CTNT and FRZB), LTB, along with transcripts representing epigenetic machinery (INO80, BRG1, MIR21 and ANRIL). The results were found consistent with the microarray data (Fig. 5). We also characterized the cardiac progenitors on D12 and cardiomyocytes obtained on D20 by studying expression of NKX2.5 (Supplementary Figure 8A) and CTNT (Supplementary Figure 8B) by immuno-fluorescence.

The extensive genetic and epigenetic characterization of KIND1 cells during differentiation into cardiac lineage, by microarray along with the qRT-PCR validation depicts the dynamic expression of various transcripts during cardiac differentiation. Though the array and the qRT-PCR validation involved differences in the expression levels, the technique reveals the global view of the genes along with illuminating the differences in their expression within the cells and their differentiated counterparts. Transcription factor NR2F2 was further studied as it showed increased expression during cardiac both by microarray and qRT-PCR.

### NR2F2, EZH2 and OCT4 were reciprocally expressed

**Figure 6:**
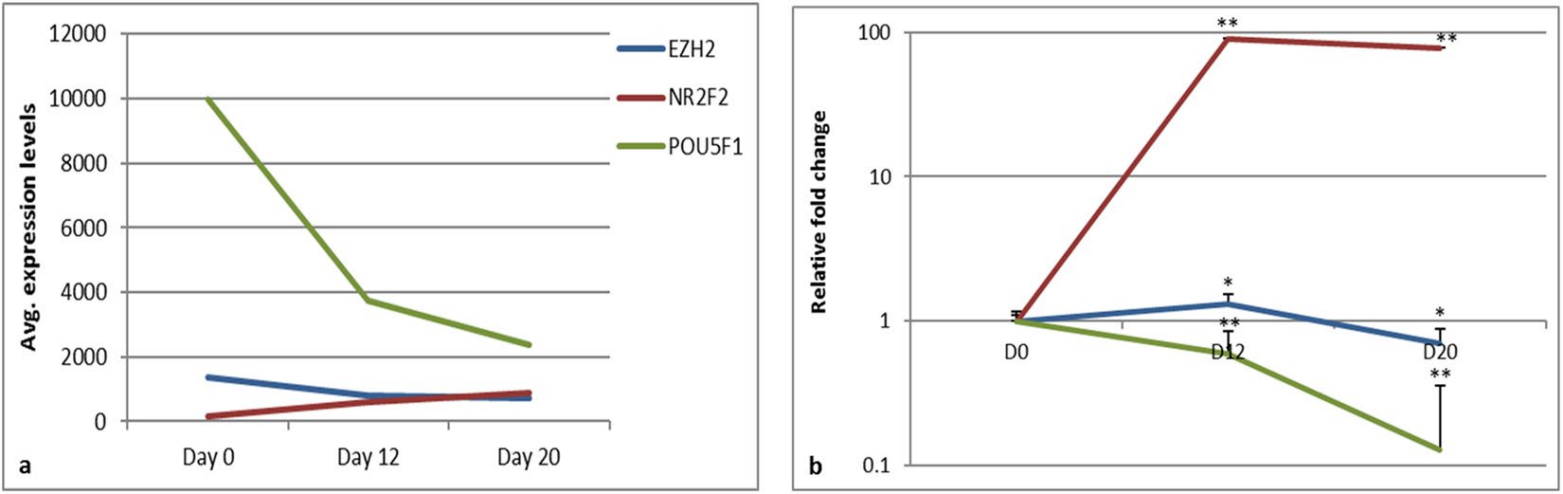
Expression pattern of EZH2, NR2F2 and OCT4. Relative expression levels of EZH2 (blue), NR2F2 (red) and OCT4 (green) by microarray (**a**) and qRT-PCR (**b**) during differentiation. Error bars represent ± SEM, statistical significance represented as *(P < 0.5), **(P < 0.01), ***(P < 0.001).

Among the various up regulated transcription factors, NR2F2, well studied in the context of neural differentiation showed increased expression in cardiac progenitors and cardiomyocytes. Processes reported to involve NR2F2 include development of atrial septal defects^31^, transcriptional regulation of OCT4 in mouse embryonal carcinoma cells as well as in hES cells undergoing neuroectoderm development^32,33^. NR2F2 is also crucial during mice heart development for angiogenesis and coronary vessel formation^34,35^. We thus interrogated the expression of NR2F2 in our differentiation system to evaluate whether it acts as regulator of early cardiac differentiation. The expression levels of NR2F2 (days 0, 12 and 20) were found to be inversely related to that of OCT4 (a crucial marker for pluripotent state) and were comparable to EZH2, a transcriptional repressor as observed both by microarray analysis and qRT-PCR validation (Fig. 6). In support to the previous studies, the inverse correlation of NR2F2 and OCT4 possibly suggest the involvement of NR2F2 during initiation of differentiation.

### ChIP sequencing

**Figure 7:**
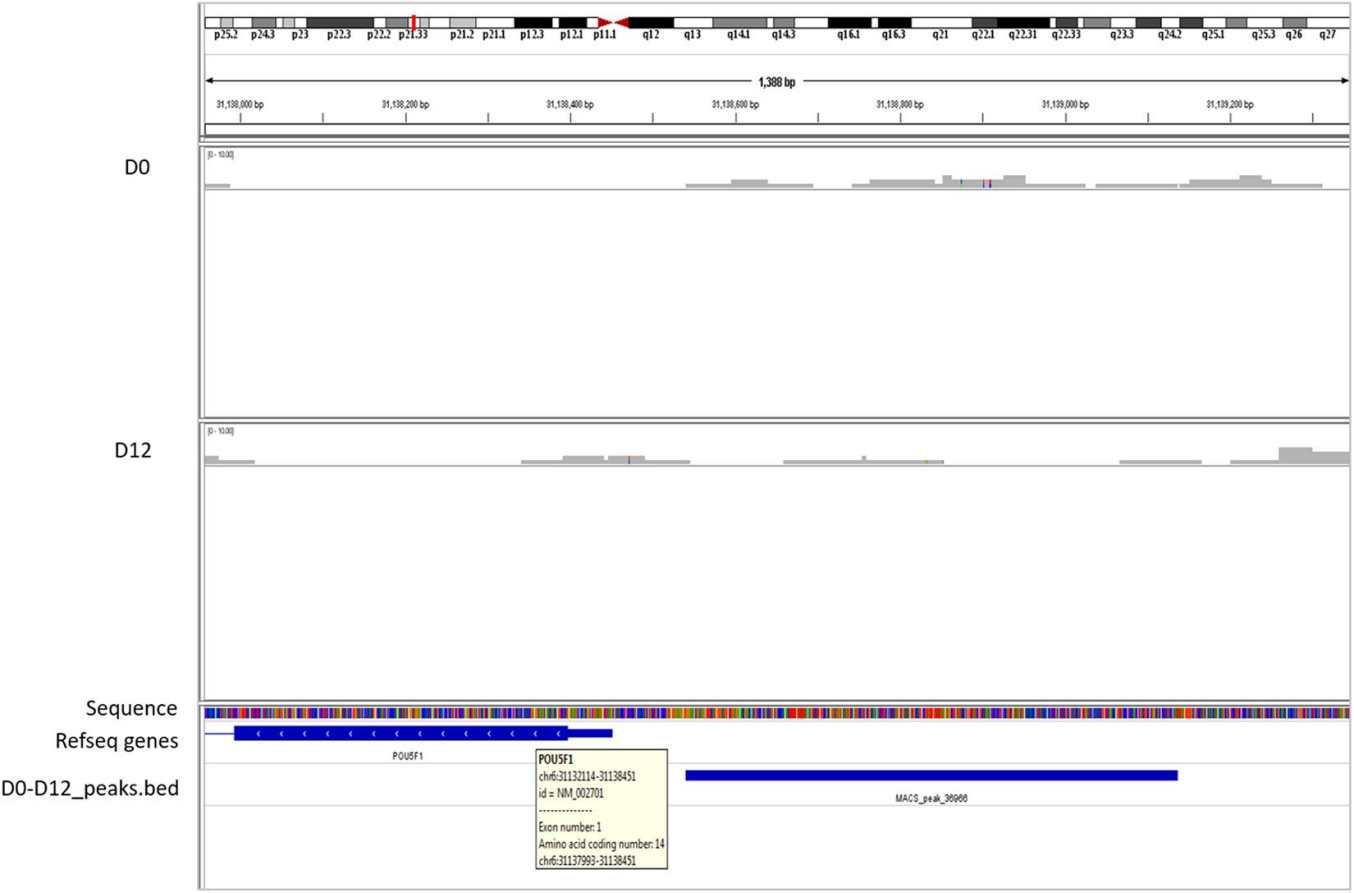
Distribution of H3K27me3 mark on OCT4. Binding profile of H3K27me3 mark on the OCT4 gene during cardiac differentiation generated by IGV genome browser mapped to human hg 19 genome.

H3K27me3 is a representative repressive mark occurring as a part of poised bivalent domain in undifferentiated hES cell state^36,37^ and its levels increase during differentiation^7^. Similarly it promotes the cardiomyocyte formation from ES cells by repressing the unnecessary transcriptional programs^23,38^. OCT4 is among the various genes during cardiac formation that lose the active mark with a gradual gain of repressive H3K27me3 mark^19^. On the other hand, elevated levels of OCT4 have been shown to lead to meso-endodermal cell fate of undifferentiated hES cells^39,40^. This underscores the need to study the mechanism of occurrence of H3K27me3 mark on OCT4 transcription factor during mesodermal differentiation. Towards this, we first spotted the occurrence of H3K27me3 mark on the OCT4 promoter during cardiac progenitor and cardiomyocyte formation by ChIP using anti-H3K27me3 followed by sequencing. As expected the H3K27me3 peaks were found to be absent in undifferentiated D0 hES cells while progressive differentiation towards the cardiac progenitor formation led to the appearance of H3K27me3 peak on the OCT4 promoter subjecting the cells for proper differentiation into cardiomyocytes (Fig. 7).

**Figure 8:**
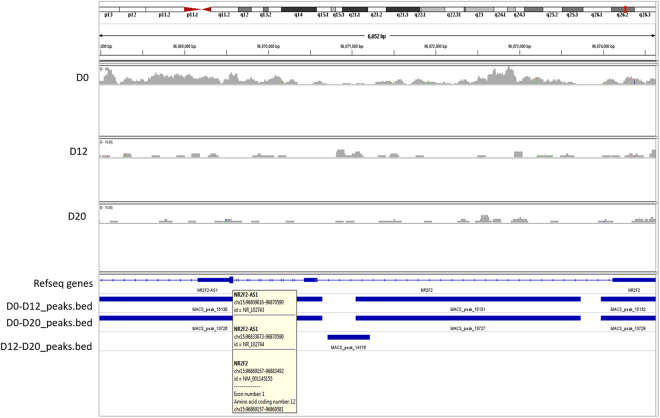
Distribution of H3K27me3 mark on NR2F2. Occupancy of H3K27me3 mark on the NR2F2 locus at days 0, 12 and 20 generated by IGV gene browser mapped to human hg 19 genome.

We then focused on the promoter of transcription factor NR2F2 during cardiac differentiation to understand the presence of H3K27me3. Existence of peaks on NR2F2 promoter in D0 hES cells revealed the poised status of the gene. Differentiation into cardiac progenitors and cardiomyocytes was guided with the resolution of the peaks from the NR2F2 promoter indicating the actively expressed state of the gene further confirming the need of NR2F2 during early cardiac formation (Fig. 8).

### EZH2 binds to NR2F2 and OCT4

**Figure 9:**
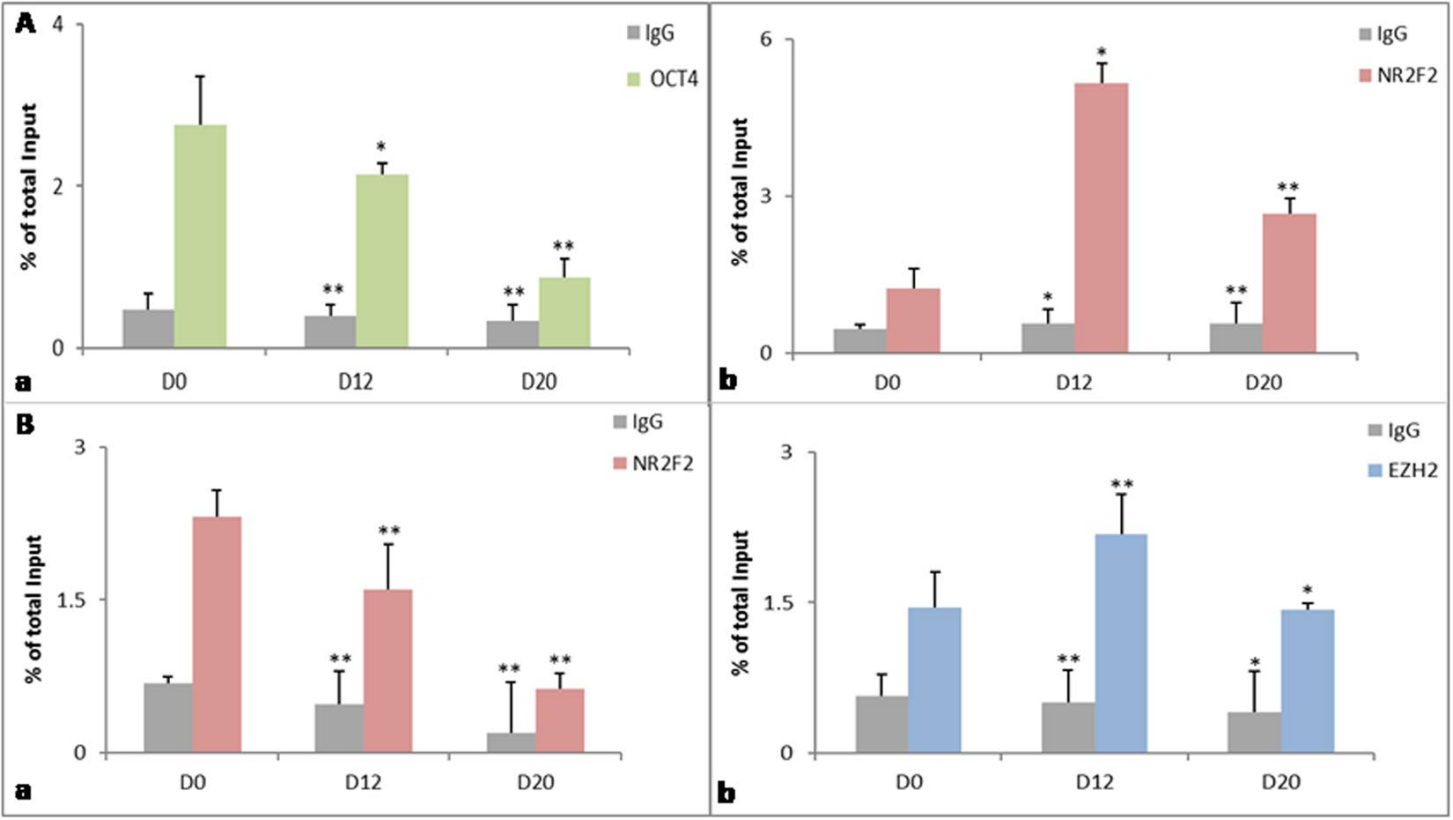
ChIP and sequential ChIP. (**A**) Chromatin immuno-precipitation with anti-EZH2 followed by qPCR with NR2F2 (a) and OCT4 (b). The binding analysis shows that EZH2 binding increases on the NR2F2 promoter at D12 that later decrease at D20 while on the OCT4 promoter the EZH2 binding is maintained with differentiation (**B**) EZH2-OCT4 sequential ChIP followed by qPCR for genes EZH2 (a) and NR2F2 (b). The experiment confirms the c0-binding of EZH2 and OCT4 on the NR2F2 promoter at D12 that decreases slightly at D20. Values in both ChIP and sequential ChIP represent the average and standard deviation of two independent experiments and were calculated as percentage of total input. Error bars represent ± SEM, statistical significance represented as *(P < 0.5), **(P < 0.01), ***(P < 0.001).

The prevailing H3K27me3 mark on NR2F2 locus identified by ChIP sequencing raised the possibility of recruitment of EZH2 on the OCT4 promoter by transcription factor NR2F2 for proper cardiac formation. We thus looked for the direct binding of EZH2 and OCT4 by chromatin immuno-precipitation followed by qPCR in undifferentiated as well as in cardiac progenitors and cardiomyocytes (Fig. 9A). The results displayed a progressive inhibition of OCT4 expression with a concomitant augmentation of NR2F2 promoter sequences with differentiation into progenitors and cardiomyocytes (Fig. 9Aa) whereas expression of NR2F2, increased upon differentiation justifying the presence of H3K27me3 mark visible by ChIP sequencing (Fig. 9Ab). Sequential ChIP of the EZH2 immuno-precipitated chromatin with OCT4 antibody revealed further the co-existence of EZH2 and OCT4 onto the NR2F2 gene evident by the expression of NR2F2 by qPCR. The increased expression of NR2F2 on D0 as compared to D12 and D20 suggested its binding with EZH2 and OCT4. Conversely, the differentiation to progenitors was accompanied with the decreased occupancy of OCT4 on NR2F2 gene that further decreased on D20. Expression pattern of EZH2 also was found to be up regulated in progenitors as compared to undifferentiated cells suggesting its involvement in binding to OCT4 along with NR2F2 (Fig. 9B).

## Discussion

Present study provides novel information on the detailed genetic and epigenetic gene expression pattern underlying cardiac differentiation *in vitro* from hES cells and delineates an epigenetic mechanism for repressing pluripotency marker OCT4A by NR2F2 via EZH2 during the process. Transcriptome analysis of undifferentiated hES cells as they transit into cardiac progenitors and cardiomyocytes is shown by microarray analysis. The differential regulation of only 19 genes when cells transit from D12 to D20 was indeed intriguing since compared to D0, almost 1400 genes showed differential expression on D12 and 1900 showed differential expression on D20. This could imply that the cells on D12 and D20 are almost similar with minimal maturation. However, of the 500 genes differentially expressed between D12 and D20, almost 55% were down regulated on D20 when compared to D0 (and not on D12) justifying the formation of mature counterparts on D20 compared to D12. The only down-regulated gene LTB between D12 vs D20 (of the 19 genes) was not included in the down-regulated gene list between D0 and D20. All the 18 genes that were up-regulated on D20 compared to D12 were not expressed on D0. Thus, we believe our results show dramatic maturation of cardiac cells on D20 compared to D12 associated with a change in only 19 genes. Of all the genes being altered during ES cells differentiation, transcription factor NR2F2 reported to repress OCT-4 during neural differentiation^32,33^, has not yet been reported during cardiac differentiation but its mutation is known to cause atrial septal defects^31^. NR2F2 showed up regulation as cells differentiate into cardiac progenitors and cardiomyocytes by both microarray and qRT-PCR (almost 100 folds). A simultaneous down regulation of EZH2 was noted. Further ChIP experiments were undertaken to study whether a cross-talk exists between NR2F2 and EZH2 for repressing OCT-4 during cardiac differentiation. We first observed that the repressive mark H3K27me3 was occupied on NR2F2 promoter on D0 while not in D12 and D20; this explains the increased expression of NR2F2 as differentiation proceeded. The results clearly showed the occupancy of H3K27me3 on OCT4 promoter in progenitor cells on D12 (and not on D0) resulting in repressed OCT4 expression during differentiation. Since this repressive mark is brought about by histone methyltransferase EZH2, we studied its binding to NR2F2 and OCT4 by ChIP and sequential ChIP. QPCR following ChIP displayed the EZH2 binding on both OCT4 and NR2F2 while sequential ChIP further confirmed that NR2F2 was bound to both EZH2 and OCT4. Based on these results, we propose that NR2F2 recruits EZH2 at the OCT4 further leading to its repression during cardiac differentiation from hES cells.

OCT4 gene is reported to generate multiple transcripts that are translated into multiple isoforms of OCT4^41^; OCT4A (true stemness marker containing unique exon 1), OCT4B (non-pluripotent variant) and OCT4B1 (putative stemness marker). In order to understand the mechanisms of hES cells pluripotency, it thus remains essential to concentrate upon the OCT4A isoform (and not total OCT4). Present study is focused on OCT-4A positive ES cells since we used primers specific to OCT4A. Moreover, in the ChIP sequencing studies, the occupancy of H3K27me3 mark on the promoter driving the expression of exon 1 that proves the involvement of ‘pluripotent OCT4 isoform’. Looking at the binding of EZH2 onto the OCT4 promoters, the primer pair specific to exon 1 which amplifies the distinct OCT4A gene further validates our findings, supporting the mechanism of binding of EZH2 and NR2F2 with OCT4A and repressing its expression for leading the hES cells to cardiac differentiation.

Epigenetics adds another layer of gene expression control by modulating the transcriptional activities in a trimly and gene specific manner. The extensively studied epigenetic feature for stem cells is the simultaneous appearance of H3K27me3, H3K4me3 termed as bivalency that holds the equal potential to be expressed or repressed and that are resolved as per the signals and MLL2 enzymes specific for H3K27me3 and H3K4me marks respectively in our undifferentiated hES cells that were later subjected to cardiac lineage formation. However, on examining the cardiac progenitors and cardiomyocytes, EZH2 had a lowered expression in cardiac progenitors and much lower in cardiomyocytes, holding the evidence in support of reported studies that EZH2 is crucial for cardiac development *in vitro* and *in vivo* ^38,42–44^. Epigenetic remodelers found to be expressed were BRG1, MTA family and HDACs known to promote cardiac cell differentiation by regulating the levels of crucial cardiac transcripts like NKX2.5, TBX5 and GATA4^45–48^. Similarly, the expression of other components of epigenetic machinery noted in our study is well studied in cardiac differentiation context; though their specific role is still unknown.

The sole down regulated gene LTB represents a TNF family ligand that majorly acts as the inducer of inflammatory response and is involved in the organization/development of lymphoid tissue^49,50^. Additionally, it has been identified as a direct regulator of vascular endothelial cells by activating the NF-ĸB pathway^51^. With respect to cardiac development, LTB is identified as a risk factor altered for cardiovascular diseases and thus the TNF antagonist potentially acts as a likely candidate to reduce the CVD risk in rheumatoid arthritis patients^52–55^. However, though there exists a dearth in studies reporting molecular mechanisms associated with LTB in cardiac development and pathogenesis, present study reports reduced levels of LTB during differentiation of hES cells into the cardiac lineage.

Number of studies till date have reported the transcriptional profiling of ES derived beating clusters^55–57^ or the endothelial cells^58^ at various stages of differentiation and the data was also compared with the transcriptional pattern in fetal and adult heart *in vitro*. However, majority of these have employed spontaneous embryoid body formation technique to obtain beating clusters that led to achieving the mixed population comprising of all the lineages. Secondly, the purifying techniques like percol gradient resulted in purified cardiac population of as low as 40%^56^. Third employed way was studying the cardiomyocytes differentiated from ES cells that were co-cultured onto the layer of END2 cells or inactivated mouse fibroblast feeders^57,59^. Despite the extensive characterization data and their comparison with the *in vivo* counterparts is available, the fact that the resulting population being studied contains the non-mesodermal population still remains. Our study on the other hand employs the directed differentiation approach that ensured the cardiac cell fate of majority of the population. Moreover, the protocol also allows studying the step wise formation of cardiomyocytes involving early nascent mesoderm, cardiac mesoderm formation followed by cardiac progenitors and beating cardiomyocytes. The protocol from the very beginning restricts the formation of ectodermal and endodermal population excluding the endodermal genes reported to be essential for mesodermal direction.

To conclude, detailed transcriptomic expression of genetic and epigenetic factors in pluripotent hES cells and during their conversion into cardiac progenitors and cardiomyocytes is reported. With a view to identify a role of NR2F2 during this differentiation, we also show the expression pattern of H3K27me3 mark on NR2F2 and OCT4 genes. Limitations of the study include use of unsorted cardiac population which otherwise would bring much clearer picture of cardiac differentiation mechanisms. Secondly, the deficiency of more confirmations like loss of function tests with respect to NR2F2 and OCT4 both *in vivo* and *in vitro* that would provide deeper insights. Despite this, our study for the first time provides the mechanism of OCT4 repression by the combined actions of NR2F2 and EZH2 that would direct the future efforts to undertake more specific experiments towards understanding cardiac development. Analysing various histone modifications and their dynamics along with the lineage specific gene expression patterns could uncover the regulatory genes involved during early human cardiac development *in vitro*.

## Materials and Methods

In-house derived human embryonic stem cells (KIND1) derived on human feeders^60^ were transitioned to feeder-free condition^17^ and used to differentiate into tripotent cardiac progenitors. Similar protocols (described in details in supplementary section) were used in the present study to obtain undifferentiated hES cells (D0), cardiovascular progenitors (D12) and beating cardiomyocytes (D20) for various studies.

### Microarray Analysis

Microarray was performed using two biological replicates of each time point using Illumina, HT-12 v4.0 platform commercially. hES cells on days 0, 12 and 20 were harvested by mechanical dislodging using a cell lifter in TRIzol reagent (Invitrogen, Carlsbad, CA, USA) for RNA extraction as per manufacturer’s instructions. Genome-wide gene expression analysis was performed commercially using Illumina, HT-12 v4.0 platform (Bencos Research Solutions, Mumbai). Gene-Enrichment and Functional Annotation analysis for significant probe list was performed using DAVID Bioinformatics Resources (http://david.abcc.ncifcrf.gov/home.jsp). All data analysis and visualization of differentially expressed genes was conducted using R 3.1.2 (www.r-project.org). Genes with fold values between −2 and +2 were omitted from the analysis. Three combinations of data were analysed, namely – Day 0 Vs Day 12, Day 0 Vs Day 20 and Day 12 Vs Day 20. QRT-PCR analysis for pluripotent (OCT4A, SOX2), cardiac specific markers (KDR2, GATA4, MESP1, MEF2C, TBX5, PKP2, NKX2.5, CTNT) including LTB and other epigenetic machinery (INO80, BRG1, MIR21 and ANRIL) validated microarray data. Human fetal and adult cardiac total RNA (DSS Takara Bio India, New Delhi, India) were used to compare the maturity of the differentiated cells and GAPDH was used as an internal control. All the primers used in the study are mentioned in Table 1. Microarray data was also validated at protein level by studying the immuno-expression of NKX2.5 and CTNT by confocal microscopy. Detailed microarray data validation is provided in Supplementary section.

**Table 1:** Primer sequences of genes in the study.

**Gene**	**Primer sequence 5′-3′**
GAPDH	**F**- GTCAGTGGTGGACCTGACCT **R**- CACCACCCTGTTGCTGTAGC
OCT4A	**F**- AGCCCTCATTTCACCAGGCC **R**- TGGGACTCCTCCGGGTTTTG
SOX2	**F**- GGGGAAAGTAGTTTGCTGCCTCT **R**- TGCCGCCGCCGATGATTGTT
NR2F2	**F**- TCGACCAGCACCATCGCAAC **R**- CGCAACAGCAGGGAAATATATCC
KDR2	**F**- CCCAGATGACAACCAGACGGAC **R**- TGGGCTGTGCTACCGGTTTGC
MEF2C	**F**- CGAGATACCCACAACACACG **R**- TTCGTTCCGGTGATCCTC
PKP2	**F**- AGCAGGTGCAGCAGACCCTC **R**- CATAGGTTTTAGGAACAGGGGAACG
MESP1	**F**- CTCTGTTGGAGACCTGGATG **R**- CCTGCTTGCCTCAAAGTG
NKX2.5	**F**- ACCTCAACAGCTCCCTGACTCT **R**- ATAATCGCCGCCACAAACTCTCC
CTNT	**F**- GGCAGCGGAAGAGGATGCTGAA **R**- GAGGCACCAAGTTGGGCATGA ACG AC
MAP2	**F**- GCATGAGCTCTTGGCAGG **R**- CCAATTGAACCCATGTAAAGCC
GATA4	**F**- AAAAGGGAGGCTTCGGTCTC **R**- CTGGATGCTAGTTCCTGGGC
TBX5	**F**- GTCTACGCGGTACGTTTTGC **R**- GTCTTGGCCCCGGGAATAAA
INO80	**F**- TGGAGTCAGAAGACCCTCTACA **R**- TGTTCTGAATTGGGGTCCCG
MIR21	**F**- ATGTTGACTGTTGAATCTCATGGC **R**- TGTCAGACAGCCCATCGAC
ANRIL	**F**- TGCTCTATCCGCCAATCAGG **R**- GCGTGCAGCGGTTTAGTTT
BRG1	**F**- TACAAGGACAGCAGTGGACG **R**- GGTGAAGACCGACTGCAAGA
LTB	**F**- GAGGACTGGTAACGGAGACGG **R**- GAAACGCCTGTTCCTTCGTC
FRZB	**F**- CCCTGTAAGTCTGTGTGCGA **R**- TACAGCGTTCACTGCTTGC

### ChIP sequencing, ChIP-qPCR, Sequential ChIP-qPCR

Anti H3K27me3 antibody was used to immuno-precipitate the DNA and occupancy of H3K27me on promoters of NRF2 and OCT4 was studied while anti-EZH2 and anti-OCT4 antibodies were employed in ChIP and sequential ChIP respectively to study their binding with NR2F2.ChIP protocol was performed using standard protocol. 1–2 million hES cells (D0, D12 and D20) were cross linked with 1% formaldehyde for 10 mins at room temperature (RT). Formaldehyde was quenched with 1.25 M glycine and cells were rinsed with ice cold 1X PBS, collected and centrifuged at 2500 rpm for 5 mins. Supernatant was aspirated leaving behind the pellet of cross linked cells. Cells were lysed with cell lysis buffer containing protease inhibitor (PI) (50 mM HEPES/tris HCL, 140 mM NaCl, 1 mM EDTA, 1% triton X-100, 0.1% SDS, pH 7.5). Lysed cells were subjected to sonication for about 25 cycles using pulse ON for 30 sec and pulse OFF for 50 sec on (Bioruptor, Cosmo Bio Co. Ltd, Japan) to obtain fragments appropriate for performing qPCR (200–500 bp) or DNA sequencing (100–300 bp) analysis. Sonicated samples were then incubated with 10ug of anti H3K27me3 antibody (9733, Cell Signalling Technology, MA, USA) for ChIP-sequencing or anti EZH2 antibody (17–662, Milipore, MA, USA) for ChIP-qPCR in blocking solution (0.5% BSA in 1X PBS). The beads with antibody - DNA complexes were washed 3 times with each of lysis buffer, IP1 buffer (lysis buffer with 500 mM NaCl), IP2 buffer (10 mM trisHCl, 250 mM LiCl, 1 mM EDTA, 0.5% NP40, 0.5% sodium deoxycholate) and Tris EDTA (TE) buffer (10 mM Tris, 1 mM EDTA). For eluting the bound complexes, the bead – complex solution was incubated with 200ul elution buffer (TE buffer with 1% SDS) at 65 °C for 50 mins. The eluted product was treated with RNase A and proteinase K at 60 °C for 60 mins. Following this the immuno-precipitated DNA was isolated Phenol:Chloroform:Isoamyl alcohol method.

Sequential ChIP was performed using standard protocols. Briefly, the cross linked chromatin sample of undifferentiated and differentiated cells was immuno-precipitated using EZH2 antibody. Following this the complex was eluted using elution buffer with higher salt concentration and DTT (10 mM tris, 1 mM EDTA, 0.1% SDS, 500 mM NaCl, 30 mM DTT) in order to remove traces of EZH2 antibody. The resulting chromatin sample was then immuno-precipitated with anti OCT4 antibody (ab19857, Abcam, Cambridge, UK) and continued with standard ChIP protocol described above. The genomic DNA isolated after both ChIP and sequential ChIP experiments was quantified by qPCR (7900HT Fast Real-Time PCR System, ThermoFisher Scientific, MA, USA) calculated as % of total input DNA. The genes included in study are listed in Table 1 along with their primer sequences.

For ChIP sequencing step, the DNA isolated after the standard ChIP protocol immuno-precipitated with anti H3K27me3 antibody was quantified using Nanodrop (NanoDrop 8000, ThermoFisher Scientific, MA, USA) and then Illumina sequencing was done at sequencing facility of Genome Institute of Singapore (GIS). The data analysis was performed by Sandor Life Sciences Pvt Ltd, Hyderabad, India. The reads obtained were mapped with the Human hg19 and aligned using Bowtie (Galaxy tool). MACS (Galaxy tool) were employed for peak calling. The resulting peaks were visualized using Integrative Genome Viewer.

### Data availability

Microarray data raw files are available on GEO NCBI data base accession number GSE97268. ChIP sequencing raw files are available on SRA NCBI data base accession number SRP115340.

## Electronic supplementary material

Supplementary Information
