# Supplementary material for: Genetic and Epigenetic Profiling Reveals EZH2-mediated Down Regulation of OCT-4 Involves NR2F2 during Cardiac Differentiation of Human Embryonic Stem Cells
Source: Sci Rep. 2017 Oct 12;7:13051. doi: 10.1038/s41598-017-13442-9 (PMC5638931; doi:10.1038/s41598-017-13442-9)
Supplement: Supplementary file 1 — Supplementary Information [file 41598_2017_13442_MOESM1_ESM.pdf]

Genetic and Epigenetic Profiling Reveals EZH2-mediated Down Regulation of OCT-4 Involves NR2F2 during Cardiac Differentiation of Human Embryonic Stem Cells

Varsha Pursani[1], Prasad Pethe[1,2], Mohsin Bashir[3], Prabha Sampath[3], Vivek Tanavde[4,5],Deepa Bhartiya[1]*

Supplementary table 1: **Studies reporting the differentiation of ES cells into cardiac lineage and their transplantation**

DIFFERENTIATION STUDIES			
**Method**	**Differentiation technique and conditions**	**Efficiency**	**Reference**
Embryoid Body (EB) formation	hES cells placed in suspension cultures for 7-10 days to form EBs and replated on gelatine coated plates for 2 weeks	1% beating areas	Kehat et al[1]
Co-Culture with END2	hES cells co-cultured with inactivated mouse visceral Endoderm-like cell line, END-2; maintained for upto 6 weeks.	10% beating cardiomyocytes	Mummery et al[2]
Hanging Drop culture and 5-azacytidine treatment	hES cells aggregated by hanging drop technique treated with 5-azacytidine for 1-3 days	70% beating clusters	Yoon et al[3]
Matrix sandwich protocol	Sequential addition of Activin A and BMP4 to monolayered hES cells along with double layers of commercially available basement membrane Matrigel; cultured for 15 days	80-90% beating areas	Zhang et al[4]
Directed differentiation techniques	Monolayered hES cells treated with recombinant Activin A (24 hrs.) and BMP4 (4 days); cultured in RPMI-B27 for 2-3 weeks	More than 30% beating cardiomyocytes observed on 12th day of differentiation	Laflamme et al[5]
	hES cell derived EBs treated sequentially with combinations of growth factors like BMP4 (24 hrs.), Activin A, bFGF (4 days), DKK1 and VEGF (4 days) during 14 days culture	50-60% beating clusters around day 12	Yang et al[6]
	Monolayered hES cells exposed to growth factors like Activin A (24 hrs.), BMP4 (4 days) and DKK1 (4 days) in RPMI-B27 medium; cultured for 20 days	60-80% beating areas on day 20	Pawani et al[7]
Manipultion of WNT pathway	ES or iPS cells subjected to WNT/beta-catenin signalling stimulation with GSK3 inhibitor followed by its suppression using specific inhibitors along with growth factors ike BMP4	80-95% beating cardiomyocytes	Lian et al[8]; Cao et al[9]; Burridge et al[10]

TRANSPLANTATION STUDIES				
**Cell source**	**Condition – Host**	**Follow-up duration**	**Findings**	**Reference**
Cardiomyocytes differentiated from hES cells (hES cells – CMs)	Complete heart block – Pig	3 weeks	Transplanted cells survived and coupled with the host cardiomyocytes electrically and histologically. No reports of teratoma formation	Kehat et al[11]
hES cells - CMs	Acute myocardial infarction – Rat	60 days	Transplanted cells engrafted with host cells histologically, attenuated ventricular tissue remodelling and improved myocardial performance. No teratomas reported.	Caspi et al[12]
hES cells- CMs	Acute myocardial infarction – Mouse	12 weeks	Improvements in ejection fraction observed at 4 weeks but not at 12 weeks.	Van Laake et al[13]
hES cells - CMs	Chronic myocardial infarction –Rat	12 weeks	Transplanted cells survive and proliferate, develop increased sarcomere organization. However no significant improvements in ventricular structure or function.	Fernandes et al[14]
hES cells – cardiac progenitors (CPs)	Postmyocardial infraction - Rhesus monkey	≤12 weeks	Transplantation reconstitutes about 20% of scar tissue. No teratoma observed.	Blin et al[15]
mES cells - CPs	Myocardil infarction – mouse	≤4 weeks	Transplanted cells engrafted in the infarct zone and formed gap junctions. Attenuated ventricular dilation and improved systolic and diastolic functions. No teratoma formed.	Christoforou et al[16]
hES cells - CMs	Acute myocardial infarction – Guinea pig	4 weeks	Transplanted cells survived and integrated wih host tissue. Suppressed arrhythmias.	Shiba et al[17]
hES cells - CMs	Acute myocardial infarction – Rat	8 weeks	Transplanted cells engrafted histologically with the host cells. Funtional improvements noted till 4 weeks.	Moon et al[18]
HES cells – CPs, CMs	Myocardial infarction - Rat	4 weeks	Transplanted cells; both CPs and CMs differentiated from hES cells improved ventricular dilation and systolic function and generated comparable grafts with host tissue.	Fernandes et al[19]

References

1. Kehat, I. et al. Human embryonic stem cells can differentiate into myocytes with structural and functional properties of cardiomyocytes. J Clin Invest. **108**, 407-14 (2001).
2. Mummery, C. et al. Differentiation of human embryonic stem cells to cardiomyocytes: role of coculture with visceral endoderm-like cells. Circulation. **107**, 2733-40 (2003).
3. Yoon, B.S. et al. Enhanced differentiation of human embryonic stem cells into cardiomyocytes by combining hanging drop culture and 5-azacytidine treatment. Differentiation. **74**, 149-59 (2006).
4. Zhang, J. et al. Extracellular matrix promotes highly efficient cardiac differentiation of human pluripotent stem cells: the matrix sandwich method. Circ Res. **111**, 1125-36 (2012).
5. Laflamme, M.A. et al. Cardiomyocytes derived from human embryonic stem cells in pro-survival factors enhance function of infarcted rat hearts. Nat Biotechnol. **25**, 1015-24 (2007).
6. Yang, L. et al. Human cardiovascular progenitor cells develop from a KDR+ embryonic-stem-cell-derived population. Nature. **453**, 524-8 (2008).
7. Pawani, H., Nagvenkar, P., Pethe, P. & Bhartiya D. Differentiation of human ES cell line KIND-2 to yield tripotent cardiovascular progenitors. In Vitro Cell Dev Biol Anim. **49**, 82-93 (2013).
8. Lian, X. et al. Robust cardiomyocyte differentiation from human pluripotent stem cells via temporal modulation of canonical Wnt signaling. Proc Natl Acad Sci U S A. **109**, E1848-57 (2012).
9. Cao, N. et al. Ascorbic acid enhances the cardiac differentiation of induced pluripotent stem cells through promoting the proliferation of cardiac progenitor cells. Cell Res. **22**, 219-36 (2012).
10. Burridge, P.W. et al. Chemically defined generation of human cardiomyocytes. Nat Methods. **11**, 855-60 (2014).
11. Kehat, I. et al. Electromechanical integration of cardiomyocytes derived from human embryonic stem cells. Nat Biotechnol. **22**, 1282-9 (2004).
12. Caspi, O. et al. Transplantation of human embryonic stem cell-derived cardiomyocytes improves myocardial performance in infarcted rat hearts. J Am Coll Cardiol. **50,** 1884-93 (2007).
13. van Laake, L.W. et al. Human embryonic stem cell-derived cardiomyocytes survive and mature in the mouse heart and transiently improve function after myocardial infarction. Stem Cell Res. **1**, 9-24 (2007).
14. Fernandes, S. et al. Human embryonic stem cell-derived cardiomyocytes engraft but do not alter cardiac remodeling after chronic infarction in rats. J. Mol. Cell. Cardiol. **49**, 941–9 (2010).
15. Blin, G. et al. A purified population of multipotent cardiovascular progenitors derived from primate pluripotent stem cells engrafts in post myocardial infarcted nonhuman primates. J Clin Invest. **120**, 1125–39 (2010).
16. Christoforou, N. et al. Implantation of mouse embryonic stem cell-derived cardiac progenitor cells preserves function of infarcted murine hearts. PLoS One. **5**, e11536 (2010).
17. Shiba, Y. et al. Human ES-cell-derived cardiomyocytes electrically couple and suppress arrhythmias in injured hearts. Nature. **489,** 322-5 (2012).
18. Moon, S.H. et al. The use of aggregates of purified cardiomyocytes derived from human ESCs for functional engraftment after myocardial infarction. Biomaterials. **34**, 4013-26 (2013).
19. Fernandes, S. et al. Comparison of human embryonic stem cell-derived cardiomyocytes, cardiovascular progenitors, and bone marrow mononuclear cells for cardiac repair. Stem Cell Reports. **5**, 753–62 (2015).

Maintenance of human embryonic stem cells

In-house derived human ES cells were grown on Geltrex (Invitrogen, Carlsbad, CA, USA) coated plates in Stempro hESC SFM medium (Invitrogen, Carlsbad, CA, USA) supplemented with basic fibroblast growth factor (bFGF) (R & D Systems, MN, USA) at a concentration of 8ng/ml in a humidified atmosphere of 37^0C with 5% CO_2. Cells were subcultured every 5-7 days by detaching them from the geltrex-coated plates mechanically using cell lifter (Sigma Aldrich, MO, USA). Prior to directed differentiation into cardiac lineage, undifferentiated hES cells were analyzed at both transcript and protein levels for the expression of pluripotent markers by qPCR, immunofluorescence and flow cytometry (Spplementary figures 3-5).

Directed Differentiation

Undifferentiated human ES cells were subjected to a series of defined growth factors to direct the differentiation towards cardiac lineage by the protocol as reported previously by our group (Pawani et al. 2013) (Supplementary figure 1). Briefly, confluent monolayer cultures of human ES cells were treated with 100ng/ml Activin A (R &D Systems) and 5ng/ml of bFGF (R&D Systems) in serum free medium RPMI 1640 supplemented with 5% B-27 and 1% glutamax for 24 hrs. Next day fresh RPMI medium was added containing 15ng/ml BMP4 (R & D Systems) and 5ng/ml bFGF (R & D Systems) for another 4 days. This was then followed by 150ng/ml DKK1 (R & D Systems) containing fresh RPMI medium for 4 days. After this on day 9 of differentiation, cultures were maintained in basal medium of RPMI 1640 plus 5% B-27 and 1% glutamax for next 2 weeks till day 20 when the large detectable beating areas were observed. Basal media was changed every alternate day during the 2 week culture. Upon gene and marker analysis, the stages selected for the study included pluripotent hES cells at D0 (OCT4, NANOG, SOX2), CPs at D12 (MESP1, NKX2.5, MEF2C) and CMs at D0 (CTNT).

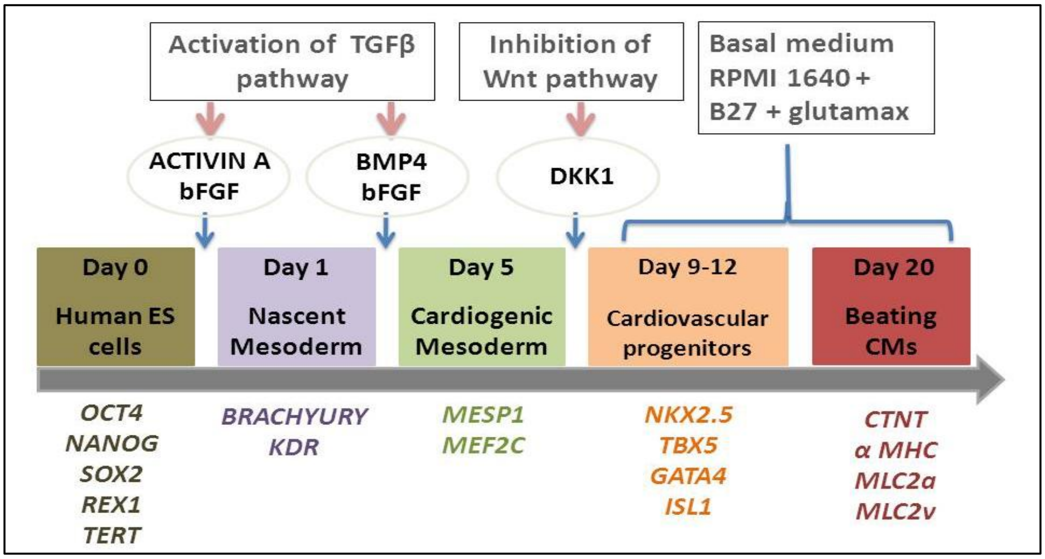

Supplementary figure 1: Differentiation of hES cells into Cardiac lineage. Schematic representation of the directed differentiation protocol followed for differentiation of hES cells into cardiac lineage.

Cardiac directed differentiation of human ES cells

KIND1 cells differentiated into cardiac lineage using reported protocol (Supplementary Fig 1), showed distinct morphological changes (Supplementary figure 2).

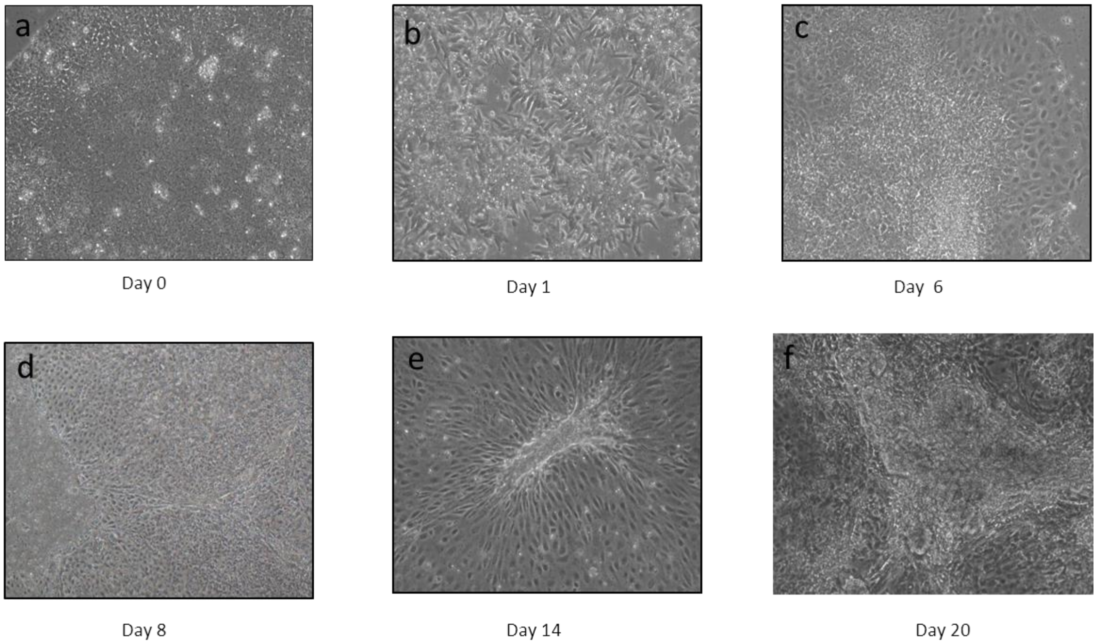

__Supplementary figure 2:__ **Undifferentiated and differentiated hES cells.** Bright field images of hES cells during their cardiac directed differentiation (a) pluripotent KIND1 cells at D0, differentiation at days 1 (b), 6 (c), 8 (d), 14 (e) and 20 (f). D20 cells represent beating cardiomyocytes. Magnification 10X

Characterization studies

Prior to directed differentiation into cardiac lineage, undifferentiated hES cells were analyzed at both transcript and protein levels for the expression of pluripotent markers by PCR and immunofluorescence respectively.

__Quantitative Real-Time PCR (qRT-PCR)__

Total RNA was isolated from undifferentiated hES cells using TRIzol reagent (Invitrogen, Carlsbad, CA, USA) as per manufacturer’s instructions. The integrity and quality of RNA was confirmed spectrophotometrically using Ultrospec 3100 pro (GE Healthcare, PA, USA). First strand or cDNA synthesis was performed from 1ug of total RNA using iScript cDNA synthesis kit (Bio-Rad Laboratories, CA, USA) and random hexamer primers as per manufacturer’s instructions using GSTORM thermal cycler (Gene Technologies, Braintree, UK). Expression of pluripotency transcripts and cardiac lineage transcripts was determined by Quantitative Real-Time PCR (qRT-PCR). qRT - PCR was performed using iQ SYBR Green Supermix (Bio Rad) on a CFX96 Real Time Machine (Bio Rad). The amplification programme consisted of the initial denaturation step at 95°C for 5 min, followed by 40 cycles of denaturation at 95°C for 10 sec, annealing at 62°C for 20 sec and elongation at 72°C for30 sec. Fluorescence emitted at the end of each extension step was measured. The

specificity and homogeneity of the amplified product was confirmed by performing melt curve analysis at the end of each amplification cycle and also by electrophoresing the products on 2% agarose gel (Bangalore Genei). Human fetal and adult whole heart RNA were used to compare the maturity of the differentiated cells. The fold change was determined for each sample of different time points using the $2^{-\Delta\Delta Ct}$ method and was expressed relative to that of GAPDH which was used as an internal control. The expression level of each gene transcript is normalized to a value of 1.0 for undifferentiated cells. The error bars represent ± standard error of the mean (SEM). All the above results are an average of at least 3 biological replicates (Supplementary figure 3). Primers used are mentioned in Table 1.

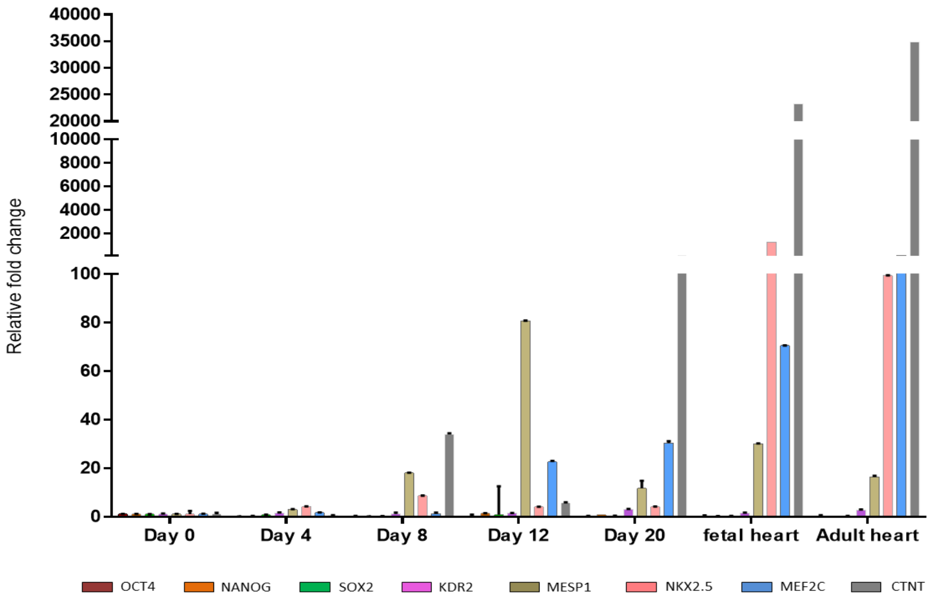

<u>Supplementary figure 3</u>: Characterization of cardiac differentiation of hES cells by qPCR Expression of transcripts representing pluripotency (OCT4, NANOG, SOX2), nascent mesoderm (KDR2), cardiac mesoderm (MESP1), cardiac progenitors (NKX2.5, MEF2C) and beating cardiomyocytes (CTNT) at days 0, 4, 8, 12, 20 during 20 days cardiac differentiation. Differentiation was also compared with the corresponding transcript levels in fetal and adult cardiac RNA obtained commercially. Error bars represent ± SEM.

The differentiation into cardiac lineage was analyzed for appearance of cardiac progenitors and cardiomyocytes by qPCR (Supplementary figure 3). Substantial decrease of key pluripotent genes as compared to the appearance of KDR2 gene revealed the formation of mesodermal lineage that was directed towards cardiac fate was confirmed by the appearance of cardiac mesodermal genes like MESP1 and TBX5. Day 12 of directed differentiation marked the formation of cardiovascular progenitors characterized by the genes MEF2C, NKX2.5, VECAD and SMA that led to the formation of beating cardiomyocytes (movie) expressing CTNT and alpha MHC by day 20. Cardiomyocytes derived from hES cells indicated a consistent differentiation when compared with fetal and adult whole heart tissue RNA at the essential mesodermal transcripts level (Supplementary figure 3).

Immunofluorescence

Undifferentiated human ES cells were subjected to immunostaining using specific primary antibodies to pluripotent markers. Briefly undifferentiated human ES cells were fixed with 4% paraformaldehyde (PFA) for 15 mins at room temperature followed by washing with 1X PBS plus 0.02% Tween 20 (Sigma Aldrich). Permeabilization for nuclear antigens was done with 0.3% triton X-100 (Sigma-Aldrich). Non-specific sites were blocked by blocking buffer containing 1X PBS plus 5% BSA (Sigma Aldrich) and 1% normal goat serum (Bangalore Genei, Bangalore, India) for 60mins at room temperature. The cells were then incubated with primary antibodies mouse anti OCT4 (1:200, Millipore, CA, USA), rabbit anti NANOG (1:200, Epitomics, CA, USA) and mouse anti SSEA4 (1:100, Millipore, CA, USA) diluted in blocking buffer for overnight at 4^{0}C. This was followed by washing and incubation with conjugated secondary antibodies goat/rabbit Alexafluor 488 (1:1000, Molecular Probes, Invitrogen) diluted in blocking buffer for 2 hrs at room temperature. Counterstaining was done using 300nM 4,6-diamidino-2-phenylindole (DAPI) (Molecular Probes, Invitrogen). Images were captured under Fluorescent microscope (90i Nikon, Japan) or laser scanning confocal microscope (Carl Zeiss, Oberkochen, Germany) (Supplementary figure 4).

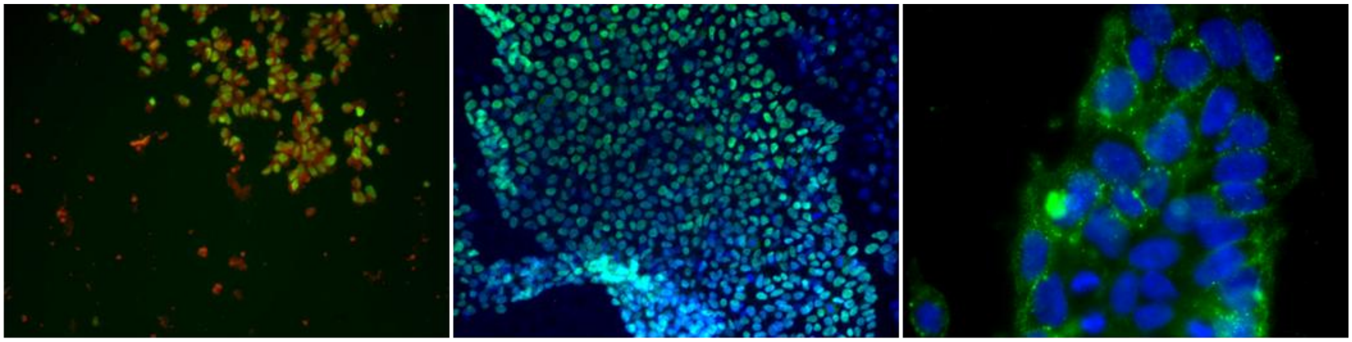

(A) (B) (C)

Supplementary figure 4: Characterization of cardiac differentiation of hES cells by Immunofluorescence. Immunofluorescence for OCT4 (red) (magnification 10X) (A) NANOG (green) (magnification 10X) (B) and SSEA4 (green) (magnification 20X) (C) in undifferentiated hES cells. Counter staining was done using PI for (A) and PI for (B) and (C). Magnifications 20X.

Flow Cytometry characterization for undifferentiated and differentiated cells

Differentiated human ES cells were analyzed at undifferentiated and at both the cardiovascular progenitor stage and cardiomyocyte stage by flow cytometry (Supplementary figure 5). Cell cultures harvested in PBS were dissociated into single cells. Antibody used to stain the pluripotent hES cells was anti –OCT4 and for the cardiac progenitor stage cells was rabbit anti-human NKX2.5. For nuclear marker staining, cells were fixed in 2% paraformaldehyde for 15 mins followed by their permeabilization using 0.3% Triton-X 100. Following multiple washes, cell samples were run on BD FACS Aria (BD Biosciences, San Jose, CA, USA) and were analyzed using BD FACS Diva Software.

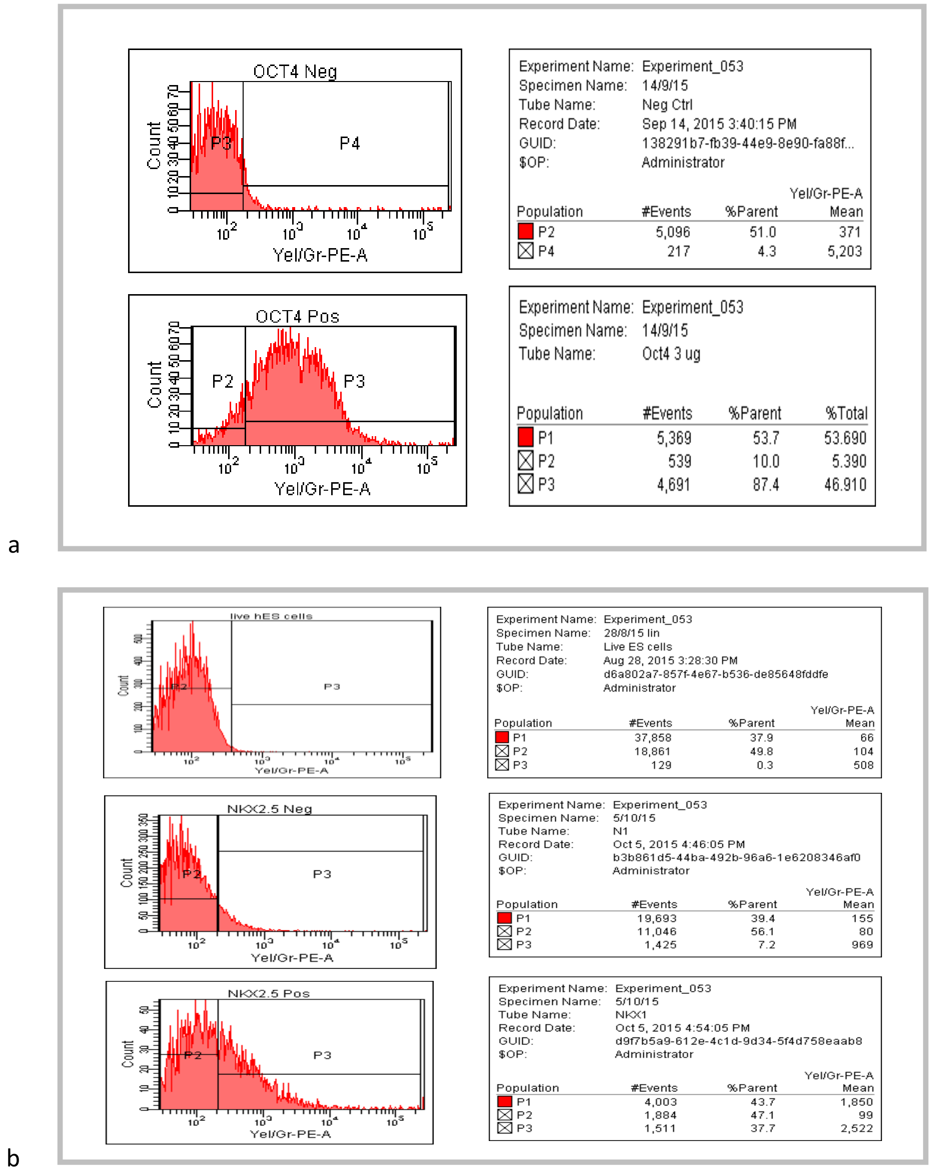

<u>**Supplementary figure 5**</u>: **Flow cytometric characterization:** Undifferentiated hES cells and differentiated cells were characterized by flow cytometry using OCT4 (A) for D0 cells and NKX2.5 (B) for D12 cells representing undifferentiated hES cells and cardiac progenitors respectively.

Microarray

Gene Ontology

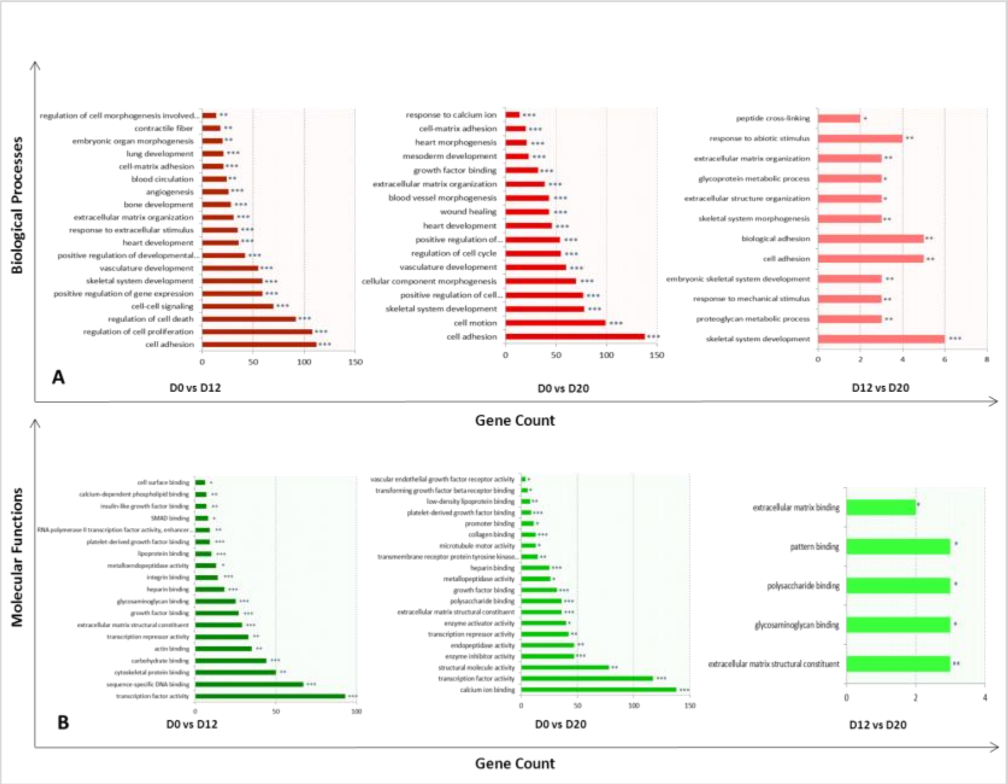

Supplementary figure 6: Gene Ontology. Significant annotations (p < 0.05) during the differentiation of cells analysed according to the biological processes (A) and molecular functions (B). Pvalue <0.05 (*), 0.01 (**), 0.001 (***).

Signaling pathways

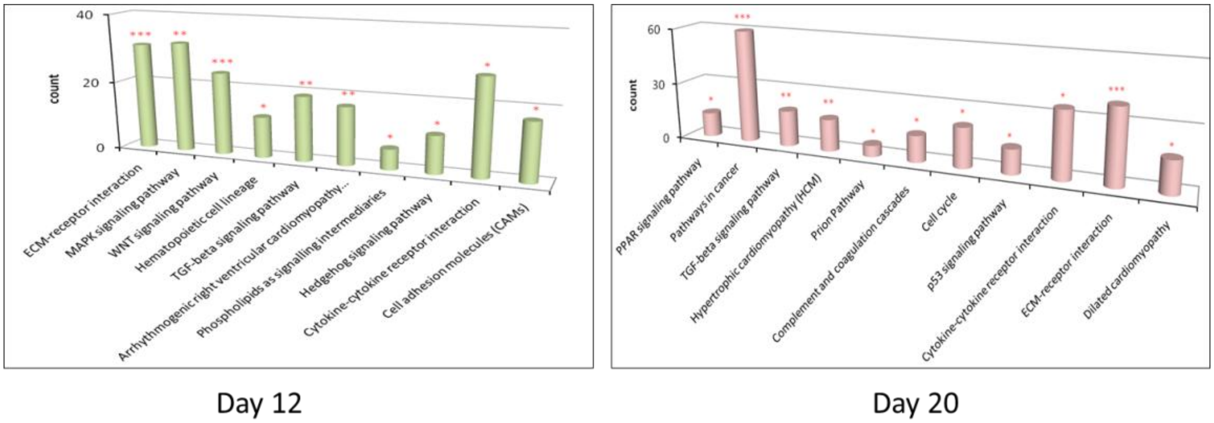

Supplementary figure 7: Pathway classification of enriched transcripts during differentiation. Up regulated genes searched against the KEGG and Biocarta pathway to identify the members of the signalling pathways involved during the cardiac differentiation. Pvalue <0.05 (*), 0.01 (**), 0.001 (***).

Microarray Data Validation

Immunofluorescence

Microarray data was also validated at protein level by studying the immuno-expression of NKX2.5 and CTNT by confocal microscopy. Immuno-staining was performed as described above using the primary antibodies specific for markers of hES derived cardiac progenitors and cardiomyocytes; rabbit anti-human NKX2.5 (R&D Systems, MAB2444) (1:200) and mouse anti-human CTNT (Abcam, ab10214) ((1:100). (Supplementary figure 8).

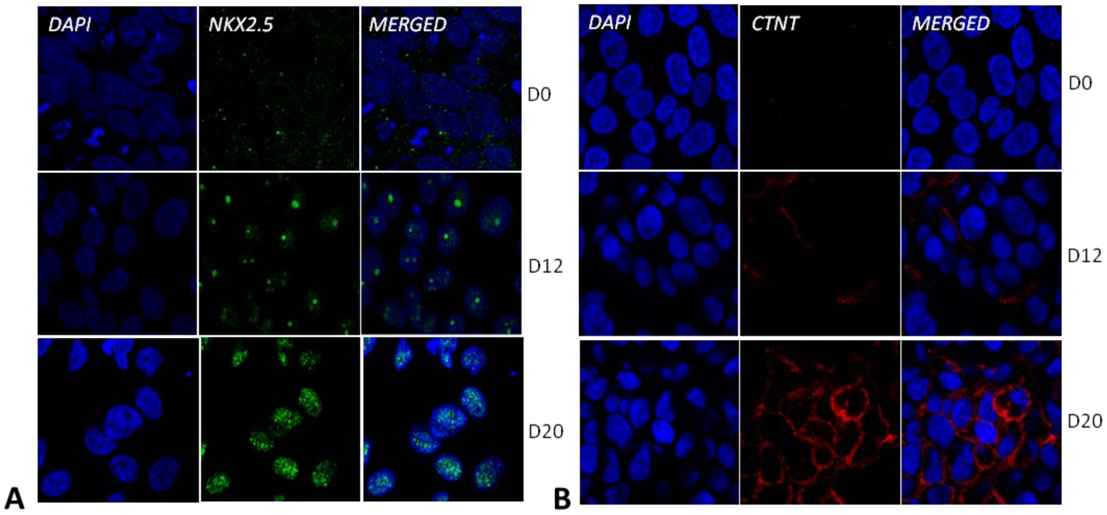

Supplementary figure 8: Validation of Microarray data by Immunofluorescence. Immunofluorescence for NKX2.5 (A) and CTNT (B) at days 0, 12 and 20. (A) Clearly shows the nuclear localization of NKX2.5 while (B) shows the surface localization of the surface marker CTNT. Also both the markers are absent at day 0. Counter staining was done using DAPI. Magnifications 20X.

Supplementary table 2: Genes up regulated in D0 as compared to D12

Refseq_NM	GeneSymbol	D12/D0.fc	DEFINITION
NM_002522.2	NPTX1	-32.593	Homo sapiens neuronal pentraxin I (NPTX1), mRNA.
NM_007015.2	LECT1	-16.402	Homo sapiens leukocyte cell derived chemotaxin 1 (LECT1), transcript variant 1, mRNA.
NM_007015.2	LECT1	-15.327	Homo sapiens leukocyte cell derived chemotaxin 1 (LECT1), transcript variant 1, mRNA.
NM_002164.3	INDO	-13.881	Homo sapiens indoleamine-pyrrole 2,3 dioxygenase (INDO), mRNA.
NM_002164.4	IDO1	-12.805	Homo sapiens indoleamine 2,3-dioxygenase 1 (IDO1), mRNA.
NM_022034.4	CUZD1	-10.320	Homo sapiens CUB and zona pellucida-like domains 1 (CUZD1), mRNA.
NM_014279.4	OLFM1	-9.391	Homo sapiens olfactomedin 1 (OLFM1), transcript variant 1, mRNA.
NM_003106.2	SOX2	-9.109	Homo sapiens SRY (sex determining region Y)-box 2 (SOX2), mRNA.
AL833138		-8.576	Homo sapiens mRNA; cDNA DKFZp313A1040 (from clone DKFZp313A1040)
NM_002523.1	NPTX2	-8.278	Homo sapiens neuronal pentraxin II (NPTX2), mRNA.
NM_015715.3	PLA2G3	-8.222	Homo sapiens phospholipase A2, group III (PLA2G3), mRNA.
NM_138328.2	RHBDL3	-8.039	Homo sapiens rhomboid, veinlet-like 3 (Drosophila) (RHBDL3), mRNA.
NM_003106.2	SOX2	-7.544	Homo sapiens SRY (sex determining region Y)-box 2 (SOX2), mRNA.
NM_005296.1	LPAR4	-6.981	Homo sapiens lysophosphatidic acid receptor 4 (LPAR4), mRNA.
NM_005756.2	GPR64	-6.821	Homo sapiens G protein-coupled receptor 64 (GPR64), transcript variant 2, mRNA.
AL049310		-6.745	Homo sapiens mRNA; cDNA DKFZp564B206 (from clone DKFZp564B206)
NM_152779.2	GLIPR1L1	-6.702	Homo sapiens GLI pathogenesis-related 1 like 1 (GLIPR1L1), mRNA.
NM_198139.1	SEMG1	-6.574	Homo sapiens semenogelin I (SEMG1), transcript variant 2, mRNA.
NM_024046.3	CAMKV	-6.442	Homo sapiens CaM kinase-like vesicle-associated (CAMKV), mRNA.
NM_001217.3	CA11	-6.437	Homo sapiens carbonic anhydrase XI (CA11), mRNA.
NM_032884.2	C1orf94	-6.430	Homo sapiens chromosome 1 open reading frame 94 (C1orf94), mRNA.
NM_022144.1	TNMD	-6.338	Homo sapiens tenomodulin (TNMD), mRNA.
NM_001042403.1	USP44	-6.283	Homo sapiens ubiquitin specific peptidase 44 (USP44), transcript variant 2, mRNA.
NM_032803.4	SLC7A3	-6.248	Homo sapiens solute carrier family 7 (cationic amino acid transporter, y+ system), member 3 (SLC7A3), transcript variant 2, mRNA.
AF038185		-6.112	Homo sapiens clone 23700 mRNA sequence
NR_003491.1	MIAT	-5.940	Homo sapiens myocardial infarction associated transcript (non-protein coding) (MIAT), non-coding RNA.
NM_001042403.1	USP44	-5.897	Homo sapiens ubiquitin specific peptidase 44 (USP44), transcript variant 2, mRNA.
NM_004538.3	NAP1L3	-5.787	Homo sapiens nucleosome assembly protein 1-like 3 (NAP1L3), mRNA.
NM_007129.2	ZIC2	-5.765	Homo sapiens Zic family member 2 (odd-paired homolog, Drosophila) (ZIC2), mRNA.
NM_172337.1	OTX2	-5.714	Homo sapiens orthodenticle homeobox 2 (OTX2), transcript variant 2, mRNA.
NM_003459.4	SLC30A3	-5.670	Homo sapiens solute carrier family 30 (zinc transporter), member 3 (SLC30A3), mRNA.
NM_006334.3	OLFM1	-5.654	Homo sapiens olfactomedin 1 (OLFM1), transcript variant 2, mRNA.
XM_001723077.1	LOC100132934	-5.649	PREDICTED: Homo sapiens similar to hCG1644233 (LOC100132934), mRNA.
NM_015541.2	LRIG1	-5.645	Homo sapiens leucine-rich repeats and immunoglobulin-like domains 1 (LRIG1), mRNA.
NM_002851.2	PTPRZ1	-5.639	Homo sapiens protein tyrosine phosphatase, receptor-type, Z polypeptide 1

			(PTPRZ1), mRNA.
NM_032446.1	MEGF10	-5.571	Homo sapiens multiple EGF-like-domains 10 (MEGF10), mRNA.
NM_001079859.1	GPR64	-5.556	Homo sapiens G protein-coupled receptor 64 (GPR64), transcript variant 2, mRNA.
NM_004734.2	DCLK1	-5.544	Homo sapiens doublecortin-like kinase 1 (DCLK1), mRNA.
NM_000517.3	HBA2	-5.499	Homo sapiens hemoglobin, alpha 2 (HBA2), mRNA.
XR_039453.1	LOC646316	-5.497	PREDICTED: Homo sapiens misc_RNA (LOC646316), miscRNA.
NM_014474.2	SMPDL3B	-5.465	Homo sapiens sphingomyelin phosphodiesterase, acid-like 3B (SMPDL3B), transcript variant 1, mRNA.
NM_002993.2	CXCL6	-5.448	Homo sapiens chemokine (C-X-C motif) ligand 6 (granulocyte chemotactic protein 2) (CXCL6), mRNA.
NM_181876.2	PPP2R2C	-5.440	Homo sapiens protein phosphatase 2 (formerly 2A), regulatory subunit B, gamma isoform (PPP2R2C), transcript variant 2, mRNA.
NM_003706.1	PLA2G4C	-5.406	Homo sapiens phospholipase A2, group IVC (cytosolic, calcium-independent) (PLA2G4C), mRNA.
NM_000517.3	HBA2	-5.339	Homo sapiens hemoglobin, alpha 2 (HBA2), mRNA.
NM_004744.3	LRAT	-5.336	Homo sapiens lecithin retinol acyltransferase (phosphatidylcholine--retinol O-acyltransferase) (LRAT), mRNA.
NM_005634.2	SOX3	-5.333	Homo sapiens SRY (sex determining region Y)-box 3 (SOX3), mRNA.
NM_002006.3	FGF2	-5.286	Homo sapiens fibroblast growth factor 2 (basic) (FGF2), mRNA.
NM_005314.2	GRPR	-5.269	Homo sapiens gastrin-releasing peptide receptor (GRPR), mRNA.
NM_006467.2	POLR3G	-5.258	Homo sapiens polymerase (RNA) III (DNA directed) polypeptide G (32kD) (POLR3G), mRNA.
NM_001001995.1	GPM6B	-5.256	Homo sapiens glycoprotein M6B (GPM6B), transcript variant 1, mRNA.
NM_015541.2	LRIG1	-5.253	Homo sapiens leucine-rich repeats and immunoglobulin-like domains 1 (LRIG1), mRNA.
XM_498560.3	LOC440132	-5.188	PREDICTED: Homo sapiens hypothetical LOC440132 (LOC440132), mRNA.
NM_001851.3	COL9A1	-5.158	Homo sapiens collagen, type IX, alpha 1 (COL9A1), transcript variant 1, mRNA.
NM_080390.3	TCEAL2	-5.094	Homo sapiens transcription elongation factor A (SII)-like 2 (TCEAL2), mRNA.
NM_002701.4	POU5F1	-5.033	Homo sapiens POU class 5 homeobox 1 (POU5F1), transcript variant 1, mRNA.
NM_000892.3	KLKB1	-5.025	Homo sapiens kallikrein B, plasma (Fletcher factor) 1 (KLKB1), mRNA.
NM_015184.3	PLCL2	-5.023	Homo sapiens phospholipase C-like 2 (PLCL2), mRNA.
NM_004522.1	KIF5C	-4.980	Homo sapiens kinesin family member 5C (KIF5C), mRNA.
NM_032805.1	ZSCAN10	-4.950	Homo sapiens zinc finger and SCAN domain containing 10 (ZSCAN10), mRNA.
NM_172337.1	OTX2	-4.855	Homo sapiens orthodenticle homeobox 2 (OTX2), transcript variant 2, mRNA.
NM_006875.2	PIM2	-4.841	Homo sapiens pim-2 oncogene (PIM2), mRNA.
NM_018228.1	C14orf180	-4.807	Homo sapiens chromosome 14 open reading frame 115 (C14orf115), mRNA.
NM_003865.1	HESX1	-4.772	Homo sapiens HESX homeobox 1 (HESX1), mRNA.
NR_002304.1	POU5F1P1	-4.771	Homo sapiens POU class 5 homeobox 1 pseudogene 1 (POU5F1P1), non-coding RNA.
NM_012183.1	FOXD3	-4.742	Homo sapiens forkhead box D3 (FOXD3), mRNA.
NM_000478.3	ALPL	-4.709	Homo sapiens alkaline phosphatase, liver/bone/kidney (ALPL), transcript variant 1, mRNA.
NM_001048164.1	SLC7A3	-4.696	Homo sapiens solute carrier family 7 (cationic amino acid transporter, y+ system), member 3 (SLC7A3), transcript variant 2, mRNA.
NM_172109.1	KCNQ2	-4.692	Homo sapiens potassium voltage-gated channel, KQT-like subfamily, member 2 (KCNQ2), transcript variant 5, mRNA.
NR_024281.1	LOC157627	-4.654	Homo sapiens hypothetical LOC157627 (LOC157627), non-coding RNA.

NM_005407.1	SALL2	-4.652	Homo sapiens sal-like 2 (Drosophila) (SALL2), mRNA.
NM_001010940.1	C9orf135	-4.604	Homo sapiens chromosome 9 open reading frame 135 (C9orf135), mRNA.
NM_005314.2	GRPR	-4.589	Homo sapiens gastrin-releasing peptide receptor (GRPR), mRNA.
NM_006159.1	NELL2	-4.563	Homo sapiens NEL-like 2 (chicken) (NELL2), mRNA.
NM_024504.2	PRDM14	-4.552	Homo sapiens PR domain containing 14 (PRDM14), mRNA.
NM_001014809.1	CRMP1	-4.527	Homo sapiens collapsin response mediator protein 1 (CRMP1), transcript variant 1, mRNA.
NR_002304.1	POU5F1P1	-4.466	Homo sapiens POU class 5 homeobox 1 pseudogene 1 (POU5F1P1), non-coding RNA.
NM_003739.4	AKR1C3	-4.434	Homo sapiens aldo-keto reductase family 1, member C3 (3-alpha hydroxysteroid dehydrogenase, type II) (AKR1C3), mRNA.
XR_016333.1	LOC642559	-4.408	PREDICTED: Homo sapiens similar to POU domain, class 5, transcription factor 1 (Octamer-binding transcription factor 3) (Oct-3) (Oct-4) (LOC642559), mRNA.
NM_001009568.1	SMPDL3B	-4.401	Homo sapiens sphingomyelin phosphodiesterase, acid-like 3B (SMPDL3B), transcript variant 2, mRNA.
NM_003413.2	ZIC3	-4.399	Homo sapiens Zic family member 3 heterotaxy 1 (odd-paired homolog, Drosophila) (ZIC3), mRNA.
NM_001001995.1	GPM6B	-4.383	Homo sapiens glycoprotein M6B (GPM6B), transcript variant 1, mRNA.
NM_004734.2	DCLK1	-4.363	Homo sapiens doublecortin-like kinase 1 (DCLK1), mRNA.
XM_939432.1	MGC61598	-4.350	PREDICTED: Homo sapiens similar to ankyrin-repeat protein Nrarp (MGC61598), mRNA.
NM_004615.2	TSPAN7	-4.327	Homo sapiens tetraspanin 7 (TSPAN7), mRNA.
NM_001080430.1	TOX3	-4.303	Homo sapiens TOX high mobility group box family member 3 (TOX3), mRNA.
NM_007084.2	SOX21	-4.301	Homo sapiens SRY (sex determining region Y)-box 21 (SOX21), mRNA.
NM_013364.4	PNMA3	-4.294	Homo sapiens paraneoplastic antigen MA3 (PNMA3), mRNA.
NM_003541.2	HIST1H4K	-4.248	Homo sapiens histone cluster 1, H4k (HIST1H4K), mRNA.
NM_001018109.1	PIR	-4.241	Homo sapiens pirin (iron-binding nuclear protein) (PIR), transcript variant 2, mRNA.
NM_014474.2	SMPDL3B	-4.157	Homo sapiens sphingomyelin phosphodiesterase, acid-like 3B (SMPDL13B), transcript variant 1, mRNA.
XM_001134320.1	FLJ25404	-4.151	PREDICTED: Homo sapiens hypothetical protein FLJ25404, transcript variant 2 (FLJ25404), mRNA.
NM_152737.2	RNF182	-4.118	Homo sapiens ring finger protein 182 (RNF182), mRNA.
AL136588		-4.104	Homo sapiens mRNA; cDNA DKFZp761D112 (from clone DKFZp761D112)
NM_203400.1	RPRML	-4.096	Homo sapiens reprimo-like (RPRML), mRNA.
NM_014692.1	SEC14L5	-4.095	Homo sapiens SEC14-like 5 (S. cerevisiae) (SEC14L5), mRNA.
AK123661		-4.050	Homo sapiens cDNA FLJ41667 fis, clone FEBRA2028366
NM_012419.4	RGS17	-4.037	Homo sapiens regulator of G-protein signaling 17 (RGS17), mRNA.
NM_013243.2	SCG3	-4.019	Homo sapiens secretogranin III (SCG3), mRNA.
NM_001449.3	FHL1	-4.004	Homo sapiens four and a half LIM domains 1 (FHL1), mRNA.
NM_003218.2	TERF1	-4.003	Homo sapiens telomeric repeat binding factor (NIMA-interacting) 1 (TERF1), transcript variant 2, mRNA.
NM_005763.2	AASS	-4.003	Homo sapiens aminoadipate-semialdehyde synthase (AASS), nuclear gene encoding mitochondrial protein, mRNA.
NM_017522.3	LRP8	-4.000	Homo sapiens low density lipoprotein receptor-related protein 8, apolipoprotein e receptor (LRP8), transcript variant 4, mRNA.
NM_181676.1	PPP2R2B	-3.993	Homo sapiens protein phosphatase 2 (formerly 2A), regulatory subunit B, beta isoform (PPP2R2B), transcript variant 4, mRNA.
NM_000216.2	KAL1	-3.992	Homo sapiens Kallmann syndrome 1 sequence (KAL1), mRNA.

NM_018476.3	BEX1	-3.989	Homo sapiens brain expressed, X-linked 1 (BEX1), mRNA.
AK091593		-3.982	Homo sapiens cDNA FLJ34274 fis, clone FEBRA2003327
NM_001823.3	CKB	-3.986	Homo sapiens creatine kinase, brain (CKB), mRNA.
NM_020927.1	VAT1L	-3.950	Homo sapiens vesicle amine transport protein 1 homolog (T. californica)-like (VAT1L), mRNA.
NM_033036.2	GAL3ST3	-3.942	Homo sapiens galactose-3-O-sulfotransferase 3 (GAL3ST3), mRNA.
NM_016307.3	PRRX2	-3.916	Homo sapiens paired related homeobox 2 (PRRX2), mRNA.
NM_138786.1	TM4SF18	-3.902	Homo sapiens transmembrane 4 L six family member 18 (TM4SF18), mRNA.
NM_012281.2	KCND2	-3.903	Homo sapiens potassium voltage-gated channel, Shal-related subfamily, member 2 (KCND2), mRNA.
NM_020659.2	TTYH1	-3.860	Homo sapiens tweety homolog 1 (Drosophila) (TTYH1), transcript variant 1, mRNA.
NM_021127.1	PMAIP1	-3.839	Homo sapiens phorbol-12-myristate-13-acetate-induced protein 1 (PMAIP1), mRNA.
NM_173462.3	PAPLN	-3.824	Homo sapiens papilin, proteoglycan-like sulfated glycoprotein (PAPLN), mRNA.
NM_001038.4	SCNN1A	-3.816	Homo sapiens sodium channel, nonvoltage-gated 1 alpha (SCNN1A), mRNA.
NM_174936.2	PCSK9	-3.801	Homo sapiens proprotein convertase subtilisin/kexin type 9 (PCSK9), mRNA.
NM_000869.2	HTR3A	-3.787	Homo sapiens 5-hydroxytryptamine (serotonin) receptor 3A (HTR3A), transcript variant 2, mRNA.
NM_020997.2	LEFTY1	-3.784	Homo sapiens left-right determination factor 1 (LEFTY1), mRNA.
NM_003507.1	FZD7	-3.775	Homo sapiens frizzled homolog 7 (Drosophila) (FZD7), mRNA.
NM_133448.1	TMEM132D	-3.770	Homo sapiens transmembrane protein 132D (TMEM132D), mRNA.
NM_017899.2	TESC	-3.764	Homo sapiens tescalcin (TESC), mRNA.
NM_000814.4	GABRB3	-3.754	Homo sapiens gamma-aminobutyric acid (GABA) A receptor, beta 3 (GABRB3), transcript variant 1, mRNA.
NM_020211.1	RGMA	-3.753	Homo sapiens RGM domain family, member A (RGMA), mRNA.
NM_181505.1	PPP1R1B	-3.746	Homo sapiens protein phosphatase 1, regulatory (inhibitor) subunit 1B (dopamine and cAMP regulated phosphoprotein, DARPP-32) (PPP1R1B), transcript variant 2, mRNA.
NM_031845.2	MAP2	-3.735	Homo sapiens microtubule-associated protein 2 (MAP2), transcript variant 1, mRNA.
NM_016518.2	PIPOX	-3.726	Homo sapiens pipecolic acid oxidase (PIPOX), mRNA.
NM_003524.2	HIST1H2BH	-3.690	Homo sapiens histone cluster 1, H2bh (HIST1H2BH), mRNA.
NM_006892.3	DNMT3B	-3.688	Homo sapiens DNA (cytosine-5-)-methyltransferase 3 beta (DNMT3B), transcript variant 1, mRNA.
NM_052943.2	FAM46B	-3.686	Homo sapiens family with sequence similarity 46, member B (FAM46B), mRNA.
XM_001717875.1	LOC732445	-3.670	PREDICTED: Homo sapiens similar to alpha 7 neuronal nicotinic acetylcholine receptor (LOC732445), mRNA.
NM_138786.2	TM4SF18	-3.658	Homo sapiens transmembrane 4 L six family member 18 (TM4SF18), mRNA.
NM_032512.2	PDZD4	-3.654	Homo sapiens PDZ domain containing 4 (PDZD4), mRNA.
NM_005763.2	AASS	-3.648	Homo sapiens aminoadipate-semialdehyde synthase (AASS), nuclear gene encoding mitochondrial protein, mRNA.
NM_001080466.1	BTBD17	-3.646	Homo sapiens BTB (POZ) domain containing 17 (BTBD17), mRNA.
XR_015766.1	LOC730051	-3.631	PREDICTED: Homo sapiens similar to Zinc finger protein 418 (LOC730051), mRNA.
NM_001275.3	CHGA	-3.630	Homo sapiens chromogranin A (parathyroid secretory protein 1) (CHGA), mRNA.
NM_182728.1	SLC7A8	-3.621	Homo sapiens solute carrier family 7 (cationic amino acid transporter, y+ system), member 8 (SLC7A8), transcript variant 2, mRNA.
NM_003026.1	SH3GL2	-3.619	Homo sapiens SH3-domain GRB2-like 2 (SH3GL2), mRNA.

NM_170678.2	ITGB1BP3	-3.619	Homo sapiens integrin beta 1 binding protein 3 (ITGB1BP3), mRNA.
NM_019035.2	PCDH18	-3.618	Homo sapiens protocadherin 18 (PCDH18), mRNA.
XM_928013.1	ACBD7	-3.615	PREDICTED: Homo sapiens acyl-Coenzyme A binding domain containing 7 (ACBD7), mRNA.
NM_173798.2	ZCCHC12	-3.603	Homo sapiens zinc finger, CCHC domain containing 12 (ZCCHC12), mRNA.
XM_939725.2	LOC654127	-3.579	PREDICTED: Homo sapiens similar to adaptor-related protein complex 1 sigma 2 subunit (LOC654127), mRNA.
NR_029858.1	MIR302C	-3.577	Homo sapiens microRNA 302c (MIR302C), microRNA.
NM_005382.1	NEFM	-3.569	Homo sapiens neurofilament, medium polypeptide 150kDa (NEFM), mRNA.
NM_018602.2	DNAJA4	-3.567	Homo sapiens DnaJ (Hsp40) homolog, subfamily A, member 4 (DNAJA4), mRNA.
NM_002374.3	MAP2	-3.549	Homo sapiens microtubule-associated protein 2 (MAP2), transcript variant 1, mRNA.
NM_019013.1	FAM64A	-3.547	Homo sapiens family with sequence similarity 64, member A (FAM64A), mRNA.
NM_017753.2	LPPR1	-3.545	Homo sapiens lipid phosphate phosphatase-related protein type 1 (LPPR1), transcript variant 2, mRNA.
NM_024785.2	FAM124B	-3.535	Homo sapiens family with sequence similarity 124B (FAM124B), transcript variant 2, mRNA.
NM_003822.3	NR5A2	-3.531	Homo sapiens nuclear receptor subfamily 5, group A, member 2 (NR5A2), transcript variant 2, mRNA.
NM_024674.4	LIN28	-3.521	Homo sapiens lin-28 homolog (C. elegans) (LIN28), mRNA.
NM_002517.2	NPAS1	-3.489	Homo sapiens neuronal PAS domain protein 1 (NPAS1), mRNA.
NM_001005502.1	CPM	-3.482	Homo sapiens carboxypeptidase M (CPM), transcript variant 3, mRNA.
NM_032446.1	MEGF10	-3.480	Homo sapiens multiple EGF-like-domains 10 (MEGF10), mRNA.
NM_014996.1	PLCH1	-3.469	Homo sapiens phospholipase C, eta 1 (PLCH1), mRNA.
NM_003822.3	NR5A2	-3.456	Homo sapiens nuclear receptor subfamily 5, group A, member 2 (NR5A2), transcript variant 2, mRNA.
NM_002006.4	FGF2	-3.448	Homo sapiens fibroblast growth factor 2 (basic) (FGF2), mRNA.
NM_013243.2	SCG3	-3.442	Homo sapiens secretogranin III (SCG3), mRNA.
NM_030763.1	NSBP1	-3.440	Homo sapiens nucleosomal binding protein 1 (NSBP1), mRNA.
NM_181676.1	PPP2R2B	-3.438	Homo sapiens protein phosphatase 2 (formerly 2A), regulatory subunit B, beta isoform (PPP2R2B), transcript variant 4, mRNA.
NM_006467.2	POLR3G	-3.437	Homo sapiens polymerase (RNA) III (DNA directed) polypeptide G (32kD) (POLR3G), mRNA.
XM_946212.2	FLJ44379	-3.434	PREDICTED: Homo sapiens similar to S-100 protein, alpha chain, transcript variant 3 (FLJ44379), mRNA.
NR_003083.2	SLC6A10P	-3.432	Homo sapiens solute carrier family 6 (neurotransmitter transporter, creatine), member 10 (pseudogene) (SLC6A10P) on chromosome 16.
NM_013244.2	MGAT4C	-3.429	Homo sapiens mannosyl (alpha-1,3-)-glycoprotein beta-1,4-N-acetylglucosaminyltransferase, isozyme C (putative) (MGAT4C), mRNA.
NM_002130.6	HMGCS1	-3.410	Homo sapiens 3-hydroxy-3-methylglutaryl-Coenzyme A synthase 1 (soluble) (HMGCS1), transcript variant 2, mRNA.
NM_002738.5	PRKCB1	-3.405	Homo sapiens protein kinase C, beta 1 (PRKCB1), transcript variant 2, mRNA.
XM_001724965.1	LOC100129268	-3.404	PREDICTED: Homo sapiens hypothetical protein LOC100129268 (LOC100129268), mRNA.
NM_024749.2	VASH2	-3.399	Homo sapiens vasohibin 2 (VASH2), mRNA.
XM_001126803.1	LOC728185	-3.391	PREDICTED: Homo sapiens similar to Gamma-2-syntrophin (G2SYN) (Syntrophin 5) (SYN5) (LOC728185), mRNA.
NM_021992.2	TMSB15A	-3.388	Homo sapiens thymosin beta 15a (TMSB15A), mRNA.
NM_007223.1	GPR176	-3.377	Homo sapiens G protein-coupled receptor 176 (GPR176), mRNA.

XM_001128032.1	MGC87042	-3.369	PREDICTED: Homo sapiens similar to Six transmembrane epithelial antigen of prostate (MGC87042), mRNA.
NM_000814.4	GABRB3	-3.361	Homo sapiens gamma-aminobutyric acid (GABA) A receptor, beta 3 (GABRB3), transcript variant 1, mRNA.
NM_198545.3	C1orf187	-3.357	Homo sapiens chromosome 1 open reading frame 187 (C1orf187), mRNA.
NM_018189.3	DPPA4	-3.346	Homo sapiens developmental pluripotency associated 4 (DPPA4), mRNA.
NM_014618.2	DBC1	-3.339	Homo sapiens deleted in bladder cancer 1 (DBC1), mRNA.
NM_013244.2	MGAT4C	-3.334	Homo sapiens mannosyl (alpha-1,3-)-glycoprotein beta-1,4-N-acetylglucosaminyltransferase, isozyme C (putative) (MGAT4C), mRNA.
XR_079546.1	MGC11082	-3.337	PREDICTED: Homo sapiens hypothetical LOC84777 (MGC11082), miscRNA.
NM_006892.3	DNMT3B	-3.333	Homo sapiens DNA (cytosine-5-)-methyltransferase 3 beta (DNMT3B), transcript variant 1, mRNA.
NM_005769.1	CHST4	-3.329	Homo sapiens carbohydrate (N-acetylglucosamine 6-O) sulfotransferase 4 (CHST4), mRNA.
NM_152280.2	SYT11	-3.327	Homo sapiens synaptotagmin XI (SYT11), mRNA.
NM_003121.2	SPIB	-3.325	Homo sapiens Spi-B transcription factor (Spi-1/PU.1 related) (SPIB), mRNA.
NM_032505.1	KBTBD8	-3.314	Homo sapiens kelch repeat and BTB (POZ) domain containing 8 (KBTBD8), mRNA.
NM_205833.1	IGSF1	-3.298	Homo sapiens immunoglobulin superfamily, member 1 (IGSF1), transcript variant 1, mRNA.
NM_006000.1	TUBA4A	-3.291	Homo sapiens tubulin, alpha 4a (TUBA4A), mRNA.
XR_000901.1	FLJ33996	-3.280	PREDICTED: Homo sapiens hypothetical protein FLJ33996 (FLJ33996), misc RNA.
NM_012247.3	SEPHS1	-3.279	Homo sapiens selenophosphate synthetase 1 (SEPHS1), mRNA.
NM_020742.2	NLGN4X	-3.273	Homo sapiens neuroligin 4, X-linked (NLGN4X), transcript variant 1, mRNA.
NM_002463.1	MX2	-3.264	Homo sapiens myxovirus (influenza virus) resistance 2 (mouse) (MX2), mRNA.
XM_001726827.1	LOC100134361	-3.254	PREDICTED: Homo sapiens similar to hCG1811002 (LOC100134361), mRNA.
NM_144698.2	ANKRD35	-3.245	Homo sapiens ankyrin repeat domain 35 (ANKRD35), mRNA.
NM_001039948.2	SGSM1	-3.237	Homo sapiens small G protein signaling modulator 1 (SGSM1), transcript variant 1, mRNA.
NM_019035.2	PCDH18	-3.234	Homo sapiens protocadherin 18 (PCDH18), mRNA.
NM_017489.1	TERF1	-3.232	Homo sapiens telomeric repeat binding factor (NIMA-interacting) 1 (TERF1), transcript variant 2, mRNA.
NR_024031.1	KIAA0114	-3.225	Homo sapiens KIAA0114 (KIAA0114), non-coding RNA.
NM_020692.1	GALNTL1	-3.224	Homo sapiens UDP-N-acetyl-alpha-D-galactosamine:polypeptide N-acetylgalactosaminyltransferase-like 1 (GALNTL1), mRNA.
NM_021808.2	GALNT9	-3.214	Homo sapiens UDP-N-acetyl-alpha-D-galactosamine:polypeptide N-acetylgalactosaminyltransferase 9 (GalNAc-T9) (GALNT9), mRNA.
NM_004336.2	BUB1	-3.208	Homo sapiens BUB1 budding uninhibited by benzimidazoles 1 homolog (yeast) (BUB1), mRNA.
NM_000196.3	HSD11B2	-3.201	Homo sapiens hydroxysteroid (11-beta) dehydrogenase 2 (HSD11B2), mRNA.
NM_003112.3	SP4	-3.197	Homo sapiens Sp4 transcription factor (SP4), mRNA.
XR_038551.1	LOC389873	-3.197	PREDICTED: Homo sapiens misc_RNA (LOC389873), miscRNA.
NM_015253.1	WSCD1	-3.180	Homo sapiens WSC domain containing 1 (WSCD1), mRNA.
NM_080872.1	UNC5D	-3.170	Homo sapiens unc-5 homolog D (C. elegans) (UNC5D), mRNA.
NM_212535.2	PRKCB	-3.170	Homo sapiens protein kinase C, beta 1 (PRKCB1), transcript variant 2, mRNA.
NM_015568.2	PPP1R16B	-3.166	Homo sapiens protein phosphatase 1, regulatory (inhibitor) subunit 16B (PPP1R16B), mRNA.
NM_014790.3	JAKMIP2	-3.162	Homo sapiens janus kinase and microtubule interacting protein 2 (JAKMIP2), mRNA.

NM_005193.1	CDX4	-3.162	Homo sapiens caudal type homeobox 4 (CDX4), mRNA.
NM_152359.1	CPT1C	-3.160	Homo sapiens carnitine palmitoyltransferase 1C (CPT1C), transcript variant 2, mRNA.
NM_006174.2	NPY5R	-3.157	Homo sapiens neuropeptide Y receptor Y5 (NPY5R), mRNA.
NM_001018109.1	PIR	-3.150	Homo sapiens pirin (iron-binding nuclear protein) (PIR), transcript variant 2, mRNA.
NM_018977.2	NLGN3	-3.136	Homo sapiens neuroligin 3 (NLGN3), mRNA.
NM_001008273.1	TAGLN3	-3.131	Homo sapiens transgelin 3 (TAGLN3), transcript variant 3, mRNA.
NM_002600.3	PDE4B	-3.126	Homo sapiens phosphodiesterase 4B, cAMP-specific (phosphodiesterase E4 dunce homolog, Drosophila) (PDE4B), transcript variant a, mRNA.
NM_004181.3	UCHL1	-3.114	Homo sapiens ubiquitin carboxyl-terminal esterase L1 (ubiquitin thiolesterase) (UCHL1), mRNA.
NM_174976.2	ZDHHC22	-3.110	Homo sapiens zinc finger, DHHC-type containing 22 (ZDHHC22), mRNA.
NM_003360.2	UGT8	-3.102	Homo sapiens UDP glycosyltransferase 8 (UDP-galactose ceramide galactosyltransferase) (UGT8), mRNA.
NM_033222.2	PSIP1	-3.098	Homo sapiens PC4 and SFRS1 interacting protein 1 (PSIP1), transcript variant 2, mRNA.
NM_001040167.1	LFNG	-3.097	Homo sapiens LFNG O-fucosylpeptide 3-beta-N-acetylglucosaminyltransferase (LFNG), transcript variant 1, mRNA.
NM_001555.2	IGSF1	-3.086	Homo sapiens immunoglobulin superfamily, member 1 (IGSF1), transcript variant 1, mRNA.
NM_005110.1	GFPT2	-3.086	Homo sapiens glutamine-fructose-6-phosphate transaminase 2 (GFPT2), mRNA.
NM_004946.1	DOCK2	-3.084	Homo sapiens dedicator of cytokinesis 2 (DOCK2), mRNA.
NM_152999.2	STEAP2	-3.082	Homo sapiens six transmembrane epithelial antigen of the prostate 2 (STEAP2), mRNA.
NM_198336.1	INSIG1	-3.076	Homo sapiens insulin induced gene 1 (INSIG1), transcript variant 2, mRNA.
NM_021127.1	PMAIP1	-3.074	Homo sapiens phorbol-12-myristate-13-acetate-induced protein 1 (PMAIP1), mRNA.
NM_005378.4	MYCN	-3.074	Homo sapiens v-myc myelocytomatosis viral related oncogene, neuroblastoma derived (avian) (MYCN), mRNA.
NM_006426.1	DPYSL4	-3.073	Homo sapiens dihydropyrimidinase-like 4 (DPYSL4), mRNA.
NM_152889.1	CHST13	-3.067	Homo sapiens carbohydrate (chondroitin 4) sulfotransferase 13 (CHST13), mRNA.
NM_005440.3	RND2	-3.064	Homo sapiens Rho family GTPase 2 (RND2), mRNA.
NM_005378.4	MYCN	-3.056	Homo sapiens v-myc myelocytomatosis viral related oncogene, neuroblastoma derived (avian) (MYCN), mRNA.
NM_024629.2	MLF1IP	-3.047	Homo sapiens MLF1 interacting protein (MLF1IP), mRNA.
NM_207406.2	BEND4	-3.047	Homo sapiens BEN domain containing 4 (BEND4), mRNA.
NM_213647.1	FGFR4	-3.046	Homo sapiens fibroblast growth factor receptor 4 (FGFR4), transcript variant 3, mRNA.
NM_198545.2	C1orf187	-3.044	Homo sapiens chromosome 1 open reading frame 187 (C1orf187), mRNA.
NM_030631.2	SLC25A21	-3.040	Homo sapiens solute carrier family 25 (mitochondrial oxodicarboxylate carrier), member 21 (SLC25A21), nuclear gene encoding mitochondrial protein, mRNA.
NM_006286.1	TFDP2	-3.034	Homo sapiens transcription factor Dp-2 (E2F dimerization partner 2) (TFDP2), mRNA.
NM_024865.1	NANOG	-3.033	Homo sapiens Nanog homeobox (NANOG), mRNA.
NM_012449.2	STEAP1	-3.015	Homo sapiens six transmembrane epithelial antigen of the prostate 1 (STEAP1), mRNA.
NM_001080450.1	BEND3	-3.014	Homo sapiens BEN domain containing 3 (BEND3), mRNA.
NM_182974.2	GLT6D1	-3.013	Homo sapiens glycosyltransferase 6 domain containing 1 (GLT6D1), mRNA.
NM_019886.2	CHST7	-3.007	Homo sapiens carbohydrate (N-acetylglucosamine 6-O) sulfotransferase 7

			(CHST7), mRNA.
NM_002045.2	GAP43	-3.001	Homo sapiens growth associated protein 43 (GAP43), mRNA.
NM_182830.2	MDGA2	-2.994	Homo sapiens MAM domain containing glycosylphosphatidylinositol anchor 2 (MDGA2), mRNA.
NM_152322.2	BTBD11	-2.992	Homo sapiens BTB (POZ) domain containing 11 (BTBD11), transcript variant 1, mRNA.
NM_004615.2	TSPAN7	-2.986	Homo sapiens tetraspanin 7 (TSPAN7), mRNA.
NM_033201.1	C16orf45	-2.978	Homo sapiens chromosome 16 open reading frame 45 (C16orf45), mRNA.
NM_018645.3	HES6	-2.977	Homo sapiens hairy and enhancer of split 6 (Drosophila) (HES6), mRNA.
NM_015225.1	KIAA0367	-2.975	Homo sapiens KIAA0367 (KIAA0367), mRNA.
NM_017954.9	CADPS2	-2.975	Homo sapiens Ca++-dependent secretion activator 2 (CADPS2), transcript variant 1, mRNA.
NM_012083.2	FRAT2	-2.973	Homo sapiens frequently rearranged in advanced T-cell lymphomas 2 (FRAT2), mRNA.
NM_014431.1	KIAA1274	-2.970	Homo sapiens KIAA1274 (KIAA1274), mRNA.
NM_032882.2	PNMA6A	-2.964	Homo sapiens paraneoplastic antigen like 6A (PNMA6A), mRNA.
NM_000745.2	CHRNA5	-2.959	Homo sapiens cholinergic receptor, nicotinic, alpha 5 (CHRNA5), mRNA.
NM_014932.2	NLGN1	-2.943	Homo sapiens neuroligin 1 (NLGN1), mRNA.
NM_000814.4	GABRB3	-2.940	Homo sapiens gamma-aminobutyric acid (GABA) A receptor, beta 3 (GABRB3), transcript variant 1, mRNA.
NM_005329.2	HAS3	-2.939	Homo sapiens hyaluronan synthase 3 (HAS3), transcript variant 1, mRNA.
XM_001725504.1	LOC100127971	-2.938	PREDICTED: Homo sapiens hypothetical protein LOC100127971 (LOC100127971), mRNA.
XR_039217.1	LOC100130769	-2.936	PREDICTED: Homo sapiens misc_RNA (LOC100130769), miscRNA.
NM_001814.2	CTSC	-2.935	Homo sapiens cathepsin C (CTSC), transcript variant 1, mRNA.
NM_004497.2	FOXA3	-2.934	Homo sapiens forkhead box A3 (FOXA3), mRNA.
NM_024534.4	FLJ12684	-2.931	Homo sapiens hypothetical protein FLJ12684 (FLJ12684), mRNA. XR_001254
NM_199478.1	PLP1	-2.928	Homo sapiens proteolipid protein 1 (PLP1), transcript variant 2, mRNA.
NM_024645.2	ZMAT4	-2.922	Homo sapiens zinc finger, matrin type 4 (ZMAT4), transcript variant 1, mRNA.
NM_014906.3	PPM1E	-2.917	Homo sapiens protein phosphatase 1E (PP2C domain containing) (PPM1E), mRNA.
NM_024333.1	FSD1	-2.913	Homo sapiens fibronectin type III and SPRY domain containing 1 (FSD1), mRNA.
NM_000903.2	NQO1	-2.913	Homo sapiens NAD(P)H dehydrogenase, quinone 1 (NQO1), transcript variant 1, mRNA.
NM_001039111.1	TRIM71	-2.912	Homo sapiens tripartite motif-containing 71 (TRIM71), mRNA.
NM_001814.2	CTSC	-2.911	Homo sapiens cathepsin C (CTSC), transcript variant 1, mRNA.
NM_017711.2	GDPD2	-2.906	Homo sapiens glycerophosphodiester phosphodiesterase domain containing 2 (GDPD2), mRNA.
NM_133474.2	ZNF721	-2.903	Homo sapiens zinc finger protein 721 (ZNF721), mRNA.
NM_018659.2	CYTL1	-2.901	Homo sapiens cytokine-like 1 (CYTL1), mRNA.
NM_178562.2	TSPAN33	-2.891	Homo sapiens tetraspanin 33 (TSPAN33), mRNA.
NM_005891.2	ACAT2	-2.890	Homo sapiens acetyl-Coenzyme A acetyltransferase 2 (ACAT2), mRNA.
NM_001097635.1	GCNT1	-2.887	Homo sapiens glucosaminyl (N-acetyl) transferase 1, core 2 (beta-1,6-N-acetylglucosaminyltransferase) (GCNT1), transcript variant 4, mRNA.
NM_004297.2	GNA14	-2.886	Homo sapiens guanine nucleotide binding protein (G protein), alpha 14 (GNA14), mRNA.
XR_037397.1	LOC100129267	-2.881	PREDICTED: Homo sapiens misc_RNA (LOC100129267), miscRNA.
NM_153034.2	ZNF488	-2.879	Homo sapiens zinc finger protein 488 (ZNF488), mRNA.

NM_017669.2	ERCC6L	-2.871	Homo sapiens excision repair cross-complementing rodent repair deficiency, complementation group 6-like (ERCC6L), mRNA.
XM_497029.2	LOC441408	-2.871	PREDICTED: Homo sapiens hypothetical LOC441408, transcript variant 1 (LOC441408), mRNA.
XM_372255.2	LOC389895	-2.870	PREDICTED: Homo sapiens similar to CG4768-PA (LOC389895), mRNA.
NM_022357.1	DPEP3	-2.866	Homo sapiens dipeptidase 3 (DPEP3), mRNA.
NM_182553.1	CNIH2	-2.865	Homo sapiens cornichon homolog 2 (Drosophila) (CNIH2), mRNA.
NM_182970.2	RIMS4	-2.861	Homo sapiens regulating synaptic membrane exocytosis 4 (RIMS4), mRNA.
NM_173078.2	SLITRK4	-2.858	Homo sapiens SLIT and NTRK-like family, member 4 (SLITRK4), mRNA.
NM_005760.2	CEBPZ	-2.847	Homo sapiens CCAAT/enhancer binding protein (C/EBP), zeta (CEBPZ), mRNA.
NM_152999.3	STEAP2	-2.846	Homo sapiens six transmembrane epithelial antigen of the prostate 2 (STEAP2), mRNA.
NM_001018054.1	LRP8	-2.842	Homo sapiens low density lipoprotein receptor-related protein 8, apolipoprotein e receptor (LRP8), transcript variant 4, mRNA.
NM_018169.2	C12orf35	-2.841	Homo sapiens chromosome 12 open reading frame 35 (C12orf35), mRNA.
NM_015028.1	TNIK	-2.839	Homo sapiens TRAF2 and NCK interacting kinase (TNIK), mRNA.
NM_021615.4	CHST6	-2.838	Homo sapiens carbohydrate (N-acetylglucosamine 6-O) sulfotransferase 6 (CHST6), mRNA.
NM_001078174.1	SLC29A1	-2.833	Homo sapiens solute carrier family 29 (nucleoside transporters), member 1 (SLC29A1), nuclear gene encoding mitochondrial protein, transcript variant 4, mRNA.
NM_017745.4	BCOR	-2.829	Homo sapiens BCL6 co-repressor (BCOR), transcript variant 1, mRNA.
NM_004664.2	LIN7A	-2.828	Homo sapiens lin-7 homolog A (C. elegans) (LIN7A), mRNA.
NM_198353.1	KCTD8	-2.828	Homo sapiens potassium channel tetramerisation domain containing 8 (KCTD8), mRNA.
NM_015162.3	ACSBG1	-2.826	Homo sapiens acyl-CoA synthetase bubblegum family member 1 (ACSBG1), mRNA.
NM_004378.1	CRABP1	-2.824	Homo sapiens cellular retinoic acid binding protein 1 (CRABP1), mRNA.
NM_005375.2	MYB	-2.823	Homo sapiens v-myb myeloblastosis viral oncogene homolog (avian) (MYB), transcript variant 2, mRNA.
NM_016582.1	SLC15A3	-2.820	Homo sapiens solute carrier family 15, member 3 (SLC15A3), mRNA.
NM_001004196.2	CD200	-2.818	Homo sapiens CD200 molecule (CD200), transcript variant 2, mRNA.
NM_001031806.1	ALDH3A2	-2.817	Homo sapiens aldehyde dehydrogenase 3 family, member A2 (ALDH3A2), transcript variant 2, mRNA.
NM_006288.2	THY1	-2.814	Homo sapiens Thy-1 cell surface antigen (THY1), mRNA.
NM_024595.1	AKIRIN1	-2.810	Homo sapiens akirin 1 (AKIRIN1), mRNA.
NM_170678.2	ITGB1BP3	-2.805	Homo sapiens integrin beta 1 binding protein 3 (ITGB1BP3), mRNA.
XM_929774.2	LOC646817	-2.800	PREDICTED: Homo sapiens similar to Protein SET (Phosphatase 2A inhibitor I2PP2A) (I-2PP2A) (Template-activating factor I) (TAF-I) (HLA-DR-associated protein II) (PHAPII) (Inhibitor of granzyme A-activated DNase) (IGAAD) (LOC646817), mRNA.
NM_014921.3	LPHN1	-2.798	Homo sapiens latrophilin 1 (LPHN1), transcript variant 2, mRNA.
NM_013264.2	DDX25	-2.791	Homo sapiens DEAD (Asp-Glu-Ala-Asp) box polypeptide 25 (DDX25), mRNA.
NM_020925.2	CACHD1	-2.790	Homo sapiens cache domain containing 1 (CACHD1), mRNA.
NM_003716.2	CADPS	-2.790	Homo sapiens Ca2+-dependent secretion activator (CADPS), transcript variant 1, mRNA.
NM_000530.3	MPZ	-2.781	Homo sapiens myelin protein zero (Charcot-Marie-Tooth neuropathy 1B) (MPZ), mRNA.
NM_022468.4	MMP25	-2.780	Homo sapiens matrix metallopeptidase 25 (MMP25), mRNA.
NM_012247.3	SEPHS1	-2.773	Homo sapiens selenophosphate synthetase 1 (SEPHS1), mRNA.

NM_000904.2	NQO2	-2.770	Homo sapiens NAD(P)H dehydrogenase, quinone 2 (NQO2), mRNA.
NM_020742.2	NLGN4X	-2.763	Homo sapiens neuroligin 4, X-linked (NLGN4X), transcript variant 1, mRNA.
NM_001017430.1	RBM3	-2.762	Homo sapiens RNA binding motif (RNP1, RRM) protein 3 (RBM3), transcript variant 2, mRNA.
NM_016089.2	ZNF589	-2.762	Homo sapiens zinc finger protein 589 (ZNF589), mRNA.
NM_001360.1	DHCR7	-2.757	Homo sapiens 7-dehydrocholesterol reductase (DHCR7), mRNA.
NM_000995.2	RPL34	-2.753	Homo sapiens ribosomal protein L34 (RPL34), transcript variant 1, mRNA.
NM_001360.2	DHCR7	-2.749	Homo sapiens 7-dehydrocholesterol reductase (DHCR7), mRNA.
NM_006739.3	MCM5	-2.746	Homo sapiens minichromosome maintenance complex component 5 (MCM5), mRNA.
XR_041527.1	ZNF788	-2.742	PREDICTED: Homo sapiens misc_RNA (ZNF788), miscRNA.
NM_017549.3	EPDR1	-2.740	Homo sapiens ependymin related protein 1 (zebrafish) (EPDR1), mRNA.
NM_021614.2	KCNN2	-2.736	Homo sapiens potassium intermediate/small conductance calcium-activated channel, subfamily N, member 2 (KCNN2), transcript variant 1, mRNA.
XM_001720872.1	LOC100127971	-2.730	PREDICTED: Homo sapiens hypothetical protein LOC100127971 (LOC100127971), mRNA.
BC038245		-2.729	Homo sapiens, clone IMAGE:5241654, mRNA
NM_145018.2	C11orf82	-2.721	Homo sapiens chromosome 11 open reading frame 82 (C11orf82), mRNA.
NM_005678.3	SNURF	-2.711	Homo sapiens SNRPN upstream reading frame (SNURF), transcript variant 1, mRNA.
NM_058237.1	PPP4R4	-2.703	Homo sapiens protein phosphatase 4, regulatory subunit 4 (PPP4R4), transcript variant 1, mRNA.
XM_001715544.1	LOC100129673	-2.700	PREDICTED: Homo sapiens similar to hCG2042915 (LOC100129673), mRNA.
NM_019106.4	SEPT3	-2.699	Homo sapiens septin 3 (SEPT3), transcript variant B, mRNA.
NM_006892.3	DNMT3B	-2.699	Homo sapiens DNA (cytosine-5-)-methyltransferase 3 beta (DNMT3B), transcript variant 1, mRNA.
NM_152312.3	GYLTL1B	-2.697	Homo sapiens glycosyltransferase-like 1B (GYLTL1B), mRNA.
NM_018644.3	B3GAT1	-2.697	Homo sapiens beta-1,3-glucuronyltransferase 1 (glucuronosyltransferase P) (B3GAT1), transcript variant 1, mRNA.
NM_152672.4	OSTalpha	-2.694	Homo sapiens organic solute transporter alpha (OSTalpha), mRNA.
NM_012113.1	CA14	-2.693	Homo sapiens carbonic anhydrase XIV (CA14), mRNA.
NM_000382.2	ALDH3A2	-2.690	Homo sapiens aldehyde dehydrogenase 3 family, member A2 (ALDH3A2), transcript variant 2, mRNA.
NM_207406.1	CCDC4	-2.690	Homo sapiens coiled-coil domain containing 4 (CCDC4), mRNA.
NM_153183.1	NUDT10	-2.682	Homo sapiens nudix (nucleoside diphosphate linked moiety X)-type motif 10 (NUDT10), mRNA.
NM_001048201.1	UHRF1	-2.679	Homo sapiens ubiquitin-like with PHD and ring finger domains 1 (UHRF1), transcript variant 1, mRNA.
NM_030639.1	BHLHB9	-2.679	Homo sapiens basic helix-loop-helix domain containing, class B, 9 (BHLHB9), mRNA.
NM_001078174.1	SLC29A1	-2.676	Homo sapiens solute carrier family 29 (nucleoside transporters), member 1 (SLC29A1), nuclear gene encoding mitochondrial protein, transcript variant 4, mRNA.
NM_153183.2	NUDT10	-2.675	Homo sapiens nudix (nucleoside diphosphate linked moiety X)-type motif 10 (NUDT10), mRNA.
NM_001015001.1	CKMT1A	-2.674	Homo sapiens creatine kinase, mitochondrial 1A (CKMT1A), nuclear gene encoding mitochondrial protein, mRNA.
NM_022449.1	RAB17	-2.674	Homo sapiens RAB17, member RAS oncogene family (RAB17), mRNA.
NM_018365.1	MNS1	-2.674	Homo sapiens meiosis-specific nuclear structural 1 (MNS1), mRNA.
NM_000399.2	EGR2	-2.669	Homo sapiens early growth response 2 (Krox-20 homolog, Drosophila) (EGR2), mRNA.

NM_024719.2	GRTP1	-2.669	Homo sapiens growth hormone regulated TBC protein 1 (GRTP1), mRNA.
NM_000170.2	GLDC	-2.663	Homo sapiens glycine dehydrogenase (decarboxylating) (GLDC), mRNA.
NM_001441.1	FAAH	-2.663	Homo sapiens fatty acid amide hydrolase (FAAH), mRNA.
NM_024693.2	ECHDC3	-2.662	Homo sapiens enoyl Coenzyme A hydratase domain containing 3 (ECHDC3), mRNA.
NM_003385.4	VSNL1	-2.662	Homo sapiens visinin-like 1 (VSNL1), mRNA.
NM_006286.1	TFDP2	-2.661	Homo sapiens transcription factor Dp-2 (E2F dimerization partner 2) (TFDP2), mRNA.
NM_004319.1	ASTN1	-2.660	Homo sapiens astrotactin 1 (ASTN1), transcript variant 1, mRNA.
NM_138375.1	CABLES1	-2.656	Homo sapiens Cdk5 and Abl enzyme substrate 1 (CABLES1), transcript variant 1, mRNA.
NM_001104.1	ACTN3	-2.652	Homo sapiens actinin, alpha 3 (ACTN3), mRNA.
NM_152649.1	MLKL	-2.651	Homo sapiens mixed lineage kinase domain-like (MLKL), mRNA.
NM_145006.2	SUSD3	-2.648	Homo sapiens sushi domain containing 3 (SUSD3), mRNA.
NM_032488.2	CNFN	-2.644	Homo sapiens cornifelin (CNFN), mRNA.
NM_138576.2	BCL11B	-2.642	Homo sapiens B-cell CLL/lymphoma 11B (zinc finger protein) (BCL11B), transcript variant 1, mRNA.
NM_138409.1	C6orf117	-2.638	Homo sapiens chromosome 6 open reading frame 117 (C6orf117), mRNA.
NM_198336.1	INSIG1	-2.636	Homo sapiens insulin induced gene 1 (INSIG1), transcript variant 2, mRNA.
NM_139354.2	MATK	-2.631	Homo sapiens megakaryocyte-associated tyrosine kinase (MATK), transcript variant 3, mRNA.
NM_033259.2	CAMK2N2	-2.632	Homo sapiens calcium/calmodulin-dependent protein kinase II inhibitor 2 (CAMK2N2), mRNA.
NM_000459.2	TEK	-2.620	Homo sapiens TEK tyrosine kinase, endothelial (venous malformations, multiple cutaneous and mucosal) (TEK), mRNA.
NM_018644.3	B3GAT1	-2.619	Homo sapiens beta-1,3-glucuronyltransferase 1 (glucuronosyltransferase P) (B3GAT1), transcript variant 1, mRNA.
NM_019079.2	L1TD1	-2.616	Homo sapiens LINE-1 type transposase domain containing 1 (L1TD1), mRNA.
NM_024794.1	ABHD9	-2.612	Homo sapiens abhydrolase domain containing 9 (ABHD9), mRNA.
NM_014750.3	DLGAP5	-2.609	Homo sapiens discs, large (Drosophila) homolog-associated protein 5 (DLGAP5), mRNA.
NM_080878.2	ITLN2	-2.607	Homo sapiens intelectin 2 (ITLN2), mRNA.
NM_006087.2	TUBB4	-2.602	Homo sapiens tubulin, beta 4 (TUBB4), mRNA.
NM_005824.1	LRRC17	-2.594	Homo sapiens leucine rich repeat containing 17 (LRRC17), transcript variant 2, mRNA.
NM_020436.2	SALL4	-2.594	Homo sapiens sal-like 4 (Drosophila) (SALL4), mRNA.
NM_152429.3	FGFBP3	-2.593	Homo sapiens fibroblast growth factor binding protein 3 (FGFBP3), mRNA.
NM_022806.2	SNRPN	-2.592	Homo sapiens small nuclear ribonucleoprotein polypeptide N (SNRPN), transcript variant 3, mRNA.
NM_020836.2	BEGAIN	-2.591	Homo sapiens brain-enriched guanylate kinase-associated homolog (rat) (BEGAIN), mRNA.
CR738291		-2.586	CR738291 Homo sapiens library (Ebert L) Homo sapiens cDNA clone IMAGp971D2453 ; IMAGE:811744 5, mRNA sequence
NM_003385.4	VSNL1	-2.586	Homo sapiens visinin-like 1 (VSNL1), mRNA.
NM_001011645.1	AR	-2.585	Homo sapiens androgen receptor (AR), transcript variant 2, mRNA.
NM_144584.1	C1orf59	-2.583	Homo sapiens chromosome 1 open reading frame 59 (C1orf59), mRNA.
CR603272		-2.578	full-length cDNA clone CS0DC013YI10 of Neuroblastoma Cot 25-normalized of Homo sapiens (human)
NM_007115.2	TNFAIP6	-2.577	Homo sapiens tumor necrosis factor, alpha-induced protein 6 (TNFAIP6), mRNA.

NM_015678.3	NBEA	-2.575	Homo sapiens neurobeachin (NBEA), mRNA.
NR_015379.2	UCA1	-2.575	Homo sapiens urothelial cancer associated 1 (non-protein coding) (UCA1), non-coding RNA.
NM_007280.1	OIP5	-2.568	Homo sapiens Opa interacting protein 5 (OIP5), mRNA.
NM_012202.1	GNG3	-2.567	Homo sapiens guanine nucleotide binding protein (G protein), gamma 3 (GNG3), mRNA.
NM_014696.2	KIAA0514	-2.564	Homo sapiens KIAA0514 (KIAA0514), mRNA.
NM_144575.2	CAPN13	-2.564	Homo sapiens calpain 13 (CAPN13), mRNA.
NM_000459.1	TEK	-2.561	Homo sapiens TEK tyrosine kinase, endothelial (venous malformations, multiple cutaneous and mucosal) (TEK), mRNA.
NM_005269.1	GLI1	-2.551	Homo sapiens glioma-associated oncogene homolog 1 (zinc finger protein) (GLI1), mRNA.
NM_002482.2	NASP	-2.550	Homo sapiens nuclear autoantigenic sperm protein (histone-binding) (NASP), transcript variant 2, mRNA.
NM_016447.2	MPP6	-2.548	Homo sapiens membrane protein, palmitoylated 6 (MAGUK p55 subfamily member 6) (MPP6), mRNA.
XM_209196.5	LOC284422	-2.547	PREDICTED: Homo sapiens similar to HSPC323 (LOC284422), mRNA.
NM_007280.1	OIP5	-2.545	Homo sapiens Opa interacting protein 5 (OIP5), mRNA.
NM_018361.2	AGPAT5	-2.540	Homo sapiens 1-acylglycerol-3-phosphate O-acyltransferase 5 (lysophosphatidic acid acyltransferase, epsilon) (AGPAT5), mRNA.
NM_138967.2	SCAMP5	-2.534	Homo sapiens secretory carrier membrane protein 5 (SCAMP5), mRNA.
NM_022652.2	DUSP6	-2.532	Homo sapiens dual specificity phosphatase 6 (DUSP6), transcript variant 2, mRNA.
NM_003543.3	HIST1H4H	-2.531	Homo sapiens histone cluster 1, H4h (HIST1H4H), mRNA.
NM_001006625.1	PDPN	-2.531	Homo sapiens podoplanin (PDPN), transcript variant 4, mRNA.
NM_018719.2	CDCA7L	-2.530	Homo sapiens cell division cycle associated 7-like (CDCA7L), mRNA.
NM_017915.2	C12orf48	-2.527	Homo sapiens chromosome 12 open reading frame 48 (C12orf48), mRNA.
NM_057162.1	KLHL4	-2.526	Homo sapiens kelch-like 4 (Drosophila) (KLHL4), transcript variant 2, mRNA.
NM_198445.2	RINL	-2.526	Homo sapiens Ras and Rab interactor-like (RINL), mRNA.
NM_018369.1	DEPDC1B	-2.525	Homo sapiens DEP domain containing 1B (DEPDC1B), mRNA.
XR_041972.1	C10orf75	-2.523	PREDICTED: Homo sapiens misc_RNA (C10orf75), miscRNA.
NM_006759.3	UGP2	-2.523	Homo sapiens UDP-glucose pyrophosphorylase 2 (UGP2), transcript variant 1, mRNA.
NM_014762.3	DHCR24	-2.522	Homo sapiens 24-dehydrocholesterol reductase (DHCR24), mRNA.
NM_001034954.1	SORBS1	-2.521	Homo sapiens sorbin and SH3 domain containing 1 (SORBS1), transcript variant 3, mRNA.
NM_016567.2	BCCIP	-2.521	Homo sapiens BRCA2 and CDKN1A interacting protein (BCCIP), transcript variant A, mRNA.
NM_133369.2	UNC5A	-2.519	Homo sapiens unc-5 homolog A (C. elegans) (UNC5A), mRNA.
NM_145061.3	C13orf3	-2.518	Homo sapiens chromosome 13 open reading frame 3 (C13orf3), mRNA.
NM_006950.3	SYN1	-2.517	Homo sapiens synapsin I (SYN1), transcript variant Ia, mRNA.
NM_006681.1	NMU	-2.515	Homo sapiens neuromedin U (NMU), mRNA.
XM_001134195.2	LOC147646	-2.515	PREDICTED: Homo sapiens hypothetical protein LOC147646 (LOC147646), mRNA.
NM_001002919.1	LOC285016	-2.498	Homo sapiens hypothetical protein LOC285016 (LOC285016), mRNA.
NM_001012978.1	BEX5	-2.497	Homo sapiens brain expressed, X-linked 5 (BEX5), mRNA.
NM_002119.3	HLA-DOA	-2.495	Homo sapiens major histocompatibility complex, class II, DO alpha (HLA-DOA), mRNA.
NM_001099645.1	RPL22L1	-2.494	Homo sapiens ribosomal protein L22-like 1 (RPL22L1), mRNA.

NM_018369.1	DEPDC1B	-2.489	Homo sapiens DEP domain containing 1B (DEPDC1B), mRNA.
U56251		-2.485	Human HeLa mRNA isolated as a false positive in a two-hybrid-screen
NM_199478.1	PLP1	-2.481	Homo sapiens proteolipid protein 1 (PLP1), transcript variant 2, mRNA.
NM_022805.2	SNRPN	-2.477	Homo sapiens small nuclear ribonucleoprotein polypeptide N (SNRPN), transcript variant 3, mRNA.
NM_001002844.1	ZNF280D	-2.469	Homo sapiens zinc finger protein 280D (ZNF280D), transcript variant 3, mRNA.
XM_933918.1	LOC646769	-2.469	PREDICTED: Homo sapiens hypothetical protein LOC646769 (LOC646769), mRNA.
NM_153768.1	CABYR	-2.458	Homo sapiens calcium binding tyrosine-(Y)-phosphorylation regulated (CABYR), transcript variant 2, mRNA.
NM_018063.3	HELLS	-2.451	Homo sapiens helicase, lymphoid-specific (HELLS), mRNA.
NM_012484.1	HMMR	-2.441	Homo sapiens hyaluronan-mediated motility receptor (RHAMM) (HMMR), transcript variant 1, mRNA.
NM_004448.2	ERBB2	-2.438	Homo sapiens v-erb-b2 erythroblastic leukemia viral oncogene homolog 2, neuro/glioblastoma derived oncogene homolog (avian) (ERBB2), transcript variant 1, mRNA.
XM_001723748.1	LOC100133489	-2.437	PREDICTED: Homo sapiens similar to hCG1983233 (LOC100133489), mRNA.
NM_004526.2	MCM2	-2.425	Homo sapiens minichromosome maintenance complex component 2 (MCM2), mRNA.
NM_022806.2	SNRPN	-2.418	Homo sapiens small nuclear ribonucleoprotein polypeptide N (SNRPN), transcript variant 3, mRNA.
NM_004426.2	PHC1	-2.415	Homo sapiens polyhomeotic homolog 1 (Drosophila) (PHC1), mRNA.
NM_021190.1	PTBP2	-2.402	Homo sapiens polypyrimidine tract binding protein 2 (PTBP2), mRNA.
NM_032776.1	JMJD1C	-2.398	Homo sapiens jumonji domain containing 1C (JMJD1C), transcript variant 1, mRNA.
NM_022770.2	GINS3	-2.397	Homo sapiens GINS complex subunit 3 (Psf3 homolog) (GINS3), mRNA.
NM_004973.2	JARID2	-2.383	Homo sapiens jumonji, AT rich interactive domain 2 (JARID2), mRNA.
NM_014750.3	DLGAP5	-2.380	Homo sapiens discs, large (Drosophila) homolog-associated protein 5 (DLGAP5), mRNA.
NM_002116.5	HLA-A	-2.377	Homo sapiens major histocompatibility complex, class I, A (HLA-A), mRNA.
NM_001124.1	ADM	-2.369	Homo sapiens adrenomedullin (ADM), mRNA.
NM_006729.3	DIAPH2	-2.362	Homo sapiens diaphanous homolog 2 (Drosophila) (DIAPH2), transcript variant 156, mRNA.
NM_001168.2	BIRC5	-2.358	Homo sapiens baculoviral IAP repeat-containing 5 (BIRC5), transcript variant 1, mRNA.
XM_939056.2	LOC649970	-2.354	PREDICTED: Homo sapiens similar to creatine kinase, mitochondrial 1B precursor (LOC649970), mRNA.
NM_005189.1	CBX2	-2.348	Homo sapiens chromobox homolog 2 (Pc class homolog, Drosophila) (CBX2), transcript variant 1, mRNA.
NM_003916.3	AP1S2	-2.341	Homo sapiens adaptor-related protein complex 1, sigma 2 subunit (AP1S2), mRNA.
NM_012238.3	SIRT1	-2.341	Homo sapiens sirtuin (silent mating type information regulation 2 homolog) 1 (S. cerevisiae) (SIRT1), mRNA.
NM_030919.2	FAM83D	-2.339	Homo sapiens family with sequence similarity 83, member D (FAM83D), mRNA.
NM_003916.3	AP1S2	-2.334	Homo sapiens adaptor-related protein complex 1, sigma 2 subunit (AP1S2), mRNA.
NM_001274.3	CHEK1	-2.326	Homo sapiens CHK1 checkpoint homolog (S. pombe) (CHEK1), mRNA.
NM_030755.4	TMX1	-2.325	Homo sapiens thioredoxin-related transmembrane protein 1 (TMX1), mRNA.
NM_000165.3	GJA1	-2.325	Homo sapiens gap junction protein, alpha 1, 43kDa (GJA1), mRNA.
NM_012485.1	HMMR	-2.322	Homo sapiens hyaluronan-mediated motility receptor (RHAMM) (HMMR), transcript variant 2, mRNA.

NM_003318.3	TTK	-2.311	Homo sapiens TTK protein kinase (TTK), mRNA.
NM_005915.4	MCM6	-2.311	Homo sapiens minichromosome maintenance complex component 6 (MCM6), mRNA.
NM_005513.1	GTF2E1	-2.299	Homo sapiens general transcription factor IIE, polypeptide 1 (alpha subunit, 56kD) (GTF2E1), mRNA.
NM_000859.1	HMGCR	-2.288	Homo sapiens 3-hydroxy-3-methylglutaryl-Coenzyme A reductase (HMGCR), mRNA.
NM_174900.3	ZFP42	-2.269	Homo sapiens zinc finger protein 42 homolog (mouse) (ZFP42), mRNA.
NM_005342.2	HMGB3	-2.261	Homo sapiens high-mobility group box 3 (HMGB3), mRNA.
NM_015570.1	AUTS2	-2.257	Homo sapiens autism susceptibility candidate 2 (AUTS2), mRNA.
NM_033625.2	RPL34	-2.251	Homo sapiens ribosomal protein L34 (RPL34), transcript variant 2, mRNA.
NM_003032.2	ST6GAL1	-2.232	Homo sapiens ST6 beta-galactosamide alpha-2,6-sialyltranferase 1 (ST6GAL1), transcript variant 2, mRNA.
NM_001033059.1	AMD1	-2.201	Homo sapiens adenosylmethionine decarboxylase 1 (AMD1), transcript variant 2, mRNA.
NM_003362.2	UNG	-2.177	Homo sapiens uracil-DNA glycosylase (UNG), nuclear gene encoding mitochondrial protein, transcript variant 1, mRNA.
NM_004321.4	KIF1A	-2.172	Homo sapiens kinesin family member 1A (KIF1A), mRNA.
NM_001212.3	C1QBP	-2.105	Homo sapiens complement component 1, q subcomponent binding protein (C1QBP), nuclear gene encoding mitochondrial protein, mRNA.
NM_005896.2	IDH1	-2.062	Homo sapiens isocitrate dehydrogenase 1 (NADP+), soluble (IDH1), mRNA.
NM_001448.2	GPC4	-2.047	Homo sapiens glypican 4 (GPC4), mRNA.

<u>Supplementary table 3</u>: Genes up regulated in Day 12 as compared to Day 0

Refseq_NM	Gene symbol	D12/D0.fc	DEFINITION
NM_014211.1	GABRP	115.164	Homo sapiens gamma-aminobutyric acid (GABA) A receptor, pi (GABRP), mRNA.
NM_024626.2	VTCN1	110.833	Homo sapiens V-set domain containing T cell activation inhibitor 1 (VTCN1), mRNA.
NM_004821.1	HAND1	109.556	Homo sapiens heart and neural crest derivatives expressed 1 (HAND1), mRNA.
NM_001430.3	EPAS1	92.690	Homo sapiens endothelial PAS domain protein 1 (EPAS1), mRNA.
NM_001042425.1	TFAP2A	80.495	Homo sapiens transcription factor AP-2 alpha (activating enhancer binding protein 2 alpha) (TFAP2A), transcript variant 3, mRNA.
NM_004613.2	TGM2	49.095	Homo sapiens transglutaminase 2 (C polypeptide, protein-glutamine-gamma-glutamyltransferase) (TGM2), transcript variant 1, mRNA.
NM_000358.1	TGFBI	48.979	Homo sapiens transforming growth factor, beta-induced, 68kDa (TGFBI), mRNA.
NR_002196.1	H19	48.264	Homo sapiens H19, imprinted maternally expressed transcript (non-protein coding) (H19), non-coding RNA.
NM_133505.2	DCN	47.699	Homo sapiens decorin (DCN), transcript variant C, mRNA.
NM_020130.3	C8orf4	42.822	Homo sapiens chromosome 8 open reading frame 4 (C8orf4), mRNA.
NM_000090.3	COL3A1	41.723	Homo sapiens collagen, type III, alpha 1 (COL3A1), mRNA.
NM_152321.1	ERP27	41.607	Homo sapiens endoplasmic reticulum protein 27 kDa (ERP27), mRNA.
NM_002345.3	LUM	41.260	Homo sapiens lumican (LUM), mRNA.
NM_004994.2	MMP9	40.432	Homo sapiens matrix metallopeptidase 9 (gelatinase B, 92kDa gelatinase, 92kDa type IV collagenase) (MMP9), mRNA.
NM_021005.2	NR2F2	36.073	Homo sapiens nuclear receptor subfamily 2, group F, member 2 (NR2F2), mRNA.
NM_184085.1	TRIM55	36.011	Homo sapiens tripartite motif-containing 55 (TRIM55), transcript variant 1, mRNA.
NM_181712.4	KANK4	35.816	Homo sapiens KN motif and ankyrin repeat domains 4 (KANK4), mRNA.
NM_181712.3	ANKRD38	34.705	Homo sapiens ankyrin repeat domain 38 (ANKRD38), mRNA.
NM_000598.4	IGFBP3	34.232	Homo sapiens insulin-like growth factor binding protein 3 (IGFBP3), transcript variant 2, mRNA.
NM_184086.1	TRIM55	34.125	Homo sapiens tripartite motif-containing 55 (TRIM55), transcript variant 3, mRNA.
NM_001013398.1	IGFBP3	33.884	Homo sapiens insulin-like growth factor binding protein 3 (IGFBP3), transcript variant 1, mRNA.
NM_182920.1	ADAMTS9	30.342	Homo sapiens ADAM metallopeptidase with thrombospondin type 1 motif, 9 (ADAMTS9), mRNA.
NM_052947.3	ALPK2	28.891	Homo sapiens alpha-kinase 2 (ALPK2), mRNA.
NM_002160.2	TNC	28.417	Homo sapiens tenascin C (TNC), mRNA.
NM_172315.1	MEIS2	28.177	Homo sapiens Meis homeobox 2 (MEIS2), transcript variant g, mRNA.
NM_000599.2	IGFBP5	26.499	Homo sapiens insulin-like growth factor binding protein 5 (IGFBP5), mRNA.
NM_032413.2	C15orf48	25.052	Homo sapiens chromosome 15 open reading frame 48 (C15orf48), transcript variant 2, mRNA.
NM_003068.3	SNAI2	24.724	Homo sapiens snail homolog 2 (Drosophila) (SNAI2), mRNA.
NM_001613.1	ACTA2	24.673	Homo sapiens actin, alpha 2, smooth muscle, aorta (ACTA2), mRNA.
NM_173054.1	RELN	24.614	Homo sapiens reelin (RELN), transcript variant 2, mRNA.
NM_000093.3	COL5A1	24.434	Homo sapiens collagen, type V, alpha 1 (COL5A1), mRNA.

NM_000599.2	IGFBP5	23.569	Homo sapiens insulin-like growth factor binding protein 5 (IGFBP5), mRNA.
NM_005630.1	SLCO2A1	23.482	Homo sapiens solute carrier organic anion transporter family, member 2A1 (SLCO2A1), mRNA.
NM_002145.3	HOXB2	23.281	Homo sapiens homeobox B2 (HOXB2), mRNA.
NM_057165.2	COL6A3	22.949	Homo sapiens collagen, type VI, alpha 3 (COL6A3), transcript variant 3, mRNA.
NM_001032280.2	TFAP2A	22.840	Homo sapiens transcription factor AP-2 alpha (activating enhancer binding protein 2 alpha) (TFAP2A), transcript variant 2, mRNA.
NM_001175.4	ARHGDIB	21.512	Homo sapiens Rho GDP dissociation inhibitor (GDI) beta (ARHGDIB), mRNA.
NM_001553.1	IGFBP7	20.972	Homo sapiens insulin-like growth factor binding protein 7 (IGFBP7), mRNA.
NM_133265.2	AMOT	19.936	Homo sapiens angiomotin (AMOT), transcript variant 2, mRNA.
NM_002653.3	PITX1	19.816	Homo sapiens paired-like homeodomain transcription factor 1 (PITX1), mRNA.
NM_002353.1	TACSTD2	19.585	Homo sapiens tumor-associated calcium signal transducer 2 (TACSTD2), mRNA.
NM_133337.1	FER1L3	19.558	Homo sapiens fer-1-like 3, myoferlin (C. elegans) (FER1L3), transcript variant 2, mRNA.
NM_013451.3	MYOF	18.731	Homo sapiens myoferlin (MYOF), transcript variant 1, mRNA.
NM_002202.1	ISL1	18.701	Homo sapiens ISL1 transcription factor, LIM/homeodomain, (islet-1) (ISL1), mRNA.
NM_001884.2	HAPLN1	18.646	Homo sapiens hyaluronan and proteoglycan link protein 1 (HAPLN1), mRNA.
NM_004369.2	COL6A3	18.540	Homo sapiens collagen, type VI, alpha 3 (COL6A3), transcript variant 1, mRNA.
NM_033255.2	EPSTI1	17.474	Homo sapiens epithelial stromal interaction 1 (breast) (EPSTI1), transcript variant 2, mRNA.
NM_024911.4	GPR177	17.212	Homo sapiens G protein-coupled receptor 177 (GPR177), transcript variant 1, mRNA.
NM_003063.2	SLN	16.983	Homo sapiens sarcolipin (SLN), mRNA.
NM_198552.1	FAM89A	16.478	Homo sapiens family with sequence similarity 89, member A (FAM89A), mRNA.
NM_014899.3	RHOBTB3	16.443	Homo sapiens Rho-related BTB domain containing 3 (RHOBTB3), mRNA.
NM_005613.3	RGS4	15.940	Homo sapiens regulator of G-protein signalling 4 (RGS4), mRNA.
NM_005161.3	APLNR	15.307	Homo sapiens apelin receptor (APLNR), mRNA.
NM_025140.1	CCDC92	15.136	Homo sapiens coiled-coil domain containing 92 (CCDC92), mRNA.
NM_000963.1	PTGS2	14.812	Homo sapiens prostaglandin-endoperoxide synthase 2 (prostaglandin G/H synthase and cyclooxygenase) (PTGS2), mRNA.
NM_001001936.1	AFAP1L2	14.713	Homo sapiens actin filament associated protein 1-like 2 (AFAP1L2), transcript variant 1, mRNA.
NM_016267.2	VGLL1	14.674	Homo sapiens vestigial like 1 (Drosophila) (VGLL1), mRNA.
NM_006727.2	CDH10	14.299	Homo sapiens cadherin 10, type 2 (T2-cadherin) (CDH10), mRNA.
NM_002521.2	NPPB	13.860	Homo sapiens natriuretic peptide precursor B (NPPB), mRNA.
NM_000862.2	HSD3B1	13.575	Homo sapiens hydroxy-delta-5-steroid dehydrogenase, 3 beta- and steroid delta-isomerase 1 (HSD3B1), mRNA.
NM_006472.2	TXNIP	13.544	Homo sapiens thioredoxin interacting protein (TXNIP), mRNA.
NM_003062.1	SLIT3	13.255	Homo sapiens slit homolog 3 (Drosophila) (SLIT3), mRNA.
NM_153321.1	PMP22	13.191	Homo sapiens peripheral myelin protein 22 (PMP22), transcript variant 2, mRNA.

NM_007173.4	PRSS23	13.124	Homo sapiens protease, serine, 23 (PRSS23), mRNA.
NM_014585.3	SLC40A1	12.967	Homo sapiens solute carrier family 40 (iron-regulated transporter), member 1 (SLC40A1), mRNA.
NM_024430.2	PSTPIP2	12.782	Homo sapiens proline-serine-threonine phosphatase interacting protein 2 (PSTPIP2), mRNA.
NM_002448.3	MSX1	12.692	Homo sapiens msh homeobox 1 (MSX1), mRNA.
NM_001002292.1	GPR177	12.622	Homo sapiens G protein-coupled receptor 177 (GPR177), transcript variant 2, mRNA.
NM_002341.1	LTB	12.520	Homo sapiens lymphotoxin beta (TNF superfamily, member 3) (LTB), transcript variant 1, mRNA.
NM_004207.2	SLC16A3	12.485	Homo sapiens solute carrier family 16, member 3 (monocarboxylic acid transporter 4) (SLC16A3), transcript variant 2, mRNA.
NM_016613.4	C4orf18	12.481	Homo sapiens chromosome 4 open reading frame 18 (C4orf18), transcript variant 2, mRNA.
NM_016613.5	C4orf18	12.266	Homo sapiens chromosome 4 open reading frame 18 (C4orf18), transcript variant 2, mRNA.
NM_201525.1	GPR56	12.184	Homo sapiens G protein-coupled receptor 56 (GPR56), transcript variant 3, mRNA.
NM_001124758.1	SPNS2	12.150	Homo sapiens spinster homolog 2 (Drosophila) (SPNS2), mRNA.
NM_001002292.1	GPR177	12.060	Homo sapiens G protein-coupled receptor 177 (GPR177), transcript variant 2, mRNA.
NM_000856.3	GUCY1A3	12.022	Homo sapiens guanylate cyclase 1, soluble, alpha 3 (GUCY1A3), mRNA.
NM_198552.1	FAM89A	12.002	Homo sapiens family with sequence similarity 89, member A (FAM89A), mRNA.
NM_206966.2	C5orf46	11.944	Homo sapiens chromosome 5 open reading frame 46 (C5orf46), mRNA.
NM_013451.2	FER1L3	11.817	Homo sapiens fer-1-like 3, myoferlin (C. elegans) (FER1L3), transcript variant 1, mRNA.
NM_003873.4	NRP1	11.583	Homo sapiens neuropilin 1 (NRP1), transcript variant 1, mRNA.
NM_000089.3	COL1A2	11.566	Homo sapiens collagen, type I, alpha 2 (COL1A2), mRNA.
NM_004428.2	EFNA1	11.539	Homo sapiens ephrin-A1 (EFNA1), transcript variant 1, mRNA.
NM_000389.2	CDKN1A	11.406	Homo sapiens cyclin-dependent kinase inhibitor 1A (p21, Cip1) (CDKN1A), transcript variant 1, mRNA.
NM_000089.3	COL1A2	11.346	Homo sapiens collagen, type I, alpha 2 (COL1A2), mRNA.
NM_001323.2	CST6	11.246	Homo sapiens cystatin E/M (CST6), mRNA.
NM_001620.1	AHNAK	11.245	Homo sapiens AHNAK nucleoprotein (AHNAK), transcript variant 1, mRNA.
NM_001037582.1	SCD5	11.134	Homo sapiens stearoyl-CoA desaturase 5 (SCD5), transcript variant 1, mRNA.
NM_015419.2	MXRA5	11.052	Homo sapiens matrix-remodelling associated 5 (MXRA5), mRNA.
NM_153366.2	SVEP1	11.012	Homo sapiens sushi, von Willebrand factor type A, EGF and pentraxin domain containing 1 (SVEP1), mRNA.
NM_152611.2	C20orf75	11.004	Homo sapiens chromosome 20 open reading frame 75 (C20orf75), mRNA.
NM_203411.1	TMEM88	10.850	Homo sapiens transmembrane protein 88 (TMEM88), mRNA.
NM_000362.4	TIMP3	10.718	Homo sapiens TIMP metallopeptidase inhibitor 3 (TIMP3), mRNA.
NM_032550.2	AFAP1L2	10.699	Homo sapiens actin filament associated protein 1-like 2 (AFAP1L2), transcript variant 2, mRNA.
NM_002543.3	OLR1	10.599	Homo sapiens oxidized low density lipoprotein (lectin-like) receptor 1 (OLR1), mRNA.
NM_003068.3	SNAI2	10.534	Homo sapiens snail homolog 2 (Drosophila) (SNAI2), mRNA.

NM_001453.1	FOXC1	10.430	Homo sapiens forkhead box C1 (FOXC1), mRNA.
NM_198951.1	TGM2	10.110	Homo sapiens transglutaminase 2 (C polypeptide, protein-glutamine-gamma-glutamyltransferase) (TGM2), transcript variant 2, mRNA.
NM_001040708.1	HEY1	10.051	Homo sapiens hairy/enhancer-of-split related with YRPW motif 1 (HEY1), transcript variant 2, mRNA.
XM_939093.1	FAM89A	10.020	PREDICTED: Homo sapiens family with sequence similarity 89, member A (FAM89A), mRNA.
AK025332		9.802	Homo sapiens cDNA: FLJ21679 fis, clone COL09221
NM_000316.2	PTH1R	9.741	Homo sapiens parathyroid hormone 1 receptor (PTH1R), mRNA.
NM_001002292.1	GPR177	9.718	Homo sapiens G protein-coupled receptor 177 (GPR177), transcript variant 2, mRNA.
NM_031910.3	C1QTNF6	9.698	Homo sapiens C1q and tumor necrosis factor related protein 6 (C1QTNF6), transcript variant 1, mRNA.
NM_201524.1	GPR56	9.697	Homo sapiens G protein-coupled receptor 56 (GPR56), transcript variant 2, mRNA.
NM_021205.4	RHOU	9.667	Homo sapiens ras homolog gene family, member U (RHOU), mRNA.
NM_000700.1	ANXA1	9.633	Homo sapiens annexin A1 (ANXA1), mRNA.
NM_003979.3	GPRC5A	9.539	Homo sapiens G protein-coupled receptor, family C, group 5, member A (GPRC5A), mRNA.
NM_003670.1	BHLHB2	9.463	Homo sapiens basic helix-loop-helix domain containing, class B, 2 (BHLHB2), mRNA.
NM_025202.2	EFHD1	9.442	Homo sapiens EF-hand domain family, member D1 (EFHD1), mRNA.
NM_019058.2	DDIT4	9.425	Homo sapiens DNA-damage-inducible transcript 4 (DDIT4), mRNA.
NM_001977.3	ENPEP	9.397	Homo sapiens glutamyl aminopeptidase (aminopeptidase A) (ENPEP), mRNA.
NM_002476.2	MYL4	9.308	Homo sapiens myosin, light chain 4, alkali; atrial, embryonic (MYL4), transcript variant 2, mRNA.
NM_133468.3	BMPER	9.227	Homo sapiens BMP binding endothelial regulator (BMPER), mRNA.
NM_001421.2	ELF4	9.200	Homo sapiens E74-like factor 4 (ets domain transcription factor) (ELF4), mRNA.
NM_032873.3	STS-1	9.129	Homo sapiens Cbl-interacting protein Sts-1 (STS-1), mRNA.
NM_004120.3	GBP2	9.106	Homo sapiens guanylate binding protein 2, interferon-inducible (GBP2), mRNA.
NM_001630.1	ANXA8	9.109	Homo sapiens annexin A8 (ANXA8), mRNA.
NM_006751.4	SSFA2	9.062	Homo sapiens sperm specific antigen 2 (SSFA2), mRNA.
NM_024519.2	FAM65A	9.026	Homo sapiens family with sequence similarity 65, member A (FAM65A), mRNA.
XM_926584.1	LOC653110	8.534	PREDICTED: Homo sapiens similar to annexin A8, transcript variant 1 (LOC653110), mRNA.
NM_001884.2	HAPLN1	8.513	Homo sapiens hyaluronan and proteoglycan link protein 1 (HAPLN1), mRNA.
NM_006255.3	PRKCH	8.506	Homo sapiens protein kinase C, eta (PRKCH), mRNA.
NM_005045.2	RELN	8.430	Homo sapiens reelin (RELN), transcript variant 1, mRNA.
NM_000905.2	NPY	8.428	Homo sapiens neuropeptide Y (NPY), mRNA.
NM_007088.2	CALB2	8.413	Homo sapiens calbindin 2 (CALB2), transcript variant CALB2c, mRNA.
NM_001114981.1	TP63	8.407	Homo sapiens tumor protein p63 (TP63), transcript variant 5, mRNA.
XR_038625.1	LOC100132535	8.402	PREDICTED: Homo sapiens misc_RNA (LOC100132535), miscRNA.
NM_001797.2	CDH11	8.369	Homo sapiens cadherin 11, type 2, OB-cadherin (osteoblast) (CDH11), mRNA.
XM_001126087.1	LOC728473	8.281	PREDICTED: Homo sapiens hypothetical LOC728473 (LOC728473), mRNA.

NM_001007023.2	DIO2	7.822	Homo sapiens deiodinase, iodothyronine, type II (DIO2), transcript variant 3, mRNA.
XM_001126471.1	LOC730278	7.793	PREDICTED: Homo sapiens hypothetical LOC730278 (LOC730278), mRNA.
NM_001007097.1	NTRK2	7.692	Homo sapiens neurotrophic tyrosine kinase, receptor, type 2 (NTRK2), transcript variant b, mRNA.
NM_001007139.3	IGF2	7.668	Homo sapiens insulin-like growth factor 2 (somatomedin A) (IGF2), transcript variant 2, mRNA.
NM_172112.1	EYA2	7.621	Homo sapiens eyes absent homolog 2 (Drosophila) (EYA2), transcript variant 4, mRNA.
NM_013231.4	FLRT2	7.579	Homo sapiens fibronectin leucine rich transmembrane protein 2 (FLRT2), mRNA.
NM_014890.2	FILIP1L	7.575	Homo sapiens filamin A interacting protein 1-like (FILIP1L), transcript variant 2, mRNA.
NM_198391.1	FLRT3	7.526	Homo sapiens fibronectin leucine rich transmembrane protein 3 (FLRT3), transcript variant 2, mRNA.
NM_014624.3	S100A6	7.484	Homo sapiens S100 calcium binding protein A6 (S100A6), mRNA.
NM_002982.3	CCL2	7.412	Homo sapiens chemokine (C-C motif) ligand 2 (CCL2), mRNA.
NM_002345.3	LUM	7.399	Homo sapiens lumican (LUM), mRNA.
NM_002449.4	MSX2	7.356	Homo sapiens msh homeobox 2 (MSX2), mRNA.
NM_002247.2	KCNMA1	7.329	Homo sapiens potassium large conductance calcium-activated channel, subfamily M, alpha member 1 (KCNMA1), transcript variant 2, mRNA.
NM_002160.1	TNC	7.322	Homo sapiens tenascin C (hexabrachion) (TNC), mRNA.
NM_199355.2	ADAMTS18	7.315	Homo sapiens ADAM metallopeptidase with thrombospondin type 1 motif, 18 (ADAMTS18), mRNA.
NM_018993.2	RIN2	7.298	Homo sapiens Ras and Rab interactor 2 (RIN2), mRNA.
NM_003966.2	SEMA5A	7.298	Homo sapiens sema domain, seven thrombospondin repeats (type 1 and type 1-like), transmembrane domain (TM) and short cytoplasmic domain, (semaphorin) 5A (SEMA5A), mRNA.
NM_005620.1	S100A11	7.275	Homo sapiens S100 calcium binding protein A11 (S100A11), mRNA.
NM_002404.1	MFAP4	7.268	Homo sapiens microfibrillar-associated protein 4 (MFAP4), mRNA.
NM_006283.1	TACC1	7.247	Homo sapiens transforming, acidic coiled-coil containing protein 1 (TACC1), mRNA.
NM_024911.4	GPR177	7.197	Homo sapiens G protein-coupled receptor 177 (GPR177), transcript variant 1, mRNA.
NM_024574.3	C4orf31	7.172	Homo sapiens chromosome 4 open reading frame 31 (C4orf31), mRNA.
NM_002203.3	ITGA2	7.176	Homo sapiens integrin, alpha 2 (CD49B, alpha 2 subunit of VLA-2 receptor) (ITGA2), mRNA.
NM_001955.2	EDN1	7.147	Homo sapiens endothelin 1 (EDN1), mRNA.
NM_002658.2	PLAU	7.076	Homo sapiens plasminogen activator, urokinase (PLAU), mRNA.
NM_006885.3	ZFHX3	7.041	Homo sapiens zinc finger homeobox 3 (ZFHX3), transcript variant A, mRNA.
NM_003392.3	WNT5A	6.981	Homo sapiens wingless-type MMTV integration site family, member 5A (WNT5A), mRNA.
NM_018670.2	MESP1	6.962	Homo sapiens mesoderm posterior 1 homolog (mouse) (MESP1), mRNA.
NM_005195.3	CEBPD	6.945	Homo sapiens CCAAT/enhancer binding protein (C/EBP), delta (CEBPD), mRNA.
NM_002026.2	FN1	6.883	Homo sapiens fibronectin 1 (FN1), transcript variant 3, mRNA.
NM_003619.2	PRSS12	6.829	Homo sapiens protease, serine, 12 (neurotrypsin, motopsin) (PRSS12), mRNA.

NM_176796.1	P2RY6	6.777	Homo sapiens pyrimidinergic receptor P2Y, G-protein coupled, 6 (P2RY6), transcript variant 3, mRNA.
NM_152753.2	SCUBE3	6.774	Homo sapiens signal peptide, CUB domain, EGF-like 3 (SCUBE3), mRNA.
NM_001017973.1	P4HA2	6.745	Homo sapiens prolyl 4-hydroxylase, alpha polypeptide II (P4HA2), transcript variant 2, mRNA.
NM_001717.2	BNC1	6.741	Homo sapiens basonuclin 1 (BNC1), mRNA.
NM_182801.1	EGFLAM	6.740	Homo sapiens EGF-like, fibronectin type III and laminin G domains (EGFLAM), transcript variant 4, mRNA.
NM_139212.2	HOPX	6.730	Homo sapiens HOP homeobox (HOPX), transcript variant 3, mRNA.
NM_032772.3	ZNF503	6.718	Homo sapiens zinc finger protein 503 (ZNF503), mRNA.
NM_078487.2	CDKN2B	6.706	Homo sapiens cyclin-dependent kinase inhibitor 2B (p15, inhibits CDK4) (CDKN2B), transcript variant 2, mRNA.
NM_021965.3	PGM5	6.651	Homo sapiens phosphoglucomutase 5 (PGM5), mRNA.
NM_000612.2	IGF2	6.641	Homo sapiens insulin-like growth factor 2 (somatomedin A) (IGF2), mRNA.
NM_022873.2	IFI6	6.635	Homo sapiens interferon, alpha-inducible protein 6 (IFI6), transcript variant 3, mRNA.
NM_020311.2	CXCR7	6.632	Homo sapiens chemokine (C-X-C motif) receptor 7 (CXCR7), mRNA.
NM_002960.1	S100A3	6.609	Homo sapiens S100 calcium binding protein A3 (S100A3), mRNA.
NM_018584.5	CAMK2N1	6.598	Homo sapiens calcium/calmodulin-dependent protein kinase II inhibitor 1 (CAMK2N1), mRNA.
NM_080645.2	COL12A1	6.585	Homo sapiens collagen, type XII, alpha 1 (COL12A1), transcript variant short, mRNA.
NM_012342.2	BAMBI	6.437	Homo sapiens BMP and activin membrane-bound inhibitor homolog (Xenopus laevis) (BAMBI), mRNA.
NM_001257.3	CDH13	6.423	Homo sapiens cadherin 13, H-cadherin (heart) (CDH13), mRNA.
NM_004878.3	PTGES	6.375	Homo sapiens prostaglandin E synthase (PTGES), mRNA.
NM_002398.2	MEIS1	6.309	Homo sapiens Meis homeobox 1 (MEIS1), mRNA.
NM_021965.3	PGM5	6.286	Homo sapiens phosphoglucomutase 5 (PGM5), mRNA.
NM_004428.2	EFNA1	6.277	Homo sapiens ephrin-A1 (EFNA1), transcript variant 1, mRNA.
NM_000088.3	COL1A1	6.272	Homo sapiens collagen, type I, alpha 1 (COL1A1), mRNA.
XR_016703.1	LOC644743	6.269	PREDICTED: Homo sapiens hypothetical LOC644743 (LOC644743), mRNA.
NM_001717.2	BNC1	6.268	Homo sapiens basonuclin 1 (BNC1), mRNA.
NM_000735.2	CGA	6.263	Homo sapiens glycoprotein hormones, alpha polypeptide (CGA), mRNA.
NM_000735.2	CGA	6.263	Homo sapiens glycoprotein hormones, alpha polypeptide (CGA), mRNA.
NM_001024912.1	CEACAM1	6.237	Homo sapiens carcinoembryonic antigen-related cell adhesion molecule 1 (biliary glycoprotein) (CEACAM1), transcript variant 2, mRNA.
NM_031476.2	CRISPLD2	6.232	Homo sapiens cysteine-rich secretory protein LCCL domain containing 2 (CRISPLD2), mRNA.
NM_002317.3	LOX	6.193	Homo sapiens lysyl oxidase (LOX), mRNA.
NM_001012964.1	KLK6	6.191	Homo sapiens kallikrein-related peptidase 6 (KLK6), transcript variant B, mRNA.
NM_001036.2	RYR3	6.169	Homo sapiens ryanodine receptor 3 (RYR3), mRNA.
NM_015691.2	WWC3	6.162	Homo sapiens WWC family member 3 (WWC3), mRNA.
NM_001423.1	EMP1	6.118	Homo sapiens epithelial membrane protein 1 (EMP1), mRNA.

NM_002609.3	PDGFRB	6.116	Homo sapiens platelet-derived growth factor receptor, beta polypeptide (PDGFRB), mRNA.
NM_178565.3	RSPO2	6.015	Homo sapiens R-spondin 2 homolog (Xenopus laevis) (RSPO2), mRNA.
NM_004093.2	EFNB2	6.013	Homo sapiens ephrin-B2 (EFNB2), mRNA.
NM_005562.1	LAMC2	5.976	Homo sapiens laminin, gamma 2 (LAMC2), transcript variant 1, mRNA.
XR_018676.1	LOC647954	5.972	PREDICTED: Homo sapiens misc_RNA (LOC647954), miscRNA.
NM_000393.3	COL5A2	5.955	Homo sapiens collagen, type V, alpha 2 (COL5A2), mRNA.
NM_015036.1	ENDOD1	5.933	Homo sapiens endonuclease domain containing 1 (ENDOD1), mRNA.
NM_000494.3	COL17A1	5.916	Homo sapiens collagen, type XVII, alpha 1 (COL17A1), mRNA.
NM_022131.1	CLSTN2	5.882	Homo sapiens calsyntenin 2 (CLSTN2), mRNA.
NM_000888.3	ITGB6	5.855	Homo sapiens integrin, beta 6 (ITGB6), mRNA.
NM_182487.2	OLFML2A	5.874	Homo sapiens olfactomedin-like 2A (OLFML2A), mRNA.
NM_145057.2	CDC42EP5	5.839	Homo sapiens CDC42 effector protein (Rho GTPase binding) 5 (CDC42EP5), mRNA.
NM_014333.3	CADM1	5.788	Homo sapiens cell adhesion molecule 1 (CADM1), transcript variant 1, mRNA.
NM_006287.4	TFPI	5.771	Homo sapiens tissue factor pathway inhibitor (lipoprotein-associated coagulation inhibitor) (TFPI), transcript variant 1, mRNA.
NM_005512.1	LRRC32	5.746	Homo sapiens leucine rich repeat containing 32 (LRRC32), mRNA.
NM_016205.1	PDGFC	5.718	Homo sapiens platelet derived growth factor C (PDGFC), mRNA.
NM_005996.3	TBX3	5.693	Homo sapiens T-box 3 (TBX3), transcript variant 1, mRNA.
NM_002147.3	HOXB5	5.686	Homo sapiens homeobox B5 (HOXB5), mRNA.
NM_207304.1	MBNL2	5.680	Homo sapiens muscleblind-like 2 (Drosophila) (MBNL2), transcript variant 3, mRNA.
NM_003238.1	TGFB2	5.668	Homo sapiens transforming growth factor, beta 2 (TGFB2), mRNA.
NM_002048.1	GAS1	5.614	Homo sapiens growth arrest-specific 1 (GAS1), mRNA.
NM_006851.2	GLIPR1	5.597	Homo sapiens GLI pathogenesis-related 1 (GLIPR1), mRNA.
NM_006142.3	SFN	5.592	Homo sapiens stratifin (SFN), mRNA.
NM_006287.4	TFPI	5.578	Homo sapiens tissue factor pathway inhibitor (lipoprotein-associated coagulation inhibitor) (TFPI), transcript variant 1, mRNA.
NM_001047841.1	CXCR7	5.568	Homo sapiens chemokine (C-X-C motif) receptor 7 (CXCR7), transcript variant 1, mRNA.
NM_000756.1	CRH	5.556	Homo sapiens corticotropin releasing hormone (CRH), mRNA.
NM_001630.2	ANXA8L2	5.551	Homo sapiens annexin A8-like 2 (ANXA8L2), mRNA.
NM_001001391.1	CD44	5.528	Homo sapiens CD44 molecule (Indian blood group) (CD44), transcript variant 4, mRNA.
NM_181873.2	MTMR11	5.498	Homo sapiens myotubularin related protein 11 (MTMR11), mRNA.
NM_001458.3	FLNC	5.488	Homo sapiens filamin C, gamma (actin binding protein 280) (FLNC), mRNA.
NM_022164.1	TINAGL1	5.479	Homo sapiens tubulointerstitial nephritis antigen-like 1 (TINAGL1), mRNA.
NM_006487.2	FBLN1	5.455	Homo sapiens fibulin 1 (FBLN1), transcript variant A, mRNA.
NM_002205.2	ITGA5	5.435	Homo sapiens integrin, alpha 5 (fibronectin receptor, alpha polypeptide) (ITGA5), mRNA.
NM_024913.3	FLJ21986	5.432	Homo sapiens hypothetical protein FLJ21986 (FLJ21986), mRNA.
NM_031439.2	SOX7	5.426	Homo sapiens SRY (sex determining region Y)-box 7 (SOX7), mRNA.
NM_000039.1	APOA1	5.425	Homo sapiens apolipoprotein A-I (APOA1), mRNA.

NM_005221.5	DLX5	5.424	Homo sapiens distal-less homeobox 5 (DLX5), mRNA.
NM_024841.3	FLJ14213	5.424	Homo sapiens protor-2 (FLJ14213), mRNA.
NM_004154.3	P2RY6	5.394	Homo sapiens pyrimidinergic receptor P2Y, G-protein coupled, 6 (P2RY6), transcript variant 4, mRNA.
NM_005228.3	EGFR	5.382	Homo sapiens epidermal growth factor receptor (erythroblastic leukemia viral (v-erb-b) oncogene homolog, avian) (EGFR), transcript variant 1, mRNA.
NM_002414.3	CD99	5.382	Homo sapiens CD99 molecule (CD99), transcript variant 1, mRNA.
NM_006129.2	BMP1	5.346	Homo sapiens bone morphogenetic protein 1 (BMP1), transcript variant BMP1-3, mRNA.
NM_005141.2	FGB	5.340	Homo sapiens fibrinogen beta chain (FGB), mRNA.
NM_030665.3	RAI1	5.331	Homo sapiens retinoic acid induced 1 (RAI1), mRNA.
NM_002581.3	PAPPA	5.331	Homo sapiens pregnancy-associated plasma protein A, pappalysin 1 (PAPPA), mRNA.
NM_002845.2	PTPRM	5.323	Homo sapiens protein tyrosine phosphatase, receptor type, M (PTPRM), mRNA.
NM_018469.3	TEX2	5.318	Homo sapiens testis expressed 2 (TEX2), mRNA.
NM_006203.3	PDE4D	5.315	Homo sapiens phosphodiesterase 4D, cAMP-specific (phosphodiesterase E3 dunce homolog, Drosophila) (PDE4D), mRNA.
XM_001723978.1	SPNS2	5.308	PREDICTED: Homo sapiens spinster homolog 2 (Drosophila) (SPNS2), mRNA.
NM_006290.2	TNFAIP3	5.299	Homo sapiens tumor necrosis factor, alpha-induced protein 3 (TNFAIP3), mRNA.
XM_001726959.1	KRT18P13	5.278	PREDICTED: Homo sapiens keratin 18 pseudogene 13 (KRT18P13), mRNA.
NM_001996.2	FBLN1	5.258	Homo sapiens fibulin 1 (FBLN1), transcript variant C, mRNA.
NM_182827.1	FKBP9L	5.248	Homo sapiens FK506 binding protein 9-like (FKBP9L), mRNA.
XM_001716667.1	LOC731932	5.220	PREDICTED: Homo sapiens hypothetical LOC731932 (LOC731932), mRNA.
NM_003722.3	TP73L	5.217	Homo sapiens tumor protein p73-like (TP73L), mRNA.
NM_003387.3	WASPIP	5.215	Homo sapiens Wiskott-Aldrich syndrome protein interacting protein (WASPIP), mRNA.
NM_032041.1	NCALD	5.211	Homo sapiens neurocalcin delta (NCALD), mRNA.
NM_178507.2	OAF	5.210	Homo sapiens OAF homolog (Drosophila) (OAF), mRNA.
NM_144729.1	DUSP10	5.183	Homo sapiens dual specificity phosphatase 10 (DUSP10), transcript variant 3, mRNA.
NM_080818.3	OXGR1	5.178	Homo sapiens oxoglutarate (alpha-ketoglutarate) receptor 1 (OXGR1), mRNA.
NM_005159.4	ACTC1	5.169	Homo sapiens actin, alpha, cardiac muscle 1 (ACTC1), mRNA.
NM_003118.2	SPARC	5.157	Homo sapiens secreted protein, acidic, cysteine-rich (osteonectin) (SPARC), mRNA.
NM_014391.2	ANKRD1	5.154	Homo sapiens ankyrin repeat domain 1 (cardiac muscle) (ANKRD1), mRNA.
NM_004067.2	CHN2	5.133	Homo sapiens chimerin (chimaerin) 2 (CHN2), transcript variant 2, mRNA.
NM_016269.2	LEF1	5.133	Homo sapiens lymphoid enhancer-binding factor 1 (LEF1), mRNA.
NM_006096.2	NDRG1	5.133	Homo sapiens N-myc downstream regulated gene 1 (NDRG1), mRNA.
NM_152330.2	FRMD6	5.130	Homo sapiens FERM domain containing 6 (FRMD6), mRNA.
NM_138455.2	CTHRC1	5.119	Homo sapiens collagen triple helix repeat containing 1 (CTHRC1), mRNA.
NM_018712.2	ELMOD1	5.114	Homo sapiens ELMO/CED-12 domain containing 1 (ELMOD1), mRNA.

NM_000891.2	KCNJ2	5.113	Homo sapiens potassium inwardly-rectifying channel, subfamily J, member 2 (KCNJ2), mRNA.
NM_174911.3	FAM84B	5.105	Homo sapiens family with sequence similarity 84, member B (FAM84B), mRNA.
NM_173624.1	FLJ40504	5.102	Homo sapiens hypothetical protein FLJ40504 (FLJ40504), mRNA.
NM_147780.2	CTSB	5.084	Homo sapiens cathepsin B (CTSB), transcript variant 2, mRNA.
NM_207361.4	FREM2	5.082	Homo sapiens FRAS1 related extracellular matrix protein 2 (FREM2), mRNA.
NM_007361.3	NID2	5.074	Homo sapiens nidogen 2 (osteonidogen) (NID2), mRNA.
NM_001684.3	ATP2B4	5.073	Homo sapiens ATPase, Ca++ transporting, plasma membrane 4 (ATP2B4), transcript variant 2, mRNA.
NM_004864.1	GDF15	5.069	Homo sapiens growth differentiation factor 15 (GDF15), mRNA.
NM_018110.2	DOK4	5.058	Homo sapiens docking protein 4 (DOK4), mRNA.
NM_004936.3	CDKN2B	5.048	Homo sapiens cyclin-dependent kinase inhibitor 2B (p15, inhibits CDK4) (CDKN2B), transcript variant 1, mRNA.
NM_001003396.1	TPD52L1	5.045	Homo sapiens tumor protein D52-like 1 (TPD52L1), transcript variant 3, mRNA.
NM_016269.2	LEF1	5.042	Homo sapiens lymphoid enhancer-binding factor 1 (LEF1), mRNA.
NM_005097.1	LGI1	5.037	Homo sapiens leucine-rich, glioma inactivated 1 (LGI1), mRNA.
XR_017100.2	LOC149501	5.036	PREDICTED: Homo sapiens misc_RNA (LOC149501), miscRNA.
XR_017543.1	LOC400578	5.035	PREDICTED: Homo sapiens similar to Keratin, type I cytoskeletal 14 (Cytokeratin-14) (CK-14) (Keratin-14) (K14) (LOC400578), mRNA.
NM_130851.1	BMP4	5.034	Homo sapiens bone morphogenetic protein 4 (BMP4), transcript variant 3, mRNA.
NM_001032394.1	GPR126	5.030	Homo sapiens G protein-coupled receptor 126 (GPR126), transcript variant a2, mRNA.
NM_004530.2	MMP2	5.025	Homo sapiens matrix metallopeptidase 2 (gelatinase A, 72kDa gelatinase, 72kDa type IV collagenase) (MMP2), mRNA.
NM_003633.1	ENC1	5.016	Homo sapiens ectodermal-neural cortex (with BTB-like domain) (ENC1), mRNA.
NM_014365.2	HSPB8	5.005	Homo sapiens heat shock 22kDa protein 8 (HSPB8), mRNA.
NM_019605.2	SERTAD4	4.996	Homo sapiens SERTA domain containing 4 (SERTAD4), mRNA.
NM_032777.6	GPR124	4.989	Homo sapiens G protein-coupled receptor 124 (GPR124), mRNA.
NM_007112.3	THBS3	4.987	Homo sapiens thrombospondin 3 (THBS3), mRNA.
NM_033274.2	ADAM19	4.982	Homo sapiens ADAM metallopeptidase domain 19 (meltrin beta) (ADAM19), mRNA.
NM_001017974.1	P4HA2	4.974	Homo sapiens prolyl 4-hydroxylase, alpha polypeptide II (P4HA2), transcript variant 3, mRNA.
NM_001001396.1	ATP2B4	4.967	Homo sapiens ATPase, Ca++ transporting, plasma membrane 4 (ATP2B4), transcript variant 1, mRNA.
NM_000104.2	CYP1B1	4.965	Homo sapiens cytochrome P450, family 1, subfamily B, polypeptide 1 (CYP1B1), mRNA.
NM_014322.2	OPN3	4.925	Homo sapiens opsin 3 (OPN3), mRNA.
NM_001629.2	ALOX5AP	4.918	Homo sapiens arachidonate 5-lipoxygenase-activating protein (ALOX5AP), mRNA.
NM_005605.3	PPP3CC	4.901	Homo sapiens protein phosphatase 3 (formerly 2B), catalytic subunit, gamma isoform (PPP3CC), mRNA.
NM_000422.1	KRT17	4.895	Homo sapiens keratin 17 (KRT17), mRNA.
NM_014900.3	COBLL1	4.884	Homo sapiens COBL-like 1 (COBLL1), mRNA.
NM_000602.1	SERPINE1	4.881	Homo sapiens serpin peptidase inhibitor, clade E (nexin, plasminogen activator inhibitor type 1), member 1 (SERPINE1), mRNA.

XR_017689.1	KRT18P28	4.881	PREDICTED: Homo sapiens misc_RNA (KRT18P28), miscRNA.
NM_021170.2	HES4	4.856	Homo sapiens hairy and enhancer of split 4 (Drosophila) (HES4), mRNA.
NM_033504.2	TMEM54	4.853	Homo sapiens transmembrane protein 54 (TMEM54), mRNA.
NM_020877.2	DNAH2	4.836	Homo sapiens dynein, axonemal, heavy chain 2 (DNAH2), mRNA.
NM_031439.2	SOX7	4.835	Homo sapiens SRY (sex determining region Y)-box 7 (SOX7), mRNA.
NM_018012.2	KIF26B	4.833	Homo sapiens kinesin family member 26B (KIF26B), mRNA.
NM_017633.2	FAM46A	4.813	Homo sapiens family with sequence similarity 46, member A (FAM46A), mRNA.
NM_019885.2	CYP26B1	4.804	Homo sapiens cytochrome P450, family 26, subfamily B, polypeptide 1 (CYP26B1), mRNA.
NM_024767.2	DLC1	4.764	Homo sapiens deleted in liver cancer 1 (DLC1), transcript variant 3, mRNA.
NM_018890.2	RAC1	4.757	Homo sapiens ras-related C3 botulinum toxin substrate 1 (rho family, small GTP binding protein Rac1) (RAC1), transcript variant Rac1b, mRNA.
NM_001873.1	CPE	4.737	Homo sapiens carboxypeptidase E (CPE), mRNA.
BC062365		4.734	Homo sapiens cDNA clone IMAGE:5922621, partial cds
NM_002780.3	PSG4	4.720	Homo sapiens pregnancy specific beta-1-glycoprotein 4 (PSG4), transcript variant 1, mRNA.
NM_004525.2	LRP2	4.719	Homo sapiens low density lipoprotein-related protein 2 (LRP2), mRNA.
NM_005491.2	MAMLD1	4.714	Homo sapiens mastermind-like domain containing 1 (MAMLD1), mRNA.
NM_003480.2	MFAP5	4.704	Homo sapiens microfibrillar associated protein 5 (MFAP5), mRNA.
NM_005139.2	ANXA3	4.669	Homo sapiens annexin A3 (ANXA3), mRNA.
XR_017241.1	LOC646723	4.698	PREDICTED: Homo sapiens similar to Keratin, type I cytoskeletal 18 (Cytokeratin-18) (CK-18) (Keratin-18) (K18) (LOC646723), mRNA.
NM_138444.3	KCTD12	4.687	Homo sapiens potassium channel tetramerisation domain containing 12 (KCTD12), mRNA.
NM_001098816.2	ODZ4	4.681	Homo sapiens odz, odd Oz/ten-m homolog 4 (Drosophila) (ODZ4), mRNA.
NM_153427.1	PITX2	4.679	Homo sapiens paired-like homeodomain 2 (PITX2), transcript variant 1, mRNA.
NM_015130.2	TBC1D9	4.654	Homo sapiens TBC1 domain family, member 9 (with GRAM domain) (TBC1D9), mRNA.
NM_015245.2	ANKS1A	4.625	Homo sapiens ankyrin repeat and sterile alpha motif domain containing 1A (ANKS1A), mRNA.
NM_021073.2	BMP5	4.614	Homo sapiens bone morphogenetic protein 5 (BMP5), mRNA.
XR_015970.1	MGC102966	4.610	PREDICTED: Homo sapiens similar to Keratin, type I cytoskeletal 16 (Cytokeratin-16) (CK-16) (Keratin-16) (K16) (MGC102966), misc RNA.
NM_004173.2	SLC7A4	4.602	Homo sapiens solute carrier family 7 (cationic amino acid transporter, y+ system), member 4 (SLC7A4), mRNA.
NM_012307.2	EPB41L3	4.601	Homo sapiens erythrocyte membrane protein band 4.1-like 3 (EPB41L3), mRNA.
NM_024702.2	ZNF750	4.579	Homo sapiens zinc finger protein 750 (ZNF750), mRNA.
NM_005211.2	CSF1R	4.570	Homo sapiens colony stimulating factor 1 receptor, formerly McDonough feline sarcoma viral (v-fms) oncogene homolog (CSF1R), mRNA.
XM_001724386.1	LOC100134265	4.564	PREDICTED: Homo sapiens similar to calbindin 2 full length protein (LOC100134265), mRNA.
NM_032181.1	TMEM166	4.559	Homo sapiens transmembrane protein 166 (TMEM166), mRNA.

XR_017231.2	KRT8P9	4.554	PREDICTED: Homo sapiens misc_RNA (KRT8P9), miscRNA.
NM_006403.2	NEDD9	4.552	Homo sapiens neural precursor cell expressed, developmentally down-regulated 9 (NEDD9), transcript variant 1, mRNA.
NM_177964.3	LYPD6B	4.546	Homo sapiens LY6/PLAUR domain containing 6B (LYPD6B), mRNA.
NM_152611.3	LRRN4	4.540	Homo sapiens leucine rich repeat neuronal 4 (LRRN4), mRNA.
NM_001099287.1	NIPAL4	4.530	Homo sapiens NIPA-like domain containing 4 (NIPAL4), mRNA.
NM_003380.2	VIM	4.529	Homo sapiens vimentin (VIM), mRNA.
NR_001562.1	ANXA2P1	4.524	Homo sapiens annexin A2 pseudogene 1 (ANXA2P1) on chromosome 4.
NM_006270.3	RRAS	4.518	Homo sapiens related RAS viral (r-ras) oncogene homolog (RRAS), mRNA.
NM_000474.3	TWIST1	4.510	Homo sapiens twist homolog 1 (Drosophila) (TWIST1), mRNA.
NM_001040619.1	ATF3	4.506	Homo sapiens activating transcription factor 3 (ATF3), transcript variant 4, mRNA.
NM_002781.2	PSG5	4.493	Homo sapiens pregnancy specific beta-1-glycoprotein 5 (PSG5), mRNA.
NM_001748.3	CAPN2	4.492	Homo sapiens calpain 2, (m/II) large subunit (CAPN2), mRNA.
NM_207336.1	ZNF467	4.488	Homo sapiens zinc finger protein 467 (ZNF467), mRNA.
NM_002966.1	S100A10	4.478	Homo sapiens S100 calcium binding protein A10 (annexin II ligand, calpactin I, light polypeptide (p11)) (S100A10), mRNA.
NM_022059.1	CXCL16	4.474	Homo sapiens chemokine (C-X-C motif) ligand 16 (CXCL16), mRNA.
NM_020387.2	RAB25	4.457	Homo sapiens RAB25, member RAS oncogene family (RAB25), mRNA.
NM_002051.2	GATA3	4.440	Homo sapiens GATA binding protein 3 (GATA3), transcript variant 2, mRNA.
NM_212474.1	FN1	4.435	Homo sapiens fibronectin 1 (FN1), transcript variant 6, mRNA.
NM_197941.2	ADAMTS6	4.420	Homo sapiens ADAM metallopeptidase with thrombospondin type 1 motif, 6 (ADAMTS6), mRNA.
XM_001128419.1	MGC16121	4.414	PREDICTED: Homo sapiens hypothetical protein MGC16121 (MGC16121), mRNA.
NM_032199.1	ARID5B	4.407	Homo sapiens AT rich interactive domain 5B (MRF1-like) (ARID5B), mRNA.
NM_001908.3	CTSB	4.406	Homo sapiens cathepsin B (CTSB), transcript variant 1, mRNA.
NM_002048.1	GAS1	4.400	Homo sapiens growth arrest-specific 1 (GAS1), mRNA.
NM_000679.3	ADRA1B	4.398	Homo sapiens adrenergic, alpha-1B-, receptor (ADRA1B), mRNA.
NM_001265.2	CDX2	4.384	Homo sapiens caudal type homeobox 2 (CDX2), mRNA.
NM_001008490.1	KLF6	4.384	Homo sapiens Kruppel-like factor 6 (KLF6), transcript variant 1, mRNA.
NM_001999.3	FBN2	4.382	Homo sapiens fibrillin 2 (FBN2), mRNA.
NM_001233.3	CAV2	4.370	Homo sapiens caveolin 2 (CAV2), transcript variant 1, mRNA.
NM_152330.3	FRMD6	4.368	Homo sapiens FERM domain containing 6 (FRMD6), transcript variant 2, mRNA.
NM_018689.1	KIAA1199	4.367	Homo sapiens KIAA1199 (KIAA1199), mRNA.
NM_001034841.2	ITPRIPL2	4.360	Homo sapiens inositol 1,4,5-triphosphate receptor interacting protein-like 2 (ITPRIPL2), mRNA.
XR_037953.1	KRT18P17	4.326	PREDICTED: Homo sapiens misc_RNA (KRT18P17), miscRNA.
NM_003174.3	SVIL	4.320	Homo sapiens supervillin (SVIL), transcript variant 1, mRNA.
NM_000963.1	PTGS2	4.317	Homo sapiens prostaglandin-endoperoxide synthase 2 (prostaglandin G/H synthase and cyclooxygenase) (PTGS2), mRNA.
NM_152680.1	TMEM154	4.314	Homo sapiens transmembrane protein 154 (TMEM154), mRNA.

NM_005556.3	KRT7	4.304	Homo sapiens keratin 7 (KRT7), mRNA.
NM_006528.2	TFPI2	4.291	Homo sapiens tissue factor pathway inhibitor 2 (TFPI2), mRNA.
NM_182909.2	FILIP1L	4.288	Homo sapiens filamin A interacting protein 1-like (FILIP1L), transcript variant 1, mRNA.
NM_001008844.1	DSP	4.283	Homo sapiens desmoplakin (DSP), transcript variant 2, mRNA.
NM_000099.2	CST3	4.278	Homo sapiens cystatin C (CST3), mRNA.
NM_177964.3	LYPD6B	4.262	Homo sapiens LY6/PLAUR domain containing 6B (LYPD6B), mRNA.
NM_005328.1	HAS2	4.253	Homo sapiens hyaluronan synthase 2 (HAS2), mRNA.
NM_080388.1	S100A16	4.240	Homo sapiens S100 calcium binding protein A16 (S100A16), mRNA.
NM_144729.1	DUSP10	4.232	Homo sapiens dual specificity phosphatase 10 (DUSP10), transcript variant 3, mRNA.
NM_032784.3	RSPO3	4.210	Homo sapiens R-spondin 3 homolog (Xenopus laevis) (RSPO3), mRNA.
AB074162		4.192	Homo sapiens primary neuroblastoma cDNA, clone:Nbla10527, full insert sequence
NM_001099743.1	GOLSYN	4.189	Homo sapiens Golgi-localized protein (GOLSYN), transcript variant 7, mRNA.
NM_022051.1	EGLN1	4.184	Homo sapiens egl nine homolog 1 (C. elegans) (EGLN1), mRNA.
NM_005585.3	SMAD6	4.181	Homo sapiens SMAD family member 6 (SMAD6), transcript variant 1, mRNA.
NM_004172.3	SLC1A3	4.177	Homo sapiens solute carrier family 1 (glial high affinity glutamate transporter), member 3 (SLC1A3), mRNA.
NM_001024074.1	HNMT	4.166	Homo sapiens histamine N-methyltransferase (HNMT), transcript variant 2, mRNA.
NM_032873.4	UBASH3B	4.162	Homo sapiens ubiquitin associated and SH3 domain containing, B (UBASH3B), mRNA.
NM_148957.2	TNFRSF19	4.156	Homo sapiens tumor necrosis factor receptor superfamily, member 19 (TNFRSF19), transcript variant 2, mRNA.
NM_015869.4	PPARG	4.155	Homo sapiens peroxisome proliferator-activated receptor gamma (PPARG), transcript variant 2, mRNA.
NM_005318.2	H1F0	4.154	Homo sapiens H1 histone family, member 0 (H1F0), mRNA.
NM_005461.3	MAFB	4.149	Homo sapiens v-maf musculoaponeurotic fibrosarcoma oncogene homolog B (avian) (MAFB), mRNA.
NM_001040058.1	SPP1	4.148	Homo sapiens secreted phosphoprotein 1 (SPP1), transcript variant 1, mRNA.
NM_004787.1	SLIT2	4.146	Homo sapiens slit homolog 2 (Drosophila) (SLIT2), mRNA.
NM_004364.2	CEBPA	4.145	Homo sapiens CCAAT/enhancer binding protein (C/EBP), alpha (CEBPA), mRNA.
NM_001542.2	IGSF3	4.138	Homo sapiens immunoglobulin superfamily, member 3 (IGSF3), transcript variant 1, mRNA.
NM_001002858.1	ANXA2	4.128	Homo sapiens annexin A2 (ANXA2), transcript variant 1, mRNA.
XM_001722168.1	LOC100128893	4.126	PREDICTED: Homo sapiens hypothetical protein LOC100128893 (LOC100128893), mRNA.
NM_030952.1	NUAK2	4.127	Homo sapiens NUAK family, SNF1-like kinase, 2 (NUAK2), mRNA.
NM_004884.3	IGDCC3	4.108	Homo sapiens immunoglobulin superfamily, DCC subclass, member 3 (IGDCC3), mRNA.
NM_000943.4	PPIC	4.102	Homo sapiens peptidylprolyl isomerase C (cyclophilin C) (PPIC), mRNA.
NM_021245.2	MYOZ1	4.099	Homo sapiens myozenin 1 (MYOZ1), mRNA.
NM_005797.2	MPZL2	4.097	Homo sapiens myelin protein zero-like 2 (MPZL2), transcript variant 1, mRNA.
NM_024935.2	KIAA1772	4.092	Homo sapiens KIAA1772 (KIAA1772), mRNA.

NR_029622.1	MIR205	4.054	Homo sapiens microRNA 205 (MIR205), microRNA.
NM_178497.2	C4orf26	4.052	Homo sapiens chromosome 4 open reading frame 26 (C4orf26), mRNA.
NM_003380.2	VIM	4.048	Homo sapiens vimentin (VIM), mRNA.
NM_025179.3	PLXNA2	4.043	Homo sapiens plexin A2 (PLXNA2), mRNA.
NR_024430.1	LOC399959	4.032	Homo sapiens hypothetical LOC399959 (LOC399959), non-coding RNA.

Supplementary table 4: **Genes up regulated in Day 0 as compared to Day 20**

Refseq_NM	GeneSymbol	D20/D12.fc	DEFINITION
NM_002522.2	NPTX1	-23.785456	Homo sapiens neuronal pentraxin I (NPTX1), mRNA.
NM_000840.2	GRM3	-12.592607	Homo sapiens glutamate receptor, metabotropic 3 (GRM3), mRNA.
NM_002164.3	INDO	-9.18744	Homo sapiens indoleamine-pyrrole 2,3 dioxygenase (INDO), mRNA.
NM_002164.4	IDO1	-8.705727	Homo sapiens indoleamine 2,3-dioxygenase 1 (IDO1), mRNA.
NM_022034.4	CUZD1	-8.256749	Homo sapiens CUB and zona pellucida-like domains 1 (CUZD1), mRNA.
AL833138		-8.009543	Homo sapiens mRNA; cDNA DKFZp313A1040 (from clone DKFZp313A1040)
NM_003706.1	PLA2G4C	-7.624982	Homo sapiens phospholipase A2, group IVC (cytosolic, calcium-independent) (PLA2G4C), mRNA.
NM_014279.4	OLFM1	-7.000224	Homo sapiens olfactomedin 1 (OLFM1), transcript variant 1, mRNA.
NM_152779.2	GLIPR1L1	-6.795108	Homo sapiens GLI pathogenesis-related 1 like 1 (GLIPR1L1), mRNA.
NM_022144.1	TNMD	-6.461491	Homo sapiens tenomodulin (TNMD), mRNA.
AL049310		-6.364673	Homo sapiens mRNA; cDNA DKFZp564B206 (from clone DKFZp564B206)
NM_198139.1	SEMG1	-6.140634	Homo sapiens semenogelin I (SEMG1), transcript variant 2, mRNA.
NM_005296.1	LPAR4	-6.126293	Homo sapiens lysophosphatidic acid receptor 4 (LPAR4), mRNA.
NM_007015.2	LECT1	-5.996149	Homo sapiens leukocyte cell derived chemotaxin 1 (LECT1), transcript variant 1, mRNA.
NM_138328.2	RHBDL3	-5.585974	Homo sapiens rhomboid, veinlet-like 3 (Drosophila) (RHBDL3), mRNA.
NM_005756.2	GPR64	-5.390976	Homo sapiens G protein-coupled receptor 64 (GPR64), transcript variant 4, mRNA.
NM_007015.2	LECT1	-5.387534	Homo sapiens leukocyte cell derived chemotaxin 1 (LECT1), transcript variant 1, mRNA.
NM_004744.3	LRAT	-5.037117	Homo sapiens lecithin retinol acyltransferase (phosphatidylcholine--retinol O-acyltransferase) (LRAT), mRNA.
NM_003459.4	SLC30A3	-4.943123	Homo sapiens solute carrier family 30 (zinc transporter), member 3 (SLC30A3), mRNA.
NM_002993.2	CXCL6	-4.874339	Homo sapiens chemokine (C-X-C motif) ligand 6 (granulocyte chemotactic protein 2) (CXCL6), mRNA.
NM_015715.3	PLA2G3	-4.846862	Homo sapiens phospholipase A2, group III (PLA2G3), mRNA.
NM_006334.3	OLFM1	-4.819602	Homo sapiens olfactomedin 1 (OLFM1), transcript variant 2, mRNA.
NM_001964.2	EGR1	-4.687462	Homo sapiens early growth response 1 (EGR1), mRNA.
NM_015184.3	PLCL2	-4.648604	Homo sapiens phospholipase C-like 2 (PLCL2), mRNA.
NM_013244.2	MGAT4C	-4.449936	Homo sapiens mannosyl (alpha-1,3-)-glycoprotein beta-1,4-N-acetylglucosaminyltransferase, isozyme C (putative) (MGAT4C), mRNA.
NM_002006.3	FGF2	-4.440724	Homo sapiens fibroblast growth factor 2 (basic) (FGF2), mRNA.
NM_172337.1	OTX2	-4.430574	Homo sapiens orthodenticle homeobox 2 (OTX2), transcript variant 2, mRNA.
NM_001217.3	CA11	-4.404762	Homo sapiens carbonic anhydrase XI (CA11), mRNA.
XR_039453.1	LOC646316	-4.401608	PREDICTED: Homo sapiens misc_RNA (LOC646316), miscRNA.
NM_003541.2	HIST1H4K	-4.399901	Homo sapiens histone cluster 1, H4k (HIST1H4K), mRNA.
NM_003524.2	HIST1H2BH	-4.354731	Homo sapiens histone cluster 1, H2bh (HIST1H2BH), mRNA.
NM_013243.2	SCG3	-4.341447	Homo sapiens secretogranin III (SCG3), mRNA.
NM_018228.1	C14orf115	-4.299288	Homo sapiens chromosome 14 open reading frame 115 (C14orf115), mRNA.
NM_032446.1	MEGF10	-4.280219	Homo sapiens multiple EGF-like-domains 10 (MEGF10), mRNA.
NM_001079859.1	GPR64	-4.252518	Homo sapiens G protein-coupled receptor 64 (GPR64), transcript variant 2, mRNA.
NM_003106.2	SOX2	-4.239885	Homo sapiens SRY (sex determining region Y)-box 2 (SOX2), mRNA.
NM_013243.2	SCG3	-4.183976	Homo sapiens secretogranin III (SCG3), mRNA.
NM_000399.2	EGR2	-4.146733	Homo sapiens early growth response 2 (Krox-20 homolog, Drosophila) (EGR2), mRNA.
NM_181876.2	PPP2R2C	-4.127478	Homo sapiens protein phosphatase 2 (formerly 2A), regulatory subunit B, gamma isoform (PPP2R2C), transcript variant 2, mRNA.
NM_005634.2	SOX3	-4.107444	Homo sapiens SRY (sex determining region Y)-box 3 (SOX3), mRNA.

NM_004734.2	DCLK1	-4.094267	Homo sapiens doublecortin-like kinase 1 (DCLK1), mRNA.
NM_001042403.1	USP44	-4.083442	Homo sapiens ubiquitin specific peptidase 44 (USP44), transcript variant 2, mRNA.
XM_939725.2	LOC654127	-4.04052	PREDICTED: Homo sapiens similar to adaptor-related protein complex 1 sigma 2 subunit (LOC654127), mRNA.
NM_004538.3	NAP1L3	-4.008645	Homo sapiens nucleosome assembly protein 1-like 3 (NAP1L3), mRNA.
AF038185		-3.935115	Homo sapiens clone 23700 mRNA sequence
NM_080390.3	TCEAL2	-3.925044	Homo sapiens transcription elongation factor A (SII)-like 2 (TCEAL2), mRNA.
NM_032884.2	C1orf94	-3.893483	Homo sapiens chromosome 1 open reading frame 94 (C1orf94), mRNA.
NM_000216.2	KAL1	-3.884991	Homo sapiens Kallmann syndrome 1 sequence (KAL1), mRNA.
NM_012419.4	RGS17	-3.861182	Homo sapiens regulator of G-protein signaling 17 (RGS17), mRNA.
NM_000903.2	NQO1	-3.856425	Homo sapiens NAD(P)H dehydrogenase, quinone 1 (NQO1), transcript variant 1, mRNA.
NM_002851.2	PTPRZ1	-3.852374	Homo sapiens protein tyrosine phosphatase, receptor-type, Z polypeptide 1 (PTPRZ1), mRNA.
NM_014474.2	SMPDL3B	-3.841301	Homo sapiens sphingomyelin phosphodiesterase, acid-like 3B (SMPDL3B), transcript variant 1, mRNA.
NM_001042403.1	USP44	-3.830662	Homo sapiens ubiquitin specific peptidase 44 (USP44), transcript variant 2, mRNA.
NM_001080430.1	TOX3	-3.816378	Homo sapiens TOX high mobility group box family member 3 (TOX3), mRNA.
NM_004734.2	DCLK1	-3.778323	Homo sapiens doublecortin-like kinase 1 (DCLK1), mRNA.
NM_153034.2	ZNF488	-3.778085	Homo sapiens zinc finger protein 488 (ZNF488), mRNA.
NM_004430.2	EGR3	-3.770779	Homo sapiens early growth response 3 (EGR3), mRNA.
NM_002523.1	NPTX2	-3.768543	Homo sapiens neuronal pentraxin II (NPTX2), mRNA.
NM_003106.2	SOX2	-3.749071	Homo sapiens SRY (sex determining region Y)-box 2 (SOX2), mRNA.
NM_172337.1	OTX2	-3.719339	Homo sapiens orthodenticle homeobox 2 (OTX2), transcript variant 2, mRNA.
NM_152737.2	RNF182	-3.639525	Homo sapiens ring finger protein 182 (RNF182), mRNA.
NM_021127.1	PMAIP1	-3.618855	Homo sapiens phorbol-12-myristate-13-acetate-induced protein 1 (PMAIP1), mRNA.
NM_024629.2	MLF1IP	-3.608929	Homo sapiens MLF1 interacting protein (MLF1IP), mRNA.
NM_020742.2	NLGN4X	-3.574269	Homo sapiens neuroligin 4, X-linked (NLGN4X), transcript variant 1, mRNA.
NM_032803.4	SLC7A3	-3.559078	Homo sapiens solute carrier family 7 (cationic amino acid transporter, y+ system), member 3 (SLC7A3), transcript variant 1, mRNA.
NM_020211.1	RGMA	-3.537839	Homo sapiens RGM domain family, member A (RGMA), mRNA.
XM_001126803.1	LOC728185	-3.513769	PREDICTED: Homo sapiens similar to Gamma-2-syntrophin (G2SYN) (Syntrophin 5) (SYN5) (LOC728185), mRNA.
NM_002463.1	MX2	-3.508532	Homo sapiens myxovirus (influenza virus) resistance 2 (mouse) (MX2), mRNA.
NM_013244.2	MGAT4C	-3.505007	Homo sapiens mannosyl (alpha-1,3-)-glycoprotein beta-1,4-N-acetylglucosaminyltransferase, isozyme C (putative) (MGAT4C), mRNA.
NM_003865.1	HESX1	-3.503672	Homo sapiens HESX homeobox 1 (HESX1), mRNA.
NM_017522.3	LRP8	-3.496848	Homo sapiens low density lipoprotein receptor-related protein 8, apolipoprotein e receptor (LRP8), transcript variant 3, mRNA.
NM_138786.1	TM4SF18	-3.481067	Homo sapiens transmembrane 4 L six family member 18 (TM4SF18), mRNA.
NM_012183.1	FOXD3	-3.47938	Homo sapiens forkhead box D3 (FOXD3), mRNA.
NM_012281.2	KCND2	-3.47339	Homo sapiens potassium voltage-gated channel, Shal-related subfamily, member 2 (KCND2), mRNA.
NM_007223.1	GPR176	-3.473057	Homo sapiens G protein-coupled receptor 176 (GPR176), mRNA.
NM_018602.2	DNAJA4	-3.46794	Homo sapiens DnaJ (Hsp40) homolog, subfamily A, member 4 (DNAJA4), mRNA.
NM_032505.1	KBTBD8	-3.456858	Homo sapiens kelch repeat and BTB (POZ) domain containing 8 (KBTBD8), mRNA.

XM_001717875.1	LOC732445	-3.436154	PREDICTED: Homo sapiens similar to alpha 7 neuronal nicotinic acetylcholine receptor (LOC732445), mRNA.
NR_003491.1	MIAT	-3.429034	Homo sapiens myocardial infarction associated transcript (non-protein coding) (MIAT), non-coding RNA.
NM_005314.2	GRPR	-3.415805	Homo sapiens gastrin-releasing peptide receptor (GRPR), mRNA.
NM_014692.1	SEC14L5	-3.413889	Homo sapiens SEC14-like 5 (S. cerevisiae) (SEC14L5), mRNA.
NM_004522.1	KIF5C	-3.403553	Homo sapiens kinesin family member 5C (KIF5C), mRNA.
NM_001014809.1	CRMP1	-3.402081	Homo sapiens collapsin response mediator protein 1 (CRMP1), transcript variant 1, mRNA.
NM_000517.3	HBA2	-3.370179	Homo sapiens hemoglobin, alpha 2 (HBA2), mRNA.
NM_138786.2	TM4SF18	-3.345729	Homo sapiens transmembrane 4 L six family member 18 (TM4SF18), mRNA.
NM_031845.2	MAP2	-3.345285	Homo sapiens microtubule-associated protein 2 (MAP2), transcript variant 2, mRNA.
NM_182974.2	GLT6D1	-3.336456	Homo sapiens glycosyltransferase 6 domain containing 1 (GLT6D1), mRNA.
NM_021127.1	PMAIP1	-3.33604	Homo sapiens phorbol-12-myristate-13-acetate-induced protein 1 (PMAIP1), mRNA.
NM_005314.2	GRPR	-3.332223	Homo sapiens gastrin-releasing peptide receptor (GRPR), mRNA.
NM_032446.1	MEGF10	-3.317238	Homo sapiens multiple EGF-like-domains 10 (MEGF10), mRNA.
NM_005407.1	SALL2	-3.307194	Homo sapiens sal-like 2 (Drosophila) (SALL2), mRNA.
NM_178232.2	HAPLN3	-3.266422	Homo sapiens hyaluronan and proteoglycan link protein 3 (HAPLN3), mRNA.
NM_022652.2	DUSP6	-3.265109	Homo sapiens dual specificity phosphatase 6 (DUSP6), transcript variant 2, mRNA.
NM_145018.2	C11orf82	-3.263836	Homo sapiens chromosome 11 open reading frame 82 (C11orf82), mRNA.
NM_001048201.1	UHRF1	-3.259137	Homo sapiens ubiquitin-like with PHD and ring finger domains 1 (UHRF1), transcript variant 1, mRNA.
NM_015894.2	STMN3	-3.258696	Homo sapiens stathmin-like 3 (STMN3), mRNA.
NM_007129.2	ZIC2	-3.258283	Homo sapiens Zic family member 2 (odd-paired homolog, Drosophila) (ZIC2), mRNA.
NM_001005502.1	CPM	-3.255894	Homo sapiens carboxypeptidase M (CPM), transcript variant 3, mRNA.
NM_015225.1	KIAA0367	-3.252589	Homo sapiens KIAA0367 (KIAA0367), mRNA.
NM_003218.2	TERF1	-3.250534	Homo sapiens telomeric repeat binding factor (NIMA-interacting) 1 (TERF1), transcript variant 2, mRNA.
NM_013364.4	PNMA3	-3.247463	Homo sapiens paraneoplastic antigen MA3 (PNMA3), mRNA.
NM_003112.3	SP4	-3.232507	Homo sapiens Sp4 transcription factor (SP4), mRNA.
NM_000517.3	HBA2	-3.2217	Homo sapiens hemoglobin, alpha 2 (HBA2), mRNA.
XM_001128032.1	MGC87042	-3.221503	PREDICTED: Homo sapiens similar to Six transmembrane epithelial antigen of prostate (MGC87042), mRNA.
XM_498560.3	LOC440132	-3.192497	PREDICTED: Homo sapiens hypothetical LOC440132 (LOC440132), mRNA.
NM_006467.2	POLR3G	-3.185516	Homo sapiens polymerase (RNA) III (DNA directed) polypeptide G (32kD) (POLR3G), mRNA.
NM_181558.2	RFC3	-3.184396	Homo sapiens replication factor C (activator 1) 3, 38kDa (RFC3), transcript variant 2, mRNA.
NM_020997.2	LEFTY1	-3.183314	Homo sapiens left-right determination factor 1 (LEFTY1), mRNA.
NM_001048164.1	SLC7A3	-3.180488	Homo sapiens solute carrier family 7 (cationic amino acid transporter, y+ system), member 3 (SLC7A3), transcript variant 2, mRNA.
NR_029858.1	MIR302C	-3.17265	Homo sapiens microRNA 302c (MIR302C), microRNA.
NM_030763.1	NSBP1	-3.172224	Homo sapiens nucleosomal binding protein 1 (NSBP1), mRNA.
NM_017669.2	ERCC6L	-3.152213	Homo sapiens excision repair cross-complementing rodent repair deficiency, complementation group 6-like (ERCC6L), mRNA.
NM_014474.2	SMPDL3B	-3.152068	Homo sapiens sphingomyelin phosphodiesterase, acid-like 3B (SMPDL3B), transcript variant 1, mRNA.
NM_000892.3	KLKB1	-3.14934	Homo sapiens kallikrein B, plasma (Fletcher factor) 1 (KLKB1), mRNA.

NM_002374.3	MAP2	-3.147445	Homo sapiens microtubule-associated protein 2 (MAP2), transcript variant 1, mRNA.
NM_181332.1	NLGN4X	-3.145263	Homo sapiens neuroligin 4, X-linked (NLGN4X), transcript variant 2, mRNA.
NM_002006.4	FGF2	-3.123623	Homo sapiens fibroblast growth factor 2 (basic) (FGF2), mRNA.
NM_000869.2	HTR3A	-3.115791	Homo sapiens 5-hydroxytryptamine (serotonin) receptor 3A (HTR3A), transcript variant 2, mRNA.
NR_022008.1	PAR5	-3.106862	Homo sapiens Prader-Willi/Angelman syndrome-5 (PAR5), non-coding RNA.
NM_001851.3	COL9A1	-3.105283	Homo sapiens collagen, type IX, alpha 1 (COL9A1), transcript variant 1, mRNA.
XM_939432.1	MGC61598	-3.085481	PREDICTED: Homo sapiens similar to ankyrin-repeat protein Nrarp (MGC61598), mRNA.
NM_017753.2	LPPR1	-3.080231	Homo sapiens lipid phosphate phosphatase-related protein type 1 (LPPR1), transcript variant 2, mRNA.
NM_173462.3	PAPLN	-3.077175	Homo sapiens papilin, proteoglycan-like sulfated glycoprotein (PAPLN), mRNA.
NM_001040167.1	LFNG	-3.076158	Homo sapiens LFNG O-fucosylpeptide 3-beta-N-acetylglucosaminyltransferase (LFNG), transcript variant 1, mRNA.
NM_004336.2	BUB1	-3.066579	Homo sapiens BUB1 budding uninhibited by benzimidazoles 1 homolog (yeast) (BUB1), mRNA.
NM_174900.3	ZFP42	-3.054767	Homo sapiens zinc finger protein 42 homolog (mouse) (ZFP42), mRNA.
NM_203400.1	RPRML	-3.043571	Homo sapiens reprimo-like (RPRML), mRNA.
NM_003507.1	FZD7	-3.031127	Homo sapiens frizzled homolog 7 (Drosophila) (FZD7), mRNA.
NR_003083.2	SLC6A10P	-3.009961	Homo sapiens solute carrier family 6 (neurotransmitter transporter, creatine), member 10 (pseudogene) (SLC6A10P) on chromosome 16.
NM_133474.2	ZNF721	-3.007608	Homo sapiens zinc finger protein 721 (ZNF721), mRNA.
NM_002738.5	PRKCB1	-3.00235	Homo sapiens protein kinase C, beta 1 (PRKCB1), transcript variant 2, mRNA.
NM_133448.1	TMEM132D	-2.991104	Homo sapiens transmembrane protein 132D (TMEM132D), mRNA.
NM_006174.2	NPY5R	-2.988126	Homo sapiens neuropeptide Y receptor Y5 (NPY5R), mRNA.
NM_002130.6	HMGCS1	-2.982733	Homo sapiens 3-hydroxy-3-methylglutaryl-Coenzyme A synthase 1 (soluble) (HMGCS1), transcript variant 2, mRNA.
XM_929774.2	LOC646817	-2.978326	PREDICTED: Homo sapiens similar to Protein SET (Phosphatase 2A inhibitor I2PP2A) (I-2PP2A) (Template-activating factor I) (TAF-I) (HLA-DR-associated protein II) (PHAPII) (Inhibitor of granzyme A-activated DNase) (IGAAD) (LOC646817), mRNA.
NM_003739.4	AKR1C3	-2.976459	Homo sapiens aldo-keto reductase family 1, member C3 (3-alpha hydroxysteroid dehydrogenase, type II) (AKR1C3), mRNA.
NM_024333.1	FSD1	-2.975923	Homo sapiens fibronectin type III and SPRY domain containing 1 (FSD1), mRNA.
NM_006286.1	TFDP2	-2.971537	Homo sapiens transcription factor Dp-2 (E2F dimerization partner 2) (TFDP2), mRNA.
XR_000901.1	FLJ33996	-2.970323	PREDICTED: Homo sapiens hypothetical protein FLJ33996 (FLJ33996), misc RNA.
NM_003360.2	UGT8	-2.966884	Homo sapiens UDP glycosyltransferase 8 (UDP-galactose ceramide galactosyltransferase) (UGT8), mRNA.
NM_014750.3	DLGAP5	-2.961052	Homo sapiens discs, large (Drosophila) homolog-associated protein 5 (DLGAP5), mRNA.
NM_019013.1	FAM64A	-2.960819	Homo sapiens family with sequence similarity 64, member A (FAM64A), mRNA.
NM_015541.2	LRIG1	-2.95741	Homo sapiens leucine-rich repeats and immunoglobulin-like domains 1 (LRIG1), mRNA.
AL136588		-2.94806	Homo sapiens mRNA; cDNA DKFZp761D112 (from clone DKFZp761D112)
NM_018151.3	RIF1	-2.937565	Homo sapiens RAP1 interacting factor homolog (yeast) (RIF1), mRNA.
NM_004946.1	DOCK2	-2.937295	Homo sapiens dedicator of cytokinesis 2 (DOCK2), mRNA.

NM_052943.2	FAM46B	-2.928099	Homo sapiens family with sequence similarity 46, member B (FAM46B), mRNA.
XR_037397.1	LOC100129267	-2.919392	PREDICTED: Homo sapiens misc_RNA (LOC100129267), miscRNA.
XM_001724965.1	LOC100129268	-2.917975	PREDICTED: Homo sapiens hypothetical protein LOC100129268 (LOC100129268), mRNA.
NM_001814.2	CTSC	-2.914963	Homo sapiens cathepsin C (CTSC), transcript variant 1, mRNA.
NM_172109.1	KCNQ2	-2.900905	Homo sapiens potassium voltage-gated channel, KQT-like subfamily, member 2 (KCNQ2), transcript variant 5, mRNA.
NM_014618.2	DBC1	-2.896471	Homo sapiens deleted in bladder cancer 1 (DBC1), mRNA.
NM_054023.3	SCGB3A2	-2.896249	Homo sapiens secretoglobin, family 3A, member 2 (SCGB3A2), mRNA.
NM_024785.2	FAM124B	-2.881163	Homo sapiens family with sequence similarity 124B (FAM124B), transcript variant 2, mRNA.
NM_005763.2	AASS	-2.872855	Homo sapiens aminoadipate-semialdehyde synthase (AASS), nuclear gene encoding mitochondrial protein, mRNA.
NM_001002844.1	ZNF280D	-2.863286	Homo sapiens zinc finger protein 280D (ZNF280D), transcript variant 3, mRNA.
NM_001042550.1	SMC2	-2.855026	Homo sapiens structural maintenance of chromosomes 2 (SMC2), transcript variant 1, mRNA.
NM_033036.2	GAL3ST3	-2.851807	Homo sapiens galactose-3-O-sulfotransferase 3 (GAL3ST3), mRNA.
NM_017899.2	TESC	-2.851763	Homo sapiens tescalcin (TESC), mRNA.
NM_024534.4	FLJ12684	-2.842479	Homo sapiens hypothetical protein FLJ12684 (FLJ12684), mRNA. XR_001254
NM_017669.2	ERCC6L	-2.832413	Homo sapiens excision repair cross-complementing rodent repair deficiency, complementation group 6-like (ERCC6L), mRNA.
XR_038551.1	LOC389873	-2.82133	PREDICTED: Homo sapiens misc_RNA (LOC389873), miscRNA.
NR_024031.1	KIAA0114	-2.820958	Homo sapiens KIAA0114 (KIAA0114), non-coding RNA.
NM_001124.1	ADM	-2.818169	Homo sapiens adrenomedullin (ADM), mRNA.
XR_079546.1	MGC11082	-2.817518	PREDICTED: Homo sapiens hypothetical LOC84777 (MGC11082), miscRNA.
AK129542		-2.816713	Homo sapiens cDNA FLJ26031 fis, clone PNC08078
NM_000814.4	GABRB3	-2.816658	Homo sapiens gamma-aminobutyric acid (GABA) A receptor, beta 3 (GABRB3), transcript variant 1, mRNA.
NM_152999.3	STEAP2	-2.809754	Homo sapiens six transmembrane epithelial antigen of the prostate 2 (STEAP2), transcript variant 1, mRNA.
NM_144584.1	C1orf59	-2.806164	Homo sapiens chromosome 1 open reading frame 59 (C1orf59), mRNA.
NM_006440.3	TXNRD2	-2.802364	Homo sapiens thioredoxin reductase 2 (TXNRD2), nuclear gene encoding mitochondrial protein, mRNA.
NM_152999.2	STEAP2	-2.801054	Homo sapiens six transmembrane epithelial antigen of the prostate 2 (STEAP2), mRNA.
NM_005763.2	AASS	-2.798996	Homo sapiens aminoadipate-semialdehyde synthase (AASS), nuclear gene encoding mitochondrial protein, mRNA.
XM_928013.1	ACBD7	-2.797921	PREDICTED: Homo sapiens acyl-Coenzyme A binding domain containing 7 (ACBD7), mRNA.
NM_001018054.1	LRP8	-2.79367	Homo sapiens low density lipoprotein receptor-related protein 8, apolipoprotein e receptor (LRP8), transcript variant 4, mRNA.
NM_018063.3	HELLS	-2.793664	Homo sapiens helicase, lymphoid-specific (HELLS), mRNA.
NM_018169.2	C12orf35	-2.792011	Homo sapiens chromosome 12 open reading frame 35 (C12orf35), mRNA.
NM_012485.1	HMMR	-2.787879	Homo sapiens hyaluronan-mediated motility receptor (RHAMM) (HMMR), transcript variant 2, mRNA.
NM_006739.3	MCM5	-2.782203	Homo sapiens minichromosome maintenance complex component 5 (MCM5), mRNA.
XM_001725504.1	LOC100127971	-2.780612	PREDICTED: Homo sapiens hypothetical protein LOC100127971 (LOC100127971), mRNA.
NM_001039590.1	USP9X	-2.778021	Homo sapiens ubiquitin specific peptidase 9, X-linked (USP9X), transcript variant 3, mRNA.
NM_182830.2	MDGA2	-2.777948	Homo sapiens MAM domain containing glycosylphosphatidylinositol

			anchor 2 (MDGA2), mRNA.
NM_144684.1	ZNF480	-2.777488	Homo sapiens zinc finger protein 480 (ZNF480), mRNA.
NM_024504.2	PRDM14	-2.773309	Homo sapiens PR domain containing 14 (PRDM14), mRNA.
XM_001134320.1	FLJ25404	-2.767252	PREDICTED: Homo sapiens hypothetical protein FLJ25404, transcript variant 2 (FLJ25404), mRNA.
NM_001039948.2	SGSM1	-2.762705	Homo sapiens small G protein signaling modulator 1 (SGSM1), transcript variant 1, mRNA.
CR738291		-2.759997	CR738291 Homo sapiens library (Ebert L) Homo sapiens cDNA clone IMAGp971D2453 ; IMAGE:811744 5, mRNA sequence
NM_004297.2	GNA14	-2.756497	Homo sapiens guanine nucleotide binding protein (G protein), alpha 14 (GNA14), mRNA.
NM_004297.2	GNA14	-2.756497	Homo sapiens guanine nucleotide binding protein (G protein), alpha 14 (GNA14), mRNA.
NM_001813.2	CENPE	-2.756429	Homo sapiens centromere protein E, 312kDa (CENPE), mRNA.
NM_005025.3	SERPINI1	-2.756039	Homo sapiens serpin peptidase inhibitor, clade I (neuroserpin), member 1 (SERPINI1), mRNA.
NM_001009568.1	SMPDL3B	-2.753406	Homo sapiens sphingomyelin phosphodiesterase, acid-like 3B (SMPDL3B), transcript variant 2, mRNA.
NM_024090.1	ELOVL6	-2.745541	Homo sapiens ELOVL family member 6, elongation of long chain fatty acids (FEN1/Elo2, SUR4/Elo3-like, yeast) (ELOVL6), mRNA.
NM_015568.2	PPP1R16B	-2.742289	Homo sapiens protein phosphatase 1, regulatory (inhibitor) subunit 16B (PPP1R16B), mRNA.
NM_006759.3	UGP2	-2.731096	Homo sapiens UDP-glucose pyrophosphorylase 2 (UGP2), transcript variant 1, mRNA.
NM_014690.2	KIAA0773	-2.728364	Homo sapiens KIAA0773 gene product (KIAA0773), mRNA.
NM_198353.1	KCTD8	-2.721982	Homo sapiens potassium channel tetramerisation domain containing 8 (KCTD8), mRNA.
NM_032336.1	GINS4	-2.721524	Homo sapiens GINS complex subunit 4 (Sld5 homolog) (GINS4), mRNA.
NM_001011645.1	AR	-2.720785	Homo sapiens androgen receptor (AR), transcript variant 2, mRNA.
NM_014906.3	PPM1E	-2.719761	Homo sapiens protein phosphatase 1E (PP2C domain containing) (PPM1E), mRNA.
NM_019079.2	L1TD1	-2.714913	Homo sapiens LINE-1 type transposase domain containing 1 (L1TD1), mRNA.
NM_005760.2	CEBPZ	-2.700685	Homo sapiens CCAAT/enhancer binding protein (C/EBP), zeta (CEBPZ), mRNA.
XM_001720872.1	LOC100127971	-2.697535	PREDICTED: Homo sapiens hypothetical protein LOC100127971 (LOC100127971), mRNA.
NM_001814.2	CTSC	-2.697054	Homo sapiens cathepsin C (CTSC), transcript variant 1, mRNA.
NM_000745.2	CHRNA5	-2.691499	Homo sapiens cholinergic receptor, nicotinic, alpha 5 (CHRNA5), mRNA.
NM_024046.3	CAMKV	-2.689437	Homo sapiens CaM kinase-like vesicle-associated (CAMKV), mRNA.
NM_024504.2	PRDM14	-2.687915	Homo sapiens PR domain containing 14 (PRDM14), mRNA.
NM_001275.3	CHGA	-2.685702	Homo sapiens chromogranin A (parathyroid secretory protein 1) (CHGA), mRNA.
NM_001441.1	FAAH	-2.683675	Homo sapiens fatty acid amide hydrolase (FAAH), mRNA.
NM_004664.2	LIN7A	-2.680706	Homo sapiens lin-7 homolog A (C. elegans) (LIN7A), mRNA.
NM_182970.2	RIMS4	-2.68021	Homo sapiens regulating synaptic membrane exocytosis 4 (RIMS4), mRNA.
XM_001715544.1	LOC100129673	-2.677989	PREDICTED: Homo sapiens similar to hCG2042915 (LOC100129673), mRNA.
XM_928583.1	LOC645566	-2.676089	PREDICTED: Homo sapiens hypothetical protein LOC645566 (LOC645566), mRNA.
NM_018977.2	NLGN3	-2.672227	Homo sapiens neuroligin 3 (NLGN3), mRNA.
NM_212535.2	PRKCB	-2.672178	Homo sapiens protein kinase C, beta (PRKCB), transcript variant 1, mRNA.
NM_014750.3	DLGAP5	-2.670621	Homo sapiens discs, large (Drosophila) homolog-associated protein 5 (DLGAP5), mRNA.
NM_152359.1	CPT1C	-2.668714	Homo sapiens carnitine palmitoyltransferase 1C (CPT1C), transcript

			variant 2, mRNA.
NM_057162.1	KLHL4	-2.666505	Homo sapiens kelch-like 4 (Drosophila) (KLHL4), transcript variant 2, mRNA.
NM_020659.2	TTYH1	-2.663693	Homo sapiens tweety homolog 1 (Drosophila) (TTYH1), transcript variant 1, mRNA.
NM_030631.2	SLC25A21	-2.662698	Homo sapiens solute carrier family 25 (mitochondrial oxodicarboxylate carrier), member 21 (SLC25A21), nuclear gene encoding mitochondrial protein, mRNA.
NM_001033059.1	AMD1	-2.660666	Homo sapiens adenosylmethionine decarboxylase 1 (AMD1), transcript variant 2, mRNA.
CR603272		-2.658834	full-length cDNA clone CS0DC013YI10 of Neuroblastoma Cot 25-normalized of Homo sapiens (human)
NM_001042610.1	DBNDD1	-2.653589	Homo sapiens dysbindin (dystrobrevin binding protein 1) domain containing 1 (DBNDD1), transcript variant 1, mRNA.
NM_000904.2	NQO2	-2.645815	Homo sapiens NAD(P)H dehydrogenase, quinone 2 (NQO2), mRNA.
NM_198336.1	INSIG1	-2.642398	Homo sapiens insulin induced gene 1 (INSIG1), transcript variant 2, mRNA.
NM_153018.2	ZFP3	-2.642004	Homo sapiens zinc finger protein 3 homolog (mouse) (ZFP3), mRNA.
NM_001274.3	CHEK1	-2.631944	Homo sapiens CHK1 checkpoint homolog (S. pombe) (CHEK1), mRNA.
NM_017816.1	LYAR	-2.630623	Homo sapiens Ly1 antibody reactive homolog (mouse) (LYAR), mRNA.
NM_030755.4	TMX1	-2.629364	Homo sapiens thioredoxin-related transmembrane protein 1 (TMX1), mRNA.
NM_003487.2	TAF15	-2.627898	Homo sapiens TAF15 RNA polymerase II, TATA box binding protein (TBP)-associated factor, 68kDa (TAF15), transcript variant 2, mRNA.
NM_032882.2	PNMA6A	-2.622746	Homo sapiens paraneoplastic antigen like 6A (PNMA6A), mRNA.
NM_021808.2	GALNT9	-2.619482	Homo sapiens UDP-N-acetyl-alpha-D-galactosamine:polypeptide N-acetylgalactosaminyltransferase 9 (GalNAc-T9) (GALNT9), mRNA.
NM_016510.3	SCLY	-2.618228	Homo sapiens selenocysteine lyase (SCLY), mRNA.
NM_001080450.1	BEND3	-2.617563	Homo sapiens BEN domain containing 3 (BEND3), mRNA.
NM_012449.2	STEAP1	-2.617346	Homo sapiens six transmembrane epithelial antigen of the prostate 1 (STEAP1), mRNA.
NM_173078.2	SLITRK4	-2.616889	Homo sapiens SLIT and NTRK-like family, member 4 (SLITRK4), mRNA.
NM_015162.3	ACSBG1	-2.616684	Homo sapiens acyl-CoA synthetase bubblegum family member 1 (ACSBG1), mRNA.
NM_004615.2	TSPAN7	-2.616513	Homo sapiens tetraspanin 7 (TSPAN7), mRNA.
NM_003318.3	TTK	-2.615362	Homo sapiens TTK protein kinase (TTK), mRNA.
NM_006467.2	POLR3G	-2.612066	Homo sapiens polymerase (RNA) III (DNA directed) polypeptide G (32kD) (POLR3G), mRNA.
NM_198545.2	C1orf187	-2.611821	Homo sapiens chromosome 1 open reading frame 187 (C1orf187), mRNA.
BC035081		-2.609576	Homo sapiens cDNA clone IMAGE:5260685
NM_005891.2	ACAT2	-2.604513	Homo sapiens acetyl-Coenzyme A acetyltransferase 2 (ACAT2), mRNA.
NM_005375.2	MYB	-2.601354	Homo sapiens v-myb myeloblastosis viral oncogene homolog (avian) (MYB), transcript variant 2, mRNA.
NM_006000.1	TUBA4A	-2.598541	Homo sapiens tubulin, alpha 4a (TUBA4A), mRNA.
NM_012484.1	HMMR	-2.596416	Homo sapiens hyaluronan-mediated motility receptor (RHAMM) (HMMR), transcript variant 1, mRNA.
NM_207406.1	CCDC4	-2.595317	Homo sapiens coiled-coil domain containing 4 (CCDC4), mRNA.
XM_001134053.1	LOC199800	-2.591395	PREDICTED: Homo sapiens hypothetical protein LOC199800 (LOC199800), mRNA.
NM_030639.1	BHLHB9	-2.590855	Homo sapiens basic helix-loop-helix domain containing, class B, 9 (BHLHB9), mRNA.
NM_020742.2	NLGN4X	-2.581618	Homo sapiens neuroligin 4, X-linked (NLGN4X), transcript variant 1, mRNA.
NM_015560.1	OPA1	-2.581115	Homo sapiens optic atrophy 1 (autosomal dominant) (OPA1), nuclear gene encoding mitochondrial protein, transcript variant 1, mRNA.

NM_174936.2	PCSK9	-2.575492	Homo sapiens proprotein convertase subtilisin/kexin type 9 (PCSK9), mRNA.
NM_018063.3	HELLS	-2.574972	Homo sapiens helicase, lymphoid-specific (HELLS), mRNA.
NM_152562.2	CDCA2	-2.569002	Homo sapiens cell division cycle associated 2 (CDCA2), mRNA.
NM_000814.4	GABRB3	-2.567669	Homo sapiens gamma-aminobutyric acid (GABA) A receptor, beta 3 (GABRB3), transcript variant 1, mRNA.
NM_198545.3	C1orf187	-2.567603	Homo sapiens chromosome 1 open reading frame 187 (C1orf187), mRNA.
NM_001018109.1	PIR	-2.566798	Homo sapiens pirin (iron-binding nuclear protein) (PIR), transcript variant 2, mRNA.
NM_181864.2	ACOT7	-2.56301	Homo sapiens acyl-CoA thioesterase 7 (ACOT7), transcript variant hBACHb, mRNA.
NM_153768.1	CABYR	-2.56226	Homo sapiens calcium binding tyrosine-(Y)-phosphorylation regulated (CABYR), transcript variant 2, mRNA.
NM_022770.2	GINS3	-2.561967	Homo sapiens GINS complex subunit 3 (Psf3 homolog) (GINS3), mRNA.
NM_173798.2	ZCCHC12	-2.559364	Homo sapiens zinc finger, CCHC domain containing 12 (ZCCHC12), mRNA.
XR_015766.1	LOC730051	-2.558471	PREDICTED: Homo sapiens similar to Zinc finger protein 418 (LOC730051), mRNA.
NM_019035.2	PCDH18	-2.558286	Homo sapiens protocadherin 18 (PCDH18), mRNA.
NM_138375.1	CABLES1	-2.557892	Homo sapiens Cdk5 and Abl enzyme substrate 1 (CABLES1), transcript variant 1, mRNA.
NM_032512.2	PDZD4	-2.557818	Homo sapiens PDZ domain containing 4 (PDZD4), mRNA.
NM_016567.2	BCCIP	-2.557776	Homo sapiens BRCA2 and CDKN1A interacting protein (BCCIP), transcript variant A, mRNA.
NM_003112.3	SP4	-2.557231	Homo sapiens Sp4 transcription factor (SP4), mRNA.
XR_041527.1	ZNF788	-2.553638	PREDICTED: Homo sapiens misc_RNA (ZNF788), miscRNA.
NM_144978.1	CCDC138	-2.553385	Homo sapiens coiled-coil domain containing 138 (CCDC138), mRNA.
NM_004624.2	VIPR1	-2.551401	Homo sapiens vasoactive intestinal peptide receptor 1 (VIPR1), mRNA.
NM_018361.2	AGPAT5	-2.549224	Homo sapiens 1-acylglycerol-3-phosphate O-acyltransferase 5 (lysophosphatidic acid acyltransferase, epsilon) (AGPAT5), mRNA.
NM_005513.1	GTF2E1	-2.549152	Homo sapiens general transcription factor IIE, polypeptide 1 (alpha subunit, 56kD) (GTF2E1), mRNA.
NM_001038.4	SCNN1A	-2.547818	Homo sapiens sodium channel, nonvoltage-gated 1 alpha (SCNN1A), mRNA.
NM_001097635.1	GCNT1	-2.547293	Homo sapiens glucosaminyl (N-acetyl) transferase 1, core 2 (beta-1,6-N-acetylglucosaminyltransferase) (GCNT1), transcript variant 4, mRNA.
XR_037892.1	LOC100130550	-2.544705	PREDICTED: Homo sapiens misc_RNA (LOC100130550), miscRNA.
NM_001002919.2	FAM150B	-2.537784	Homo sapiens family with sequence similarity 150, member B (FAM150B), mRNA.
NM_017745.4	BCOR	-2.537144	Homo sapiens BCL6 co-repressor (BCOR), transcript variant 1, mRNA.
NM_033222.2	PSIP1	-2.535331	Homo sapiens PC4 and SFRS1 interacting protein 1 (PSIP1), transcript variant 2, mRNA.
NM_003980.3	MAP7	-2.532212	Homo sapiens microtubule-associated protein 7 (MAP7), mRNA.
NM_018644.3	B3GAT1	-2.52683	Homo sapiens beta-1,3-glucuronyltransferase 1 (glucuronosyltransferase P) (B3GAT1), transcript variant 1, mRNA.
NM_021968.3	HIST1H4J	-2.521186	Homo sapiens histone cluster 1, H4j (HIST1H4J), mRNA.
NM_020300.3	MGST1	-2.520732	Homo sapiens microsomal glutathione S-transferase 1 (MGST1), transcript variant 1b, mRNA.
NM_001316.2	CSE1L	-2.519681	Homo sapiens CSE1 chromosome segregation 1-like (yeast) (CSE1L), mRNA.
NM_001001995.1	GPM6B	-2.519282	Homo sapiens glycoprotein M6B (GPM6B), transcript variant 1, mRNA.
NM_018151.3	RIF1	-2.515646	Homo sapiens RAP1 interacting factor homolog (yeast) (RIF1), mRNA.
NM_020926.2	BCOR	-2.514079	Homo sapiens BCL6 co-repressor (BCOR), transcript variant 2, mRNA.
NM_182728.1	SLC7A8	-2.513152	Homo sapiens solute carrier family 7 (cationic amino acid transporter, y+ system), member 8 (SLC7A8), transcript variant 2, mRNA.
NM_024094.1	DSCC1	-2.508388	Homo sapiens defective in sister chromatid cohesion 1 homolog (S.

			cerevisiae) (DSCC1), mRNA.
NM_001039876.1	C19orf46	-2.508177	Homo sapiens chromosome 19 open reading frame 46 (C19orf46), mRNA.
NM_005382.1	NEFM	-2.50674	Homo sapiens neurofilament, medium polypeptide 150kDa (NEFM), mRNA.
NM_030666.2	SERPINB1	-2.506226	Homo sapiens serpin peptidase inhibitor, clade B (ovalbumin), member 1 (SERPINB1), mRNA.
NM_020675.3	SPC25	-2.503434	Homo sapiens SPC25, NDC80 kinetochore complex component, homolog (S. cerevisiae) (SPC25), mRNA.
NM_080872.1	UNC5D	-2.502088	Homo sapiens unc-5 homolog D (C. elegans) (UNC5D), mRNA.
CR603183		-2.501954	full-length cDNA clone CS0DM012YE14 of Fetal liver of Homo sapiens (human)
NM_000057.2	BLM	-2.500414	Homo sapiens Bloom syndrome, RecQ helicase-like (BLM), mRNA.
NM_002461.1	MVD	-2.497685	Homo sapiens mevalonate (diphospho) decarboxylase (MVD), mRNA.
NM_178562.2	TSPAN33	-2.495402	Homo sapiens tetraspanin 33 (TSPAN33), mRNA.
AK123319		-2.494066	Homo sapiens cDNA FLJ41325 fis, clone BRAMY2046871
NM_005915.4	MCM6	-2.493921	Homo sapiens minichromosome maintenance complex component 6 (MCM6), mRNA.
NM_014996.1	PLCH1	-2.490897	Homo sapiens phospholipase C, eta 1 (PLCH1), mRNA.
NM_017816.1	LYAR	-2.488899	Homo sapiens Ly1 antibody reactive homolog (mouse) (LYAR), mRNA.
NM_033201.1	C16orf45	-2.488588	Homo sapiens chromosome 16 open reading frame 45 (C16orf45), mRNA.
NM_138374.1	ZNF845	-2.485464	Homo sapiens zinc finger protein 845 (ZNF845), mRNA. XM_039908
NR_002940.1	LRRC37A4	-2.485131	Homo sapiens leucine rich repeat containing 37, member A4 (pseudogene) (LRRC37A4), non-coding RNA. XM_934274 XM_941652 XM_945358 XM_945361 XM_945364
NM_001100.3	ACTA1	-2.483246	Homo sapiens actin, alpha 1, skeletal muscle (ACTA1), mRNA.
NM_014736.4	KIAA0101	-2.480605	Homo sapiens KIAA0101 (KIAA0101), transcript variant 1, mRNA.
NM_017915.2	C12orf48	-2.479953	Homo sapiens chromosome 12 open reading frame 48 (C12orf48), mRNA.
NM_001002919.1	LOC285016	-2.479288	Homo sapiens hypothetical protein LOC285016 (LOC285016), mRNA.
NM_001197.3	BIK	-2.478377	Homo sapiens BCL2-interacting killer (apoptosis-inducing) (BIK), mRNA.
NM_004523.2	KIF11	-2.478367	Homo sapiens kinesin family member 11 (KIF11), mRNA.
NM_017711.2	GDPD2	-2.478335	Homo sapiens glycerophosphodiester phosphodiesterase domain containing 2 (GDPD2), mRNA.
NR_024281.1	LOC157627	-2.478044	Homo sapiens hypothetical LOC157627 (LOC157627), non-coding RNA.
NM_019030.2	DHX29	-2.477001	Homo sapiens DEAH (Asp-Glu-Ala-His) box polypeptide 29 (DHX29), mRNA.
NM_014790.3	JAKMIP2	-2.473336	Homo sapiens janus kinase and microtubule interacting protein 2 (JAKMIP2), mRNA.
NM_015541.2	LRIG1	-2.470183	Homo sapiens leucine-rich repeats and immunoglobulin-like domains 1 (LRIG1), mRNA.
NM_033625.2	RPL34	-2.467607	Homo sapiens ribosomal protein L34 (RPL34), transcript variant 2, mRNA.
NM_024565.5	CCNJL	-2.466302	Homo sapiens cyclin J-like (CCNJL), mRNA.
NM_014762.3	DHCR24	-2.458714	Homo sapiens 24-dehydrocholesterol reductase (DHCR24), mRNA.
NM_182746.1	MCM4	-2.454214	Homo sapiens minichromosome maintenance complex component 4 (MCM4), transcript variant 2, mRNA.
NM_005445.3	SMC3	-2.453964	Homo sapiens structural maintenance of chromosomes 3 (SMC3), mRNA.
NM_018360.1	CXorf15	-2.452717	Homo sapiens chromosome X open reading frame 15 (CXorf15), mRNA.
NM_018369.1	DEPDC1B	-2.45203	Homo sapiens DEP domain containing 1B (DEPDC1B), mRNA.
NM_152312.3	GYLTL1B	-2.450864	Homo sapiens glycosyltransferase-like 1B (GYLTL1B), mRNA.
NM_014264.3	PLK4	-2.450081	Homo sapiens polo-like kinase 4 (Drosophila) (PLK4), mRNA.
NM_021963.2	NAP1L2	-2.449983	Homo sapiens nucleosome assembly protein 1-like 2 (NAP1L2), mRNA.
NM_174976.2	ZDHHC22	-2.448561	Homo sapiens zinc finger, DHHC-type containing 22 (ZDHHC22), mRNA.
NM_016195.2	KIF20B	-2.447154	Homo sapiens kinesin family member 20B (KIF20B), mRNA.

NM_012247.3	SEPHS1	-2.447047	Homo sapiens selenophosphate synthetase 1 (SEPHS1), mRNA.
NM_006286.1	TFDP2	-2.446586	Homo sapiens transcription factor Dp-2 (E2F dimerization partner 2) (TFDP2), mRNA.
NM_004490.2	GRB14	-2.443434	Homo sapiens growth factor receptor-bound protein 14 (GRB14), mRNA.
NM_182679.1	GPATCH4	-2.442508	Homo sapiens G patch domain containing 4 (GPATCH4), transcript variant 2, mRNA.
NM_001013437.1	SEH1L	-2.441029	Homo sapiens SEH1-like (S. cerevisiae) (SEH1L), transcript variant 1, mRNA.
NM_015678.3	NBEA	-2.437975	Homo sapiens neurobeachin (NBEA), mRNA.
NM_001316.2	CSE1L	-2.436721	Homo sapiens CSE1 chromosome segregation 1-like (yeast) (CSE1L), mRNA.
NM_024595.1	AKIRIN1	-2.436027	Homo sapiens akirin 1 (AKIRIN1), mRNA.
XR_016129.1	LOC653108	-2.435504	PREDICTED: Homo sapiens similar to coxsackie virus and adenovirus receptor precursor (LOC653108), mRNA.
NM_030928.2	CDT1	-2.433737	Homo sapiens chromatin licensing and DNA replication factor 1 (CDT1), mRNA.
NM_015028.1	TNIK	-2.432942	Homo sapiens TRAF2 and NCK interacting kinase (TNIK), mRNA.
NM_018365.1	MNS1	-2.432063	Homo sapiens meiosis-specific nuclear structural 1 (MNS1), mRNA.
NM_152524.3	SGOL2	-2.417783	Homo sapiens shugoshin-like 2 (S. pombe) (SGOL2), mRNA.
NM_024945.2	RMI1	-2.415608	Homo sapiens RMI1, RecQ mediated genome instability 1, homolog (S. cerevisiae) (RMI1), mRNA.
NM_018087.3	TMEM48	-2.415343	Homo sapiens transmembrane protein 48 (TMEM48), mRNA.
NM_017934.4	PHIP	-2.413013	Homo sapiens pleckstrin homology domain interacting protein (PHIP), mRNA.
NM_017954.9	CADPS2	-2.412455	Homo sapiens Ca++-dependent secretion activator 2 (CADPS2), transcript variant 1, mRNA.
NM_024860.1	SETD6	-2.411448	Homo sapiens SET domain containing 6 (SETD6), mRNA.
NM_033267.3	IRX2	-2.410852	Homo sapiens iroquois homeobox 2 (IRX2), mRNA.
NM_005440.3	RND2	-2.406724	Homo sapiens Rho family GTPase 2 (RND2), mRNA.
NM_020927.1	VAT1L	-2.405415	Homo sapiens vesicle amine transport protein 1 homolog (T. californica)-like (VAT1L), mRNA.
NM_017906.2	PAK1IP1	-2.404698	Homo sapiens PAK1 interacting protein 1 (PAK1IP1), mRNA.
NM_198175.1	NME1	-2.403625	Homo sapiens non-metastatic cells 1, protein (NM23A) expressed in (NME1), transcript variant 1, mRNA.
NM_020819.2	FAM135A	-2.402734	Homo sapiens family with sequence similarity 135, member A (FAM135A), mRNA.
NM_004432.2	ELAVL2	-2.402218	Homo sapiens ELAV (embryonic lethal, abnormal vision, Drosophila)-like 2 (Hu antigen B) (ELAVL2), mRNA.
NM_001813.2	CENPE	-2.400644	Homo sapiens centromere protein E, 312kDa (CENPE), mRNA.
NM_152649.1	MLKL	-2.400346	Homo sapiens mixed lineage kinase domain-like (MLKL), mRNA.
NM_024749.2	VASH2	-2.398513	Homo sapiens vasohibin 2 (VASH2), mRNA.
NM_152672.4	OSTalpha	-2.397985	Homo sapiens organic solute transporter alpha (OSTalpha), mRNA.
NM_207406.2	BEND4	-2.39769	Homo sapiens BEN domain containing 4 (BEND4), mRNA.
NM_032776.1	JMJD1C	-2.396438	Homo sapiens jumonji domain containing 1C (JMJD1C), transcript variant 1, mRNA.
NM_017975.3	ZWILCH	-2.394908	Homo sapiens Zwilch, kinetochore associated, homolog (Drosophila) (ZWILCH), transcript variant 1, mRNA.
NM_014109.2	ATAD2	-2.394525	Homo sapiens ATPase family, AAA domain containing 2 (ATAD2), mRNA.
NM_024090.1	ELOVL6	-2.391472	Homo sapiens ELOVL family member 6, elongation of long chain fatty acids (FEN1/Elo2, SUR4/Elo3-like, yeast) (ELOVL6), mRNA.
NM_001448.2	GPC4	-2.389164	Homo sapiens glypican 4 (GPC4), mRNA.
XM_373461.3	LOC387683	-2.388867	PREDICTED: Homo sapiens hypothetical LOC387683 (LOC387683), mRNA.
NM_005193.1	CDX4	-2.388466	Homo sapiens caudal type homeobox 4 (CDX4), mRNA.
NM_145061.3	C13orf3	-2.388271	Homo sapiens chromosome 13 open reading frame 3 (C13orf3), mRNA.
NM_005656.2	TMPRSS2	-2.387695	Homo sapiens transmembrane protease, serine 2 (TMPRSS2), mRNA.

NM_016391.4	NOP16	-2.386996	Homo sapiens NOP16 nucleolar protein homolog (yeast) (NOP16), mRNA.
XM_946212.2	FLJ44379	-2.385273	PREDICTED: Homo sapiens similar to S-100 protein, alpha chain, transcript variant 3 (FLJ44379), mRNA.
NM_021128.3	POLR2L	-2.385158	Homo sapiens polymerase (RNA) II (DNA directed) polypeptide L, 7.6kDa (POLR2L), mRNA.
NM_013230.2	CD24	-2.384854	Homo sapiens CD24 molecule (CD24), mRNA.
NM_021992.2	TMSB15A	-2.384516	Homo sapiens thymosin beta 15a (TMSB15A), mRNA.
NM_030622.6	CYP2S1	-2.38216	Homo sapiens cytochrome P450, family 2, subfamily S, polypeptide 1 (CYP2S1), mRNA.
NM_018644.3	B3GAT1	-2.381834	Homo sapiens beta-1,3-glucuronyltransferase 1 (glucuronosyltransferase P) (B3GAT1), transcript variant 1, mRNA.
NM_001338.3	CXADR	-2.378511	Homo sapiens coxsackie virus and adenovirus receptor (CXADR), mRNA.
NM_006759.3	UGP2	-2.378334	Homo sapiens UDP-glucose pyrophosphorylase 2 (UGP2), transcript variant 1, mRNA.
NM_005483.2	CHAF1A	-2.375863	Homo sapiens chromatin assembly factor 1, subunit A (p150) (CHAF1A), mRNA.
NM_014317.3	PDSS1	-2.37556	Homo sapiens prenyl (decaprenyl) diphosphate synthase, subunit 1 (PDSS1), mRNA.
NM_181676.1	PPP2R2B	-2.375048	Homo sapiens protein phosphatase 2 (formerly 2A), regulatory subunit B, beta isoform (PPP2R2B), transcript variant 4, mRNA.
NM_000530.3	MPZ	-2.374266	Homo sapiens myelin protein zero (Charcot-Marie-Tooth neuropathy 1B) (MPZ), mRNA.
NM_203394.2	E2F7	-2.371138	Homo sapiens E2F transcription factor 7 (E2F7), mRNA.
NM_012250.3	RRAS2	-2.369973	Homo sapiens related RAS viral (r-ras) oncogene homolog 2 (RRAS2), mRNA.
NM_001025249.1	DUT	-2.369185	Homo sapiens deoxyuridine triphosphatase (DUT), nuclear gene encoding mitochondrial protein, transcript variant 3, mRNA.
NM_145014.1	HYLS1	-2.368543	Homo sapiens hydrolethalus syndrome 1 (HYLS1), mRNA.
XM_001715311.1	LOC100128485	-2.367755	PREDICTED: Homo sapiens similar to conserved hypothetical protein (LOC100128485), mRNA.
NR_002789.1	LOC168474	-2.365809	Homo sapiens selenophosphate synthetase pseudogene (LOC168474), non-coding RNA.
XR_037327.1	LOC100128007	-2.36182	PREDICTED: Homo sapiens misc_RNA (LOC100128007), miscRNA.
NM_020890.1	KIAA1524	-2.361463	Homo sapiens KIAA1524 (KIAA1524), mRNA.
AK123661		-2.361028	Homo sapiens cDNA FLJ41667 fis, clone FEBRA2028366
NM_033213.2	ZNF670	-2.36087	Homo sapiens zinc finger protein 670 (ZNF670), mRNA.
NM_052899.2	GPRIN1	-2.360495	Homo sapiens G protein regulated inducer of neurite outgrowth 1 (GPRIN1), mRNA.
NM_006101.1	NDC80	-2.359413	Homo sapiens NDC80 homolog, kinetochore complex component (S. cerevisiae) (NDC80), mRNA.
NM_006159.1	NELL2	-2.353069	Homo sapiens NEL-like 2 (chicken) (NELL2), mRNA.
NM_152280.2	SYT11	-2.352348	Homo sapiens synaptotagmin XI (SYT11), mRNA.
NM_016391.3	HSPC111	-2.352144	Homo sapiens hypothetical protein HSPC111 (HSPC111), mRNA.
NM_016518.2	PIPOX	-2.351986	Homo sapiens pipecolic acid oxidase (PIPOX), mRNA.
NM_021190.1	PTBP2	-2.350104	Homo sapiens polypyrimidine tract binding protein 2 (PTBP2), mRNA.
NM_005445.3	SMC3	-2.349912	Homo sapiens structural maintenance of chromosomes 3 (SMC3), mRNA.
NM_001024662.1	RPL6	-2.349645	Homo sapiens ribosomal protein L6 (RPL6), transcript variant 1, mRNA.
NM_006690.3	MMP24	-2.347497	Homo sapiens matrix metallopeptidase 24 (membrane-inserted) (MMP24), mRNA.
NM_014363.3	SACS	-2.347022	Homo sapiens spastic ataxia of Charlevoix-Saguenay (sacsin) (SACS), mRNA.
NM_000814.4	GABRB3	-2.343051	Homo sapiens gamma-aminobutyric acid (GABA) A receptor, beta 3 (GABRB3), transcript variant 1, mRNA.
NM_014739.2	BCLAF1	-2.342602	Homo sapiens BCL2-associated transcription factor 1 (BCLAF1), transcript variant 1, mRNA.

NM_002735.1	PRKAR1B	-2.342474	Homo sapiens protein kinase, cAMP-dependent, regulatory, type I, beta (PRKAR1B), mRNA.
NM_015462.3	NOL11	-2.34159	Homo sapiens nucleolar protein 11 (NOL11), mRNA.
NM_020819.2	FAM135A	-2.337071	Homo sapiens family with sequence similarity 135, member A (FAM135A), mRNA.
NM_000322.3	PRPH2	-2.336572	Homo sapiens peripherin 2 (retinal degeneration, slow) (PRPH2), mRNA.
NM_004181.3	UCHL1	-2.334514	Homo sapiens ubiquitin carboxyl-terminal esterase L1 (ubiquitin thiolesterase) (UCHL1), mRNA.
XM_209104.2	LOC284293	-2.333931	PREDICTED: Homo sapiens similar to Placental thrombin inhibitor (Cytoplasmic antiproteinase) (CAP) (Protease inhibitor 6) (PI-6) (Serpin B6) (LOC284293), mRNA.
XM_001134002.1	C17orf41	-2.332753	PREDICTED: Homo sapiens chromosome 17 open reading frame 41 (C17orf41), mRNA.
NM_000478.3	ALPL	-2.331149	Homo sapiens alkaline phosphatase, liver/bone/kidney (ALPL), transcript variant 1, mRNA.
NM_018518.3	MCM10	-2.33057	Homo sapiens minichromosome maintenance complex component 10 (MCM10), transcript variant 2, mRNA.
NM_000170.2	GLDC	-2.330469	Homo sapiens glycine dehydrogenase (decarboxylating) (GLDC), mRNA.
NM_020242.1	KIF15	-2.328548	Homo sapiens kinesin family member 15 (KIF15), mRNA.
NM_182553.1	CNIH2	-2.326781	Homo sapiens cornichon homolog 2 (Drosophila) (CNIH2), mRNA.
NM_020692.1	GALNTL1	-2.325452	Homo sapiens UDP-N-acetyl-alpha-D-galactosamine:polypeptide N-acetylgalactosaminyltransferase-like 1 (GALNTL1), mRNA.
NM_001039111.1	TRIM71	-2.324831	Homo sapiens tripartite motif-containing 71 (TRIM71), mRNA.
NM_014875.1	KIF14	-2.32423	Homo sapiens kinesin family member 14 (KIF14), mRNA.
NM_003026.1	SH3GL2	-2.323926	Homo sapiens SH3-domain GRB2-like 2 (SH3GL2), mRNA.
NM_180991.4	SLCO4C1	-2.323851	Homo sapiens solute carrier organic anion transporter family, member 4C1 (SLCO4C1), mRNA.
NM_004728.2	DDX21	-2.323712	Homo sapiens DEAD (Asp-Glu-Ala-Asp) box polypeptide 21 (DDX21), mRNA.
NM_004448.2	ERBB2	-2.32327	Homo sapiens v-erb-b2 erythroblastic leukemia viral oncogene homolog 2, neuro/glioblastoma derived oncogene homolog (avian) (ERBB2), transcript variant 1, mRNA.
NM_080599.1	UPF2	-2.320852	Homo sapiens UPF2 regulator of nonsense transcripts homolog (yeast) (UPF2), transcript variant 1, mRNA.
XM_001723077.1	LOC100132934	-2.320723	PREDICTED: Homo sapiens similar to hCG1644233 (LOC100132934), mRNA.
NM_021615.4	CHST6	-2.320083	Homo sapiens carbohydrate (N-acetylglucosamine 6-O) sulfotransferase 6 (CHST6), mRNA.
NM_001449.3	FHL1	-2.318175	Homo sapiens four and a half LIM domains 1 (FHL1), mRNA.
NM_014932.2	NLGN1	-2.315733	Homo sapiens neuroligin 1 (NLGN1), mRNA.
NM_001039591.2	USP9X	-2.313567	Homo sapiens ubiquitin specific peptidase 9, X-linked (USP9X), transcript variant 4, mRNA.
NM_007274.3	ACOT7	-2.312653	Homo sapiens acyl-CoA thioesterase 7 (ACOT7), transcript variant hBACHa, mRNA.
NM_024755.2	SLTM	-2.310426	Homo sapiens SAFB-like, transcription modulator (SLTM), transcript variant 1, mRNA.
NM_015414.2	RPL36	-2.310257	Homo sapiens ribosomal protein L36 (RPL36), transcript variant 2, mRNA.
NM_024674.4	LIN28	-2.309441	Homo sapiens lin-28 homolog (C. elegans) (LIN28), mRNA.
NM_003716.2	CADPS	-2.309055	Homo sapiens Ca2+-dependent secretion activator (CADPS), transcript variant 1, mRNA.
NM_005639.1	SYT1	-2.308776	Homo sapiens synaptotagmin I (SYT1), mRNA.
NM_024645.2	ZMAT4	-2.306506	Homo sapiens zinc finger, matrin type 4 (ZMAT4), transcript variant 1, mRNA.
NM_016014.2	FAM108B1	-2.305399	Homo sapiens family with sequence similarity 108, member B1 (FAM108B1), transcript variant 1, mRNA.
NM_007280.1	OIP5	-2.304904	Homo sapiens Opa interacting protein 5 (OIP5), mRNA.

U56251		-2.304339	Human HeLa mRNA isolated as a false positive in a two-hybrid-screen
NM_001002019.1	PUS1	-2.300626	Homo sapiens pseudouridylate synthase 1 (PUS1), transcript variant 2, mRNA.
NM_024333.1	FSD1	-2.297574	Homo sapiens fibronectin type III and SPRY domain containing 1 (FSD1), mRNA.
NM_003121.2	SPIB	-2.296659	Homo sapiens Spi-B transcription factor (Spi-1/PU.1 related) (SPIB), mRNA.
NM_145176.2	SLC2A12	-2.29604	Homo sapiens solute carrier family 2 (facilitated glucose transporter), member 12 (SLC2A12), mRNA.
NM_001360.2	DHCR7	-2.295874	Homo sapiens 7-dehydrocholesterol reductase (DHCR7), transcript variant 1, mRNA.
XM_931068.1	C13orf25	-2.293515	PREDICTED: Homo sapiens chromosome 13 open reading frame 25 (C13orf25), mRNA.
NM_004523.2	KIF11	-2.29307	Homo sapiens kinesin family member 11 (KIF11), mRNA.
NM_006372.3	SYNCRIP	-2.292412	Homo sapiens synaptotagmin binding, cytoplasmic RNA interacting protein (SYNCRIP), mRNA.
NM_005378.4	MYCN	-2.291062	Homo sapiens v-myc myelocytomatosis viral related oncogene, neuroblastoma derived (avian) (MYCN), mRNA.
NM_004615.2	TSPAN7	-2.290982	Homo sapiens tetraspanin 7 (TSPAN7), mRNA.
NM_021190.1	PTBP2	-2.290735	Homo sapiens polypyrimidine tract binding protein 2 (PTBP2), mRNA.
NM_001360.1	DHCR7	-2.2903	Homo sapiens 7-dehydrocholesterol reductase (DHCR7), mRNA.
NM_000995.2	RPL34	-2.28994	Homo sapiens ribosomal protein L34 (RPL34), transcript variant 1, mRNA.
NM_005689.1	ABCB6	-2.289676	Homo sapiens ATP-binding cassette, sub-family B (MDR/TAP), member 6 (ABCB6), nuclear gene encoding mitochondrial protein, mRNA.
XR_039217.1	LOC100130769	-2.289634	PREDICTED: Homo sapiens misc_RNA (LOC100130769), miscRNA.
NM_005025.2	SERPINI1	-2.288278	Homo sapiens serpin peptidase inhibitor, clade I (neuroserpin), member 1 (SERPINI1), mRNA.
NM_001789.2	CDC25A	-2.287323	Homo sapiens cell division cycle 25 homolog A (S. pombe) (CDC25A), transcript variant 1, mRNA.
NM_020836.2	BEGAIN	-2.286561	Homo sapiens brain-enriched guanylate kinase-associated homolog (rat) (BEGAIN), mRNA.
NM_018476.3	BEX1	-2.284646	Homo sapiens brain expressed, X-linked 1 (BEX1), mRNA.
NM_021723.2	ADAM22	-2.284512	Homo sapiens ADAM metallopeptidase domain 22 (ADAM22), transcript variant 1, mRNA.
NM_005391.2	PDK3	-2.284511	Homo sapiens pyruvate dehydrogenase kinase, isozyme 3 (PDK3), mRNA.
NM_001001995.1	GPM6B	-2.284144	Homo sapiens glycoprotein M6B (GPM6B), transcript variant 1, mRNA.
NM_001008708.1	CHAC2	-2.282615	Homo sapiens ChaC, cation transport regulator homolog 2 (E. coli) (CHAC2), mRNA.
NM_003822.3	NR5A2	-2.282109	Homo sapiens nuclear receptor subfamily 5, group A, member 2 (NR5A2), transcript variant 2, mRNA.
NM_000251.1	MSH2	-2.281796	Homo sapiens mutS homolog 2, colon cancer, nonpolyposis type 1 (E. coli) (MSH2), mRNA.
AK000776		-2.278745	Homo sapiens cDNA FLJ20769 fis, clone COL06674
NM_001007157.1	PHF14	-2.278036	Homo sapiens PHD finger protein 14 (PHF14), transcript variant 1, mRNA.
NM_004526.2	MCM2	-2.276597	Homo sapiens minichromosome maintenance complex component 2 (MCM2), mRNA.
NM_030622.6	CYP2S1	-2.276331	Homo sapiens cytochrome P450, family 2, subfamily S, polypeptide 1 (CYP2S1), mRNA.
NM_052896.3	CSMD2	-2.274462	Homo sapiens CUB and Sushi multiple domains 2 (CSMD2), mRNA.
NM_015596.1	KLK13	-2.27434	Homo sapiens kallikrein-related peptidase 13 (KLK13), mRNA.
NM_032415.2	CARD11	-2.270161	Homo sapiens caspase recruitment domain family, member 11 (CARD11), mRNA.
NM_014480.1	ZNF544	-2.269865	Homo sapiens zinc finger protein 544 (ZNF544), mRNA.
NM_006426.1	DPYSL4	-2.268969	Homo sapiens dihydropyrimidinase-like 4 (DPYSL4), mRNA.

NR_001292.1	SNORD108	-2.267843	Homo sapiens small nucleolar RNA, C/D box 108 (SNORD108), small nucleolar RNA.
NM_003885.2	CDK5R1	-2.263895	Homo sapiens cyclin-dependent kinase 5, regulatory subunit 1 (p35) (CDK5R1), mRNA.
NM_013250.1	ZNF215	-2.262814	Homo sapiens zinc finger protein 215 (ZNF215), mRNA.
NM_014862.3	ARNT2	-2.262698	Homo sapiens aryl-hydrocarbon receptor nuclear translocator 2 (ARNT2), mRNA.
NM_024554.2	PGBD5	-2.262277	Homo sapiens piggyBac transposable element derived 5 (PGBD5), mRNA.
NM_152329.3	PPIL5	-2.262052	Homo sapiens peptidylprolyl isomerase (cyclophilin)-like 5 (PPIL5), transcript variant 1, mRNA.
NM_018369.1	DEPDC1B	-2.261629	Homo sapiens DEP domain containing 1B (DEPDC1B), mRNA.
NM_018136.3	ASPM	-2.259969	Homo sapiens asp (abnormal spindle) homolog, microcephaly associated (Drosophila) (ASPM), mRNA.
NM_012202.1	GNG3	-2.25968	Homo sapiens guanine nucleotide binding protein (G protein), gamma 3 (GNG3), mRNA.
NM_198336.1	INSIG1	-2.256387	Homo sapiens insulin induced gene 1 (INSIG1), transcript variant 2, mRNA.
NM_198445.2	RINL	-2.256382	Homo sapiens Ras and Rab interactor-like (RINL), mRNA.
NM_018518.3	MCM10	-2.255763	Homo sapiens minichromosome maintenance complex component 10 (MCM10), transcript variant 2, mRNA.
NM_024719.2	GRTP1	-2.251642	Homo sapiens growth hormone regulated TBC protein 1 (GRTP1), mRNA.
NM_005802.2	TOPORS	-2.25009	Homo sapiens topoisomerase I binding, arginine/serine-rich (TOPORS), mRNA.
NM_019035.2	PCDH18	-2.246307	Homo sapiens protocadherin 18 (PCDH18), mRNA.
NM_016937.2	POLA1	-2.245145	Homo sapiens polymerase (DNA directed), alpha 1, catalytic subunit (POLA1), mRNA.
NM_020436.2	SALL4	-2.244758	Homo sapiens sal-like 4 (Drosophila) (SALL4), mRNA.
NM_138467.1	TYW3	-2.242608	Homo sapiens tRNA-yW synthesizing protein 3 homolog (S. cerevisiae) (TYW3), mRNA.
NM_016122.2	CCDC41	-2.238745	Homo sapiens coiled-coil domain containing 41 (CCDC41), transcript variant 1, mRNA.
NM_001080449.1	DNA2	-2.23597	Homo sapiens DNA replication helicase 2 homolog (yeast) (DNA2), mRNA.
NM_153267.3	MAMDC2	-2.235629	Homo sapiens MAM domain containing 2 (MAMDC2), mRNA.
NM_144949.2	SOCS5	-2.235346	Homo sapiens suppressor of cytokine signaling 5 (SOCS5), transcript variant 2, mRNA.
NM_173582.3	PGM2L1	-2.232593	Homo sapiens phosphoglucomutase 2-like 1 (PGM2L1), mRNA.
NM_178558.3	ZNF680	-2.229517	Homo sapiens zinc finger protein 680 (ZNF680), mRNA.
NM_181676.1	PPP2R2B	-2.22856	Homo sapiens protein phosphatase 2 (formerly 2A), regulatory subunit B, beta isoform (PPP2R2B), transcript variant 4, mRNA.
NM_032805.1	ZSCAN10	-2.228432	Homo sapiens zinc finger and SCAN domain containing 10 (ZSCAN10), mRNA.
NM_057175.3	NARG1	-2.227658	Homo sapiens NMDA receptor regulated 1 (NARG1), mRNA.
NM_054016.1	SFRS13A	-2.226336	Homo sapiens splicing factor, arginine/serine-rich 13A (SFRS13A), transcript variant 2, mRNA.
NM_004153.2	ORC1L	-2.224933	Homo sapiens origin recognition complex, subunit 1-like (yeast) (ORC1L), mRNA.
NM_001034194.1	EXOSC9	-2.224314	Homo sapiens exosome component 9 (EXOSC9), transcript variant 1, mRNA.
NM_019886.2	CHST7	-2.222375	Homo sapiens carbohydrate (N-acetylglucosamine 6-O) sulfotransferase 7 (CHST7), mRNA.
NM_058246.3	DNAJB6	-2.219849	Homo sapiens DnaJ (Hsp40) homolog, subfamily B, member 6 (DNAJB6), transcript variant 1, mRNA.
NM_018645.3	HES6	-2.217329	Homo sapiens hairy and enhancer of split 6 (Drosophila) (HES6), mRNA.
NM_024037.1	C1orf135	-2.216984	Homo sapiens chromosome 1 open reading frame 135 (C1orf135),

			mRNA.
NM_005189.1	CBX2	-2.210596	Homo sapiens chromobox homolog 2 (Pc class homolog, Drosophila) (CBX2), transcript variant 1, mRNA.
NM_000859.1	HMGCR	-2.209555	Homo sapiens 3-hydroxy-3-methylglutaryl-Coenzyme A reductase (HMGCR), mRNA.
NM_007152.2	ZNF195	-2.209382	Homo sapiens zinc finger protein 195 (ZNF195), mRNA.
NM_007280.1	OIP5	-2.207592	Homo sapiens Opa interacting protein 5 (OIP5), mRNA.
NM_004856.4	KIF23	-2.206272	Homo sapiens kinesin family member 23 (KIF23), transcript variant 2, mRNA.
NM_001018160.1	NAE1	-2.205923	Homo sapiens NEDD8 activating enzyme E1 subunit 1 (NAE1), transcript variant 3, mRNA.
NM_019042.3	PUS7	-2.204779	Homo sapiens pseudouridylate synthase 7 homolog (S. cerevisiae) (PUS7), mRNA.
NM_006892.3	DNMT3B	-2.20451	Homo sapiens DNA (cytosine-5-)-methyltransferase 3 beta (DNMT3B), transcript variant 1, mRNA.
NM_138285.3	NUP35	-2.200961	Homo sapiens nucleoporin 35kDa (NUP35), mRNA.
NM_001007157.1	PHF14	-2.198621	Homo sapiens PHD finger protein 14 (PHF14), transcript variant 1, mRNA.
NM_006950.3	SYN1	-2.198428	Homo sapiens synapsin I (SYN1), transcript variant Ia, mRNA.
NM_019106.4	SEPT3	-2.197992	Homo sapiens septin 3 (SEPT3), transcript variant B, mRNA.
XR_040993.1	LOC729082	-2.197198	PREDICTED: Homo sapiens misc_RNA (LOC729082), miscRNA.
NR_003105.1	ZWILCH	-2.196975	Homo sapiens Zwilch, kinetochore associated, homolog (Drosophila) (ZWILCH), transcript variant 2, transcribed RNA.
NM_024098.1	CCDC86	-2.195915	Homo sapiens coiled-coil domain containing 86 (CCDC86), mRNA.
NM_003012.3	SFRP1	-2.193358	Homo sapiens secreted frizzled-related protein 1 (SFRP1), mRNA.
NM_138792.2	LEO1	-2.189913	Homo sapiens Leo1, Paf1/RNA polymerase II complex component, homolog (S. cerevisiae) (LEO1), mRNA.
NM_001098525.1	CKAP2	-2.189321	Homo sapiens cytoskeleton associated protein 2 (CKAP2), transcript variant 2, mRNA.
NM_016299.2	HSPA14	-2.188773	Homo sapiens heat shock 70kDa protein 14 (HSPA14), transcript variant 1, mRNA.
NM_001168.2	BIRC5	-2.186204	Homo sapiens baculoviral IAP repeat-containing 5 (BIRC5), transcript variant 1, mRNA.
NM_014317.3	PDSS1	-2.185678	Homo sapiens prenyl (decaprenyl) diphosphate synthase, subunit 1 (PDSS1), mRNA.
NM_014431.1	KIAA1274	-2.185141	Homo sapiens KIAA1274 (KIAA1274), mRNA.
NM_018728.2	MYO5C	-2.184255	Homo sapiens myosin VC (MYO5C), mRNA.
NM_012083.2	FRAT2	-2.183249	Homo sapiens frequently rearranged in advanced T-cell lymphomas 2 (FRAT2), mRNA.
NM_002263.2	KIFC1	-2.182326	Homo sapiens kinesin family member C1 (KIFC1), mRNA.
XM_001125680.1	LOC730432	-2.182074	PREDICTED: Homo sapiens similar to serine/threonine/tyrosine interacting protein, transcript variant 1 (LOC730432), mRNA.
NM_020726.3	NLN	-2.18138	Homo sapiens neurolysin (metallopeptidase M3 family) (NLN), mRNA.
NM_015570.1	AUTS2	-2.180329	Homo sapiens autism susceptibility candidate 2 (AUTS2), mRNA.
NM_018353.3	C14orf106	-2.179813	Homo sapiens chromosome 14 open reading frame 106 (C14orf106), mRNA.
NM_015895.3	GMNN	-2.178742	Homo sapiens geminin, DNA replication inhibitor (GMNN), mRNA.
NM_012247.3	SEPHS1	-2.174957	Homo sapiens selenophosphate synthetase 1 (SEPHS1), mRNA.
NM_032776.1	JMJD1C	-2.173318	Homo sapiens jumonji domain containing 1C (JMJD1C), transcript variant 1, mRNA.
NM_012124.1	CHORDC1	-2.173167	Homo sapiens cysteine and histidine-rich domain (CHORD)-containing 1 (CHORDC1), mRNA.
NM_199420.3	POLQ	-2.17245	Homo sapiens polymerase (DNA directed), theta (POLQ), mRNA.
NM_017489.1	TERF1	-2.172419	Homo sapiens telomeric repeat binding factor (NIMA-interacting) 1 (TERF1), transcript variant 1, mRNA.
NM_006875.2	PIM2	-2.172344	Homo sapiens pim-2 oncogene (PIM2), mRNA.

NM_012482.3	ZNF281	-2.172209	Homo sapiens zinc finger protein 281 (ZNF281), mRNA.
NM_001002019.1	PUS1	-2.171728	Homo sapiens pseudouridylate synthase 1 (PUS1), transcript variant 2, mRNA.
NM_001025248.1	DUT	-2.171241	Homo sapiens deoxyuridine triphosphatase (DUT), nuclear gene encoding mitochondrial protein, transcript variant 1, mRNA.
NM_019030.2	DHX29	-2.169254	Homo sapiens DEAH (Asp-Glu-Ala-His) box polypeptide 29 (DHX29), mRNA.
NM_001040876.1	ABCE1	-2.154975	Homo sapiens ATP-binding cassette, sub-family E (OABP), member 1 (ABCE1), transcript variant 2, mRNA.
NM_030919.2	FAM83D	-2.151738	Homo sapiens family with sequence similarity 83, member D (FAM83D), mRNA.
NM_001010940.1	C9orf135	-2.151406	Homo sapiens chromosome 9 open reading frame 135 (C9orf135), mRNA.
NM_003390.2	WEE1	-2.149893	Homo sapiens WEE1 homolog (S. pombe) (WEE1), mRNA.
BC037864		-2.149693	Homo sapiens cDNA clone IMAGE:5272804
NM_016052.3	RRP15	-2.149159	Homo sapiens ribosomal RNA processing 15 homolog (S. cerevisiae) (RRP15), mRNA.
NM_003916.3	AP1S2	-2.148544	Homo sapiens adaptor-related protein complex 1, sigma 2 subunit (AP1S2), mRNA.
NM_003916.3	AP1S2	-2.147476	Homo sapiens adaptor-related protein complex 1, sigma 2 subunit (AP1S2), mRNA.
NM_032171.1	CEP78	-2.147266	Homo sapiens centrosomal protein 78kDa (CEP78), transcript variant 2, mRNA.
NM_003503.2	CDC7	-2.14692	Homo sapiens cell division cycle 7 homolog (S. cerevisiae) (CDC7), mRNA.
NR_002912.1	SNORA67	-2.146051	Homo sapiens small nucleolar RNA, H/ACA box 67 (SNORA67), small nucleolar RNA.
NM_001010879.2	ZIK1	-2.144061	Homo sapiens zinc finger protein interacting with K protein 1 homolog (mouse) (ZIK1), mRNA.
NM_021021.2	SNTB1	-2.141368	Homo sapiens syntrophin, beta 1 (dystrophin-associated protein A1, 59kDa, basic component 1) (SNTB1), mRNA.
NM_017412.2	FZD3	-2.138846	Homo sapiens frizzled homolog 3 (Drosophila) (FZD3), mRNA.
NM_004508.2	IDI1	-2.138552	Homo sapiens isopentenyl-diphosphate delta isomerase 1 (IDI1), mRNA.
BX537762		-2.13836	Homo sapiens mRNA; cDNA DKFZp779F0411 (from clone DKFZp779F0411)
NM_032860.3	LTV1	-2.137515	Homo sapiens LTV1 homolog (S. cerevisiae) (LTV1), mRNA.
NM_006617.1	NES	-2.134659	Homo sapiens nestin (NES), mRNA.
NM_001034194.1	EXOSC9	-2.134024	Homo sapiens exosome component 9 (EXOSC9), transcript variant 1, mRNA.
NM_001077440.1	BCLAF1	-2.132395	Homo sapiens BCL2-associated transcription factor 1 (BCLAF1), transcript variant 2, mRNA.
NM_001042610.1	DBNDD1	-2.130069	Homo sapiens dysbindin (dystrobrevin binding protein 1) domain containing 1 (DBNDD1), transcript variant 1, mRNA.
NM_002830.2	PTPN4	-2.12995	Homo sapiens protein tyrosine phosphatase, non-receptor type 4 (megakaryocyte) (PTPN4), mRNA.
NM_145792.1	MGST1	-2.127705	Homo sapiens microsomal glutathione S-transferase 1 (MGST1), transcript variant 1a, mRNA.
NM_006197.2	PCM1	-2.123255	Homo sapiens pericentriolar material 1 (PCM1), mRNA.
NM_032471.4	PKIB	-2.123032	Homo sapiens protein kinase (cAMP-dependent, catalytic) inhibitor beta (PKIB), transcript variant 3, mRNA.
NM_006479.3	RAD51AP1	-2.122621	Homo sapiens RAD51 associated protein 1 (RAD51AP1), mRNA.
NM_001002259.1	CAPRIN2	-2.120813	Homo sapiens caprin family member 2 (CAPRIN2), transcript variant 1, mRNA.
NM_006745.3	SC4MOL	-2.120452	Homo sapiens sterol-C4-methyl oxidase-like (SC4MOL), transcript variant 1, mRNA.
NM_020685.3	C3orf14	-2.12009	Homo sapiens chromosome 3 open reading frame 14 (C3orf14), mRNA.

NM_012250.3	RRAS2	-2.118279	Homo sapiens related RAS viral (r-ras) oncogene homolog 2 (RRAS2), mRNA.
NM_018846.2	KLHL7	-2.116314	Homo sapiens kelch-like 7 (Drosophila) (KLHL7), transcript variant 2, mRNA.
NM_002703.3	PPAT	-2.116258	Homo sapiens phosphoribosyl pyrophosphate amidotransferase (PPAT), mRNA.
NM_003276.1	TMPO	-2.113373	Homo sapiens thymopoietin (TMPO), transcript variant 1, mRNA.
NM_024595.1	AKIRIN1	-2.11228	Homo sapiens akirin 1 (AKIRIN1), mRNA.
NM_004321.4	KIF1A	-2.111551	Homo sapiens kinesin family member 1A (KIF1A), mRNA.
NM_006802.2	SF3A3	-2.109976	Homo sapiens splicing factor 3a, subunit 3, 60kDa (SF3A3), mRNA.
NM_004792.2	PPIG	-2.106757	Homo sapiens peptidylprolyl isomerase G (cyclophilin G) (PPIG), mRNA.
NM_001099666.1	PTAR1	-2.104933	Homo sapiens protein prenyltransferase alpha subunit repeat containing 1 (PTAR1), mRNA.
NM_023925.3	CAPRIN2	-2.101634	Homo sapiens caprin family member 2 (CAPRIN2), transcript variant 2, mRNA.
NM_017760.5	NCAPG2	-2.100361	Homo sapiens non-SMC condensin II complex, subunit G2 (NCAPG2), mRNA.
NM_032168.1	WDR75	-2.097438	Homo sapiens WD repeat domain 75 (WDR75), mRNA.
NM_001025780.1	FAM108B1	-2.097015	Homo sapiens family with sequence similarity 108, member B1 (FAM108B1), transcript variant 2, mRNA.
XR_015176.2	TYW1B	-2.096112	PREDICTED: Homo sapiens misc_RNA (TYW1B), miscRNA.
NM_198686.1	RAB15	-2.095562	Homo sapiens RAB15, member RAS onocogene family (RAB15), mRNA.
NM_004671.2	PIAS2	-2.093755	Homo sapiens protein inhibitor of activated STAT, 2 (PIAS2), transcript variant beta, mRNA.
NM_018387.2	STRBP	-2.093214	Homo sapiens spermatid perinuclear RNA binding protein (STRBP), mRNA.
NM_018079.3	SRBD1	-2.090884	Homo sapiens S1 RNA binding domain 1 (SRBD1), mRNA.
NM_005342.2	HMGB3	-2.089198	Homo sapiens high-mobility group box 3 (HMGB3), mRNA.
NM_024755.2	SLTM	-2.08784	Homo sapiens SAFB-like, transcription modulator (SLTM), transcript variant 1, mRNA.
NM_005381.2	NCL	-2.084943	Homo sapiens nucleolin (NCL), mRNA.
NM_000946.2	PRIM1	-2.08198	Homo sapiens primase, DNA, polypeptide 1 (49kDa) (PRIM1), mRNA.
XM_001723047.1	LOC100132901	-2.081865	PREDICTED: Homo sapiens similar to KIAA1874 protein (LOC100132901), mRNA.
NM_004280.3	EEF1E1	-2.080595	Homo sapiens eukaryotic translation elongation factor 1 epsilon 1 (EEF1E1), mRNA.
NM_015187.3	SEL1L3	-2.076223	Homo sapiens sel-1 suppressor of lin-12-like 3 (C. elegans) (SEL1L3), mRNA.
NM_022346.3	NCAPG	-2.075397	Homo sapiens non-SMC condensin I complex, subunit G (NCAPG), mRNA.
NM_018169.2	C12orf35	-2.075071	Homo sapiens chromosome 12 open reading frame 35 (C12orf35), mRNA.
NM_003333.3	UBA52	-2.071065	Homo sapiens ubiquitin A-52 residue ribosomal protein fusion product 1 (UBA52), transcript variant 2, mRNA.
NM_024945.1	RMI1	-2.068026	Homo sapiens RMI1, RecQ mediated genome instability 1, homolog (S. cerevisiae) (RMI1), mRNA.
NM_022805.2	SNRPN	-2.065371	Homo sapiens small nuclear ribonucleoprotein polypeptide N (SNRPN), transcript variant 2, mRNA.
NR_001561.1	CYCSL1	-2.065085	Homo sapiens cytochrome c, somatic-like 1 (CYCSL1) on chromosome 6.
NM_022074.2	FAM111A	-2.062076	Homo sapiens family with sequence similarity 111, member A (FAM111A), transcript variant 1, mRNA.
XR_017449.2	LOC647150	-2.059644	PREDICTED: Homo sapiens misc_RNA (LOC647150), miscRNA.
NM_005914.2	MCM4	-2.053223	Homo sapiens minichromosome maintenance complex component 4 (MCM4), transcript variant 1, mRNA.
NM_138421.2	SAAL1	-2.049945	Homo sapiens serum amyloid A-like 1 (SAAL1), mRNA.
NM_006729.3	DIAPH2	-2.048642	Homo sapiens diaphanous homolog 2 (Drosophila) (DIAPH2), transcript

			variant 156, mRNA.
NM_002692.2	POLE2	-2.047799	Homo sapiens polymerase (DNA directed), epsilon 2 (p59 subunit) (POLE2), mRNA.
NM_020401.2	NUP107	-2.043124	Homo sapiens nucleoporin 107kDa (NUP107), mRNA.
NM_001384.4	DPH2	-2.042606	Homo sapiens DPH2 homolog (S. cerevisiae) (DPH2), transcript variant 1, mRNA.
NM_024032.2	C17orf53	-2.042441	Homo sapiens chromosome 17 open reading frame 53 (C17orf53), mRNA.
NM_017736.3	THUMPD1	-2.042321	Homo sapiens THUMP domain containing 1 (THUMPD1), mRNA.
NM_020236.2	MRPL1	-2.04232	Homo sapiens mitochondrial ribosomal protein L1 (MRPL1), nuclear gene encoding mitochondrial protein, mRNA.
NM_000553.3	WRN	-2.041627	Homo sapiens Werner syndrome (WRN), mRNA.
NM_001018160.1	NAE1	-2.040359	Homo sapiens NEDD8 activating enzyme E1 subunit 1 (NAE1), transcript variant 3, mRNA.
NM_017892.3	PRPF40A	-2.039571	Homo sapiens PRP40 pre-mRNA processing factor 40 homolog A (S. cerevisiae) (PRPF40A), mRNA.
NM_032991.2	CASP3	-2.037361	Homo sapiens caspase 3, apoptosis-related cysteine peptidase (CASP3), transcript variant beta, mRNA.
NM_001040876.1	ABCE1	-2.035343	Homo sapiens ATP-binding cassette, sub-family E (OABP), member 1 (ABCE1), transcript variant 2, mRNA.
NM_080820.4	DTD1	-2.034862	Homo sapiens D-tyrosyl-tRNA deacylase 1 homolog (S. cerevisiae) (DTD1), nuclear gene encoding mitochondrial protein, mRNA.
NM_005378.4	MYCN	-2.03264	Homo sapiens v-myc myelocytomatosis viral related oncogene, neuroblastoma derived (avian) (MYCN), mRNA.
NM_003129.3	SQLE	-2.030881	Homo sapiens squalene epoxidase (SQLE), mRNA.
NM_016447.2	MPP6	-2.030018	Homo sapiens membrane protein, palmitoylated 6 (MAGUK p55 subfamily member 6) (MPP6), mRNA.
NM_148174.2	AZIN1	-2.01757	Homo sapiens antizyme inhibitor 1 (AZIN1), transcript variant 2, mRNA.
NM_014412.2	CACYBP	-2.014085	Homo sapiens calcyclin binding protein (CACYBP), transcript variant 1, mRNA.
XR_037888.1	LOC100128266	-2.013594	PREDICTED: Homo sapiens misc_RNA (LOC100128266), miscRNA.
NM_001823.3	CKB	-2.012195	Homo sapiens creatine kinase, brain (CKB), mRNA.
NM_006892.3	DNMT3B	-2.00701	Homo sapiens DNA (cytosine-5-)-methyltransferase 3 beta (DNMT3B), transcript variant 1, mRNA.
NM_004111.4	FEN1	-2.006428	Homo sapiens flap structure-specific endonuclease 1 (FEN1), mRNA.

Supplementary table 5: Genes up regulated in Day 20 as compared to Day 0

Refseq_NM	Gene Symbol	D20/D12.fc	DEFINITION
NM_133505.2	DCN	162.804	Homo sapiens decorin (DCN), transcript variant C, mRNA.
NM_004821.1	HAND1	139.821	Homo sapiens heart and neural crest derivatives expressed 1 (HAND1), mRNA.
NM_000090.3	COL3A1	87.871	Homo sapiens collagen, type III, alpha 1 (COL3A1), mRNA.
NR_002196.1	H19	85.521	Homo sapiens H19, imprinted maternally expressed transcript (non-protein coding) (H19), non-coding RNA.
NM_024626.2	VTCN1	82.579	Homo sapiens V-set domain containing T cell activation inhibitor 1 (VTCN1), mRNA.
NM_014211.1	GABRP	81.411	Homo sapiens gamma-aminobutyric acid (GABA) A receptor, pi (GABRP), mRNA.
NM_001430.3	EPAS1	70.053	Homo sapiens endothelial PAS domain protein 1 (EPAS1), mRNA.
NM_057165.2	COL6A3	66.164	Homo sapiens collagen, type VI, alpha 3 (COL6A3), transcript variant 3, mRNA.
NM_004369.2	COL6A3	63.564	Homo sapiens collagen, type VI, alpha 3 (COL6A3), transcript variant 1, mRNA.
NM_002345.3	LUM	59.950	Homo sapiens lumican (LUM), mRNA.
NM_173054.1	RELN	47.025	Homo sapiens reelin (RELN), transcript variant 2, mRNA.
NM_003063.2	SLN	46.604	Homo sapiens sarcolipin (SLN), mRNA.
NM_001042425.1	TFAP2A	43.285	Homo sapiens transcription factor AP-2 alpha (activating enhancer binding protein 2 alpha) (TFAP2A), transcript variant 3, mRNA.
NM_005613.3	RGS4	42.137	Homo sapiens regulator of G-protein signalling 4 (RGS4), mRNA.
NM_000093.3	COL5A1	40.979	Homo sapiens collagen, type V, alpha 1 (COL5A1), mRNA.
NM_000598.4	IGFBP3	40.190	Homo sapiens insulin-like growth factor binding protein 3 (IGFBP3), transcript variant 2, mRNA.
NM_052947.3	ALPK2	39.904	Homo sapiens alpha-kinase 2 (ALPK2), mRNA.
NM_001013398.1	IGFBP3	38.739	Homo sapiens insulin-like growth factor binding protein 3 (IGFBP3), transcript variant 1, mRNA.
NM_004613.2	TGM2	36.125	Homo sapiens transglutaminase 2 (C polypeptide, protein-glutamine-gamma-glutamyltransferase) (TGM2), transcript variant 1, mRNA.
NM_003068.3	SNAI2	35.599	Homo sapiens snail homolog 2 (Drosophila) (SNAI2), mRNA.
NM_002404.1	MFAP4	33.801	Homo sapiens microfibrillar-associated protein 4 (MFAP4), mRNA.
NM_002145.3	HOXB2	33.640	Homo sapiens homeobox B2 (HOXB2), mRNA.
NM_014585.3	SLC40A1	32.469	Homo sapiens solute carrier family 40 (iron-regulated transporter), member 1 (SLC40A1), mRNA.
NM_001855.3	COL15A1	31.048	Homo sapiens collagen, type XV, alpha 1 (COL15A1), mRNA.
NM_003836.4	DLK1	30.983	Homo sapiens delta-like 1 homolog (Drosophila) (DLK1), mRNA.
NM_182920.1	ADAMTS9	30.766	Homo sapiens ADAM metallopeptidase with thrombospondin type 1 motif, 9 (ADAMTS9), mRNA.
NM_000856.3	GUCY1A3	30.608	Homo sapiens guanylate cyclase 1, soluble, alpha 3 (GUCY1A3), mRNA.
NM_002160.2	TNC	29.931	Homo sapiens tenascin C (TNC), mRNA.
NM_172315.1	MEIS2	29.870	Homo sapiens Meis homeobox 2 (MEIS2), transcript variant g, mRNA.
NM_001007139.3	IGF2	29.801	Homo sapiens insulin-like growth factor 2 (somatomedin A) (IGF2), transcript variant 2, mRNA.
NM_000599.2	IGFBP5	29.482	Homo sapiens insulin-like growth factor binding protein 5 (IGFBP5), mRNA.
NM_178565.3	RSPO2	29.278	Homo sapiens R-spondin 2 homolog (Xenopus laevis) (RSPO2), mRNA.
NM_015507.2	EGFL6	26.999	Homo sapiens EGF-like-domain, multiple 6 (EGFL6), mRNA.
NM_000599.2	IGFBP5	26.890	Homo sapiens insulin-like growth factor binding protein 5 [illegible]
NM_003062.1	SLIT3	26.070	Homo sapiens slit homolog 3 (Drosophila) (SLIT3), mRNA.

NM_000900.2	MGP	23.730	Homo sapiens matrix Gla protein (MGP), mRNA.
NM_021005.2	NR2F2	23.443	Homo sapiens nuclear receptor subfamily 2, group F, member 2 (NR2F2), mRNA.
NM_153321.1	PMP22	23.305	Homo sapiens peripheral myelin protein 22 (PMP22), transcript variant 2, mRNA.
NM_000612.2	IGF2	22.730	Homo sapiens insulin-like growth factor 2 (somatomedin A) (IGF2), mRNA.
NM_002448.3	MSX1	22.710	Homo sapiens msh homeobox 1 (MSX1), mRNA.
NM_203411.1	TMEM88	21.612	Homo sapiens transmembrane protein 88 (TMEM88), mRNA.
NM_004994.2	MMP9	21.467	Homo sapiens matrix metallopeptidase 9 (gelatinase B, 92kDa gelatinase, 92kDa type IV collagenase) (MMP9), mRNA.
NM_000039.1	APOA1	20.968	Homo sapiens apolipoprotein A-I (APOA1), mRNA.
XM_001130278.1	MAB21L2	20.859	PREDICTED: Homo sapiens mab-21-like 2 (C. elegans) (MAB21L2), mRNA.
NM_003873.4	NRP1	20.568	Homo sapiens neuropilin 1 (NRP1), transcript variant 1, mRNA.
NM_015419.2	MXRA5	20.373	Homo sapiens matrix-remodelling associated 5 (MXRA5), mRNA.
NM_184085.1	TRIM55	20.218	Homo sapiens tripartite motif-containing 55 (TRIM55), transcript variant 1, mRNA.
NM_024911.4	GPR177	20.020	Homo sapiens G protein-coupled receptor 177 (GPR177), transcript variant 1, mRNA.
NM_184086.1	TRIM55	19.834	Homo sapiens tripartite motif-containing 55 (TRIM55), transcript variant 3, mRNA.
NM_000089.3	COL1A2	19.678	Homo sapiens collagen, type I, alpha 2 (COL1A2), mRNA.
NM_000089.3	COL1A2	19.404	Homo sapiens collagen, type I, alpha 2 (COL1A2), mRNA.
XM_378360		19.389	PREDICTED: Homo sapiens hypothetical LOC400043 (LOC400043), mRNA
NM_005161.3	APLNR	19.317	Homo sapiens apelin receptor (APLNR), mRNA.
NM_031476.2	CRISPLD2	19.231	Homo sapiens cysteine-rich secretory protein LCCL domain containing 2 (CRISPLD2), mRNA.
NM_133265.2	AMOT	17.903	Homo sapiens angiomotin (AMOT), transcript variant 2, mRNA.
NM_153366.2	SVEP1	17.403	Homo sapiens sushi, von Willebrand factor type A, EGF and pentraxin domain containing 1 (SVEP1), mRNA.
NM_015507.2	EGFL6	17.205	Homo sapiens EGF-like-domain, multiple 6 (EGFL6), mRNA.
NM_198552.1	FAM89A	17.132	Homo sapiens family with sequence similarity 89, member A (FAM89A), mRNA.
NM_001553.1	IGFBP7	16.959	Homo sapiens insulin-like growth factor binding protein 7 (IGFBP7), mRNA.
NM_021965.3	PGM5	16.935	Homo sapiens phosphoglucomutase 5 (PGM5), mRNA.
NM_005630.1	SLCO2A1	16.422	Homo sapiens solute carrier organic anion transporter family, member 2A1 (SLCO2A1), mRNA.
NM_181712.3	ANKRD38	16.165	Homo sapiens ankyrin repeat domain 38 (ANKRD38), mRNA.
XM_001126087.1	LOC728473	16.127	PREDICTED: Homo sapiens hypothetical LOC728473 (LOC728473), mRNA.
NM_001001936.1	AFAP1L2	16.125	Homo sapiens actin filament associated protein 1-like 2 (AFAP1L2), transcript variant 1, mRNA.
NM_181712.4	KANK4	16.064	Homo sapiens KN motif and ankyrin repeat domains 4 (KANK4), mRNA.
NM_001037582.1	SCD5	15.892	Homo sapiens stearoyl-CoA desaturase 5 (SCD5), transcript variant 1, mRNA.
NM_005045.2	RELN	15.779	Homo sapiens reelin (RELN), transcript variant 1, mRNA.
NM_130386.1	COLEC12	15.631	Homo sapiens collectin sub-family member 12 (COLEC12), mRNA.
NM_032777.6	GPR124	15.619	Homo sapiens G protein-coupled receptor 124 (GPR124), mRNA.
NM_001048.3	SST	15.576	Homo sapiens somatostatin (SST), mRNA.
NM_021965.3	PGM5	15.564	Homo sapiens phosphoglucomutase 5 (PGM5), mRNA.
NM_139212.2	HOPX	15.380	Homo sapiens HOP homeobox (HOPX), transcript variant 3, mRNA.
NM_002202.1	ISL1	15.240	Homo sapiens ISL1 transcription factor, LIM/homeodomain, (islet-

			1) (ISL1), mRNA.
NM_004887.3	CXCL14	15.145	Homo sapiens chemokine (C-X-C motif) ligand 14 (CXCL14), mRNA.
NM_025140.1	CCDC92	15.031	Homo sapiens coiled-coil domain containing 92 (CCDC92), mRNA.
NM_002476.2	MYL4	15.022	Homo sapiens myosin, light chain 4, alkali; atrial, embryonic (MYL4), transcript variant 2, mRNA.
NM_133337.1	FER1L3	14.677	Homo sapiens fer-1-like 3, myoferlin (C. elegans) (FER1L3), transcript variant 2, mRNA.
NM_001012964.1	KLK6	14.360	Homo sapiens kallikrein-related peptidase 6 (KLK6), transcript variant B, mRNA.
NM_002345.3	LUM	14.100	Homo sapiens lumican (LUM), mRNA.
NM_013451.3	MYOF	13.938	Homo sapiens myoferlin (MYOF), transcript variant 1, mRNA.
NM_013231.4	FLRT2	13.735	Homo sapiens fibronectin leucine rich transmembrane protein 2 (FLRT2), mRNA.
NM_004120.3	GBP2	13.548	Homo sapiens guanylate binding protein 2, interferon-inducible (GBP2), mRNA.
XR_038625.1	LOC100132535	13.311	PREDICTED: Homo sapiens misc_RNA (LOC100132535), miscRNA.
NM_153427.1	PITX2	13.280	Homo sapiens paired-like homeodomain 2 (PITX2), transcript variant 1, mRNA.
NM_001643.1	APOA2	13.242	Homo sapiens apolipoprotein A-II (APOA2), mRNA.
NM_001002292.1	GPR177	13.145	Homo sapiens G protein-coupled receptor 177 (GPR177), transcript variant 2, mRNA.
NM_001017973.1	P4HA2	12.969	Homo sapiens prolyl 4-hydroxylase, alpha polypeptide II (P4HA2), transcript variant 2, mRNA.
NM_000389.2	CDKN1A	12.900	Homo sapiens cyclin-dependent kinase inhibitor 1A (p21, Cip1) (CDKN1A), transcript variant 1, mRNA.
NM_032413.2	C15orf48	12.897	Homo sapiens chromosome 15 open reading frame 48 (C15orf48), transcript variant 2, mRNA.
NM_001032280.2	TFAP2A	12.842	Homo sapiens transcription factor AP-2 alpha (activating enhancer binding protein 2 alpha) (TFAP2A), transcript variant 2, mRNA.
NM_006727.2	CDH10	12.682	Homo sapiens cadherin 10, type 2 (T2-cadherin) (CDH10), mRNA.
NM_033255.2	EPSTI1	12.558	Homo sapiens epithelial stromal interaction 1 (breast) (EPSTI1), transcript variant 2, mRNA.
NM_014899.3	RHOBTB3	12.533	Homo sapiens Rho-related BTB domain containing 3 (RHOBTB3), mRNA.
NM_024574.3	C4orf31	12.202	Homo sapiens chromosome 4 open reading frame 31 (C4orf31), mRNA.
NM_000362.4	TIMP3	12.094	Homo sapiens TIMP metallopeptidase inhibitor 3 (TIMP3), mRNA.
NM_001630.1	ANXA8	12.061	Homo sapiens annexin A8 (ANXA8), mRNA.
NM_000088.3	COL1A1	11.801	Homo sapiens collagen, type I, alpha 1 (COL1A1), mRNA.
NM_031910.3	C1QTNF6	11.786	Homo sapiens C1q and tumor necrosis factor related protein 6 (C1QTNF6), transcript variant 1, mRNA.
NM_007361.3	NID2	11.751	Homo sapiens nidogen 2 (osteonidogen) (NID2), mRNA.
NM_004207.2	SLC16A3	11.642	Homo sapiens solute carrier family 16, member 3 (monocarboxylic acid transporter 4) (SLC16A3), transcript variant 2, mRNA.
NM_000963.1	PTGS2	11.568	Homo sapiens prostaglandin-endoperoxide synthase 2 (prostaglandin G/H synthase and cyclooxygenase) (PTGS2), mRNA.
NM_152611.2	C20orf75	11.505	Homo sapiens chromosome 20 open reading frame 75 (C20orf75), mRNA.
NM_032550.2	AFAP1L2	11.431	Homo sapiens actin filament associated protein 1-like 2 (AFAP1L2), transcript variant 2, mRNA.
NM_206966.2	C5orf46	11.356	Homo sapiens chromosome 5 open reading frame 46 (C5orf46), mRNA.
NM_018689.1	KIAA1199	11.215	Homo sapiens KIAA1199 (KIAA1199), mRNA.

NM_002353.1	TACSTD2	11.057	Homo sapiens tumor-associated calcium signal transducer 2 (TACSTD2), mRNA.
AB074172		10.923	Homo sapiens primary neuroblastoma cDNA, clone:Nbla10111, full insert sequence
NM_201525.1	GPR56	10.861	Homo sapiens G protein-coupled receptor 56 (GPR56), transcript variant 3, mRNA.
NM_005512.1	LRRC32	10.792	Homo sapiens leucine rich repeat containing 32 (LRRC32), mRNA.
XM_939093.1	FAM89A	10.578	PREDICTED: Homo sapiens family with sequence similarity 89, member A (FAM89A), mRNA.
NM_006472.2	TXNIP	10.531	Homo sapiens thioredoxin interacting protein (TXNIP), mRNA.
NM_000316.2	PTH1R	10.520	Homo sapiens parathyroid hormone 1 receptor (PTH1R), mRNA.
NM_021205.4	RHOU	10.370	Homo sapiens ras homolog gene family, member U (RHOU), mRNA.
NM_001032278.1	MMP28	10.274	Homo sapiens matrix metallopeptidase 28 (MMP28), transcript variant 3, mRNA.
NM_133468.3	BMPER	10.273	Homo sapiens BMP binding endothelial regulator (BMPER), mRNA.
NM_002317.3	LOX	10.257	Homo sapiens lysyl oxidase (LOX), mRNA.
NM_003966.2	SEMA5A	10.006	Homo sapiens sema domain, seven thrombospondin repeats (type 1 and type 1-like), transmembrane domain (TM) and short cytoplasmic domain, (semaphorin) 5A (SEMA5A), mRNA.
NM_032784.3	RSPO3	9.974	Homo sapiens R-spondin 3 homolog (Xenopus laevis) (RSPO3), mRNA.
NM_022873.2	IFI6	9.844	Homo sapiens interferon, alpha-inducible protein 6 (IFI6), transcript variant 3, mRNA.
NM_000371.1	TTR	9.805	Homo sapiens transthyretin (prealbumin, amyloidosis type I) (TTR), mRNA.
NM_001453.1	FOXC1	9.793	Homo sapiens forkhead box C1 (FOXC1), mRNA.
NM_003118.2	SPARC	9.732	Homo sapiens secreted protein, acidic, cysteine-rich (osteonectin) (SPARC), mRNA.
NM_005195.3	CEBPD	9.726	Homo sapiens CCAAT/enhancer binding protein (C/EBP), delta (CEBPD), mRNA.
NM_016613.5	C4orf18	9.701	Homo sapiens chromosome 4 open reading frame 18 (C4orf18), transcript variant 2, mRNA.
NM_001040708.1	HEY1	9.659	Homo sapiens hairy/enhancer-of-split related with YRPW motif 1 (HEY1), transcript variant 2, mRNA.
NM_007173.4	PRSS23	9.601	Homo sapiens protease, serine, 23 (PRSS23), mRNA.
NM_014890.2	FILIP1L	9.581	Homo sapiens filamin A interacting protein 1-like (FILIP1L), transcript variant 2, mRNA.
NM_182799.1	EGFLAM	9.558	Homo sapiens EGF-like, fibronectin type III and laminin G domains (EGFLAM), transcript variant 3, mRNA.
NM_000053.2	ATP7B	9.507	Homo sapiens ATPase, Cu++ transporting, beta polypeptide (ATP7B), transcript variant 1, mRNA.
NM_000393.3	COL5A2	9.429	Homo sapiens collagen, type V, alpha 2 (COL5A2), mRNA.
NM_002581.3	PAPPA	9.425	Homo sapiens pregnancy-associated plasma protein A, pappalysin 1 (PAPPA), mRNA.
NM_000638.3	VTN	9.295	Homo sapiens vitronectin (VTN), mRNA.
NM_002609.3	PDGFRB	9.294	Homo sapiens platelet-derived growth factor receptor, beta polypeptide (PDGFRB), mRNA.
NM_014579.1	SLC39A2	9.286	Homo sapiens solute carrier family 39 (zinc transporter), member 2 (SLC39A2), mRNA.
NM_001098816.2	ODZ4	9.208	Homo sapiens odz, odd Oz/ten-m homolog 4 (Drosophila) (ODZ4), mRNA.
NM_004503.3	HOXC6	9.095	Homo sapiens homeobox C6 (HOXC6), transcript variant 1, mRNA.
NM_001920.3	DCN	9.022	Homo sapiens decorin (DCN), transcript variant A1, mRNA.
NM_013451.2	FER1L3	9.014	Homo sapiens fer-1-like 3, myoferlin (C. elegans) (FER1L3), transcript variant 1, mRNA.

NM_018993.2	RIN2	8.961	Homo sapiens Ras and Rab interactor 2 (RIN2), mRNA.
NM_020547.1	AMHR2	8.919	Homo sapiens anti-Mullerian hormone receptor, type II (AMHR2), mRNA.
NM_032772.3	ZNF503	8.919	Homo sapiens zinc finger protein 503 (ZNF503), mRNA.
NM_145057.2	CDC42EP5	8.903	Homo sapiens CDC42 effector protein (Rho GTPase binding) 5 (CDC42EP5), mRNA.
NM_001017974.1	P4HA2	8.903	Homo sapiens prolyl 4-hydroxylase, alpha polypeptide II (P4HA2), transcript variant 3, mRNA.
NM_138444.3	KCTD12	8.850	Homo sapiens potassium channel tetramerisation domain containing 12 (KCTD12), mRNA.
NM_001323.2	CST6	8.792	Homo sapiens cystatin E/M (CST6), mRNA.
NM_018670.2	MESP1	8.779	Homo sapiens mesoderm posterior 1 homolog (mouse) (MESP1), mRNA.
NM_144966.4	FREM1	8.776	Homo sapiens FRAS1 related extracellular matrix 1 (FREM1), mRNA.
NM_145260.2	OSR1	8.743	Homo sapiens odd-skipped related 1 (Drosophila) (OSR1), mRNA.
NM_001620.1	AHNAK	8.739	Homo sapiens AHNAK nucleoprotein (AHNAK), transcript variant 1, mRNA.
NM_019885.2	CYP26B1	8.721	Homo sapiens cytochrome P450, family 26, subfamily B, polypeptide 1 (CYP26B1), mRNA.
NM_078487.2	CDKN2B	8.429	Homo sapiens cyclin-dependent kinase inhibitor 2B (p15, inhibits CDK4) (CDKN2B), transcript variant 2, mRNA.
NM_001884.2	HAPLN1	8.395	Homo sapiens hyaluronan and proteoglycan link protein 1 (HAPLN1), mRNA.
NM_022658.3	HOXC8	8.394	Homo sapiens homeobox C8 (HOXC8), mRNA.
NM_020311.2	CXCR7	8.386	Homo sapiens chemokine (C-X-C motif) receptor 7 (CXCR7), mRNA.
NM_022131.1	CLSTN2	8.371	Homo sapiens calsyntenin 2 (CLSTN2), mRNA.
NM_012342.2	BAMBI	8.368	Homo sapiens BMP and activin membrane-bound inhibitor homolog (Xenopus laevis) (BAMBI), mRNA.
NM_201524.1	GPR56	8.311	Homo sapiens G protein-coupled receptor 56 (GPR56), transcript variant 2, mRNA.
NM_006475.1	POSTN	8.272	Homo sapiens periostin, osteoblast specific factor (POSTN), mRNA.
NM_001898.2	CST1	8.056	Homo sapiens cystatin SN (CST1), mRNA.
NM_006108.2	SPON1	8.056	Homo sapiens spondin 1, extracellular matrix protein (SPON1), mRNA.
NM_002449.4	MSX2	8.000	Homo sapiens msh homeobox 2 (MSX2), mRNA.
NM_016269.2	LEF1	7.923	Homo sapiens lymphoid enhancer-binding factor 1 (LEF1), mRNA.
BC062365		7.882	Homo sapiens cDNA clone IMAGE:5922621, partial cds
NM_080645.2	COL12A1	7.855	Homo sapiens collagen, type XII, alpha 1 (COL12A1), transcript variant short, mRNA.
NM_003392.3	WNT5A	7.773	Homo sapiens wingless-type MMTV integration site family, member 5A (WNT5A), mRNA.
NM_024911.4	GPR177	7.729	Homo sapiens G protein-coupled receptor 177 (GPR177), transcript variant 1, mRNA.
NM_002026.2	FN1	7.659	Homo sapiens fibronectin 1 (FN1), transcript variant 3, mRNA.
NM_001711.3	BGN	7.617	Homo sapiens biglycan (BGN), mRNA.
NM_207336.1	ZNF467	7.574	Homo sapiens zinc finger protein 467 (ZNF467), mRNA.
NM_015036.1	ENDOD1	7.567	Homo sapiens endonuclease domain containing 1 (ENDOD1), mRNA.
NM_000700.1	ANXA1	7.559	Homo sapiens annexin A1 (ANXA1), mRNA.
NM_003250.4	THRA	7.482	Homo sapiens thyroid hormone receptor, alpha (erythroblastic leukemia viral (v-erb-a) oncogene homolog, avian) (THRA), transcript variant 2, mRNA.
NM_024519.2	FAM65A	7.473	Homo sapiens family with sequence similarity 65, member A (FAM65A), mRNA.

NM_003979.3	GPRC5A	7.302	Homo sapiens G protein-coupled receptor, family C, group 5, member A (GPRC5A), mRNA.
NM_198391.1	FLRT3	7.263	Homo sapiens fibronectin leucine rich transmembrane protein 3 (FLRT3), transcript variant 2, mRNA.
NM_004787.1	SLIT2	7.252	Homo sapiens slit homolog 2 (Drosophila) (SLIT2), mRNA.
NM_021965.3	PGM5	7.171	Homo sapiens phosphoglucomutase 5 (PGM5), mRNA.
NM_000856.2	GUCY1A3	7.152	Homo sapiens guanylate cyclase 1, soluble, alpha 3 (GUCY1A3), mRNA.
NM_003206.2	TCF21	7.119	Homo sapiens transcription factor 21 (TCF21), transcript variant 2, mRNA.
NM_002048.1	GAS1	7.109	Homo sapiens growth arrest-specific 1 (GAS1), mRNA.
NM_032181.1	TMEM166	7.059	Homo sapiens transmembrane protein 166 (TMEM166), mRNA.
NM_000905.2	NPY	7.058	Homo sapiens neuropeptide Y (NPY), mRNA.
NM_005585.3	SMAD6	7.045	Homo sapiens SMAD family member 6 (SMAD6), transcript variant 1, mRNA.
NM_020877.2	DNAH2	7.035	Homo sapiens dynein, axonemal, heavy chain 2 (DNAH2), mRNA.
NM_001124758.1	SPNS2	7.033	Homo sapiens spinster homolog 2 (Drosophila) (SPNS2), mRNA.
NM_130851.1	BMP4	6.955	Homo sapiens bone morphogenetic protein 4 (BMP4), transcript variant 3, mRNA.
XM_001722168.1	LOC100128893	6.936	PREDICTED: Homo sapiens hypothetical protein LOC100128893 (LOC100128893), mRNA.
NM_006206.3	PDGFRA	6.913	Homo sapiens platelet-derived growth factor receptor, alpha polypeptide (PDGFRA), mRNA.
NR_024430.1	LOC399959	6.901	Homo sapiens hypothetical LOC399959 (LOC399959), non-coding RNA.
NM_006287.4	TFPI	6.893	Homo sapiens tissue factor pathway inhibitor (lipoprotein-associated coagulation inhibitor) (TFPI), transcript variant 1, mRNA.
NM_002398.2	MEIS1	6.856	Homo sapiens Meis homeobox 1 (MEIS1), mRNA.
NM_001007097.1	NTRK2	6.853	Homo sapiens neurotrophic tyrosine kinase, receptor, type 2 (NTRK2), transcript variant b, mRNA.
XM_001129442.1	CST6	6.849	PREDICTED: Homo sapiens cystatin E/M (CST6), mRNA.
NM_001042377.1	INS-IGF2	6.821	Homo sapiens insulin- insulin-like growth factor 2 (INS-IGF2), transcript variant 1, mRNA.
NM_207304.1	MBNL2	6.814	Homo sapiens muscleblind-like 2 (Drosophila) (MBNL2), transcript variant 3, mRNA.
NM_018110.2	DOK4	6.723	Homo sapiens docking protein 4 (DOK4), mRNA.
NM_003243.2	TGFBR3	6.705	Homo sapiens transforming growth factor, beta receptor III (TGFBR3), mRNA.
NM_002023.3	FMOD	6.644	Homo sapiens fibromodulin (FMOD), mRNA.
NM_001001391.1	CD44	6.624	Homo sapiens CD44 molecule (Indian blood group) (CD44), transcript variant 4, mRNA.
NM_016352.2	CPA4	6.545	Homo sapiens carboxypeptidase A4 (CPA4), mRNA.
NM_005328.1	HAS2	6.490	Homo sapiens hyaluronan synthase 2 (HAS2), mRNA.
NM_012242.2	DKK1	6.475	Homo sapiens dickkopf homolog 1 (Xenopus laevis) (DKK1), mRNA.
NM_006885.3	ZFHX3	6.456	Homo sapiens zinc finger homeobox 3 (ZFHX3), transcript variant A, mRNA.
NM_024423.1	DSC3	6.451	Homo sapiens desmocollin 3 (DSC3), transcript variant Dsc3b, mRNA.
NM_003670.1	BHLHB2	6.448	Homo sapiens basic helix-loop-helix domain containing, class B, 2 (BHLHB2), mRNA.
NM_002160.1	TNC	6.437	Homo sapiens tenascin C (hexabrachion) (TNC), mRNA.
NM_006206.3	PDGFRA	6.400	Homo sapiens platelet-derived growth factor receptor, alpha polypeptide (PDGFRA), mRNA.
NM_000509.4	FGG	6.399	Homo sapiens fibrinogen gamma chain (FGG), transcript variant gamma-A, mRNA.

AK025332		6.390	Homo sapiens cDNA: FLJ21679 fis, clone COL09221
NM_001078.2	VCAM1	6.363	Homo sapiens vascular cell adhesion molecule 1 (VCAM1), transcript variant 1, mRNA.
NM_016267.2	VGLL1	6.343	Homo sapiens vestigial like 1 (Drosophila) (VGLL1), mRNA.
NM_017633.2	FAM46A	6.316	Homo sapiens family with sequence similarity 46, member A (FAM46A), mRNA.
NM_020962.1	IGDCC4	6.303	Homo sapiens immunoglobulin superfamily, DCC subclass, member 4 (IGDCC4), mRNA.
NM_001047841.1	CXCR7	6.300	Homo sapiens chemokine (C-X-C motif) receptor 7 (CXCR7), transcript variant 1, mRNA.
NM_001080951.1	PLAGL1	6.292	Homo sapiens pleiomorphic adenoma gene-like 1 (PLAGL1), transcript variant 3, mRNA.
NM_032041.1	NCALD	6.266	Homo sapiens neurocalcin delta (NCALD), mRNA.
NM_004428.2	EFNA1	6.231	Homo sapiens ephrin-A1 (EFNA1), transcript variant 1, mRNA.
NM_025202.2	EFHD1	6.223	Homo sapiens EF-hand domain family, member D1 (EFHD1), mRNA.
XM_001126471.1	LOC730278	6.220	PREDICTED: Homo sapiens hypothetical LOC730278 (LOC730278), mRNA.
NM_006182.2	DDR2	6.197	Homo sapiens discoidin domain receptor tyrosine kinase 2 (DDR2), transcript variant 2, mRNA.
NM_005797.2	MPZL2	6.140	Homo sapiens myelin protein zero-like 2 (MPZL2), transcript variant 1, mRNA.
NM_005221.5	DLX5	6.112	Homo sapiens distal-less homeobox 5 (DLX5), mRNA.
NM_018584.5	CAMK2N1	6.109	Homo sapiens calcium/calmodulin-dependent protein kinase II inhibitor 1 (CAMK2N1), mRNA.
NM_016509.3	CLEC1B	6.024	Homo sapiens C-type lectin domain family 1, member B (CLEC1B), transcript variant 1, mRNA.
NM_006096.2	NDRG1	5.992	Homo sapiens N-myc downstream regulated gene 1 (NDRG1), mRNA.
NM_002982.3	CCL2	5.970	Homo sapiens chemokine (C-C motif) ligand 2 (CCL2), mRNA.
NM_006283.1	TACC1	5.970	Homo sapiens transforming, acidic coiled-coil containing protein 1 (TACC1), mRNA.
NM_004878.3	PTGES	5.943	Homo sapiens prostaglandin E synthase (PTGES), mRNA.
NM_001717.2	BNC1	5.916	Homo sapiens basonuclin 1 (BNC1), mRNA.
NM_000099.2	CST3	5.901	Homo sapiens cystatin C (CST3), mRNA.
NM_014585.4	SLC40A1	5.890	Homo sapiens solute carrier family 40 (iron-regulated transporter), member 1 (SLC40A1), mRNA.
NM_002048.1	GAS1	5.888	Homo sapiens growth arrest-specific 1 (GAS1), mRNA.
NM_182827.1	FKBP9L	5.880	Homo sapiens FK506 binding protein 9-like (FKBP9L), mRNA.
NM_014624.3	S100A6	5.870	Homo sapiens S100 calcium binding protein A6 (S100A6), mRNA.
NM_000943.4	PPIC	5.820	Homo sapiens peptidylprolyl isomerase C (cyclophilin C) (PPIC), mRNA.
NM_021005.2	NR2F2	5.807	Homo sapiens nuclear receptor subfamily 2, group F, member 2 (NR2F2), mRNA.
NM_001451.2	FOXF1	5.807	Homo sapiens forkhead box F1 (FOXF1), mRNA.
NM_145056.1	DACT3	5.793	Homo sapiens dapper, antagonist of beta-catenin, homolog 3 (Xenopus laevis) (DACT3), mRNA.
NM_006487.2	FBLN1	5.791	Homo sapiens fibulin 1 (FBLN1), transcript variant A, mRNA.
NM_002414.3	CD99	5.773	Homo sapiens CD99 molecule (CD99), transcript variant 1, mRNA.
NM_002380.3	MATN2	5.716	Homo sapiens matrilin 2 (MATN2), transcript variant 1, mRNA.
NM_004936.3	CDKN2B	5.677	Homo sapiens cyclin-dependent kinase inhibitor 2B (p15, inhibits CDK4) (CDKN2B), transcript variant 1, mRNA.
NM_001854.3	COL11A1	5.661	Homo sapiens collagen, type XI, alpha 1 (COL11A1), transcript variant A, mRNA.
NM_000325.5	PITX2	5.660	Homo sapiens paired-like homeodomain 2 (PITX2), transcript variant 3, mRNA.
NM_001629.2	ALOX5AP	5.660	Homo sapiens arachidonate 5-lipoxygenase-activating protein

			(ALOX5AP), mRNA.
NM_018012.2	KIF26B	5.645	Homo sapiens kinesin family member 26B (KIF26B), mRNA.
NM_002191.2	INHA	5.613	Homo sapiens inhibin, alpha (INHA), mRNA.
NM_199355.2	ADAMTS18	5.604	Homo sapiens ADAM metallopeptidase with thrombospondin type 1 motif, 18 (ADAMTS18), mRNA.
NM_006751.4	SSFA2	5.598	Homo sapiens sperm specific antigen 2 (SSFA2), mRNA.
NM_032199.1	ARID5B	5.588	Homo sapiens AT rich interactive domain 5B (MRF1-like) (ARID5B), mRNA.
NM_001134.1	AFP	5.576	Homo sapiens alpha-fetoprotein (AFP), mRNA.
NM_001717.2	BNC1	5.575	Homo sapiens basonuclin 1 (BNC1), mRNA.
NM_018712.2	ELMOD1	5.572	Homo sapiens ELMO/CED-12 domain containing 1 (ELMOD1), mRNA.
NM_000888.3	ITGB6	5.556	Homo sapiens integrin, beta 6 (ITGB6), mRNA.
NM_006255.3	PRKCH	5.495	Homo sapiens protein kinase C, eta (PRKCH), mRNA.
NM_014900.3	COBLL1	5.469	Homo sapiens COBL-like 1 (COBLL1), mRNA.
NM_006307.3	SRPX	5.463	Homo sapiens sushi-repeat-containing protein, X-linked (SRPX), mRNA.
NM_014795.2	ZEB2	5.462	Homo sapiens zinc finger E-box binding homeobox 2 (ZEB2), mRNA.
NM_019555.1	ARHGEF3	5.459	Homo sapiens Rho guanine nucleotide exchange factor (GEF) 3 (ARHGEF3), mRNA.
NM_005620.1	S100A11	5.451	Homo sapiens S100 calcium binding protein A11 (S100A11), mRNA.
NM_147780.2	CTSB	5.428	Homo sapiens cathepsin B (CTSB), transcript variant 2, mRNA.
NM_000477.3	ALB	5.425	Homo sapiens albumin (ALB), mRNA.
NM_001024912.1	CEACAM1	5.415	Homo sapiens carcinoembryonic antigen-related cell adhesion molecule 1 (biliary glycoprotein) (CEACAM1), transcript variant 2, mRNA.
NM_015130.2	TBC1D9	5.391	Homo sapiens TBC1 domain family, member 9 (with GRAM domain) (TBC1D9), mRNA.
NM_001684.3	ATP2B4	5.365	Homo sapiens ATPase, Ca++ transporting, plasma membrane 4 (ATP2B4), transcript variant 2, mRNA.
NM_005429.2	VEGFC	5.364	Homo sapiens vascular endothelial growth factor C (VEGFC), mRNA.
NM_212474.1	FN1	5.348	Homo sapiens fibronectin 1 (FN1), transcript variant 6, mRNA.
NM_016657.1	KDELR3	5.338	Homo sapiens KDEL (Lys-Asp-Glu-Leu) endoplasmic reticulum protein retention receptor 3 (KDELR3), transcript variant 2, mRNA.
NM_002247.2	KCNMA1	5.330	Homo sapiens potassium large conductance calcium-activated channel, subfamily M, alpha member 1 (KCNMA1), transcript variant 2, mRNA.
NM_016174.3	CERCAM	5.310	Homo sapiens cerebral endothelial cell adhesion molecule (CERCAM), mRNA.
NM_030665.3	RAI1	5.307	Homo sapiens retinoic acid induced 1 (RAI1), mRNA.
NM_006855.2	KDELR3	5.262	Homo sapiens KDEL (Lys-Asp-Glu-Leu) endoplasmic reticulum protein retention receptor 3 (KDELR3), transcript variant 1, mRNA.
NM_006902.3	PRRX1	5.260	Homo sapiens paired related homeobox 1 (PRRX1), transcript variant pmx-1a, mRNA.
NM_014391.2	ANKRD1	5.241	Homo sapiens ankyrin repeat domain 1 (cardiac muscle) (ANKRD1), mRNA.
NM_003613.2	CILP	5.231	Homo sapiens cartilage intermediate layer protein, nucleotide pyrophosphohydrolase (CILP), mRNA.
NM_005996.3	TBX3	5.220	Homo sapiens T-box 3 (TBX3), transcript variant 1, mRNA.
NM_022872.2	IFI6	5.207	Homo sapiens interferon, alpha-inducible protein 6 (IFI6), transcript variant 2, mRNA.
NM_001033047.1	NPNT	5.184	Homo sapiens nephronectin (NPNT), mRNA.

NM_005786.4	TSHZ1	5.179	Homo sapiens teashirt zinc finger homeobox 1 (TSHZ1), mRNA.
NM_001001396.1	ATP2B4	5.177	Homo sapiens ATPase, Ca++ transporting, plasma membrane 4 (ATP2B4), transcript variant 1, mRNA.
NM_003714.2	STC2	5.175	Homo sapiens stanniocalcin 2 (STC2), mRNA.
NM_014459.2	PCDH17	5.144	Homo sapiens protocadherin 17 (PCDH17), mRNA.
NM_024336.1	IRX3	5.142	Homo sapiens iroquois homeobox 3 (IRX3), mRNA.
NM_002276.3	KRT19	5.138	Homo sapiens keratin 19 (KRT19), mRNA.
NM_001996.2	FBLN1	5.130	Homo sapiens fibulin 1 (FBLN1), transcript variant C, mRNA.
NM_001842.3	CNTFR	5.124	Homo sapiens ciliary neurotrophic factor receptor (CNTFR), transcript variant 2, mRNA.
NM_080473.3	GATA5	5.121	Homo sapiens GATA binding protein 5 (GATA5), mRNA.
NM_002521.2	NPPB	5.113	Homo sapiens natriuretic peptide precursor B (NPPB), mRNA.
NM_003380.2	VIM	5.084	Homo sapiens vimentin (VIM), mRNA.
NM_002845.2	PTPRM	5.073	Homo sapiens protein tyrosine phosphatase, receptor type, M (PTPRM), mRNA.
NM_004864.1	GDF15	5.070	Homo sapiens growth differentiation factor 15 (GDF15), mRNA.
NM_001032281.2	TFPI	5.070	Homo sapiens tissue factor pathway inhibitor (lipoprotein-associated coagulation inhibitor) (TFPI), transcript variant 2, mRNA.
NM_024430.2	PSTPIP2	5.067	Homo sapiens proline-serine-threonine phosphatase interacting protein 2 (PSTPIP2), mRNA.
NM_001001431.1	TNNT2	5.064	Homo sapiens troponin T type 2 (cardiac) (TNNT2), transcript variant 3, mRNA.
NM_197941.2	ADAMTS6	5.064	Homo sapiens ADAM metallopeptidase with thrombospondin type 1 motif, 6 (ADAMTS6), mRNA.
NM_182909.2	FILIP1L	5.058	Homo sapiens filamin A interacting protein 1-like (FILIP1L), transcript variant 1, mRNA.
NM_004093.2	EFNB2	5.056	Homo sapiens ephrin-B2 (EFNB2), mRNA.
XM_001133042.1	PDZRN3	5.055	PREDICTED: Homo sapiens PDZ domain containing RING finger 3 (PDZRN3), mRNA.
NM_134268.3	CYGB	5.015	Homo sapiens cytoglobin (CYGB), mRNA.
NM_031479.3	INHBE	5.007	Homo sapiens inhibin, beta E (INHBE), mRNA.
XR_017543.1	LOC400578	4.999	PREDICTED: Homo sapiens similar to Keratin, type I cytoskeletal 14 (Cytokeratin-14) (CK-14) (Keratin-14) (K14) (LOC400578), mRNA.
NM_144766.1	RGS13	4.974	Homo sapiens regulator of G-protein signaling 13 (RGS13), transcript variant 2, mRNA.
NM_000735.2	CGA	4.965	Homo sapiens glycoprotein hormones, alpha polypeptide (CGA), mRNA.
NM_153362.1	PRSS35	4.959	Homo sapiens protease, serine, 35 (PRSS35), mRNA.
NM_000104.2	CYP1B1	4.952	Homo sapiens cytochrome P450, family 1, subfamily B, polypeptide 1 (CYP1B1), mRNA.
AK095831		4.952	Homo sapiens cDNA FLJ38512 fis, clone HCHON2000503
NM_002205.2	ITGA5	4.935	Homo sapiens integrin, alpha 5 (fibronectin receptor, alpha polypeptide) (ITGA5), mRNA.
NM_014365.2	HSPB8	4.925	Homo sapiens heat shock 22kDa protein 8 (HSPB8), mRNA.
NM_006403.2	NEDD9	4.918	Homo sapiens neural precursor cell expressed, developmentally down-regulated 9 (NEDD9), transcript variant 1, mRNA.
NM_006983.1	MMP23B	4.912	Homo sapiens matrix metallopeptidase 23B (MMP23B), mRNA.
NM_018469.3	TEX2	4.902	Homo sapiens testis expressed 2 (TEX2), mRNA.
NM_020962.1	IGDCC4	4.865	Homo sapiens immunoglobulin superfamily, DCC subclass, member 4 (IGDCC4), mRNA.
NM_006129.2	BMP1	4.862	Homo sapiens bone morphogenetic protein 1 (BMP1), transcript variant BMP1-3, mRNA.
NM_172112.1	EYA2	4.857	Homo sapiens eyes absent homolog 2 (Drosophila) (EYA2), transcript variant 4, mRNA.
NM_001042459.1	FILIP1L	4.846	Homo sapiens filamin A interacting protein 1-like (FILIP1L),

			transcript variant 3, mRNA.
NM_201433.1	GAS7	4.831	Homo sapiens growth arrest-specific 7 (GAS7), transcript variant c, mRNA.
NM_005114.2	HS3ST1	4.816	Homo sapiens heparan sulfate (glucosamine) 3-O-sulfotransferase 1 (HS3ST1), mRNA.
NM_004530.2	MMP2	4.800	Homo sapiens matrix metallopeptidase 2 (gelatinase A, 72kDa gelatinase, 72kDa type IV collagenase) (MMP2), mRNA.
NM_001998.2	FBLN2	4.783	Homo sapiens fibulin 2 (FBLN2), transcript variant 2, mRNA.
NM_152611.3	LRRN4	4.781	Homo sapiens leucine rich repeat neuronal 4 (LRRN4), mRNA.
NM_182487.2	OLFML2A	4.760	Homo sapiens olfactomedin-like 2A (OLFML2A), mRNA.
NM_003469.3	SCG2	4.748	Homo sapiens secretogranin II (chromogranin C) (SCG2), mRNA.
NM_001006666.1	APOBEC3F	4.745	Homo sapiens apolipoprotein B mRNA editing enzyme, catalytic polypeptide-like 3F (APOBEC3F), transcript variant 2, mRNA.
NM_005576.2	LOXL1	4.732	Homo sapiens lysyl oxidase-like 1 (LOXL1), mRNA.
NM_004411.3	DYNC1I1	4.720	Homo sapiens dynein, cytoplasmic 1, intermediate chain 1 (DYNC1I1), mRNA.
NM_006203.3	PDE4D	4.720	Homo sapiens phosphodiesterase 4D, cAMP-specific (phosphodiesterase E3 dunce homolog, Drosophila) (PDE4D), mRNA.
NM_003380.2	VIM	4.710	Homo sapiens vimentin (VIM), mRNA.
NM_025179.3	PLXNA2	4.693	Homo sapiens plexin A2 (PLXNA2), mRNA.
NM_003982.2	SLC7A7	4.690	Homo sapiens solute carrier family 7 (cationic amino acid transporter, y+ system), member 7 (SLC7A7), mRNA.
NM_007112.3	THBS3	4.654	Homo sapiens thrombospondin 3 (THBS3), mRNA.
NM_002543.3	OLR1	4.647	Homo sapiens oxidized low density lipoprotein (lectin-like) receptor 1 (OLR1), mRNA.
NR_001562.1	ANXA2P1	4.638	Homo sapiens annexin A2 pseudogene 1 (ANXA2P1) on chromosome 4.
NM_003033.2	ST3GAL1	4.637	Homo sapiens ST3 beta-galactoside alpha-2,3-sialyltransferase 1 (ST3GAL1), transcript variant 1, mRNA.
NM_020808.3	SIPA1L2	4.635	Homo sapiens signal-induced proliferation-associated 1 like 2 (SIPA1L2), mRNA.
NM_001846.2	COL4A2	4.628	Homo sapiens collagen, type IV, alpha 2 (COL4A2), mRNA.
NM_000878.2	IL2RB	4.613	Homo sapiens interleukin 2 receptor, beta (IL2RB), mRNA.
NM_003882.2	WISP1	4.609	Homo sapiens WNT1 inducible signaling pathway protein 1 (WISP1), transcript variant 1, mRNA.
NM_004884.3	IGDCC3	4.605	Homo sapiens immunoglobulin superfamily, DCC subclass, member 3 (IGDCC3), mRNA.
NM_006270.3	RRAS	4.598	Homo sapiens related RAS viral (r-ras) oncogene homolog (RRAS), mRNA.
NM_004154.3	P2RY6	4.591	Homo sapiens pyrimidinergic receptor P2Y, G-protein coupled, 6 (P2RY6), transcript variant 4, mRNA.
NM_003238.1	TGFB2	4.573	Homo sapiens transforming growth factor, beta 2 (TGFB2), mRNA.
NM_207361.4	FREM2	4.572	Homo sapiens FRAS1 related extracellular matrix protein 2 (FREM2), mRNA.
NM_032918.1	RERG	4.550	Homo sapiens RAS-like, estrogen-regulated, growth inhibitor (RERG), mRNA.
NM_002966.1	S100A10	4.540	Homo sapiens S100 calcium binding protein A10 (annexin II ligand, calpactin I, light polypeptide (p11)) (S100A10), mRNA.
NM_001458.3	FLNC	4.523	Homo sapiens filamin C, gamma (actin binding protein 280) (FLNC), mRNA.
NM_001002236.1	SERPINA1	4.523	Homo sapiens serpin peptidase inhibitor, clade A (alpha-1 antiproteinase, antitrypsin), member 1 (SERPINA1), transcript variant 2, mRNA.
NM_002380.3	MATN2	4.513	Homo sapiens matrilin 2 (MATN2), transcript variant 1, mRNA.
NM_001040619.1	ATF3	4.506	Homo sapiens activating transcription factor 3 (ATF3), transcript

			variant 4, mRNA.
NM_198252.2	GSN	4.495	Homo sapiens gelsolin (amyloidosis, Finnish type) (GSN), transcript variant 2, mRNA.
NM_052966.2	FAM129A	4.466	Homo sapiens family with sequence similarity 129, member A (FAM129A), transcript variant 2, mRNA.
NM_022369.2	STRA6	4.454	Homo sapiens stimulated by retinoic acid gene 6 homolog (mouse) (STRA6), mRNA.
NM_015529.2	MOXD1	4.450	Homo sapiens monooxygenase, DBH-like 1 (MOXD1), transcript variant 2, mRNA.
NM_175873.4	ANKRD43	4.445	Homo sapiens ankyrin repeat domain 43 (ANKRD43), mRNA.
NM_030672.2	ARHGAP28	4.432	Homo sapiens Rho GTPase activating protein 28 (ARHGAP28), transcript variant 2, mRNA.
NM_015691.2	WWC3	4.408	Homo sapiens WWC family member 3 (WWC3), mRNA.
NM_013279.1	C11orf9	4.399	Homo sapiens chromosome 11 open reading frame 9 (C11orf9), transcript variant 1, mRNA.
NM_005907.2	MAN1A1	4.398	Homo sapiens mannosidase, alpha, class 1A, member 1 (MAN1A1), mRNA.
NM_133367.3	PAQR8	4.394	Homo sapiens progestin and adipoQ receptor family member VIII (PAQR8), mRNA.
NM_144729.1	DUSP10	4.393	Homo sapiens dual specificity phosphatase 10 (DUSP10), transcript variant 3, mRNA.
NM_006290.2	TNFAIP3	4.387	Homo sapiens tumor necrosis factor, alpha-induced protein 3 (TNFAIP3), mRNA.
NM_000784.2	CYP27A1	4.386	Homo sapiens cytochrome P450, family 27, subfamily A, polypeptide 1 (CYP27A1), nuclear gene encoding mitochondrial protein, mRNA.
NM_016205.1	PDGFC	4.378	Homo sapiens platelet derived growth factor C (PDGFC), mRNA.
NM_006851.2	GLIPR1	4.377	Homo sapiens GLI pathogenesis-related 1 (GLIPR1), mRNA.
NM_001423.1	EMP1	4.374	Homo sapiens epithelial membrane protein 1 (EMP1), mRNA.
NM_005318.2	H1F0	4.370	Homo sapiens H1 histone family, member 0 (H1F0), mRNA.
NM_177964.3	LYPD6B	4.362	Homo sapiens LY6/PLAUR domain containing 6B (LYPD6B), mRNA.
BX537506		4.360	Homo sapiens mRNA; cDNA DKFZp779K2051 (from clone DKFZp779K2051)
NM_002966.2	S100A10	4.346	Homo sapiens S100 calcium binding protein A10 (S100A10), mRNA.
NM_015170.1	SULF1	4.338	Homo sapiens sulfatase 1 (SULF1), mRNA.
NM_005562.1	LAMC2	4.328	Homo sapiens laminin, gamma 2 (LAMC2), transcript variant 1, mRNA.
NM_001463.2	FRZB	4.327	Homo sapiens frizzled-related protein (FRZB), mRNA.
NM_001548.3	IFIT1	4.321	Homo sapiens interferon-induced protein with tetratricopeptide repeats 1 (IFIT1), transcript variant 2, mRNA.
NM_014583.2	LMCD1	4.312	Homo sapiens LIM and cysteine-rich domains 1 (LMCD1), mRNA.
NM_198392.1	TCF21	4.307	Homo sapiens transcription factor 21 (TCF21), transcript variant 1, mRNA.
NM_014333.3	CADM1	4.299	Homo sapiens cell adhesion molecule 1 (CADM1), transcript variant 1, mRNA.
NM_001998.2	FBLN2	4.293	Homo sapiens fibulin 2 (FBLN2), transcript variant 2, mRNA.
NM_015206.1	KIAA1024	4.288	Homo sapiens KIAA1024 (KIAA1024), mRNA.
NM_173848.5	RALYL	4.276	Homo sapiens RALY RNA binding protein-like (RALYL), transcript variant 3, mRNA.
NM_178565.3	RSPO2	4.271	Homo sapiens R-spondin 2 homolog (Xenopus laevis) (RSPO2), mRNA.
NM_001040626.1	NCALD	4.265	Homo sapiens neurocalcin delta (NCALD), transcript variant 3, mRNA.
NM_006475.1	POSTN	4.260	Homo sapiens periostin, osteoblast specific factor (POSTN), mRNA.
NM_000689.3	ALDH1A1	4.259	Homo sapiens aldehyde dehydrogenase 1 family, member A1

			(ALDH1A1), mRNA.
NM_014935.2	PLEKHA6	4.259	Homo sapiens pleckstrin homology domain containing, family A member 6 (PLEKHA6), mRNA.
NM_003181.2	T	4.255	Homo sapiens T, brachyury homolog (mouse) (T), mRNA.
NM_003275.2	TMOD1	4.246	Homo sapiens tropomodulin 1 (TMOD1), mRNA.
NM_000163.2	GHR	4.242	Homo sapiens growth hormone receptor (GHR), mRNA.
NM_021223.2	MYL7	4.220	Homo sapiens myosin, light chain 7, regulatory (MYL7), mRNA.
XR_015970.1	MGC102966	4.214	PREDICTED: Homo sapiens similar to Keratin, type I cytoskeletal 16 (Cytokeratin-16) (CK-16) (Keratin-16) (K16) (MGC102966), misc RNA.
NM_080805.2	COL13A1	4.213	Homo sapiens collagen, type XIII, alpha 1 (COL13A1), transcript variant 9, mRNA.
NM_005228.3	EGFR	4.208	Homo sapiens epidermal growth factor receptor (erythroblastic leukemia viral (v-erb-b) oncogene homolog, avian) (EGFR), transcript variant 1, mRNA.
NM_003633.1	ENC1	4.208	Homo sapiens ectodermal-neural cortex (with BTB-like domain) (ENC1), mRNA.
NM_000300.2	PLA2G2A	4.201	Homo sapiens phospholipase A2, group IIA (platelets, synovial fluid) (PLA2G2A), mRNA.
NM_022055.1	KCNK12	4.187	Homo sapiens potassium channel, subfamily K, member 12 (KCNK12), mRNA.
NM_172313.1	CSF3R	4.184	Homo sapiens colony stimulating factor 3 receptor (granulocyte) (CSF3R), transcript variant 4, mRNA.
NM_007088.2	CALB2	4.167	Homo sapiens calbindin 2 (CALB2), transcript variant CALB2c, mRNA.
NM_080826.1	ISM1	4.141	Homo sapiens isthmin 1 homolog (zebrafish) (ISM1), mRNA.
NM_001005340.1	GPNMB	4.139	Homo sapiens glycoprotein (transmembrane) nmb (GPNMB), transcript variant 1, mRNA.
XM_931359.2	LOC338758	4.137	PREDICTED: Homo sapiens hypothetical protein LOC338758 (LOC338758), mRNA.
NM_024928.3	OBFC1	4.127	Homo sapiens oligonucleotide/oligosaccharide-binding fold containing 1 (OBFC1), mRNA.
NM_004364.2	CEBPA	4.124	Homo sapiens CCAAT/enhancer binding protein (C/EBP), alpha (CEBPA), mRNA.
NM_003937.2	KYNU	4.115	Homo sapiens kynureninase (L-kynurenine hydrolase) (KYNU), transcript variant 1, mRNA.
NM_005654.4	NR2F1	4.094	Homo sapiens nuclear receptor subfamily 2, group F, member 1 (NR2F1), mRNA.
NM_181873.2	MTMR11	4.080	Homo sapiens myotubularin related protein 11 (MTMR11), mRNA.
NM_006528.2	TFPI2	4.079	Homo sapiens tissue factor pathway inhibitor 2 (TFPI2), mRNA.
NM_005605.3	PPP3CC	4.078	Homo sapiens protein phosphatase 3 (formerly 2B), catalytic subunit, gamma isoform (PPP3CC), mRNA.
NM_021973.2	HAND2	4.066	Homo sapiens heart and neural crest derivatives expressed 2 (HAND2), mRNA.
NM_198098.1	AQP1	4.053	Homo sapiens aquaporin 1 (Colton blood group) (AQP1), mRNA.
NM_001630.2	ANXA8L2	4.041	Homo sapiens annexin A8-like 2 (ANXA8L2), mRNA.
NM_052941.3	GBP4	4.038	Homo sapiens guanylate binding protein 4 (GBP4), mRNA.
NM_033504.2	TMEM54	4.037	Homo sapiens transmembrane protein 54 (TMEM54), mRNA.
NM_000404.1	GLB1	4.036	Homo sapiens galactosidase, beta 1 (GLB1), transcript variant 179423, mRNA.
NM_014631.2	SH3PXD2A	4.003	Homo sapiens SH3 and PX domains 2A (SH3PXD2A), mRNA.
NM_002402.2	MEST	3.985	Homo sapiens mesoderm specific transcript homolog (mouse) (MEST), transcript variant 1, mRNA.
NM_003335.2	UBA7	3.984	Homo sapiens ubiquitin-like modifier activating enzyme 7 (UBA7), mRNA.
NM_203370.1	C3orf54	3.983	Homo sapiens chromosome 3 open reading frame 54 (C3orf54),

			mRNA.
NM_001036.2	RYR3	3.982	Homo sapiens ryanodine receptor 3 (RYR3), mRNA.
NM_033380.1	COL4A5	3.976	Homo sapiens collagen, type IV, alpha 5 (COL4A5), transcript variant 2, mRNA.
NM_004525.2	LRP2	3.957	Homo sapiens low density lipoprotein-related protein 2 (LRP2), mRNA.
NM_006834.2	RAB32	3.953	Homo sapiens RAB32, member RAS oncogene family (RAB32), mRNA.
NM_033119.3	NKD1	3.943	Homo sapiens naked cuticle homolog 1 (Drosophila) (NKD1), mRNA.
NM_004884.3	IGDCC3	3.939	Homo sapiens immunoglobulin superfamily, DCC subclass, member 3 (IGDCC3), mRNA.
NM_139314.1	ANGPTL4	3.935	Homo sapiens angiopoietin-like 4 (ANGPTL4), transcript variant 1, mRNA.
NM_014322.2	OPN3	3.935	Homo sapiens opsin 3 (OPN3), mRNA.
NM_000494.3	COL17A1	3.929	Homo sapiens collagen, type XVII, alpha 1 (COL17A1), mRNA.
NM_002273.2	KRT8	3.925	Homo sapiens keratin 8 (KRT8), mRNA.
NM_001002858.1	ANXA2	3.920	Homo sapiens annexin A2 (ANXA2), transcript variant 1, mRNA.
NM_022059.1	CXCL16	3.909	Homo sapiens chemokine (C-X-C motif) ligand 16 (CXCL16), mRNA.
NM_024690.2	MUC16	3.905	Homo sapiens mucin 16, cell surface associated (MUC16), mRNA.
AF131784		3.899	Homo sapiens clone 25194 mRNA sequence
NM_001908.3	CTSB	3.882	Homo sapiens cathepsin B (CTSB), transcript variant 1, mRNA.
NM_005908.3	MANBA	3.877	Homo sapiens mannosidase, beta A, lysosomal (MANBA), mRNA.
NM_080629.2	COL11A1	3.876	Homo sapiens collagen, type XI, alpha 1 (COL11A1), transcript variant B, mRNA.
NM_152680.1	TMEM154	3.853	Homo sapiens transmembrane protein 154 (TMEM154), mRNA.
NM_001849.3	COL6A2	3.848	Homo sapiens collagen, type VI, alpha 2 (COL6A2), transcript variant 2C2, mRNA.
NM_022138.1	SMOC2	3.847	Homo sapiens SPARC related modular calcium binding 2 (SMOC2), mRNA.
NM_005097.1	LGI1	3.839	Homo sapiens leucine-rich, glioma inactivated 1 (LGI1), mRNA.
NM_002306.1	LGALS3	3.837	Homo sapiens lectin, galactoside-binding, soluble, 3 (galectin 3) (LGALS3), mRNA.
NM_080388.1	S100A16	3.830	Homo sapiens S100 calcium binding protein A16 (S100A16), mRNA.
NM_004789.3	LHX2	3.829	Homo sapiens LIM homeobox 2 (LHX2), mRNA.
NM_001425.1	EMP3	3.825	Homo sapiens epithelial membrane protein 3 (EMP3), mRNA.
NM_002291.1	LAMB1	3.825	Homo sapiens laminin, beta 1 (LAMB1), mRNA.
NM_001147.1	ANGPT2	3.810	Homo sapiens angiopoietin 2 (ANGPT2), mRNA.
NM_024841.3	FLJ14213	3.799	Homo sapiens protor-2 (FLJ14213), mRNA.
NM_030665.3	RAI1	3.779	Homo sapiens retinoic acid induced 1 (RAI1), mRNA.
NM_005460.2	SNCAIP	3.771	Homo sapiens synuclein, alpha interacting protein (SNCAIP), mRNA.
NM_000891.2	KCNJ2	3.770	Homo sapiens potassium inwardly-rectifying channel, subfamily J, member 2 (KCNJ2), mRNA.
NM_001006932.1	RPS6KA2	3.769	Homo sapiens ribosomal protein S6 kinase, 90kDa, polypeptide 2 (RPS6KA2), transcript variant 2, mRNA.
NM_001079811.1	GLB1	3.762	Homo sapiens galactosidase, beta 1 (GLB1), transcript variant 2, mRNA.
NM_001233.3	CAV2	3.760	Homo sapiens caveolin 2 (CAV2), transcript variant 1, mRNA.
NM_138389.1	FAM114A1	3.756	Homo sapiens family with sequence similarity 114, member A1 (FAM114A1), mRNA.
NM_016651.5	DACT1	3.754	Homo sapiens dapper, antagonist of beta-catenin, homolog 1 (Xenopus laevis) (DACT1), transcript variant 1, mRNA.
NM_022062.2	PKNOX2	3.751	Homo sapiens PBX/knotted 1 homeobox 2 (PKNOX2), mRNA.
NM_032873.4	UBASH3B	3.731	Homo sapiens ubiquitin associated and SH3 domain containing, B

			(UBASH3B), mRNA.
NM_001014447.1	CPZ	3.730	Homo sapiens carboxypeptidase Z (CPZ), transcript variant 1, mRNA.
NM_001766.3	CD1D	3.727	Homo sapiens CD1d molecule (CD1D), mRNA.
XR_017100.2	LOC149501	3.724	PREDICTED: Homo sapiens misc_RNA (LOC149501), miscRNA.
NM_001878.2	CRABP2	3.724	Homo sapiens cellular retinoic acid binding protein 2 (CRABP2), mRNA.
NM_177964.3	LYPD6B	3.716	Homo sapiens LY6/PLAUR domain containing 6B (LYPD6B), mRNA.
NM_001343.2	DAB2	3.710	Homo sapiens disabled homolog 2, mitogen-responsive phosphoprotein (Drosophila) (DAB2), mRNA.
NM_000877.2	IL1R1	3.709	Homo sapiens interleukin 1 receptor, type I (IL1R1), mRNA.
NM_005461.3	MAFB	3.700	Homo sapiens v-maf musculoaponeurotic fibrosarcoma oncogene homolog B (avian) (MAFB), mRNA.
NM_006714.2	SMPDL3A	3.700	Homo sapiens sphingomyelin phosphodiesterase, acid-like 3A (SMPDL3A), mRNA.
NM_012071.2	COMMD3	3.698	Homo sapiens COMM domain containing 3 (COMMD3), mRNA.
NM_178839.4	LRRTM1	3.686	Homo sapiens leucine rich repeat transmembrane neuronal 1 (LRRTM1), mRNA.
NM_005531.1	IFI16	3.685	Homo sapiens interferon, gamma-inducible protein 16 (IFI16), mRNA.
NM_000862.2	HSD3B1	3.680	Homo sapiens hydroxy-delta-5-steroid dehydrogenase, 3 beta- and steroid delta-isomerase 1 (HSD3B1), mRNA.
NM_001873.1	CPE	3.677	Homo sapiens carboxypeptidase E (CPE), mRNA.
NM_033274.2	ADAM19	3.674	Homo sapiens ADAM metallopeptidase domain 19 (meltrin beta) (ADAM19), mRNA.
NM_005211.2	CSF1R	3.673	Homo sapiens colony stimulating factor 1 receptor, formerly McDonough feline sarcoma viral (v-fms) oncogene homolog (CSF1R), mRNA.
NM_012211.3	ITGA11	3.670	Homo sapiens integrin, alpha 11 (ITGA11), transcript variant 2, mRNA.
NM_006169.2	NNMT	3.665	Homo sapiens nicotinamide N-methyltransferase (NNMT), mRNA.
NM_005226.2	S1PR3	3.663	Homo sapiens sphingosine-1-phosphate receptor 3 (S1PR3), mRNA.
NM_000693.1	ALDH1A3	3.661	Homo sapiens aldehyde dehydrogenase 1 family, member A3 (ALDH1A3), mRNA.
NM_016815.2	GYPC	3.658	Homo sapiens glycophorin C (Gerbich blood group) (GYPC), transcript variant 2, mRNA.
NM_198057.2	TSC22D3	3.657	Homo sapiens TSC22 domain family, member 3 (TSC22D3), transcript variant 1, mRNA.
NM_172037.2	RDH10	3.655	Homo sapiens retinol dehydrogenase 10 (all-trans) (RDH10), mRNA.
NM_016081.3	PALLD	3.651	Homo sapiens palladin, cytoskeletal associated protein (PALLD), transcript variant 2, mRNA.
NM_014988.1	LIMCH1	3.647	Homo sapiens LIM and calponin homology domains 1 (LIMCH1), mRNA.
NM_001077269.1	WIPF1	3.638	Homo sapiens WAS/WASL interacting protein family, member 1 (WIPF1), transcript variant 2, mRNA.
NM_182908.3	DHRS2	3.630	Homo sapiens dehydrogenase/reductase (SDR family) member 2 (DHRS2), transcript variant 1, mRNA.
XM_927536.1	ALDH1L2	3.621	PREDICTED: Homo sapiens aldehyde dehydrogenase 1 family, member L2 (ALDH1L2), mRNA.
NM_018440.3	PAG1	3.620	Homo sapiens phosphoprotein associated with glycosphingolipid microdomains 1 (PAG1), mRNA.
NM_198501.1	SMTNL2	3.619	Homo sapiens smoothelin-like 2 (SMTNL2), mRNA.
NM_016446.2	C9orf127	3.612	Homo sapiens chromosome 9 open reading frame 127 (C9orf127), mRNA.
NM_001034841.2	ITPRIPL2	3.610	Homo sapiens inositol 1,4,5-triphosphate receptor interacting

			protein-like 2 (ITPRIPL2), mRNA.
NM_014344.2	FJX1	3.607	Homo sapiens four jointed box 1 (Drosophila) (FJX1), mRNA.
NM_002781.2	PSG5	3.605	Homo sapiens pregnancy specific beta-1-glycoprotein 5 (PSG5), mRNA.
NM_001848.2	COL6A1	3.602	Homo sapiens collagen, type VI, alpha 1 (COL6A1), mRNA.
NM_002780.3	PSG4	3.602	Homo sapiens pregnancy specific beta-1-glycoprotein 4 (PSG4), transcript variant 1, mRNA.
NM_000917.2	P4HA1	3.600	Homo sapiens procollagen-proline, 2-oxoglutarate 4-dioxygenase (proline 4-hydroxylase), alpha polypeptide I (P4HA1), transcript variant 1, mRNA.
NM_173624.1	FLJ40504	3.597	Homo sapiens hypothetical protein FLJ40504 (FLJ40504), mRNA.
NM_001010990.1	HERPUD1	3.594	Homo sapiens homocysteine-inducible, endoplasmic reticulum stress-inducible, ubiquitin-like domain member 1 (HERPUD1), transcript variant 3, mRNA.
NM_006329.2	FBLN5	3.589	Homo sapiens fibulin 5 (FBLN5), mRNA.
NM_002430.2	MN1	3.560	Homo sapiens meningioma (disrupted in balanced translocation) 1 (MN1), mRNA.
NM_014786.2	ARHGEF17	3.558	Homo sapiens Rho guanine nucleotide exchange factor (GEF) 17 (ARHGEF17), mRNA.
NM_015869.4	PPARG	3.557	Homo sapiens peroxisome proliferator-activated receptor gamma (PPARG), transcript variant 2, mRNA.
XM_001133042.1	PDZRN3	3.557	PREDICTED: Homo sapiens PDZ domain containing RING finger 3 (PDZRN3), mRNA.
NM_153713.1	LIX1L	3.546	Homo sapiens Lix1 homolog (mouse)-like (LIX1L), mRNA.
NM_003196.1	TCEA3	3.541	Homo sapiens transcription elongation factor A (SII), 3 (TCEA3), mRNA.
NM_001008528.1	MXRA7	3.538	Homo sapiens matrix-remodelling associated 7 (MXRA7), transcript variant 1, mRNA.
NM_183376.1	ARRDC4	3.536	Homo sapiens arrestin domain containing 4 (ARRDC4), mRNA.
NM_002102.3	GYPE	3.532	Homo sapiens glycophorin E (GYPE), transcript variant 1, mRNA.
NM_001073.1	UGT2B11	3.530	Homo sapiens UDP glucuronosyltransferase 2 family, polypeptide B11 (UGT2B11), mRNA.
NM_018653.3	GPRC5C	3.525	Homo sapiens G protein-coupled receptor, family C, group 5, member C (GPRC5C), transcript variant 2, mRNA.
NM_022051.1	EGLN1	3.524	Homo sapiens egl nine homolog 1 (C. elegans) (EGLN1), mRNA.
NM_004048.2	B2M	3.520	Homo sapiens beta-2-microglobulin (B2M), mRNA.
XR_018676.1	LOC647954	3.515	PREDICTED: Homo sapiens misc_RNA (LOC647954), miscRNA.
NM_025008.3	ADAMTSL4	3.513	Homo sapiens ADAMTS-like 4 (ADAMTSL4), transcript variant 2, mRNA.
AK092751		3.512	Homo sapiens cDNA FLJ35432 fis, clone SMINT2002311
NM_001542.2	IGSF3	3.512	Homo sapiens immunoglobulin superfamily, member 3 (IGSF3), transcript variant 1, mRNA.
XR_017241.1	LOC646723	3.505	PREDICTED: Homo sapiens similar to Keratin, type I cytoskeletal 18 (Cytokeratin-18) (CK-18) (Keratin-18) (K18) (LOC646723), mRNA.
NM_001003396.1	TPD52L1	3.487	Homo sapiens tumor protein D52-like 1 (TPD52L1), transcript variant 3, mRNA.
NM_001542.2	IGSF3	3.482	Homo sapiens immunoglobulin superfamily, member 3 (IGSF3), transcript variant 1, mRNA.
NM_001018008.1	TPM1	3.477	Homo sapiens tropomyosin 1 (alpha) (TPM1), transcript variant 6, mRNA.
XM_940375.2	KLHL29	3.475	PREDICTED: Homo sapiens kelch-like 29 (Drosophila) (KLHL29), mRNA.
NM_004659.1	MMP23A	3.473	Homo sapiens matrix metallopeptidase 23A (MMP23A), mRNA.
NM_002615.4	SERPINF1	3.465	Homo sapiens serpin peptidase inhibitor, clade F (alpha-2 antiplasmin, pigment epithelium derived factor), member 1 (SERPINF1), mRNA.

NM_194431.1	RNASE4	3.463	Homo sapiens ribonuclease, RNase A family, 4 (RNASE4), transcript variant 3, mRNA.
NM_000508.3	FGA	3.458	Homo sapiens fibrinogen alpha chain (FGA), transcript variant alpha-E, mRNA.
NM_021158.3	TRIB3	3.457	Homo sapiens tribbles homolog 3 (Drosophila) (TRIB3), mRNA.
NM_022164.1	TINAGL1	3.453	Homo sapiens tubulointerstitial nephritis antigen-like 1 (TINAGL1), mRNA.
XM_001726959.1	KRT18P13	3.442	PREDICTED: Homo sapiens keratin 18 pseudogene 13 (KRT18P13), mRNA.
NM_018557.2	LRP1B	3.440	Homo sapiens low density lipoprotein-related protein 1B (deleted in tumors) (LRP1B), mRNA.
NM_182571.2	FLJ35258	3.438	Homo sapiens hypothetical protein 284297 (FLJ35258), mRNA.
NM_001042445.1	CAST	3.437	Homo sapiens calpastatin (CAST), transcript variant 11, mRNA.
NM_153225.2	RPESP	3.436	Homo sapiens RPE-spondin (RPESP), mRNA.
NM_004089.3	TSC22D3	3.432	Homo sapiens TSC22 domain family, member 3 (TSC22D3), transcript variant 2, mRNA.
NM_001257.3	CDH13	3.416	Homo sapiens cadherin 13, H-cadherin (heart) (CDH13), mRNA.
NM_000495.3	COL4A5	3.411	Homo sapiens collagen, type IV, alpha 5 (COL4A5), transcript variant 1, mRNA.
NM_015245.2	ANKS1A	3.410	Homo sapiens ankyrin repeat and sterile alpha motif domain containing 1A (ANKS1A), mRNA.
NM_002960.1	S100A3	3.405	Homo sapiens S100 calcium binding protein A3 (S100A3), mRNA.
NM_016113.3	TRPV2	3.399	Homo sapiens transient receptor potential cation channel, subfamily V, member 2 (TRPV2), mRNA.
NM_017540.3	GALNT10	3.399	Homo sapiens UDP-N-acetyl-alpha-D-galactosamine:polypeptide N-acetylgalactosaminyltransferase 10 (GalNAc-T10) (GALNT10), transcript variant 2, mRNA.
NM_015009.1	PDZRN3	3.398	Homo sapiens PDZ domain containing ring finger 3 (PDZRN3), mRNA.
NM_130783.3	TSPAN18	3.396	Homo sapiens tetraspanin 18 (TSPAN18), transcript variant 2, mRNA.
NM_001135032.1	FAM176A	3.393	Homo sapiens family with sequence similarity 176, member A (FAM176A), transcript variant 1, mRNA.
NM_001005376.1	PLAUR	3.392	Homo sapiens plasminogen activator, urokinase receptor (PLAUR), transcript variant 2, mRNA.
NM_001856.3	COL16A1	3.389	Homo sapiens collagen, type XVI, alpha 1 (COL16A1), mRNA.
NM_021958.2	HLX	3.388	Homo sapiens H2.0-like homeobox (HLX), mRNA.
XR_038464.1	LOC100132535	3.385	PREDICTED: Homo sapiens misc_RNA (LOC100132535), miscRNA.
NM_003239.1	TGFB3	3.384	Homo sapiens transforming growth factor, beta 3 (TGFB3), mRNA.
NM_004568.4	SERPINB6	3.378	Homo sapiens serpin peptidase inhibitor, clade B (ovalbumin), member 6 (SERPINB6), mRNA.
NM_001042437.1	ST3GAL5	3.373	Homo sapiens ST3 beta-galactoside alpha-2,3-sialyltransferase 5 (ST3GAL5), transcript variant 2, mRNA.
NM_003377.3	VEGFB	3.371	Homo sapiens vascular endothelial growth factor B (VEGFB), mRNA.
NM_006982.1	CART1	3.362	Homo sapiens cartilage paired-class homeoprotein 1 (CART1), mRNA.
NM_001849.3	COL6A2	3.360	Homo sapiens collagen, type VI, alpha 2 (COL6A2), transcript variant 2C2, mRNA.
NM_002168.2	IDH2	3.356	Homo sapiens isocitrate dehydrogenase 2 (NADP+), mitochondrial (IDH2), nuclear gene encoding mitochondrial protein, mRNA.
NM_024702.2	ZNF750	3.351	Homo sapiens zinc finger protein 750 (ZNF750), mRNA.
NM_006266.2	RALGDS	3.349	Homo sapiens ral guanine nucleotide dissociation stimulator (RALGDS), transcript variant 1, mRNA.
NM_148957.2	TNFRSF19	3.349	Homo sapiens tumor necrosis factor receptor superfamily,

			member 19 (TNFRSF19), transcript variant 2, mRNA.
NM_005556.3	KRT7	3.341	Homo sapiens keratin 7 (KRT7), mRNA.
NR_001562.1	ANXA2P1	3.341	Homo sapiens annexin A2 pseudogene 1 (ANXA2P1) on chromosome 4.
NM_001080121.1	PRNP	3.341	Homo sapiens prion protein (PRNP), transcript variant 3, mRNA.
NM_133486.1	MBNL3	3.337	Homo sapiens muscleblind-like 3 (Drosophila) (MBNL3), transcript variant R, mRNA.
NM_005202.1	COL8A2	3.317	Homo sapiens collagen, type VIII, alpha 2 (COL8A2), mRNA.
NM_001008490.1	KLF6	3.313	Homo sapiens Kruppel-like factor 6 (KLF6), transcript variant 1, mRNA.
NM_001135032.1	FAM176A	3.304	Homo sapiens family with sequence similarity 176, member A (FAM176A), transcript variant 1, mRNA.
NM_004428.2	EFNA1	3.304	Homo sapiens ephrin-A1 (EFNA1), transcript variant 1, mRNA.
NM_003060.2	SLC22A5	3.303	Homo sapiens solute carrier family 22 (organic cation transporter), member 5 (SLC22A5), mRNA.
NM_152320.1	ZNF641	3.295	Homo sapiens zinc finger protein 641 (ZNF641), mRNA.
NM_058172.3	ANTXR2	3.295	Homo sapiens anthrax toxin receptor 2 (ANTXR2), mRNA.
NM_001010000.1	ARHGAP28	3.291	Homo sapiens Rho GTPase activating protein 28 (ARHGAP28), transcript variant 1, mRNA.
NM_020404.2	CD248	3.288	Homo sapiens CD248 molecule, endosialin (CD248), mRNA.
NM_144650.2	ADHFE1	3.287	Homo sapiens alcohol dehydrogenase, iron containing, 1 (ADHFE1), nuclear gene encoding mitochondrial protein, mRNA.
NM_032642.2	WNT5B	3.285	Homo sapiens wingless-type MMTV integration site family, member 5B (WNT5B), transcript variant 1, mRNA.
NM_153321.1	PMP22	3.281	Homo sapiens peripheral myelin protein 22 (PMP22), transcript variant 2, mRNA.
NM_198793.2	CD47	3.272	Homo sapiens CD47 molecule (CD47), transcript variant 2, mRNA.
NM_001018008.1	TPM1	3.272	Homo sapiens tropomyosin 1 (alpha) (TPM1), transcript variant 6, mRNA.
NM_016651.4	DACT1	3.269	Homo sapiens dapper, antagonist of beta-catenin, homolog 1 (Xenopus laevis) (DACT1), mRNA.
NM_012168.4	FBXO2	3.267	Homo sapiens F-box protein 2 (FBXO2), mRNA.
NM_002253.1	KDR	3.265	Homo sapiens kinase insert domain receptor (a type III receptor tyrosine kinase) (KDR), mRNA.
NM_147175.3	HS6ST2	3.262	Homo sapiens heparan sulfate 6-O-sulfotransferase 2 (HS6ST2), transcript variant S, mRNA.
NM_018837.2	SULF2	3.259	Homo sapiens sulfatase 2 (SULF2), transcript variant 1, mRNA.
NM_015314.2	KIAA0895	3.258	Homo sapiens KIAA0895 (KIAA0895), transcript variant 2, mRNA.
NM_014398.2	LAMP3	3.257	Homo sapiens lysosomal-associated membrane protein 3 (LAMP3), mRNA.
NM_001007023.2	DIO2	3.256	Homo sapiens deiodinase, iodothyronine, type II (DIO2), transcript variant 3, mRNA.
NM_138455.2	CTHRC1	3.251	Homo sapiens collagen triple helix repeat containing 1 (CTHRC1), mRNA.
NM_000689.3	ALDH1A1	3.250	Homo sapiens aldehyde dehydrogenase 1 family, member A1 (ALDH1A1), mRNA.
NM_004048.2	B2M	3.247	Homo sapiens beta-2-microglobulin (B2M), mRNA.
NM_183079.2	PRNP	3.247	Homo sapiens prion protein (PRNP), transcript variant 2, mRNA.
NM_004252.2	SLC9A3R1	3.246	Homo sapiens solute carrier family 9 (sodium/hydrogen exchanger), member 3 regulator 1 (SLC9A3R1), mRNA.
NM_001040708.1	HEY1	3.244	Homo sapiens hairy/enhancer-of-split related with YRPW motif 1 (HEY1), transcript variant 2, mRNA.
NM_014970.2	KIFAP3	3.244	Homo sapiens kinesin-associated protein 3 (KIFAP3), mRNA.
NM_001012334.1	MDK	3.242	Homo sapiens midkine (neurite growth-promoting factor 2) (MDK), transcript variant 1, mRNA.
NM_144729.1	DUSP10	3.240	Homo sapiens dual specificity phosphatase 10 (DUSP10), transcript variant 3, mRNA.

NM_001620.1	AHNAK	3.229	Homo sapiens AHNAK nucleoprotein (AHNAK), transcript variant 1, mRNA.
NM_024692.3	CLIP4	3.228	Homo sapiens CAP-GLY domain containing linker protein family, member 4 (CLIP4), mRNA.
NM_003316.3	TTC3	3.220	Homo sapiens tetratricopeptide repeat domain 3 (TTC3), transcript variant 1, mRNA.
NM_001099743.1	GOLSYN	3.218	Homo sapiens Golgi-localized protein (GOLSYN), transcript variant 7, mRNA.
NM_000935.2	PLOD2	3.196	Homo sapiens procollagen-lysine, 2-oxoglutarate 5-dioxygenase 2 (PLOD2), transcript variant 2, mRNA.
NM_194071.2	CREB3L2	3.188	Homo sapiens cAMP responsive element binding protein 3-like 2 (CREB3L2), mRNA.
NM_016341.3	PLCE1	3.184	Homo sapiens phospholipase C, epsilon 1 (PLCE1), transcript variant 1, mRNA.
NM_001343.1	DAB2	3.183	Homo sapiens disabled homolog 2, mitogen-responsive phosphoprotein (Drosophila) (DAB2), mRNA.
XM_001723978.1	SPNS2	3.180	PREDICTED: Homo sapiens spinster homolog 2 (Drosophila) (SPNS2), mRNA.
NM_001400.4	S1PR1	3.174	Homo sapiens sphingosine-1-phosphate receptor 1 (S1PR1), mRNA.
NM_145753.1	PHLDB2	3.168	Homo sapiens pleckstrin homology-like domain, family B, member 2 (PHLDB2), mRNA.
NM_018640.3	LMO3	3.164	Homo sapiens LIM domain only 3 (rhombotin-like 2) (LMO3), transcript variant 1, mRNA.
NM_152261.1	C12orf23	3.152	Homo sapiens chromosome 12 open reading frame 23 (C12orf23), mRNA.
NM_138284.1	IL17D	3.148	Homo sapiens interleukin 17D (IL17D), mRNA.
NM_003884.4	KAT2B	3.145	Homo sapiens K(lysine) acetyltransferase 2B (KAT2B), mRNA.
NM_001300.4	KLF6	3.144	Homo sapiens Kruppel-like factor 6 (KLF6), transcript variant 2, mRNA.
XM_001726834.1	LOC100129681	3.143	PREDICTED: Homo sapiens similar to NPC-A-7 (LOC100129681), mRNA.
NM_018222.3	PARVA	3.131	Homo sapiens parvin, alpha (PARVA), mRNA.
NM_003567.2	BCAR3	3.130	Homo sapiens breast cancer anti-estrogen resistance 3 (BCAR3), mRNA.
NM_182943.2	PLOD2	3.130	Homo sapiens procollagen-lysine, 2-oxoglutarate 5-dioxygenase 2 (PLOD2), transcript variant 1, mRNA.
NM_004067.2	CHN2	3.130	Homo sapiens chimerin (chimaerin) 2 (CHN2), transcript variant 2, mRNA.
NM_006053.2	TCIRG1	3.128	Homo sapiens T-cell, immune regulator 1, ATPase, H+ transporting, lysosomal V0 subunit A3 (TCIRG1), transcript variant 2, mRNA.
NM_019554.2	S100A4	3.127	Homo sapiens S100 calcium binding protein A4 (S100A4), transcript variant 2, mRNA.
NM_001669.2	ARSD	3.127	Homo sapiens arylsulfatase D (ARSD), transcript variant 1, mRNA.
NM_001114981.1	TP63	3.124	Homo sapiens tumor protein p63 (TP63), transcript variant 5, mRNA.
NM_006142.3	SFN	3.122	Homo sapiens stratifin (SFN), mRNA.
NM_014674.1	EDEM1	3.119	Homo sapiens ER degradation enhancer, mannosidase alpha-like 1 (EDEM1), mRNA.
NM_175907.3	ZADH2	3.111	Homo sapiens zinc binding alcohol dehydrogenase domain containing 2 (ZADH2), mRNA.
NM_001010990.1	HERPUD1	3.110	Homo sapiens homocysteine-inducible, endoplasmic reticulum stress-inducible, ubiquitin-like domain member 1 (HERPUD1), transcript variant 3, mRNA.
NM_175856.4	CHSY3	3.104	Homo sapiens chondroitin sulfate synthase 3 (CHSY3), mRNA.
NM_000305.2	PON2	3.103	Homo sapiens paraoxonase 2 (PON2), transcript variant 1, mRNA.

NM_032048.2	EMILIN2	3.096	Homo sapiens elastin microfibril interfacer 2 (EMILIN2), mRNA.
NM_175744.4	RHOC	3.095	Homo sapiens ras homolog gene family, member C (RHOC), transcript variant 1, mRNA.
NM_000865.1	HTR1E	3.083	Homo sapiens 5-hydroxytryptamine (serotonin) receptor 1E (HTR1E), mRNA.
NM_002166.4	ID2	3.081	Homo sapiens inhibitor of DNA binding 2, dominant negative helix-loop-helix protein (ID2), mRNA.
NM_001153.2	ANXA4	3.080	Homo sapiens annexin A4 (ANXA4), mRNA.
NM_001010000.1	ARHGAP28	3.076	Homo sapiens Rho GTPase activating protein 28 (ARHGAP28), transcript variant 1, mRNA.
NM_000602.1	SERPINE1	3.076	Homo sapiens serpin peptidase inhibitor, clade E (nexin, plasminogen activator inhibitor type 1), member 1 (SERPINE1), mRNA.
NM_001031615.1	ALDH3B2	3.073	Homo sapiens aldehyde dehydrogenase 3 family, member B2 (ALDH3B2), transcript variant 2, mRNA.
NM_001719.1	BMP7	3.072	Homo sapiens bone morphogenetic protein 7 (osteogenic protein 1) (BMP7), mRNA.
XM_001716667.1	LOC731932	3.061	PREDICTED: Homo sapiens hypothetical LOC731932 (LOC731932), mRNA.
NR_024330.1	NCRNA00085	3.056	Homo sapiens non-protein coding RNA 85 (NCRNA00085), non-coding RNA.
NM_002662.2	PLD1	3.054	Homo sapiens phospholipase D1, phosphatidylcholine-specific (PLD1), mRNA.
NM_032508.1	TMEM185A	3.051	Homo sapiens transmembrane protein 185A (TMEM185A), mRNA.
NM_000846.4	GSTA2	3.050	Homo sapiens glutathione S-transferase alpha 2 (GSTA2), mRNA.
NM_019605.2	SERTAD4	3.050	Homo sapiens SERTA domain containing 4 (SERTAD4), mRNA.
NM_001497.2	B4GALT1	3.050	Homo sapiens UDP-Gal:betaGlcNAc beta 1,4-galactosyltransferase, polypeptide 1 (B4GALT1), mRNA.
NM_198285.1	WDR86	3.048	Homo sapiens WD repeat domain 86 (WDR86), mRNA.
NM_005950.1	MT1G	3.045	Homo sapiens metallothionein 1G (MT1G), mRNA.
NM_001748.3	CAPN2	3.038	Homo sapiens calpain 2, (m/II) large subunit (CAPN2), mRNA.
NM_001018056.1	VLDLR	3.036	Homo sapiens very low density lipoprotein receptor (VLDLR), transcript variant 2, mRNA.
NM_203434.2	IER5L	3.035	Homo sapiens immediate early response 5-like (IER5L), mRNA.
NM_001012513.1	GRP	3.033	Homo sapiens gastrin-releasing peptide (GRP), transcript variant 3, mRNA.
NM_001024455.2	RGAG4	3.030	Homo sapiens retrotransposon gag domain containing 4 (RGAG4), mRNA.
NM_003221.3	TFAP2B	3.030	Homo sapiens transcription factor AP-2 beta (activating enhancer binding protein 2 beta) (TFAP2B), mRNA.
NM_000428.2	LTBP2	3.027	Homo sapiens latent transforming growth factor beta binding protein 2 (LTBP2), mRNA.
NM_005302.2	GPR37	3.027	Homo sapiens G protein-coupled receptor 37 (endothelin receptor type B-like) (GPR37), mRNA.
NM_000693.2	ALDH1A3	3.021	Homo sapiens aldehyde dehydrogenase 1 family, member A3 (ALDH1A3), mRNA.
NM_012098.2	ANGPTL2	3.016	Homo sapiens angiopoietin-like 2 (ANGPTL2), mRNA.
XR_015662.2	LOC731954	3.015	PREDICTED: Homo sapiens similar to supervillin (LOC731954), mRNA.
NM_020197.1	SMYD2	3.014	Homo sapiens SET and MYND domain containing 2 (SMYD2), mRNA.
NM_015059.1	TLN2	3.012	Homo sapiens talin 2 (TLN2), mRNA.
NM_014220.2	TM4SF1	3.006	Homo sapiens transmembrane 4 L six family member 1 (TM4SF1), mRNA.
NM_133486.1	MBNL3	2.995	Homo sapiens muscleblind-like 3 (Drosophila) (MBNL3), transcript variant R, mRNA.

NM_002575.1	SERPINB2	2.993	Homo sapiens serpin peptidase inhibitor, clade B (ovalbumin), member 2 (SERPINB2), mRNA.
NM_001098500.1	KIAA1217	2.993	Homo sapiens KIAA1217 (KIAA1217), transcript variant 2, mRNA.
NM_002051.2	GATA3	2.992	Homo sapiens GATA binding protein 3 (GATA3), transcript variant 2, mRNA.
NM_199138.1	FAM123A	2.989	Homo sapiens family with sequence similarity 123A (FAM123A), transcript variant 2, mRNA.
NM_001204.5	BMPR2	2.988	Homo sapiens bone morphogenetic protein receptor, type II (serine/threonine kinase) (BMPR2), mRNA.
NM_005328.1	HAS2	2.983	Homo sapiens hyaluronan synthase 2 (HAS2), mRNA.
NM_144629.1	RFTN2	2.981	Homo sapiens raftlin family member 2 (RFTN2), mRNA.
NM_003632.1	CNTNAP1	2.981	Homo sapiens contactin associated protein 1 (CNTNAP1), mRNA.
NM_018194.2	HHAT	2.980	Homo sapiens hedgehog acyltransferase (HHAT), mRNA.
XM_001717454.1	KLHL29	2.973	PREDICTED: Homo sapiens kelch-like 29 (Drosophila) (KLHL29), mRNA.
NM_020440.2	PTGFRN	2.968	Homo sapiens prostaglandin F2 receptor negative regulator (PTGFRN), mRNA.
BQ717127		2.968	AGENCOURT_8109349 Lupski_sympathetic_trunk Homo sapiens cDNA clone IMAGE:6189406 5, mRNA sequence
NM_016619.1	PLAC8	2.967	Homo sapiens placenta-specific 8 (PLAC8), mRNA.
NM_005559.2	LAMA1	2.964	Homo sapiens laminin, alpha 1 (LAMA1), mRNA.
NM_001003397.1	TPD52L1	2.957	Homo sapiens tumor protein D52-like 1 (TPD52L1), transcript variant 4, mRNA.
NM_018271.2	FLJ10916	2.949	Homo sapiens hypothetical protein FLJ10916 (FLJ10916), mRNA.
NM_005194.2	CEBPB	2.948	Homo sapiens CCAAT/enhancer binding protein (C/EBP), beta (CEBPB), mRNA.
NM_005860.2	FSTL3	2.945	Homo sapiens follistatin-like 3 (secreted glycoprotein) (FSTL3), mRNA.
NM_016644.1	PRR16	2.943	Homo sapiens proline rich 16 (PRR16), mRNA.
NM_152447.2	LRFN5	2.943	Homo sapiens leucine rich repeat and fibronectin type III domain containing 5 (LRFN5), mRNA.
NM_012307.2	EPB41L3	2.943	Homo sapiens erythrocyte membrane protein band 4.1-like 3 (EPB41L3), mRNA.
NM_002204.1	ITGA3	2.943	Homo sapiens integrin, alpha 3 (antigen CD49C, alpha 3 subunit of VLA-3 receptor) (ITGA3), transcript variant a, mRNA.
BC036485		2.933	Homo sapiens cDNA clone IMAGE:5261213
NM_175738.3	RAB37	2.932	Homo sapiens RAB37, member RAS oncogene family (RAB37), transcript variant 3, mRNA.
XR_017231.2	KRT8P9	2.926	PREDICTED: Homo sapiens misc_RNA (KRT8P9), miscRNA.
NM_015529.2	MOXD1	2.921	Homo sapiens monooxygenase, DBH-like 1 (MOXD1), transcript variant 2, mRNA.
CR627122		2.921	Homo sapiens mRNA; cDNA DKFZp779M2422 (from clone DKFZp779M2422)
NM_001080471.1	PEAR1	2.919	Homo sapiens platelet endothelial aggregation receptor 1 (PEAR1), mRNA.
NM_001024074.1	HNMT	2.918	Homo sapiens histamine N-methyltransferase (HNMT), transcript variant 2, mRNA.
NM_021945.4	C6orf85	2.914	Homo sapiens chromosome 6 open reading frame 85 (C6orf85), mRNA.
NM_000350.2	ABCA4	2.912	Homo sapiens ATP-binding cassette, sub-family A (ABC1), member 4 (ABCA4), mRNA.
NM_003246.2	THBS1	2.911	Homo sapiens thrombospondin 1 (THBS1), mRNA.
NM_004388.1	CTBS	2.907	Homo sapiens chitobiase, di-N-acetyl- (CTBS), mRNA.
XR_040503.1	LOC729680	2.906	PREDICTED: Homo sapiens misc_RNA (LOC729680), miscRNA.
NM_020440.2	PTGFRN	2.903	Homo sapiens prostaglandin F2 receptor negative regulator (PTGFRN), mRNA.
NM_005902.3	SMAD3	2.901	Homo sapiens SMAD family member 3 (SMAD3), transcript

			variant 1, mRNA.
NM_021101.3	CLDN1	2.899	Homo sapiens claudin 1 (CLDN1), mRNA.
NM_015927.3	TGFB1I1	2.899	Homo sapiens transforming growth factor beta 1 induced transcript 1 (TGFB1I1), transcript variant 2, mRNA.
NM_001008539.2	SLC7A2	2.898	Homo sapiens solute carrier family 7 (cationic amino acid transporter, y+ system), member 2 (SLC7A2), transcript variant 2, mRNA.
NM_005518.2	HMGCS2	2.898	Homo sapiens 3-hydroxy-3-methylglutaryl-Coenzyme A synthase 2 (mitochondrial) (HMGCS2), nuclear gene encoding mitochondrial protein, mRNA.
NM_172060.1	EYA1	2.897	Homo sapiens eyes absent homolog 1 (Drosophila) (EYA1), transcript variant 1, mRNA.
NM_020251.2	ARRB1	2.896	Homo sapiens arrestin, beta 1 (ARRB1), transcript variant 2, mRNA.
NM_001221.2	CAMK2D	2.895	Homo sapiens calcium/calmodulin-dependent protein kinase (CaM kinase) II delta (CAMK2D), transcript variant 3, mRNA.
NM_014057.3	OGN	2.890	Homo sapiens osteoglycin (OGN), transcript variant 3, mRNA.
NM_033394.1	TANC1	2.887	Homo sapiens tetratricopeptide repeat, ankyrin repeat and coiled-coil containing 1 (TANC1), mRNA.
NM_013379.2	DPP7	2.887	Homo sapiens dipeptidyl-peptidase 7 (DPP7), mRNA.
NM_138991.1	BACE2	2.885	Homo sapiens beta-site APP-cleaving enzyme 2 (BACE2), transcript variant c, mRNA.
NM_002184.2	IL6ST	2.883	Homo sapiens interleukin 6 signal transducer (gp130, oncostatin M receptor) (IL6ST), transcript variant 1, mRNA.
NM_015917.1	GSTK1	2.882	Homo sapiens glutathione S-transferase kappa 1 (GSTK1), mRNA.
NM_001616.3	ACVR2A	2.877	Homo sapiens activin A receptor, type IIA (ACVR2A), mRNA.
NM_018438.4	FBXO6	2.877	Homo sapiens F-box protein 6 (FBXO6), mRNA.
NM_001993.2	F3	2.876	Homo sapiens coagulation factor III (thromboplastin, tissue factor) (F3), mRNA.
NM_015374.1	UNC84B	2.868	Homo sapiens unc-84 homolog B (C. elegans) (UNC84B), mRNA.
NM_001042442.1	CAST	2.868	Homo sapiens calpastatin (CAST), transcript variant 8, mRNA.
NM_006982.2	ALX1	2.864	Homo sapiens ALX homeobox 1 (ALX1), mRNA.
NM_000203.3	IDUA	2.863	Homo sapiens iduronidase, alpha-L- (IDUA), mRNA.
NM_007310.1	COMT	2.862	Homo sapiens catechol-O-methyltransferase (COMT), transcript variant S-COMT, mRNA.
NM_178497.2	C4orf26	2.861	Homo sapiens chromosome 4 open reading frame 26 (C4orf26), mRNA.
NM_001007236.1	ERVK6	2.859	Homo sapiens endogenous retroviral sequence K, 6 (ERVK6), mRNA.
NM_018327.2	SPTLC3	2.858	Homo sapiens serine palmitoyltransferase, long chain base subunit 3 (SPTLC3), mRNA.
NM_021070.2	LTBP3	2.857	Homo sapiens latent transforming growth factor beta binding protein 3 (LTBP3), mRNA.
NM_001995.2	ACSL1	2.854	Homo sapiens acyl-CoA synthetase long-chain family member 1 (ACSL1), mRNA.
NM_004335.2	BST2	2.850	Homo sapiens bone marrow stromal cell antigen 2 (BST2), mRNA.
NM_001924.2	GADD45A	2.849	Homo sapiens growth arrest and DNA-damage-inducible, alpha (GADD45A), mRNA.
NM_005046.2	KLK7	2.844	Homo sapiens kallikrein-related peptidase 7 (KLK7), transcript variant 1, mRNA.
NM_178314.2	RILPL1	2.840	Homo sapiens Rab interacting lysosomal protein-like 1 (RILPL1), mRNA.
NM_201554.1	DGKA	2.840	Homo sapiens diacylglycerol kinase, alpha 80kDa (DGKA), transcript variant 4, mRNA.
NM_030672.2	ARHGAP28	2.839	Homo sapiens Rho GTPase activating protein 28 (ARHGAP28), transcript variant 2, mRNA.
NM_182676.1	PLTP	2.836	Homo sapiens phospholipid transfer protein (PLTP), transcript

			variant 2, mRNA.
NM_006760.2	UPK2	2.835	Homo sapiens uroplakin 2 (UPK2), mRNA.
NM_003078.3	SMARCD3	2.830	Homo sapiens SWI/SNF related, matrix associated, actin dependent regulator of chromatin, subfamily d, member 3 (SMARCD3), transcript variant 2, mRNA.
NM_001040058.1	SPP1	2.830	Homo sapiens secreted phosphoprotein 1 (SPP1), transcript variant 1, mRNA.
NM_152509.1	FLJ31568	2.827	Homo sapiens FLJ31568 protein (FLJ31568), mRNA.
NM_015881.5	DKK3	2.823	Homo sapiens dickkopf homolog 3 (Xenopus laevis) (DKK3), transcript variant 1, mRNA.
NM_004173.2	SLC7A4	2.818	Homo sapiens solute carrier family 7 (cationic amino acid transporter, y+ system), member 4 (SLC7A4), mRNA.
NM_022168.2	IFIH1	2.817	Homo sapiens interferon induced with helicase C domain 1 (IFIH1), mRNA.
NM_001009934.1	DNASE1L1	2.811	Homo sapiens deoxyribonuclease I-like 1 (DNASE1L1), transcript variant 4, mRNA.
AL833463		2.810	Homo sapiens mRNA; cDNA DKFZp686P07116 (from clone DKFZp686P07116)
NM_134264.2	WSB1	2.808	Homo sapiens WD repeat and SOCS box-containing 1 (WSB1), transcript variant 3, mRNA.
NM_005951.2	MT1H	2.806	Homo sapiens metallothionein 1H (MT1H), mRNA.
NM_001754.3	RUNX1	2.803	Homo sapiens runt-related transcription factor 1 (acute myeloid leukemia 1; aml1 oncogene) (RUNX1), transcript variant 1, mRNA.
NM_002430.2	MN1	2.801	Homo sapiens meningioma (disrupted in balanced translocation) 1 (MN1), mRNA.
NM_145753.1	PHLDB2	2.800	Homo sapiens pleckstrin homology-like domain, family B, member 2 (PHLDB2), mRNA.
XM_939337.2	RBM20	2.797	PREDICTED: Homo sapiens RNA binding motif protein 20 (RBM20), mRNA.
NM_001114734.1	PABPC4L	2.795	Homo sapiens poly(A) binding protein, cytoplasmic 4-like (PABPC4L), mRNA.
NM_052880.3	PIK3IP1	2.793	Homo sapiens phosphoinositide-3-kinase interacting protein 1 (PIK3IP1), mRNA.
NM_000676.2	ADORA2B	2.791	Homo sapiens adenosine A2b receptor (ADORA2B), mRNA.
NM_001024912.1	CEACAM1	2.791	Homo sapiens carcinoembryonic antigen-related cell adhesion molecule 1 (biliary glycoprotein) (CEACAM1), transcript variant 2, mRNA.
NM_024060.2	AHNAK	2.790	Homo sapiens AHNAK nucleoprotein (AHNAK), transcript variant 2, mRNA.
NM_001560.2	IL13RA1	2.789	Homo sapiens interleukin 13 receptor, alpha 1 (IL13RA1), mRNA.
NM_020387.2	RAB25	2.789	Homo sapiens RAB25, member RAS oncogene family (RAB25), mRNA.
NM_020387.2	RAB25	2.789	Homo sapiens RAB25, member RAS oncogene family (RAB25), mRNA.
NM_003759.2	SLC4A4	2.784	Homo sapiens solute carrier family 4, sodium bicarbonate cotransporter, member 4 (SLC4A4), transcript variant 2, mRNA.
NM_004052.2	BNIP3	2.784	Homo sapiens BCL2/adenovirus E1B 19kDa interacting protein 3 (BNIP3), nuclear gene encoding mitochondrial protein, mRNA.
NM_024866.4	ADM2	2.782	Homo sapiens adrenomedullin 2 (ADM2), mRNA.
NM_004560.2	ROR2	2.778	Homo sapiens receptor tyrosine kinase-like orphan receptor 2 (ROR2), mRNA.
NM_001018065.1	NTRK2	2.777	Homo sapiens neurotrophic tyrosine kinase, receptor, type 2 (NTRK2), transcript variant d, mRNA.
NM_000717.2	CA4	2.774	Homo sapiens carbonic anhydrase IV (CA4), mRNA.
NM_004172.3	SLC1A3	2.774	Homo sapiens solute carrier family 1 (glial high affinity glutamate transporter), member 3 (SLC1A3), mRNA.
XM_001129441.2	LOC645166	2.773	PREDICTED: Homo sapiens similar to lymphocyte-specific protein

			1 (LOC645166), mRNA.
NM_199069.1	NDUFAF3	2.773	Homo sapiens NADH dehydrogenase (ubiquinone) 1 alpha subcomplex, assembly factor 3 (NDUFAF3), nuclear gene encoding mitochondrial protein, transcript variant 1, mRNA.
NM_178156.1	FUT8	2.772	Homo sapiens fucosyltransferase 8 (alpha (1,6) fucosyltransferase) (FUT8), transcript variant 3, mRNA.
NM_182760.2	SUMF1	2.769	Homo sapiens sulfatase modifying factor 1 (SUMF1), mRNA.
NM_024935.2	KIAA1772	2.769	Homo sapiens KIAA1772 (KIAA1772), mRNA.
NM_004443.3	EPHB3	2.763	Homo sapiens EPH receptor B3 (EPHB3), mRNA.
AB074162		2.762	Homo sapiens primary neuroblastoma cDNA, clone:Nbla10527, full insert sequence
NM_001042443.1	CAST	2.762	Homo sapiens calpastatin (CAST), transcript variant 9, mRNA.
NM_006042.1	HS3ST3A1	2.761	Homo sapiens heparan sulfate (glucosamine) 3-O-sulfotransferase 3A1 (HS3ST3A1), mRNA.
NM_006522.3	WNT6	2.760	Homo sapiens wingless-type MMTV integration site family, member 6 (WNT6), mRNA.
NM_001137601.1	ZBTB42	2.759	Homo sapiens zinc finger and BTB domain containing 42 (ZBTB42), mRNA.
NM_001924.2	GADD45A	2.759	Homo sapiens growth arrest and DNA-damage-inducible, alpha (GADD45A), mRNA.
NM_080815.2	COL13A1	2.755	Homo sapiens collagen, type XIII, alpha 1 (COL13A1), transcript variant 19, mRNA.
NM_017983.4	WIPI1	2.754	Homo sapiens WD repeat domain, phosphoinositide interacting 1 (WIPI1), mRNA.
NM_080829.2	FAM65C	2.752	Homo sapiens family with sequence similarity 65, member C (FAM65C), mRNA.
NM_001615.3	ACTG2	2.751	Homo sapiens actin, gamma 2, smooth muscle, enteric (ACTG2), mRNA.
NM_152423.2	MUM1L1	2.750	Homo sapiens melanoma associated antigen (mutated) 1-like 1 (MUM1L1), mRNA.
NM_031439.2	SOX7	2.750	Homo sapiens SRY (sex determining region Y)-box 7 (SOX7), mRNA.
NM_005491.2	MAMLD1	2.750	Homo sapiens mastermind-like domain containing 1 (MAMLD1), mRNA.
NM_025106.2	SPSB1	2.748	Homo sapiens splA/ryanodine receptor domain and SOCS box containing 1 (SPSB1), mRNA.
NM_181533.3	ABHD12B	2.745	Homo sapiens abhydrolase domain containing 12B (ABHD12B), transcript variant 2, mRNA.
NM_001077188.1	HS6ST2	2.744	Homo sapiens heparan sulfate 6-O-sulfotransferase 2 (HS6ST2), transcript variant L, mRNA.
NM_006645.2	STARD10	2.743	Homo sapiens StAR-related lipid transfer (START) domain containing 10 (STARD10), mRNA.
NM_001804.2	CDX1	2.741	Homo sapiens caudal type homeobox 1 (CDX1), mRNA.
AL157484		2.740	Homo sapiens mRNA; cDNA DKFZp762M127 (from clone DKFZp762M127)
NM_006854.3	KDELR2	2.736	Homo sapiens KDEL (Lys-Asp-Glu-Leu) endoplasmic reticulum protein retention receptor 2 (KDELR2), transcript variant 1, mRNA.
NM_000305.2	PON2	2.733	Homo sapiens paraoxonase 2 (PON2), transcript variant 1, mRNA.
NM_012343.3	NNT	2.727	Homo sapiens nicotinamide nucleotide transhydrogenase (NNT), nuclear gene encoding mitochondrial protein, transcript variant 1, mRNA.
XM_944321.1	LOC402560	2.726	PREDICTED: Homo sapiens hypothetical LOC402560 (LOC402560), mRNA.
NM_002633.2	PGM1	2.725	Homo sapiens phosphoglucomutase 1 (PGM1), mRNA.
NM_013271.2	PCSK1N	2.724	Homo sapiens proprotein convertase subtilisin/kexin type 1 inhibitor (PCSK1N), mRNA.

NM_173354.3	SIK1	2.719	Homo sapiens salt-inducible kinase 1 (SIK1), mRNA.
XM_001724386.1	LOC100134265	2.716	PREDICTED: Homo sapiens similar to calbindin 2 full length protein (LOC100134265), mRNA.
NM_005559.2	LAMA1	2.713	Homo sapiens laminin, alpha 1 (LAMA1), mRNA.
NM_004126.3	GNG11	2.710	Homo sapiens guanine nucleotide binding protein (G protein), gamma 11 (GNG11), mRNA.
NM_001005242.1	PKP2	2.708	Homo sapiens plakophilin 2 (PKP2), transcript variant 2a, mRNA.
NM_004598.3	SPOCK1	2.707	Homo sapiens sparc/osteonectin, cwcv and kazal-like domains proteoglycan (testican) 1 (SPOCK1), mRNA.
NM_015274.1	MAN2B2	2.706	Homo sapiens mannosidase, alpha, class 2B, member 2 (MAN2B2), mRNA.
NM_021972.2	SPHK1	2.704	Homo sapiens sphingosine kinase 1 (SPHK1), transcript variant 1, mRNA.
NM_002614.3	PDZK1	2.704	Homo sapiens PDZ domain containing 1 (PDZK1), mRNA.
NM_024605.3	ARHGAP10	2.703	Homo sapiens Rho GTPase activating protein 10 (ARHGAP10), mRNA.
NM_198392.1	TCF21	2.702	Homo sapiens transcription factor 21 (TCF21), transcript variant 1, mRNA.
NM_198951.1	TGM2	2.701	Homo sapiens transglutaminase 2 (C polypeptide, protein-glutamine-gamma-glutamyltransferase) (TGM2), transcript variant 2, mRNA.
NM_001037633.1	SIL1	2.700	Homo sapiens SIL1 homolog, endoplasmic reticulum chaperone (S. cerevisiae) (SIL1), transcript variant 1, mRNA.
NM_181501.1	ITGA1	2.698	Homo sapiens integrin, alpha 1 (ITGA1), mRNA.
NM_000935.2	PLOD2	2.695	Homo sapiens procollagen-lysine, 2-oxoglutarate 5-dioxygenase 2 (PLOD2), transcript variant 2, mRNA.
NM_199181.2	C20orf117	2.692	Homo sapiens chromosome 20 open reading frame 117 (C20orf117), transcript variant 2, mRNA.
NM_152629.3	GLIS3	2.690	Homo sapiens GLIS family zinc finger 3 (GLIS3), transcript variant 2, mRNA.
AK123807		2.688	Homo sapiens cDNA FLJ41813 fis, clone NT2RI2011450
NM_032305.1	POLR3GL	2.687	Homo sapiens polymerase (RNA) III (DNA directed) polypeptide G (32kD)-like (POLR3GL), mRNA.
NM_152387.2	KCTD18	2.682	Homo sapiens potassium channel tetramerisation domain containing 18 (KCTD18), mRNA.
NM_133436.1	ASNS	2.675	Homo sapiens asparagine synthetase (ASNS), transcript variant 1, mRNA.
NM_005141.2	FGB	2.674	Homo sapiens fibrinogen beta chain (FGB), mRNA.
NM_014745.1	FAM38A	2.666	Homo sapiens family with sequence similarity 38, member A (FAM38A), mRNA.
NM_021034.2	IFITM3	2.666	Homo sapiens interferon induced transmembrane protein 3 (1-8U) (IFITM3), mRNA.
XR_017689.1	KRT18P28	2.664	PREDICTED: Homo sapiens misc_RNA (KRT18P28), miscRNA.
NM_053032.2	MYLK	2.662	Homo sapiens myosin light chain kinase (MYLK), transcript variant 8, mRNA.
NM_175617.3	MT1E	2.661	Homo sapiens metallothionein 1E (MT1E), mRNA.
NM_004099.4	STOM	2.661	Homo sapiens stomatin (STOM), transcript variant 1, mRNA.
NM_001024630.2	RUNX2	2.661	Homo sapiens runt-related transcription factor 2 (RUNX2), transcript variant 1, mRNA.
NM_130435.2	PTPRE	2.659	Homo sapiens protein tyrosine phosphatase, receptor type, E (PTPRE), transcript variant 2, mRNA.
NM_001012334.1	MDK	2.657	Homo sapiens midkine (neurite growth-promoting factor 2) (MDK), transcript variant 1, mRNA.
NM_007029.2	STMN2	2.657	Homo sapiens stathmin-like 2 (STMN2), mRNA.
NM_001604.3	PAX6	2.656	Homo sapiens paired box 6 (PAX6), transcript variant 2, mRNA.
NM_003480.2	MFAP5	2.653	Homo sapiens microfibrillar associated protein 5 (MFAP5), mRNA.
NM_013995.1	LAMP2	2.653	Homo sapiens lysosomal-associated membrane protein 2

			(LAMP2), transcript variant LAMP2B, mRNA.
NM_020433.4	JPH2	2.653	Homo sapiens junctophilin 2 (JPH2), transcript variant 1, mRNA.
NM_014476.1	PDLIM3	2.653	Homo sapiens PDZ and LIM domain 3 (PDLIM3), mRNA.
NM_001032394.1	GPR126	2.651	Homo sapiens G protein-coupled receptor 126 (GPR126), transcript variant a2, mRNA.
NM_198291.1	SRC	2.649	Homo sapiens v-src sarcoma (Schmidt-Ruppin A-2) viral oncogene homolog (avian) (SRC), transcript variant 2, mRNA.
NM_139289.1	AKAP4	2.647	Homo sapiens A kinase (PRKA) anchor protein 4 (AKAP4), transcript variant 2, mRNA.
NM_001001870.1	C17orf91	2.646	Homo sapiens chromosome 17 open reading frame 91 (C17orf91), transcript variant 2, mRNA.
NM_178006.1	STARD13	2.645	Homo sapiens START domain containing 13 (STARD13), transcript variant alpha, mRNA.
NM_016243.2	CYB5R1	2.644	Homo sapiens cytochrome b5 reductase 1 (CYB5R1), mRNA.
NM_199168.2	CXCL12	2.642	Homo sapiens chemokine (C-X-C motif) ligand 12 (stromal cell-derived factor 1) (CXCL12), transcript variant 1, mRNA.
NM_000714.4	TSPO	2.641	Homo sapiens translocator protein (18kDa) (TSPO), transcript variant PBR, mRNA.
NM_000930.2	PLAT	2.641	Homo sapiens plasminogen activator, tissue (PLAT), transcript variant 1, mRNA.
NM_001129.3	AEBP1	2.641	Homo sapiens AE binding protein 1 (AEBP1), mRNA.
NM_020992.2	PDLIM1	2.641	Homo sapiens PDZ and LIM domain 1 (PDLIM1), mRNA.
NM_001001787.1	ATP1B1	2.639	Homo sapiens ATPase, Na+/K+ transporting, beta 1 polypeptide (ATP1B1), transcript variant 2, mRNA.
NM_006407.3	ARL6IP5	2.636	Homo sapiens ADP-ribosylation-like factor 6 interacting protein 5 (ARL6IP5), mRNA.
NM_002506.2	NGF	2.636	Homo sapiens nerve growth factor (beta polypeptide) (NGF), mRNA.
NM_005946.2	MT1A	2.635	Homo sapiens metallothionein 1A (MT1A), mRNA.
NM_005257.3	GATA6	2.633	Homo sapiens GATA binding protein 6 (GATA6), mRNA.
NR_024618.1	LOC100129550	2.627	Homo sapiens hypothetical LOC100129550 (LOC100129550), non-coding RNA.
NM_003122.2	SPINK1	2.623	Homo sapiens serine peptidase inhibitor, Kazal type 1 (SPINK1), mRNA.
NM_001077593.1	ADHFE1	2.623	Homo sapiens alcohol dehydrogenase, iron containing, 1 (ADHFE1), transcript variant 1, mRNA.
NM_002166.4	ID2	2.619	Homo sapiens inhibitor of DNA binding 2, dominant negative helix-loop-helix protein (ID2), mRNA.
NM_003377.3	VEGFB	2.613	Homo sapiens vascular endothelial growth factor B (VEGFB), mRNA.
NM_006825.2	CKAP4	2.612	Homo sapiens cytoskeleton-associated protein 4 (CKAP4), mRNA.
NM_003816.2	ADAM9	2.610	Homo sapiens ADAM metallopeptidase domain 9 (meltrin gamma) (ADAM9), transcript variant 1, mRNA.
NM_000391.3	TPP1	2.603	Homo sapiens tripeptidyl peptidase I (TPP1), mRNA.
NR_029622.1	MIR205	2.600	Homo sapiens microRNA 205 (MIR205), microRNA.
NM_022486.3	SUSD1	2.599	Homo sapiens sushi domain containing 1 (SUSD1), mRNA.
NM_014764.2	DAZAP2	2.599	Homo sapiens DAZ associated protein 2 (DAZAP2), mRNA.
NM_013253.4	DKK3	2.599	Homo sapiens dickkopf homolog 3 (Xenopus laevis) (DKK3), transcript variant 2, mRNA.
NM_005241.1	EVI1	2.594	Homo sapiens ecotropic viral integration site 1 (EVI1), mRNA.
NM_000302.2	PLOD1	2.586	Homo sapiens procollagen-lysine 1, 2-oxoglutarate 5-dioxygenase 1 (PLOD1), mRNA.
NM_080760.3	DACH1	2.585	Homo sapiens dachshund homolog 1 (Drosophila) (DACH1), transcript variant 2, mRNA.
NM_152680.1	TMEM154	2.584	Homo sapiens transmembrane protein 154 (TMEM154), mRNA.
NM_002033.2	FUT4	2.582	Homo sapiens fucosyltransferase 4 (alpha (1,3) fucosyltransferase, myeloid-specific) (FUT4), mRNA.

NM_032508.1	TMEM185A	2.579	Homo sapiens transmembrane protein 185A (TMEM185A), mRNA.
NM_025185.3	TANC2	2.576	Homo sapiens tetratricopeptide repeat, ankyrin repeat and coiled-coil containing 2 (TANC2), mRNA.
NM_020998.2	MST1	2.576	Homo sapiens macrophage stimulating 1 (hepatocyte growth factor-like) (MST1), mRNA.
NM_005067.5	SIAH2	2.575	Homo sapiens seven in absentia homolog 2 (Drosophila) (SIAH2), mRNA.
NM_024767.2	DLC1	2.575	Homo sapiens deleted in liver cancer 1 (DLC1), transcript variant 3, mRNA.
NM_031305.2	ARHGAP24	2.575	Homo sapiens Rho GTPase activating protein 24 (ARHGAP24), transcript variant 2, mRNA.
NM_018416.2	FOXJ2	2.571	Homo sapiens forkhead box J2 (FOXJ2), mRNA.
NM_002555.3	SLC22A18	2.567	Homo sapiens solute carrier family 22 (organic cation transporter), member 18 (SLC22A18), transcript variant 1, mRNA.
NM_005980.2	S100P	2.563	Homo sapiens S100 calcium binding protein P (S100P), mRNA.
NM_000846.3	GSTA2	2.563	Homo sapiens glutathione S-transferase A2 (GSTA2), mRNA.
NM_001007593.1	SMPD1	2.562	Homo sapiens sphingomyelin phosphodiesterase 1, acid lysosomal (SMPD1), transcript variant ASM-2, mRNA.
NM_024302.3	MMP28	2.560	Homo sapiens matrix metallopeptidase 28 (MMP28), transcript variant 1, mRNA.
NM_004099.4	STOM	2.555	Homo sapiens stomatin (STOM), transcript variant 1, mRNA.
NM_014622.4	VWA5A	2.546	Homo sapiens von Willebrand factor A domain containing 5A (VWA5A), transcript variant 1, mRNA.
NM_182507.2	KRT80	2.546	Homo sapiens keratin 80 (KRT80), transcript variant 1, mRNA.
NM_001003845.1	SP5	2.545	Homo sapiens Sp5 transcription factor (SP5), mRNA.
NM_015286.5	SYNM	2.544	Homo sapiens synemin, intermediate filament protein (SYNM), transcript variant B, mRNA.
NM_177925.2	H2AFJ	2.542	Homo sapiens H2A histone family, member J (H2AFJ), transcript variant 1, mRNA.
NM_005308.2	GRK5	2.540	Homo sapiens G protein-coupled receptor kinase 5 (GRK5), mRNA.
NM_002659.2	PLAUR	2.539	Homo sapiens plasminogen activator, urokinase receptor (PLAUR), transcript variant 1, mRNA.
NM_018327.2	SPTLC3	2.538	Homo sapiens serine palmitoyltransferase, long chain base subunit 3 (SPTLC3), mRNA.
NM_005178.2	BCL3	2.536	Homo sapiens B-cell CLL/lymphoma 3 (BCL3), mRNA.
NM_021977.2	SLC22A3	2.534	Homo sapiens solute carrier family 22 (extraneuronal monoamine transporter), member 3 (SLC22A3), mRNA.
XR_037953.1	KRT18P17	2.533	PREDICTED: Homo sapiens misc_RNA (KRT18P17), miscRNA.
NM_007003.2	PAGE4	2.531	Homo sapiens P antigen family, member 4 (prostate associated) (PAGE4), mRNA.
BX640888		2.529	Homo sapiens mRNA; cDNA DKFZp686H20120 (from clone DKFZp686H20120)
NM_030634.2	ZNF436	2.527	Homo sapiens zinc finger protein 436 (ZNF436), transcript variant 2, mRNA.
NM_130811.1	SNAP25	2.526	Homo sapiens synaptosomal-associated protein, 25kDa (SNAP25), transcript variant 2, mRNA.
NM_025135.2	FHOD3	2.523	Homo sapiens formin homology 2 domain containing 3 (FHOD3), mRNA.
NM_005775.3	SORBS3	2.522	Homo sapiens sorbin and SH3 domain containing 3 (SORBS3), transcript variant 1, mRNA.
NM_018300.2	ZNF83	2.521	Homo sapiens zinc finger protein 83 (ZNF83), mRNA.
NM_024843.2	CYBRD1	2.519	Homo sapiens cytochrome b reductase 1 (CYBRD1), mRNA.
NM_152392.2	AHSA2	2.516	Homo sapiens AHA1, activator of heat shock 90kDa protein ATPase homolog 2 (yeast) (AHSA2), mRNA.
NM_002076.2	GNS	2.515	Homo sapiens glucosamine (N-acetyl)-6-sulfatase (Sanfilippo

			disease IIID) (GNS), mRNA.
NM_022769.3	CRTC3	2.515	Homo sapiens CREB regulated transcription coactivator 3 (CRTC3), transcript variant 1, mRNA.
NM_021180.2	GRHL3	2.515	Homo sapiens grainyhead-like 3 (Drosophila) (GRHL3), transcript variant 1, mRNA.
NM_005780.1	LHFP	2.515	Homo sapiens lipoma HMGIC fusion partner (LHFP), mRNA.
XM_001726946.1	NBPF8	2.514	PREDICTED: Homo sapiens neuroblastoma breakpoint family, member 8 (NBPF8), mRNA.
NM_006403.2	NEDD9	2.514	Homo sapiens neural precursor cell expressed, developmentally down-regulated 9 (NEDD9), transcript variant 1, mRNA.
NM_001014279.1	C5orf39	2.513	Homo sapiens chromosome 5 open reading frame 39 (C5orf39), mRNA.
NM_005398.4	PPP1R3C	2.513	Homo sapiens protein phosphatase 1, regulatory (inhibitor) subunit 3C (PPP1R3C), mRNA.
NM_012343.3	NNT	2.512	Homo sapiens nicotinamide nucleotide transhydrogenase (NNT), nuclear gene encoding mitochondrial protein, transcript variant 1, mRNA.
NM_001105079.1	FBRS	2.512	Homo sapiens fibrosin (FBRS), mRNA.
NM_001031717.2	CRELD1	2.512	Homo sapiens cysteine-rich with EGF-like domains 1 (CRELD1), transcript variant 1, mRNA.
NM_014476.1	PDLIM3	2.510	Homo sapiens PDZ and LIM domain 3 (PDLIM3), mRNA.
NM_006537.2	USP3	2.510	Homo sapiens ubiquitin specific peptidase 3 (USP3), mRNA.
NM_001735.2	C5	2.509	Homo sapiens complement component 5 (C5), mRNA.
NM_020766.1	PCDH19	2.508	Homo sapiens protocadherin 19 (PCDH19), mRNA.
NM_001204.5	BMPR2	2.507	Homo sapiens bone morphogenetic protein receptor, type II (serine/threonine kinase) (BMPR2), mRNA.
NM_153756.1	FNDC5	2.504	Homo sapiens fibronectin type III domain containing 5 (FNDC5), mRNA.
NM_022763.3	FNDC3B	2.504	Homo sapiens fibronectin type III domain containing 3B (FNDC3B), transcript variant 1, mRNA.
NM_001248.1	ENTPD3	2.504	Homo sapiens ectonucleoside triphosphate diphosphohydrolase 3 (ENTPD3), mRNA.
NM_177444.1	PPFIBP1	2.501	Homo sapiens PTPRF interacting protein, binding protein 1 (liprin beta 1) (PPFIBP1), transcript variant 2, mRNA.
NM_138972.2	BACE1	2.501	Homo sapiens beta-site APP-cleaving enzyme 1 (BACE1), transcript variant b, mRNA.
NM_004067.2	CHN2	2.497	Homo sapiens chimerin (chimaerin) 2 (CHN2), transcript variant 2, mRNA.
NM_006379.2	SEMA3C	2.496	Homo sapiens sema domain, immunoglobulin domain (Ig), short basic domain, secreted, (semaphorin) 3C (SEMA3C), mRNA.
NM_005651.1	TDO2	2.495	Homo sapiens tryptophan 2,3-dioxygenase (TDO2), mRNA.
NM_015482.1	SLC22A23	2.494	Homo sapiens solute carrier family 22, member 23 (SLC22A23), transcript variant 1, mRNA.
NR_002797.1	LOC255783	2.493	Homo sapiens hypothetical protein LOC255783 (LOC255783), non-coding RNA.
NM_000047.1	ARSE	2.492	Homo sapiens arylsulfatase E (chondrodysplasia punctata 1) (ARSE), mRNA.
NM_018342.3	TMEM144	2.491	Homo sapiens transmembrane protein 144 (TMEM144), mRNA.
NM_130435.2	PTPRE	2.487	Homo sapiens protein tyrosine phosphatase, receptor type, E (PTPRE), transcript variant 2, mRNA.
NM_001095.2	ACCN2	2.483	Homo sapiens amiloride-sensitive cation channel 2, neuronal (ACCN2), transcript variant 2, mRNA.
NM_031439.2	SOX7	2.482	Homo sapiens SRY (sex determining region Y)-box 7 (SOX7), mRNA.
NM_002210.2	ITGAV	2.482	Homo sapiens integrin, alpha V (vitronectin receptor, alpha polypeptide, antigen CD51) (ITGAV), mRNA.
NM_006763.2	BTG2	2.481	Homo sapiens BTG family, member 2 (BTG2), mRNA.

NM_020338.2	ZMIZ1	2.475	Homo sapiens zinc finger, MIZ-type containing 1 (ZMIZ1), mRNA.
NM_000817.2	GAD1	2.475	Homo sapiens glutamate decarboxylase 1 (brain, 67kDa) (GAD1), transcript variant GAD67, mRNA.
NM_030799.6	YIPF5	2.473	Homo sapiens Yip1 domain family, member 5 (YIPF5), transcript variant 2, mRNA.
NM_000156.4	GAMT	2.471	Homo sapiens guanidinoacetate N-methyltransferase (GAMT), transcript variant 1, mRNA.
NM_017709.3	FAM46C	2.468	Homo sapiens family with sequence similarity 46, member C (FAM46C), mRNA.
NM_006092.1	NOD1	2.466	Homo sapiens nucleotide-binding oligomerization domain containing 1 (NOD1), mRNA.
NM_005895.3	GOLGA3	2.465	Homo sapiens golgi autoantigen, golgin subfamily a, 3 (GOLGA3), mRNA.
NM_178422.4	PAQR7	2.463	Homo sapiens progestin and adipoQ receptor family member VII (PAQR7), mRNA.
NM_015150.1	RFTN1	2.461	Homo sapiens raftlin, lipid raft linker 1 (RFTN1), mRNA.
NM_018952.4	HOXB6	2.460	Homo sapiens homeobox B6 (HOXB6), mRNA.
NM_144642.3	SYNPR	2.460	Homo sapiens synaptoporin (SYNPR), mRNA.
NM_139125.2	MASP1	2.459	Homo sapiens mannan-binding lectin serine peptidase 1 (C4/C2 activating component of Ra-reactive factor) (MASP1), transcript variant 2, mRNA.
NM_013401.2	RAB3IL1	2.452	Homo sapiens RAB3A interacting protein (rabin3)-like 1 (RAB3IL1), mRNA.
NM_004354.1	CCNG2	2.452	Homo sapiens cyclin G2 (CCNG2), mRNA.
NM_031458.1	PARP9	2.452	Homo sapiens poly (ADP-ribose) polymerase family, member 9 (PARP9), mRNA.
NM_152996.1	ST6GALNAC3	2.450	Homo sapiens ST6 (alpha-N-acetyl-neuraminyl-2,3-beta-galactosyl-1, 3)-N-acetylgalactosaminide alpha-2,6-sialyltransferase 3 (ST6GALNAC3), mRNA.
NM_002198.1	IRF1	2.449	Homo sapiens interferon regulatory factor 1 (IRF1), mRNA.
NM_002100.3	GYPB	2.446	Homo sapiens glycophorin B (MNS blood group) (GYPB), mRNA.
NM_152330.2	FRMD6	2.445	Homo sapiens FERM domain containing 6 (FRMD6), mRNA.
NM_014909.3	VASH1	2.444	Homo sapiens vasohibin 1 (VASH1), mRNA.
NM_012435.1	SHC2	2.441	Homo sapiens SHC (Src homology 2 domain containing) transforming protein 2 (SHC2), mRNA.
NM_003222.3	TFAP2C	2.441	Homo sapiens transcription factor AP-2 gamma (activating enhancer binding protein 2 gamma) (TFAP2C), mRNA.
NM_001882.3	CRHBP	2.440	Homo sapiens corticotropin releasing hormone binding protein (CRHBP), mRNA.
NM_138690.1	GRIN3B	2.439	Homo sapiens glutamate receptor, ionotropic, N-methyl-D-aspartate 3B (GRIN3B), mRNA.
NM_201554.1	DGKA	2.436	Homo sapiens diacylglycerol kinase, alpha 80kDa (DGKA), transcript variant 4, mRNA.
NM_005347.2	HSPA5	2.435	Homo sapiens heat shock 70kDa protein 5 (glucose-regulated protein, 78kDa) (HSPA5), mRNA.
NM_153367.2	ZCCHC24	2.435	Homo sapiens zinc finger, CCHC domain containing 24 (ZCCHC24), mRNA.
NM_002413.3	MGST2	2.433	Homo sapiens microsomal glutathione S-transferase 2 (MGST2), mRNA.
NM_018166.1	FAM176B	2.433	Homo sapiens family with sequence similarity 176, member B (FAM176B), mRNA.
NM_000127.2	EXT1	2.428	Homo sapiens exostoses (multiple) 1 (EXT1), mRNA.
NM_016657.1	KDELR3	2.425	Homo sapiens KDEL (Lys-Asp-Glu-Leu) endoplasmic reticulum protein retention receptor 3 (KDELR3), transcript variant 2, mRNA.
NM_022572.2	PNKD	2.422	Homo sapiens paroxysmal nonkinesiogenic dyskinesia (PNKD), transcript variant 2, mRNA.

NM_001002857.1	ANXA2	2.420	Homo sapiens annexin A2 (ANXA2), transcript variant 2, mRNA.
NM_001007189.1	C5orf53	2.416	Homo sapiens chromosome 5 open reading frame 53 (C5orf53), mRNA.
NM_032854.2	CORO6	2.415	Homo sapiens coronin 6 (CORO6), mRNA.
NM_170677.2	MEIS2	2.414	Homo sapiens Meis homeobox 2 (MEIS2), transcript variant a, mRNA.
NR_003041.1	SNORD13	2.414	Homo sapiens small nucleolar RNA, C/D box 13 (SNORD13), small nucleolar RNA.
NM_001852.3	COL9A2	2.413	Homo sapiens collagen, type IX, alpha 2 (COL9A2), mRNA.
NM_018649.2	H2AFY2	2.407	Homo sapiens H2A histone family, member Y2 (H2AFY2), mRNA.
NM_015383.1	NBPF14	2.406	Homo sapiens neuroblastoma breakpoint family, member 14 (NBPF14), mRNA.
NM_003887.2	ASAP2	2.405	Homo sapiens ArfGAP with SH3 domain, ankyrin repeat and PH domain 2 (ASAP2), transcript variant 1, mRNA.
NM_032961.1	PCDH10	2.404	Homo sapiens protocadherin 10 (PCDH10), transcript variant 1, mRNA.
XM_930677.1	LOC642299	2.404	PREDICTED: Homo sapiens hypothetical protein LOC642299 (LOC642299), mRNA.
NM_004107.3	FCGRT	2.403	Homo sapiens Fc fragment of IgG, receptor, transporter, alpha (FCGRT), transcript variant 1, mRNA.
NM_001135095.1	FNDC3B	2.403	Homo sapiens fibronectin type III domain containing 3B (FNDC3B), transcript variant 2, mRNA.
NM_032017.1	STK40	2.402	Homo sapiens serine/threonine kinase 40 (STK40), mRNA.
NM_000913.3	OPRL1	2.401	Homo sapiens opiate receptor-like 1 (OPRL1), transcript variant 2, mRNA.
NM_002676.1	PMM1	2.398	Homo sapiens phosphomannomutase 1 (PMM1), mRNA.
NM_153234.3	LIX1	2.395	Homo sapiens Lix1 homolog (mouse) (LIX1), mRNA.
NM_005953.2	MT2A	2.394	Homo sapiens metallothionein 2A (MT2A), mRNA.
NM_001759.2	CCND2	2.394	Homo sapiens cyclin D2 (CCND2), mRNA.
NM_004512.3	IL11RA	2.393	Homo sapiens interleukin 11 receptor, alpha (IL11RA), transcript variant 1, mRNA.
NM_198080.2	MSRB3	2.392	Homo sapiens methionine sulfoxide reductase B3 (MSRB3), transcript variant 1, mRNA.
NM_183372.3	LOC200030	2.390	Homo sapiens neuroblastoma breakpoint family, member 11-like (LOC200030), mRNA.
XM_374137.4	LOC389328	2.387	PREDICTED: Homo sapiens hypothetical LOC389328 (LOC389328), mRNA.
NM_018004.1	TMEM45A	2.386	Homo sapiens transmembrane protein 45A (TMEM45A), mRNA.
NM_000690.2	ALDH2	2.385	Homo sapiens aldehyde dehydrogenase 2 family (mitochondrial) (ALDH2), nuclear gene encoding mitochondrial protein, mRNA.
NM_016246.2	HSD17B14	2.384	Homo sapiens hydroxysteroid (17-beta) dehydrogenase 14 (HSD17B14), mRNA.
NM_024815.3	NUDT18	2.384	Homo sapiens nudix (nucleoside diphosphate linked moiety X)-type motif 18 (NUDT18), mRNA.
NM_181533.3	ABHD12B	2.383	Homo sapiens abhydrolase domain containing 12B (ABHD12B), transcript variant 2, mRNA.
NM_001406.3	EFNB3	2.381	Homo sapiens ephrin-B3 (EFNB3), mRNA.
NM_005419.2	STAT2	2.380	Homo sapiens signal transducer and activator of transcription 2, 113kDa (STAT2), mRNA.
NM_138371.1	FAM113B	2.378	Homo sapiens family with sequence similarity 113, member B (FAM113B), mRNA.
NM_013995.1	LAMP2	2.376	Homo sapiens lysosomal-associated membrane protein 2 (LAMP2), transcript variant LAMP2B, mRNA.
NM_003156.2	STIM1	2.373	Homo sapiens stromal interaction molecule 1 (STIM1), mRNA.
XM_001134449.1	KBTBD9	2.372	PREDICTED: Homo sapiens kelch repeat and BTB (POZ) domain containing 9 (KBTBD9), mRNA.
XM_001719242.1	LOC284297	2.369	PREDICTED: Homo sapiens hypothetical LOC284297 (LOC284297),

			mRNA.
NM_014570.3	ARFGAP3	2.369	Homo sapiens ADP-ribosylation factor GTPase activating protein 3 (ARFGAP3), mRNA.
NM_006343.2	MERTK	2.367	Homo sapiens c-mer proto-oncogene tyrosine kinase (MERTK), mRNA.
NM_177947.2	ARMCX3	2.365	Homo sapiens armadillo repeat containing, X-linked 3 (ARMCX3), transcript variant 2, mRNA.
NM_018286.2	TMEM100	2.363	Homo sapiens transmembrane protein 100 (TMEM100), transcript variant 2, mRNA.
NM_001002857.1	ANXA2	2.363	Homo sapiens annexin A2 (ANXA2), transcript variant 2, mRNA.
NM_002310.3	LIFR	2.361	Homo sapiens leukemia inhibitory factor receptor alpha (LIFR), mRNA.
NM_017594.3	DIRAS2	2.355	Homo sapiens DIRAS family, GTP-binding RAS-like 2 (DIRAS2), mRNA.
NM_031302.3	GLT8D2	2.354	Homo sapiens glycosyltransferase 8 domain containing 2 (GLT8D2), mRNA.
NM_004454.1	ETV5	2.353	Homo sapiens ets variant gene 5 (ets-related molecule) (ETV5), mRNA.
NM_024345.3	DCAF10	2.353	Homo sapiens DDB1 and CUL4 associated factor 10 (DCAF10), mRNA.
NM_030806.3	C1orf21	2.352	Homo sapiens chromosome 1 open reading frame 21 (C1orf21), mRNA.
NM_001018073.1	PCK2	2.352	Homo sapiens phosphoenolpyruvate carboxykinase 2 (mitochondrial) (PCK2), nuclear gene encoding mitochondrial protein, transcript variant 2, mRNA.
NM_015470.2	RAB11FIP5	2.352	Homo sapiens RAB11 family interacting protein 5 (class I) (RAB11FIP5), mRNA.
NM_198595.2	AFAP1	2.350	Homo sapiens actin filament associated protein 1 (AFAP1), transcript variant 2, mRNA.
NM_000628.3	IL10RB	2.350	Homo sapiens interleukin 10 receptor, beta (IL10RB), mRNA.
NM_004200.2	SYT7	2.350	Homo sapiens synaptotagmin VII (SYT7), mRNA.
NM_198845.2	SIGLEC6	2.350	Homo sapiens sialic acid binding Ig-like lectin 6 (SIGLEC6), transcript variant 2, mRNA.
NM_203417.1	RCAN1	2.348	Homo sapiens regulator of calcineurin 1 (RCAN1), transcript variant 2, mRNA.
NM_002409.4	MGAT3	2.348	Homo sapiens mannosyl (beta-1,4-)-glycoprotein beta-1,4-N-acetylglucosaminyltransferase (MGAT3), transcript variant 1, mRNA.
NM_007021.2	C10orf10	2.345	Homo sapiens chromosome 10 open reading frame 10 (C10orf10), mRNA.
NM_024569.3	MPZL1	2.345	Homo sapiens myelin protein zero-like 1 (MPZL1), transcript variant 2, mRNA.
NM_006123.2	IDS	2.344	Homo sapiens iduronate 2-sulfatase (Hunter syndrome) (IDS), transcript variant 2, mRNA.
NM_017515.3	SLC35F2	2.342	Homo sapiens solute carrier family 35, member F2 (SLC35F2), mRNA.
XM_941789.2	LOC647169	2.341	PREDICTED: Homo sapiens similar to glutathione S-transferase alpha 3 (LOC647169), mRNA.
NM_080818.3	OXGR1	2.341	Homo sapiens oxoglutarate (alpha-ketoglutarate) receptor 1 (OXGR1), mRNA.
NM_001014447.1	CPZ	2.340	Homo sapiens carboxypeptidase Z (CPZ), transcript variant 1, mRNA.
XM_928128.1	LOC643431	2.340	PREDICTED: Homo sapiens similar to Keratin, type II cytoskeletal 8 (Cytokeratin-8) (CK-8) (Keraton-8) (K8) (LOC643431), mRNA.
NM_001362.2	DIO3	2.338	Homo sapiens deiodinase, iodothyronine, type III (DIO3), mRNA.
NM_032256.1	TMEM117	2.337	Homo sapiens transmembrane protein 117 (TMEM117), mRNA.
NM_002222.4	ITPR1	2.336	Homo sapiens inositol 1,4,5-triphosphate receptor, type 1

			(ITPR1), transcript variant 2, mRNA.
NM_173546.1	KLHDC8B	2.336	Homo sapiens kelch domain containing 8B (KLHDC8B), mRNA.
NM_080821.2	C20orf108	2.334	Homo sapiens chromosome 20 open reading frame 108 (C20orf108), mRNA.
NM_001769.2	CD9	2.332	Homo sapiens CD9 molecule (CD9), mRNA.
NM_006022.2	TSC22D1	2.326	Homo sapiens TSC22 domain family, member 1 (TSC22D1), transcript variant 2, mRNA.
NM_005748.3	YAF2	2.326	Homo sapiens YY1 associated factor 2 (YAF2), mRNA.
AK127526		2.325	Homo sapiens cDNA FLJ45619 fis, clone BRTHA3027318
NM_002207.2	ITGA9	2.324	Homo sapiens integrin, alpha 9 (ITGA9), mRNA.
NM_002575.1	SERPINB2	2.323	Homo sapiens serpin peptidase inhibitor, clade B (ovalbumin), member 2 (SERPINB2), mRNA.
NM_001039703.1	NBPF10	2.323	## ## ## ## ##
NM_014734.2	KIAA0247	2.323	Homo sapiens KIAA0247 (KIAA0247), mRNA.
NM_207443.1	FLJ45244	2.322	Homo sapiens FLJ45244 protein (FLJ45244), mRNA.
NM_000346.2	SOX9	2.320	Homo sapiens SRY (sex determining region Y)-box 9 (campomelic dysplasia, autosomal sex-reversal) (SOX9), mRNA.
NM_001375.2	DNASE2	2.319	Homo sapiens deoxyribonuclease II, lysosomal (DNASE2), mRNA.
NM_001040455.1	SIDT2	2.313	Homo sapiens SID1 transmembrane family, member 2 (SIDT2), mRNA.
NM_003877.3	SOCS2	2.311	Homo sapiens suppressor of cytokine signaling 2 (SOCS2), mRNA.
NM_019858.1	GPR162	2.310	Homo sapiens G protein-coupled receptor 162 (GPR162), transcript variant A-2, mRNA.
NM_017515.3	SLC35F2	2.309	Homo sapiens solute carrier family 35, member F2 (SLC35F2), mRNA.
NM_001825.2	CKMT2	2.309	Homo sapiens creatine kinase, mitochondrial 2 (sarcomeric) (CKMT2), nuclear gene encoding mitochondrial protein, transcript variant 1, mRNA.
NM_021913.2	AXL	2.309	Homo sapiens AXL receptor tyrosine kinase (AXL), transcript variant 1, mRNA.
NM_020899.2	ZBTB4	2.309	Homo sapiens zinc finger and BTB domain containing 4 (ZBTB4), mRNA.
NM_006084.4	IRF9	2.308	Homo sapiens interferon regulatory factor 9 (IRF9), mRNA.
NM_001248.1	ENTPD3	2.304	Homo sapiens ectonucleoside triphosphate diphosphohydrolase 3 (ENTPD3), mRNA.
NM_021623.1	PLEKHA2	2.302	Homo sapiens pleckstrin homology domain containing, family A (phosphoinositide binding specific) member 2 (PLEKHA2), mRNA.
NM_030929.3	KAZALD1	2.301	Homo sapiens Kazal-type serine peptidase inhibitor domain 1 (KAZALD1), mRNA.
NM_033254.2	BOC	2.297	Homo sapiens Boc homolog (mouse) (BOC), mRNA.
NM_172128.1	CAMK2D	2.296	Homo sapiens calcium/calmodulin-dependent protein kinase (CaM kinase) II delta (CAMK2D), transcript variant 2, mRNA.
NM_017514.2	PLXNA3	2.293	Homo sapiens plexin A3 (PLXNA3), mRNA.
NM_004496.2	FOXA1	2.292	Homo sapiens forkhead box A1 (FOXA1), mRNA.
NM_001005376.1	PLAUR	2.291	Homo sapiens plasminogen activator, urokinase receptor (PLAUR), transcript variant 2, mRNA.
NM_014452.3	TNFRSF21	2.290	Homo sapiens tumor necrosis factor receptor superfamily, member 21 (TNFRSF21), mRNA.
NM_004235.3	KLF4	2.289	Homo sapiens Kruppel-like factor 4 (gut) (KLF4), mRNA.
NM_001033873.1	SMAGP	2.288	Homo sapiens small cell adhesion glycoprotein (SMAGP), transcript variant 2, mRNA.
NM_000381.1	MID1	2.287	Homo sapiens midline 1 (Opitz/BBB syndrome) (MID1), transcript variant 1, mRNA.

NM_001080467.1	MYO5B	2.287	Homo sapiens myosin VB (MYO5B), mRNA.
NM_003070.3	SMARCA2	2.286	Homo sapiens SWI/SNF related, matrix associated, actin dependent regulator of chromatin, subfamily a, member 2 (SMARCA2), transcript variant 1, mRNA.
NM_003764.2	STX11	2.282	Homo sapiens syntaxin 11 (STX11), mRNA.
NM_014556.2	EVC	2.282	Homo sapiens Ellis van Creveld syndrome (EVC), transcript variant 1, mRNA.
NM_002979.3	SCP2	2.278	Homo sapiens sterol carrier protein 2 (SCP2), transcript variant 1, mRNA.
NM_001017922.1	ERMAP	2.276	Homo sapiens erythroblast membrane-associated protein (Scianna blood group) (ERMAP), transcript variant 1, mRNA.
NM_173354.3	SIK1	2.276	Homo sapiens salt-inducible kinase 1 (SIK1), mRNA.
NM_032211.6	LOXL4	2.274	Homo sapiens lysyl oxidase-like 4 (LOXL4), mRNA.
NM_005574.2	LMO2	2.273	Homo sapiens LIM domain only 2 (rhombotin-like 1) (LMO2), mRNA.
NM_153214.1	FBLN7	2.271	Homo sapiens fibulin 7 (FBLN7), mRNA.
NM_001003802.1	SMARCD3	2.271	Homo sapiens SWI/SNF related, matrix associated, actin dependent regulator of chromatin, subfamily d, member 3 (SMARCD3), transcript variant 1, mRNA.
NM_001792.2	CDH2	2.269	Homo sapiens cadherin 2, type 1, N-cadherin (neuronal) (CDH2), mRNA.
NM_005139.2	ANXA3	2.269	Homo sapiens annexin A3 (ANXA3), mRNA.
NM_003887.1	DDEF2	2.268	Homo sapiens development and differentiation enhancing factor 2 (DDEF2), mRNA.
NM_012453.2	TBL2	2.267	Homo sapiens transducin (beta)-like 2 (TBL2), mRNA.
NM_177947.2	ARMCX3	2.265	Homo sapiens armadillo repeat containing, X-linked 3 (ARMCX3), transcript variant 2, mRNA.
NM_002293.2	LAMC1	2.265	Homo sapiens laminin, gamma 1 (formerly LAMB2) (LAMC1), mRNA.
NM_002292.3	LAMB2	2.264	Homo sapiens laminin, beta 2 (laminin S) (LAMB2), mRNA.
NM_030915.1	LBH	2.263	Homo sapiens limb bud and heart development homolog (mouse) (LBH), mRNA.
NM_001006667.1	TAC3	2.262	Homo sapiens tachykinin 3 (neuromedin K, neurokinin beta) (TAC3), transcript variant 1, mRNA.
NM_024422.2	DSC2	2.262	Homo sapiens desmocollin 2 (DSC2), transcript variant Dsc2a, mRNA.
NM_198552.1	FAM89A	2.262	Homo sapiens family with sequence similarity 89, member A (FAM89A), mRNA.
NM_001677.3	ATP1B1	2.261	Homo sapiens ATPase, Na+/K+ transporting, beta 1 polypeptide (ATP1B1), transcript variant 1, mRNA.
NM_001024629.1	NRP1	2.258	Homo sapiens neuropilin 1 (NRP1), transcript variant 3, mRNA.
NM_033290.2	MID1	2.256	Homo sapiens midline 1 (Opitz/BBB syndrome) (MID1), transcript variant 3, mRNA.
NM_018043.4	TMEM16A	2.256	Homo sapiens transmembrane protein 16A (TMEM16A), mRNA.
NM_016240.2	SCARA3	2.254	Homo sapiens scavenger receptor class A, member 3 (SCARA3), transcript variant 1, mRNA.
NM_032354.2	TMEM107	2.254	Homo sapiens transmembrane protein 107 (TMEM107), transcript variant 1, mRNA.
NM_006986.3	MAGED1	2.252	Homo sapiens melanoma antigen family D, 1 (MAGED1), transcript variant 2, mRNA.
NM_032323.1	TMEM79	2.251	Homo sapiens transmembrane protein 79 (TMEM79), mRNA.
NM_003887.2	ASAP2	2.249	Homo sapiens ArfGAP with SH3 domain, ankyrin repeat and PH domain 2 (ASAP2), transcript variant 1, mRNA.
NM_005723.2	TSPAN5	2.246	Homo sapiens tetraspanin 5 (TSPAN5), mRNA.
NM_002928.2	RGS16	2.244	Homo sapiens regulator of G-protein signalling 16 (RGS16), mRNA.
NM_005859.3	PURA	2.242	Homo sapiens purine-rich element binding protein A (PURA),

			mRNA.
NM_031946.3	CENTG3	2.241	Homo sapiens centaurin, gamma 3 (CENTG3), mRNA.
NM_003761.2	VAMP8	2.239	Homo sapiens vesicle-associated membrane protein 8 (endobrevin) (VAMP8), mRNA.
NM_007029.2	STMN2	2.238	Homo sapiens stathmin-like 2 (STMN2), mRNA.
NM_138373.3	MYADM	2.236	Homo sapiens myeloid-associated differentiation marker (MYADM), transcript variant 2, mRNA.
NM_015338.4	ASXL1	2.230	Homo sapiens additional sex combs like 1 (Drosophila) (ASXL1), mRNA.
NM_005514.5	HLA-B	2.222	Homo sapiens major histocompatibility complex, class I, B (HLA-B), mRNA.
NM_005949.2	MT1F	2.216	Homo sapiens metallothionein 1F (MT1F), mRNA.
NM_006134.5	TMEM50B	2.215	Homo sapiens transmembrane protein 50B (TMEM50B), mRNA.
XM_372780.3	LOC391045	2.211	PREDICTED: Homo sapiens similar to Solute carrier family 2, facilitated glucose transporter member 3 (Glucose transporter type 3, brain) (LOC391045), mRNA.
NM_001014431.1	AKT1	2.209	Homo sapiens v-akt murine thymoma viral oncogene homolog 1 (AKT1), transcript variant 3, mRNA.
NM_003998.2	NFKB1	2.208	Homo sapiens nuclear factor of kappa light polypeptide gene enhancer in B-cells 1 (NFKB1), mRNA.
NM_020815.1	PCDH10	2.206	Homo sapiens protocadherin 10 (PCDH10), transcript variant 2, mRNA.
NR_031742.1	MIR1978	2.204	Homo sapiens microRNA 1978 (MIR1978), microRNA.
XR_036921.1	LOC399965	2.204	PREDICTED: Homo sapiens misc_RNA (LOC399965), miscRNA.
XM_929667.1	LOC653778	2.202	PREDICTED: Homo sapiens similar to solute carrier family 25, member 37 (LOC653778), mRNA.
NM_007364.2	TMED3	2.198	Homo sapiens transmembrane emp24 protein transport domain containing 3 (TMED3), mRNA.
NR_002954.1	SNORA12	2.195	Homo sapiens small nucleolar RNA, H/ACA box 12 (SNORA12), small nucleolar RNA.
NM_134445.2	CD99L2	2.193	Homo sapiens CD99 molecule-like 2 (CD99L2), transcript variant 3, mRNA.
NM_001005742.1	GBA	2.190	Homo sapiens glucosidase, beta; acid (includes glucosylceramidase) (GBA), transcript variant 3, mRNA.
NM_198148.1	CPXM2	2.185	Homo sapiens carboxypeptidase X (M14 family), member 2 (CPXM2), mRNA.
NM_020443.2	NAV1	2.180	Homo sapiens neuron navigator 1 (NAV1), mRNA.
NM_014262.2	LEPREL2	2.177	Homo sapiens leprecan-like 2 (LEPREL2), mRNA.
NM_016941.2	DLL3	2.176	Homo sapiens delta-like 3 (Drosophila) (DLL3), transcript variant 1, mRNA.
NM_133436.1	ASNS	2.176	Homo sapiens asparagine synthetase (ASNS), transcript variant 1, mRNA.
NM_001200.2	BMP2	2.175	Homo sapiens bone morphogenetic protein 2 (BMP2), mRNA.
NM_024843.2	CYBRD1	2.173	Homo sapiens cytochrome b reductase 1 (CYBRD1), mRNA.
NM_001006944.1	RPS6KA4	2.168	Homo sapiens ribosomal protein S6 kinase, 90kDa, polypeptide 4 (RPS6KA4), transcript variant 2, mRNA.
NM_003364.2	UPP1	2.165	Homo sapiens uridine phosphorylase 1 (UPP1), transcript variant 1, mRNA.
NM_002227.2	JAK1	2.162	Homo sapiens Janus kinase 1 (JAK1), mRNA.
NM_000153.2	GALC	2.159	Homo sapiens galactosylceramidase (GALC), transcript variant 1, mRNA.
NM_003254.2	TIMP1	2.157	Homo sapiens TIMP metallopeptidase inhibitor 1 (TIMP1), mRNA.
NM_152493.2	ZNF362	2.155	Homo sapiens zinc finger protein 362 (ZNF362), mRNA.
NM_001040092.1	ENPP2	2.148	Homo sapiens ectonucleotide pyrophosphatase/phosphodiesterase 2 (ENPP2), transcript variant 2, mRNA.
NM_018660.2	ZNF395	2.142	Homo sapiens zinc finger protein 395 (ZNF395), mRNA.

NM_001024211.1	S100A13	2.139	Homo sapiens S100 calcium binding protein A13 (S100A13), transcript variant 3, mRNA.
NM_003641.3	IFITM1	2.139	Homo sapiens interferon induced transmembrane protein 1 (9-27) (IFITM1), mRNA.
NM_205767.1	C19orf70	2.136	Homo sapiens chromosome 19 open reading frame 70 (C19orf70), mRNA.
NM_182972.2	IRF2BP2	2.134	Homo sapiens interferon regulatory factor 2 binding protein 2 (IRF2BP2), transcript variant 1, mRNA.
BG698090		2.131	602659965F1 NCI_CGAP_Skn3 Homo sapiens cDNA clone IMAGE:4802969 5, mRNA sequence
NM_000022.2	ADA	2.126	Homo sapiens adenosine deaminase (ADA), mRNA.
NM_006810.2	PDIA5	2.126	Homo sapiens protein disulfide isomerase family A, member 5 (PDIA5), mRNA.
NM_003842.3	TNFRSF10B	2.125	Homo sapiens tumor necrosis factor receptor superfamily, member 10b (TNFRSF10B), transcript variant 1, mRNA.
NM_005952.2	MT1X	2.119	Homo sapiens metallothionein 1X (MT1X), mRNA.
NM_031219.2	HDHD3	2.119	Homo sapiens haloacid dehalogenase-like hydrolase domain containing 3 (HDHD3), mRNA.
NM_139343.1	BIN1	2.118	Homo sapiens bridging integrator 1 (BIN1), transcript variant 1, mRNA.
NM_138370.2	PKDCC	2.114	Homo sapiens protein kinase domain containing, cytoplasmic homolog (mouse) (PKDCC), mRNA.
NM_144779.1	FXYD5	2.114	Homo sapiens FXYD domain containing ion transport regulator 5 (FXYD5), transcript variant 1, mRNA.
NM_021913.2	AXL	2.112	Homo sapiens AXL receptor tyrosine kinase (AXL), transcript variant 1, mRNA.
NM_016021.2	UBE2J1	2.111	Homo sapiens ubiquitin-conjugating enzyme E2, J1 (UBC6 homolog, yeast) (UBE2J1), mRNA.
AK091747		2.111	Homo sapiens cDNA FLJ34428 fis, clone HLUNG2000761
NM_001020820.1	MYADM	2.107	Homo sapiens myeloid-associated differentiation marker (MYADM), transcript variant 4, mRNA.
NM_201222.1	MAGED2	2.107	Homo sapiens melanoma antigen family D, 2 (MAGED2), transcript variant 3, mRNA.
NM_014164.4	FXYD5	2.106	Homo sapiens FXYD domain containing ion transport regulator 5 (FXYD5), transcript variant 2, mRNA.
NM_001024668.1	LETMD1	2.100	Homo sapiens LETM1 domain containing 1 (LETMD1), transcript variant 2, mRNA.
NM_201630.1	LRRN2	2.100	Homo sapiens leucine rich repeat neuronal 2 (LRRN2), transcript variant 2, mRNA.
NM_012106.3	ARL2BP	2.097	Homo sapiens ADP-ribosylation factor-like 2 binding protein (ARL2BP), mRNA.
NM_002294.1	LAMP2	2.096	Homo sapiens lysosomal-associated membrane protein 2 (LAMP2), transcript variant LAMP2A, mRNA.
NM_032928.2	TMEM141	2.091	Homo sapiens transmembrane protein 141 (TMEM141), mRNA.
NM_000156.4	GAMT	2.089	Homo sapiens guanidinoacetate N-methyltransferase (GAMT), transcript variant 1, mRNA.
NM_001037537.1	PHYH	2.086	Homo sapiens phytanoyl-CoA 2-hydroxylase (PHYH), transcript variant 2, mRNA.
NM_201414.1	APP	2.085	Homo sapiens amyloid beta (A4) precursor protein (APP), transcript variant 3, mRNA.
NM_005516.4	HLA-E	2.085	Homo sapiens major histocompatibility complex, class I, E (HLA-E), mRNA.
NM_001023567.2	GOLGA8B	2.085	Homo sapiens golgi autoantigen, golgin subfamily a, 8B (GOLGA8B), mRNA.
NM_001014432.1	AKT1	2.075	Homo sapiens v-akt murine thymoma viral oncogene homolog 1 (AKT1), transcript variant 2, mRNA.
NM_001040437.1	C6orf48	2.056	Homo sapiens chromosome 6 open reading frame 48 (C6orf48),

			transcript variant 1, mRNA.
XM_939368.1	LOC654103	2.054	PREDICTED: Homo sapiens similar to solute carrier family 25, member 37 (LOC654103), mRNA.
NM_005779.1	LHFPL2	2.052	Homo sapiens lipoma HMGIC fusion partner-like 2 (LHFPL2), mRNA.
NM_201414.1	APP	2.050	Homo sapiens amyloid beta (A4) precursor protein (APP), transcript variant 3, mRNA.
NM_033668.1	ITGB1	2.049	Homo sapiens integrin, beta 1 (fibronectin receptor, beta polypeptide, antigen CD29 includes MDF2, MSK12) (ITGB1), transcript variant 1D, mRNA.
NM_153682.2	PIGP	2.040	Homo sapiens phosphatidylinositol glycan anchor biosynthesis, class P (PIGP), transcript variant 2, mRNA.
NM_000202.3	IDS	2.037	Homo sapiens iduronate 2-sulfatase (Hunter syndrome) (IDS), transcript variant 1, mRNA.
NM_181509.1	MAP1LC3A	2.022	Homo sapiens microtubule-associated protein 1 light chain 3 alpha (MAP1LC3A), transcript variant 2, mRNA.
NM_001614.2	ACTG1	2.018	Homo sapiens actin, gamma 1 (ACTG1), mRNA.
NM_001144.4	AMFR	2.011	Homo sapiens autocrine motility factor receptor (AMFR), mRNA.
NM_032102.2	SFRS2B	2.011	Homo sapiens splicing factor, arginine/serine-rich 2B (SFRS2B), mRNA.
NM_006675.3	TSPAN9	2.007	Homo sapiens tetraspanin 9 (TSPAN9), mRNA.
NM_002775.3	HTRA1	6.923	Homo sapiens HtrA serine peptidase 1 (HTRA1), mRNA.
NM_017949.1	CUEDC1	2.492	Homo sapiens CUE domain containing 1 (CUEDC1), mRNA.
NM_019064.3	SDK2	2.284	Homo sapiens sidekick homolog 2 (chicken) (SDK2), mRNA.
NM_004083.4	DDIT3	6.132	Homo sapiens DNA-damage-inducible transcript 3 (DDIT3), mRNA.
NM_004415.2	DSP	2.892	Homo sapiens desmoplakin (DSP), transcript variant 1, mRNA.
NM_024913.3	FLJ21986	13.684	Homo sapiens hypothetical protein FLJ21986 (FLJ21986), mRNA.
NM_000963.1	PTGS2	3.643	Homo sapiens prostaglandin-endoperoxide synthase 2 (prostaglandin G/H synthase and cyclooxygenase) (PTGS2), mRNA.
NM_001031677.2	RAB24	2.151	Homo sapiens RAB24, member RAS oncogene family (RAB24), transcript variant 1, mRNA.
NM_005141.2	FGB	12.141	Homo sapiens fibrinogen beta chain (FGB), mRNA.
NM_001175.4	ARHGDIB	18.076	Homo sapiens Rho GDP dissociation inhibitor (GDI) beta (ARHGDIB), mRNA.
NM_000902.3	MME	5.293	Homo sapiens membrane metallo-endopeptidase (MME), transcript variant 1, mRNA.
NM_001037633.1	SIL1	3.181	Homo sapiens SIL1 homolog, endoplasmic reticulum chaperone (S. cerevisiae) (SIL1), transcript variant 1, mRNA.
NM_001884.2	HAPLN1	18.816	Homo sapiens hyaluronan and proteoglycan link protein 1 (HAPLN1), mRNA.
NM_022469.3	GREM2	2.727	Homo sapiens gremlin 2, cysteine knot superfamily, homolog (Xenopus laevis) (GREM2), mRNA.
NM_001013251.1	SLC3A2	2.236	Homo sapiens solute carrier family 3 (activators of dibasic and neutral amino acid transport), member 2 (SLC3A2), transcript variant 6, mRNA.
NM_016269.2	LEF1	7.560	Homo sapiens lymphoid enhancer-binding factor 1 (LEF1), mRNA.
XM_001128725.1	UGT2B7	4.120	PREDICTED: Homo sapiens UDP glucuronosyltransferase 2 family, polypeptide B7 (UGT2B7), mRNA.
NM_147189.2	FAM110B	2.972	Homo sapiens family with sequence similarity 110, member B (FAM110B), mRNA.
NM_198951.1	TGM2	5.299	Homo sapiens transglutaminase 2 (C polypeptide, protein-glutamine-gamma-glutamyltransferase) (TGM2), transcript variant 2, mRNA.
NM_000679.3	ADRA1B	5.164	Homo sapiens adrenergic, alpha-1B-, receptor (ADRA1B), mRNA.

XM_001128419.1	MGC16121	9.205	PREDICTED: Homo sapiens hypothetical protein MGC16121 (MGC16121), mRNA.
NM_006988.3	ADAMTS1	3.913	Homo sapiens ADAM metallopeptidase with thrombospondin type 1 motif, 1 (ADAMTS1), mRNA.
NM_002237.3	KCNG1	2.652	Homo sapiens potassium voltage-gated channel, subfamily G, member 1 (KCNG1), mRNA.
NM_138609.2	H2AFY	7.076	Homo sapiens H2A histone family, member Y (H2AFY), transcript variant 1, mRNA.
NM_139346.1	BIN1	2.430	Homo sapiens bridging integrator 1 (BIN1), transcript variant 4, mRNA.
NM_002653.3	PITX1	57.922	Homo sapiens paired-like homeodomain transcription factor 1 (PITX1), mRNA.
NM_032823.3	C9orf3	2.445	Homo sapiens chromosome 9 open reading frame 3 (C9orf3), mRNA.
NM_001844.3	COL2A1	2.546	Homo sapiens collagen, type II, alpha 1 (primary osteoarthritis, spondyloepiphyseal dysplasia, congenital) (COL2A1), transcript variant 1, mRNA.
NM_002147.3	HOXB5	12.260	Homo sapiens homeobox B5 (HOXB5), mRNA.
NM_001025356.1	ANO6	2.753	Homo sapiens anoctamin 6 (ANO6), mRNA.
NM_018022.1	TMEM51	3.316	Homo sapiens transmembrane protein 51 (TMEM51), mRNA.
NM_016574.2	DRD2	2.372	Homo sapiens dopamine receptor D2 (DRD2), transcript variant 2, mRNA.
NM_001977.3	ENPEP	3.302	Homo sapiens glutamyl aminopeptidase (aminopeptidase A) (ENPEP), mRNA.
NM_003621.1	PPFIBP2	2.602	Homo sapiens PTPRF interacting protein, binding protein 2 (liprin beta 2) (PPFIBP2), mRNA.
NM_001847.2	COL4A6	5.798	Homo sapiens collagen, type IV, alpha 6 (COL4A6), transcript variant A, mRNA.
NM_001003793.1	RBMS3	2.458	Homo sapiens RNA binding motif, single stranded interacting protein (RBMS3), transcript variant 1, mRNA.
XM_001131480.1	ALOX15	3.549	PREDICTED: Homo sapiens arachidonate 15-lipoxygenase (ALOX15), mRNA.
NM_198080.2	MSRB3	4.699	Homo sapiens methionine sulfoxide reductase B3 (MSRB3), transcript variant 1, mRNA.
NM_032832.4	LRP11	3.719	Homo sapiens low density lipoprotein receptor-related protein 11 (LRP11), mRNA.
NM_014467.2	SRPX2	5.944	Homo sapiens sushi-repeat-containing protein, X-linked 2 (SRPX2), mRNA.
NM_015347.3	RIMBP2	3.960	Homo sapiens RIMS binding protein 2 (RIMBP2), mRNA.
NM_000615.5	NCAM1	2.322	Homo sapiens neural cell adhesion molecule 1 (NCAM1), transcript variant 1, mRNA.
NM_022760.3	FAM113A	2.994	Homo sapiens family with sequence similarity 113, member A (FAM113A), mRNA.
NM_014045.3	LRP10	2.289	Homo sapiens low density lipoprotein receptor-related protein 10 (LRP10), mRNA.
NM_018242.2	SLC47A1	4.173	Homo sapiens solute carrier family 47, member 1 (SLC47A1), mRNA.
NM_174911.3	FAM84B	3.028	Homo sapiens family with sequence similarity 84, member B (FAM84B), mRNA.
NM_001999.3	FBN2	5.458	Homo sapiens fibrillin 2 (FBN2), mRNA.
NM_001616.3	ACVR2A	2.211	Homo sapiens activin A receptor, type IIA (ACVR2A), mRNA.
NM_144607.3	CYB5D1	3.570	Homo sapiens cytochrome b5 domain containing 1 (CYB5D1), mRNA.
NM_001265.2	CDX2	7.423	Homo sapiens caudal type homeobox 2 (CDX2), mRNA.
NM_000965.2	RARB	4.076	Homo sapiens retinoic acid receptor, beta (RARB), transcript variant 1, mRNA.
NM_001002292.1	GPR177	14.683	Homo sapiens G protein-coupled receptor 177 (GPR177),

			transcript variant 2, mRNA.
NM_003174.3	SVIL	3.889	Homo sapiens supervillin (SVIL), transcript variant 1, mRNA.
NM_003730.3	RNASET2	2.179	Homo sapiens ribonuclease T2 (RNASET2), mRNA.
NM_018281.2	ECHDC2	2.581	Homo sapiens enoyl Coenzyme A hydratase domain containing 2 (ECHDC2), mRNA.
NM_001613.1	ACTA2	26.846	Homo sapiens actin, alpha 2, smooth muscle, aorta (ACTA2), mRNA.
NM_004563.2	PCK2	3.057	Homo sapiens phosphoenolpyruvate carboxykinase 2 (mitochondrial) (PCK2), nuclear gene encoding mitochondrial protein, transcript variant 1, mRNA.
NM_000313.1	PROS1	2.166	Homo sapiens protein S (alpha) (PROS1), mRNA.
XR_016703.1	LOC644743	4.519	PREDICTED: Homo sapiens hypothetical LOC644743 (LOC644743), mRNA.
NM_016613.4	C4orf18	10.483	Homo sapiens chromosome 4 open reading frame 18 (C4orf18), transcript variant 2, mRNA.
NM_006486.2	FBLN1	4.390	Homo sapiens fibulin 1 (FBLN1), transcript variant D, mRNA.
NM_001797.2	CDH11	12.410	Homo sapiens cadherin 11, type 2, OB-cadherin (osteoblast) (CDH11), mRNA.
NM_003619.2	PRSS12	4.532	Homo sapiens protease, serine, 12 (neurotrypsin, motopsin) (PRSS12), mRNA.
NM_001092.3	ABR	2.250	Homo sapiens active BCR-related gene (ABR), transcript variant 2, mRNA.
NM_000474.3	TWIST1	13.168	Homo sapiens twist homolog 1 (Drosophila) (TWIST1), mRNA.
NM_017656.2	ZNF562	2.865	Homo sapiens zinc finger protein 562 (ZNF562), mRNA.
NM_001042678.1	RHOC	2.860	Homo sapiens ras homolog gene family, member C (RHOC), transcript variant 2, mRNA.
NM_006435.2	IFITM2	2.437	Homo sapiens interferon induced transmembrane protein 2 (1-8D) (IFITM2), mRNA.
NM_000422.1	KRT17	5.101	Homo sapiens keratin 17 (KRT17), mRNA.
NM_001759.2	CCND2	2.697	Homo sapiens cyclin D2 (CCND2), mRNA.
NM_002194.2	INPP1	2.335	Homo sapiens inositol polyphosphate-1-phosphatase (INPP1), mRNA.
NM_018837.2	SULF2	2.327	Homo sapiens sulfatase 2 (SULF2), transcript variant 1, mRNA.
NM_002342.1	LTBR	2.420	Homo sapiens lymphotoxin beta receptor (TNFR superfamily, member 3) (LTBR), mRNA.
NM_022748.10	TNS3	2.437	Homo sapiens tensin 3 (TNS3), mRNA.
NM_145263.2	SPATA18	3.530	Homo sapiens spermatogenesis associated 18 homolog (rat) (SPATA18), mRNA.
NM_052966.1	C1orf24	4.139	Homo sapiens chromosome 1 open reading frame 24 (C1orf24), transcript variant 2, mRNA.
NM_000756.1	CRH	5.218	Homo sapiens corticotropin releasing hormone (CRH), mRNA.
NM_016815.2	GYPC	4.393	Homo sapiens glycophorin C (Gerbich blood group) (GYPC), transcript variant 2, mRNA.
NM_178507.2	OAF	12.847	Homo sapiens OAF homolog (Drosophila) (OAF), mRNA.
NM_003387.3	WASPIP	5.470	Homo sapiens Wiskott-Aldrich syndrome protein interacting protein (WASPIP), mRNA.
NM_014876.3	JOSD1	2.075	Homo sapiens Josephin domain containing 1 (JOSD1), mRNA.
NM_153449.2	SLC2A14	2.242	Homo sapiens solute carrier family 2 (facilitated glucose transporter), member 14 (SLC2A14), mRNA.
NM_031944.1	MIXL1	2.642	Homo sapiens Mix1 homeobox-like 1 (Xenopus laevis) (MIXL1), mRNA.
NM_000943.4	PPIC	5.807	Homo sapiens peptidylprolyl isomerase C (cyclophilin C) (PPIC), mRNA.
NM_033260.3	FOXQ1	2.231	Homo sapiens forkhead box Q1 (FOXQ1), mRNA.
NM_138992.1	BACE2	2.258	Homo sapiens beta-site APP-cleaving enzyme 2 (BACE2), transcript variant b, mRNA.
NM_015037.2	KIAA0913	2.017	Homo sapiens KIAA0913 (KIAA0913), mRNA.

NM_004403.2	DFNA5	2.612	Homo sapiens deafness, autosomal dominant 5 (DFNA5), transcript variant 1, mRNA.
NM_002999.2	SDC4	2.639	Homo sapiens syndecan 4 (SDC4), mRNA.
NM_006079.3	CITED2	2.052	Homo sapiens Cbp/p300-interacting transactivator, with Glu/Asp-rich carboxy-terminal domain, 2 (CITED2), transcript variant 1, mRNA.
NM_002381.4	MATN3	2.257	Homo sapiens matrilin 3 (MATN3), mRNA.
NR_003578.1	ZNF702P	2.395	Homo sapiens zinc finger protein 702 (pseudogene) (ZNF702P), non-coding RNA.
NM_000240.2	MAOA	2.205	Homo sapiens monoamine oxidase A (MAOA), nuclear gene encoding mitochondrial protein, mRNA.
NM_001030050.1	KLK3	3.138	Homo sapiens kallikrein-related peptidase 3 (KLK3), transcript variant 6, mRNA.
NM_000358.1	TGFBI	36.094	Homo sapiens transforming growth factor, beta-induced, 68kDa (TGFBI), mRNA.
NM_172373.2	ELF1	3.100	Homo sapiens E74-like factor 1 (ets domain transcription factor) (ELF1), mRNA.
NM_000384.2	APOB	2.699	Homo sapiens apolipoprotein B (including Ag(x) antigen) (APOB), mRNA.
NM_000364.2	TNNT2	8.914	Homo sapiens troponin T type 2 (cardiac) (TNNT2), transcript variant 1, mRNA.
NM_006329.2	FBLN5	3.332	Homo sapiens fibulin 5 (FBLN5), mRNA.
NM_022356.2	LEPRE1	3.054	Homo sapiens leucine proline-enriched proteoglycan (leprecan) 1 (LEPRE1), mRNA.
NM_004512.3	IL11RA	3.729	Homo sapiens interleukin 11 receptor, alpha (IL11RA), transcript variant 1, mRNA.
NM_001457.1	FLNB	2.576	Homo sapiens filamin B, beta (actin binding protein 278) (FLNB), mRNA.
NM_006371.3	CRTAP	2.889	Homo sapiens cartilage associated protein (CRTAP), mRNA.
NM_018948.2	ERRFI1	2.940	Homo sapiens ERBB receptor feedback inhibitor 1 (ERRFI1), mRNA.
NM_002203.3	ITGA2	2.725	Homo sapiens integrin, alpha 2 (CD49B, alpha 2 subunit of VLA-2 receptor) (ITGA2), mRNA.
NM_080491.1	GAB2	2.550	Homo sapiens GRB2-associated binding protein 2 (GAB2), transcript variant 1, mRNA.
NM_015689.2	DENND2A	3.121	Homo sapiens DENN/MADD domain containing 2A (DENND2A), mRNA.
NM_173465.2	COL23A1	2.486	Homo sapiens collagen, type XXIII, alpha 1 (COL23A1), mRNA.
NM_001001794.2	FAM116B	2.244	Homo sapiens family with sequence similarity 116, member B (FAM116B), mRNA.
NM_012396.3	PHLDA3	3.569	Homo sapiens pleckstrin homology-like domain, family A, member 3 (PHLDA3), mRNA.
NM_003506.2	FZD6	2.201	Homo sapiens frizzled homolog 6 (Drosophila) (FZD6), mRNA.
NM_152753.2	SCUBE3	6.178	Homo sapiens signal peptide, CUB domain, EGF-like 3 (SCUBE3), mRNA.
NM_000231.1	SGCG	2.811	Homo sapiens sarcoglycan, gamma (35kDa dystrophin-associated glycoprotein) (SGCG), mRNA.
NM_014061.3	MAGEH1	2.419	Homo sapiens melanoma antigen family H, 1 (MAGEH1), mRNA.
NM_000807.1	GABRA2	2.556	Homo sapiens gamma-aminobutyric acid (GABA) A receptor, alpha 2 (GABRA2), mRNA.
NM_004281.3	BAG3	2.743	Homo sapiens BCL2-associated athanogene 3 (BAG3), mRNA.
NM_014330.2	PPP1R15A	2.212	Homo sapiens protein phosphatase 1, regulatory (inhibitor) subunit 15A (PPP1R15A), mRNA.
NM_176796.1	P2RY6	5.767	Homo sapiens pyrimidinergic receptor P2Y, G-protein coupled, 6 (P2RY6), transcript variant 3, mRNA.
NM_003638.1	ITGA8	3.508	Homo sapiens integrin, alpha 8 (ITGA8), mRNA.
NM_183376.1	ARRDC4	2.885	Homo sapiens arrestin domain containing 4 (ARRDC4), mRNA.

NM_001002292.1	GPR177	9.127	Homo sapiens G protein-coupled receptor 177 (GPR177), transcript variant 2, mRNA.
NM_021073.2	BMP5	6.799	Homo sapiens bone morphogenetic protein 5 (BMP5), mRNA.
NM_006868.2	RAB31	2.618	Homo sapiens RAB31, member RAS oncogene family (RAB31), mRNA.
NM_152550.2	SH3RF2	3.598	Homo sapiens SH3 domain containing ring finger 2 (SH3RF2), mRNA.
NM_019058.2	DDIT4	9.474	Homo sapiens DNA-damage-inducible transcript 4 (DDIT4), mRNA.
XM_926584.1	LOC653110	6.731	PREDICTED: Homo sapiens similar to annexin A8, transcript variant 1 (LOC653110), mRNA.
NM_152882.2	PTK7	2.182	Homo sapiens PTK7 protein tyrosine kinase 7 (PTK7), transcript variant PTK7-4, mRNA.
NM_006287.4	TFPI	7.195	Homo sapiens tissue factor pathway inhibitor (lipoprotein-associated coagulation inhibitor) (TFPI), transcript variant 1, mRNA.
NM_002922.3	RGS1	3.715	Homo sapiens regulator of G-protein signaling 1 (RGS1), mRNA.
NM_002658.2	PLAU	4.669	Homo sapiens plasminogen activator, urokinase (PLAU), mRNA.
NM_020130.3	C8orf4	17.318	Homo sapiens chromosome 8 open reading frame 4 (C8orf4), mRNA.
NM_023915.2	GPR87	2.257	Homo sapiens G protein-coupled receptor 87 (GPR87), mRNA.
NM_021871.2	FGA	4.206	Homo sapiens fibrinogen alpha chain (FGA), transcript variant alpha, mRNA.
NM_002899.2	RBP1	2.243	Homo sapiens retinol binding protein 1, cellular (RBP1), mRNA.
NM_003038.2	SLC1A4	2.375	Homo sapiens solute carrier family 1 (glutamate/neutral amino acid transporter), member 4 (SLC1A4), mRNA.
NM_003966.1	SEMA5A	3.884	Homo sapiens sema domain, seven thrombospondin repeats (type 1 and type 1-like), transmembrane domain (TM) and short cytoplasmic domain, (semaphorin) 5A (SEMA5A), mRNA.
NM_153000.3	APCDD1	4.814	Homo sapiens adenomatosis polyposis coli down-regulated 1 (APCDD1), mRNA.
NM_001040002.1	MEOX1	2.285	Homo sapiens mesenchyme homeobox 1 (MEOX1), transcript variant 3, mRNA.
NM_018192.2	LEPREL1	2.070	Homo sapiens leprecan-like 1 (LEPREL1), mRNA.
NM_144778.2	MBNL2	3.224	Homo sapiens muscleblind-like 2 (Drosophila) (MBNL2), transcript variant 1, mRNA.
NM_001677.3	ATP1B1	2.694	Homo sapiens ATPase, Na+/K+ transporting, beta 1 polypeptide (ATP1B1), transcript variant 1, mRNA.
NM_015430.2	PAMR1	5.812	Homo sapiens peptidase domain containing associated with muscle regeneration 1 (PAMR1), transcript variant 1, mRNA.
NM_025216.2	WNT10A	3.007	Homo sapiens wingless-type MMTV integration site family, member 10A (WNT10A), mRNA.
NM_014059.2	C13orf15	9.160	Homo sapiens chromosome 13 open reading frame 15 (C13orf15), mRNA.
NM_152369.2	SLC44A3	2.775	Homo sapiens solute carrier family 44, member 3 (SLC44A3), mRNA.
NM_005159.4	ACTC1	2.892	Homo sapiens actin, alpha, cardiac muscle 1 (ACTC1), mRNA.
NM_006456.1	ST6GALNAC2	2.425	Homo sapiens ST6 (alpha-N-acetyl-neuraminyl-2,3-beta-galactosyl-1, 3)-N-acetylgalactosaminide alpha-2,6-sialyltransferase 2 (ST6GALNAC2), mRNA.
NM_002291.1	LAMB1	3.952	Homo sapiens laminin, beta 1 (LAMB1), mRNA.
NM_001233.3	CAV2	2.893	Homo sapiens caveolin 2 (CAV2), transcript variant 1, mRNA.
NM_006516.1	SLC2A1	2.950	Homo sapiens solute carrier family 2 (facilitated glucose transporter), member 1 (SLC2A1), mRNA.
NM_022343.2	GLIPR2	2.883	Homo sapiens GLI pathogenesis-related 2 (GLIPR2), mRNA.
NM_198552.1	FAM89A	12.036	Homo sapiens family with sequence similarity 89, member A (FAM89A), mRNA.

NM_003836.4	DLK1	20.348	Homo sapiens delta-like 1 homolog (Drosophila) (DLK1), mRNA.
NM_001845.4	COL4A1	4.620	Homo sapiens collagen, type IV, alpha 1 (COL4A1), mRNA.
NM_016938.2	EFEMP2	3.898	Homo sapiens EGF-containing fibulin-like extracellular matrix protein 2 (EFEMP2), mRNA.
NM_021170.2	HES4	4.138	Homo sapiens hairy and enhancer of split 4 (Drosophila) (HES4), mRNA.
NM_001466.2	FZD2	2.503	Homo sapiens frizzled homolog 2 (Drosophila) (FZD2), mRNA.
NM_005562.1	LAMC2	2.479	Homo sapiens laminin, gamma 2 (LAMC2), transcript variant 1, mRNA.
NM_032873.3	STS-1	7.843	Homo sapiens Cbl-interacting protein Sts-1 (STS-1), mRNA.
NM_001031694.1	SCMH1	2.369	Homo sapiens sex comb on midleg homolog 1 (Drosophila) (SCMH1), transcript variant 1, mRNA.
NM_182801.1	EGFLAM	27.065	Homo sapiens EGF-like, fibronectin type III and laminin G domains (EGFLAM), transcript variant 4, mRNA.
NM_152240.1	ZMAT3	2.905	Homo sapiens zinc finger, matrin type 3 (ZMAT3), transcript variant 2, mRNA.
NM_024015.3	HOXB4	2.383	Homo sapiens homeo box B4 (HOXB4), mRNA.
NM_015149.3	RGL1	3.901	Homo sapiens ral guanine nucleotide dissociation stimulator-like 1 (RGL1), mRNA.
NM_004429.3	EFNB1	4.428	Homo sapiens ephrin-B1 (EFNB1), mRNA.
NM_001198.2	PRDM1	2.291	Homo sapiens PR domain containing 1, with ZNF domain (PRDM1), transcript variant 1, mRNA.
NM_152321.1	ERP27	25.526	Homo sapiens endoplasmic reticulum protein 27 kDa (ERP27), mRNA.
NM_021137.3	TNFAIP1	2.682	Homo sapiens tumor necrosis factor, alpha-induced protein 1 (endothelial) (TNFAIP1), mRNA.
NM_006216.2	SERPINE2	3.273	Homo sapiens serpin peptidase inhibitor, clade E (nexin, plasminogen activator inhibitor type 1), member 2 (SERPINE2), mRNA.
NM_001008844.1	DSP	3.569	Homo sapiens desmoplakin (DSP), transcript variant 2, mRNA.
NM_006455.2	SC65	2.234	Homo sapiens synaptonemal complex protein SC65 (SC65), mRNA.
NM_003068.3	SNAI2	14.582	Homo sapiens snail homolog 2 (Drosophila) (SNAI2), mRNA.
NM_000494.3	COL17A1	7.738	Homo sapiens collagen, type XVII, alpha 1 (COL17A1), mRNA.
NM_001004439.1	ITGA11	3.316	Homo sapiens integrin, alpha 11 (ITGA11), mRNA.
NM_014755.1	SERTAD2	2.709	Homo sapiens SERTA domain containing 2 (SERTAD2), mRNA.
NM_000582.2	SPP1	2.758	Homo sapiens secreted phosphoprotein 1 (SPP1), transcript variant 2, mRNA.
NM_080430.2	SELM	2.487	Homo sapiens selenoprotein M (SELM), mRNA.
NM_153705.4	KDELC2	3.936	Homo sapiens KDEL (Lys-Asp-Glu-Leu) containing 2 (KDELC2), mRNA.
NM_003596.2	TPST1	2.366	Homo sapiens tyrosylprotein sulfotransferase 1 (TPST1), mRNA.
NM_001421.2	ELF4	6.136	Homo sapiens E74-like factor 4 (ets domain transcription factor) (ELF4), mRNA.
NM_018890.2	RAC1	3.098	Homo sapiens ras-related C3 botulinum toxin substrate 1 (rho family, small GTP binding protein Rac1) (RAC1), transcript variant Rac1b, mRNA.

Supplementary table 6: Genes down regulated in Day 12 as compared to Day 20

Refseq_NM	Gene Symbol	D20/D12.fc	DEFINITION
NM_002341.1	LTB	-6.002494	Homo sapiens lymphotoxin beta (TNF superfamily, member 3) (LTB), transcript variant 1, mRNA.

Supplementary table 7: Genes up regulated in Day 20 as compared to Day 12

Refseq_NM	GeneSymbol	D20/D12.fc	DEFINITION
NM_003836.4	DLK1	13.44694	Homo sapiens delta-like 1 homolog (Drosophila) (DLK1), mRNA.
NM_003836.4	DLK1	12.544575	Homo sapiens delta-like 1 homolog (Drosophila) (DLK1), mRNA.
NM_000900.2	MGP	12.288249	Homo sapiens matrix Gla protein (MGP), mRNA.
NM_015507.2	EGFL6	11.061904	Homo sapiens EGF-like-domain, multiple 6 (EGFL6), mRNA.
NM_001855.3	COL15A1	9.711415	Homo sapiens collagen, type XV, alpha 1 (COL15A1), mRNA.
NM_015507.2	EGFL6	8.986263	Homo sapiens EGF-like-domain, multiple 6 (EGFL6), mRNA.
NM_001048.3	SST	7.557473	Homo sapiens somatostatin (SST), mRNA.
NM_001463.2	FRZB	6.754676	Homo sapiens frizzled-related protein (FRZB), mRNA.
NM_178565.3	RSPO2	4.867798	Homo sapiens R-spondin 2 homolog (Xenopus laevis) (RSPO2), mRNA.
NM_005950.1	MT1G	4.821604	Homo sapiens metallothionein 1G (MT1G), mRNA.
NM_003613.2	CILP	4.790009	Homo sapiens cartilage intermediate layer protein, nucleotide pyrophosphohydrolase (CILP), mRNA.
NM_004887.3	CXCL14	4.749055	Homo sapiens chemokine (C-X-C motif) ligand 14 (CXCL14), mRNA.
NM_002404.1	MFAP4	4.65037	Homo sapiens microfibrillar-associated protein 4 (MFAP4), mRNA.
NM_004503.3	HOXC6	4.641027	Homo sapiens homeobox C6 (HOXC6), transcript variant 1, mRNA.
NM_022658.3	HOXC8	4.57334	Homo sapiens homeobox C8 (HOXC8), mRNA.
NM_001854.3	COL11A1	4.457517	Homo sapiens collagen, type XI, alpha 1 (COL11A1), transcript variant A, mRNA.
NM_182801.1	EGFLAM	4.015794	Homo sapiens EGF-like, fibronectin type III and laminin G domains (EGFLAM), transcript variant 4, mRNA.
NM_133505.2	DCN	3.41316	Homo sapiens decorin (DCN), transcript variant C, mRNA.
